# A Heteroleptic Dirhodium Catalyst for Asymmetric Cyclopropanation with α‐Stannyl α‐Diazoacetate. “Stereoretentive” Stille Coupling with Formation of Chiral Quarternary Carbon Centers

**DOI:** 10.1002/anie.202004377

**Published:** 2020-06-04

**Authors:** Fabio P. Caló, Alois Fürstner

**Affiliations:** ^1^ Max-Planck-Institut für Kohlenforschung 45470 Mülheim/Ruhr Germany

**Keywords:** asymmetric catalysis, cyclopropanation, heteroleptic complexes, quarternary chiral centers, rhodium carbenes, Stille coupling

## Abstract

The heteroleptic dirhodium paddlewheel catalyst **7** with a chiral carboxylate/acetamidate ligand sphere is uniquely effective in asymmetric [2+1] cycloadditions with α‐diazo‐α‐trimethylstannyl (silyl, germyl) acetate. Originally discovered as a trace impurity in a sample of the homoleptic parent complex [Rh_2_((*R*)‐TPCP)_4_] (**5**), it is shown that the protic acetamidate ligand is quintessential for rendering **7** highly enantioselective. The ‐NH group is thought to lock the ensuing metal carbene in place via interligand hydrogen bonding. The resulting stannylated cyclopropanes undergo “stereoretentive” cross coupling, which shows for the first time that even chiral quarternary carbon centers can be made by the Stille–Migita reaction.

## Graphical abstract

**Less is more**: Take one chiral ligand from a standard paddlewheel dirhodium tetracarboxylate complex out and replace it by acetamidate and you switch on reactivity and harness outstanding enantioselectivity in cyclopropanations with α‐stannylated‐α‐diazoesters. Even though the products contain a *tert*‐alkylstannane moiety, “stereoretentive” Stille cross coupling is shown to be possible.

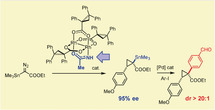

## Introduction

**Scheme 1:**
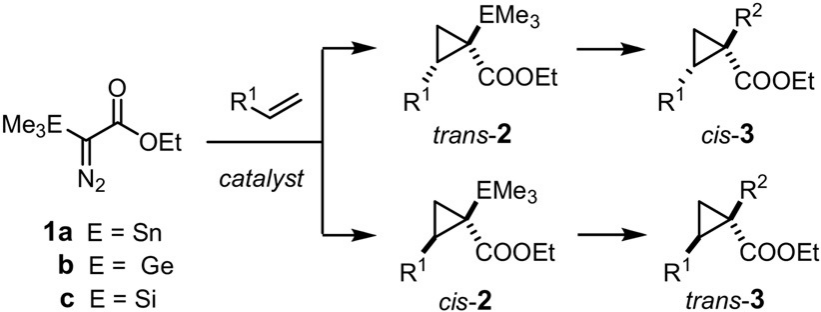
Conceptual outline.

During the course of our investigations into (chiral) metal carbene complexes,^1^, ^2^, ^3^, ^4^, ^5^, ^6^ we became aware that reactions of silylated, germylated or stannylated α‐diazoacetate derivatives **1** largely fail to meet the standards of modern asymmetric catalysis. Substrates of this type are easy to make on multigram scale and safe to handle;^7^, ^8^ the derived transition metal carbenes are known to be well‐behaved intermediates in cyclopropanation and C−H insertion reactions, to mention but a few.^9^, ^10^, ^11^, ^12^ Yet, highly enantioselective versions are basically unknown,^13^ except for a single report of an intramolecular case.^14^ This methodological gap is all the more regrettable as the resulting products featuring an ester and a metalloid center next to each other provide ample opportunity for downstream manipulation. In this context, stannylated (silylated) cyclopropanes **2** are deemed particularly relevant (Scheme 1),^15^ not least because of the rapidly increasing demand of contemporary medicinal as well as natural product chemistry for small‐ring systems.^16^ Provided that the tertiary alkylstannane moiety of **2 a** (E=Sn) can be engaged in cross coupling—which in itself is a highly challenging transformation—such building blocks should open access to products **3** and surrounding chemical space that can be difficult to reach otherwise.

## Results and Discussion

**Catalyst Development**. In a first attempt to meet the challenge, a series of standard chiral dirhodium tetracarboxylate catalysts was screened in reactions with the readily available stannylated ester **1 a** (for details, see the SI).^8^ [Rh_2_((*R*)‐TPCP)_4_] (**5**)^17^, ^18^ gave the only notable “hit”, but the outcome proved extremely erratic when different batches of this catalyst were used. This puzzling situation suggested that minor impurities might massively interfere with the results.

**Figure 1:**
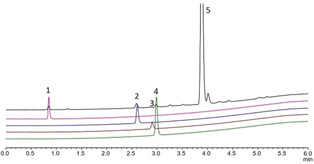
Representative HPLC trace of a sample of **5** (=fraction 5) prepared by method B; fraction 1 is unreacted acid **4**; for the other fractions, see Text.

**Figure 2:**
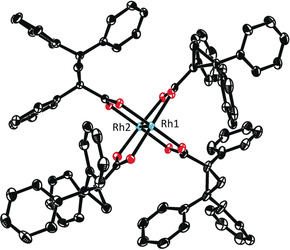
Structure of [Rh_2_((*R*)‐TPCP)_4_]⋅2 MeCN (**5**⋅2 MeCN) in the solid state; coordinated and solute MeCN is removed and H‐atoms are omitted for clarity. The entire structure is shown in the SI, which also contains a second crystal structure of the same complex in a different space group.

**Scheme 2:**
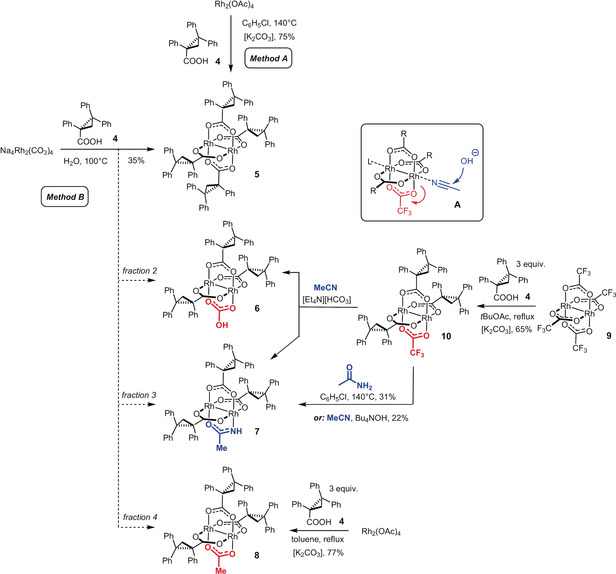
Formation of the homoleptic complex **5** and by‐products derived from impurities in different samples of Na_4_Rh_2_(CO_3_)_4_, cf. Text; targeted syntheses of heteroleptic siblings.

Therefore we embarked into a more systematic investigation and prepared samples of this catalyst by two different routes (Scheme 2): Method A reacts [Rh_2_(OAc)_4_] with acid **4** in refluxing chlorobenzene. The ligand exchange is driven to completion by passing the high‐boiling solvent through a Soxlet extractor filled with K_2_CO_3_,^19^ which traps the released HOAc. All samples of **5** prepared in this manner were essentially pure (NMR, HPLC) but invariably inactive in the model reaction (Table 1, entry 1). The structure of **5** in the solid state shows quasi‐*C*_2_ symmetric binding sites about the Rh atoms, which might be too narrow to accommodate the stannylated diazoester (Figure 2).^20^ Method B reacts Na_4_[Rh_2_(CO_3_)_4_]⋅2.5 (H_2_O) with acid **4** in boiling water according to the literature.^17^ In this case, the catalyst samples were slightly less clean as evident from a representative HPLC trace which shows several minor impurities in addition to a small amount of free ligand **4** (Figure 1). Such samples led to highly variable but occasionally excellent *ee*’s; after HPLC separation, the pure sample of **5** (>99 %) again failed to catalyze the test reaction (entries 2/3). Three additional fractions were collected, delivering minuscule amounts of unknown rhodium‐containing species: fraction 2 decomposed when kept in CD_3_CN solution, but the two other samples could be tested despite the minute available quantities. Fraction 4 gave only modest asymmetric induction, whereas the seemingly negligible fraction 3 furnished the stannylated cyclopropanes *cis*‐**2 aa** and *trans*‐**2 aa** with ≥95 % *ee* in what appeared to be a fast and clean transformation (entries 4/6).

**Table 1:** Screening of different catalysts (for the full list, see the SI).^[a]^ 
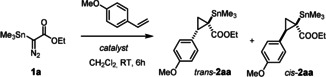

Entry	Catalyst	*ee* [%]		Yield [%]^[b]^
		*trans*‐**2 aa**	*cis*‐**2 aa**	
1	**5** (method A)	–	–	NR
2	**5** (crude, method B)	up to 92	up to 96	up to quant.^[c]^
3	**5** (>99 % pure, method B)	–	–	NR
4	fraction 4	54	76	n. d.
5	**8**	53	76	56
6	fraction 3	96	97	n.d.
7	**7**	95	97	76
8	**10**	30	53	n.d.
9	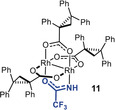	93	95	44
10	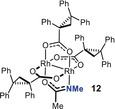	7	39	48

[a] In all entries, the *cis:trans* ratio was ≈1:1. [b] Yield of isolated material, unless stated otherwise. [c] NMR yield; NR=no reaction; n.d.=not determined.

At this point, incomplete replacement of the carbonate ligands of Na_4_[Rh_2_(CO_3_)_4_] by the chiral acid **4** was deemed the most plausible explanation for the formation of these minor by‐products. To the best of our knowledge, only a single chiral heteroleptic paddlewheel complex comprising hydrogen carbonate groups is known in the literature; its exact structure, however, is unclear and the catalytic performance not fully convincing.^21^, ^22^ This specific case notwithstanding, it seemed reasonable that a heteroleptic dirhodium complex might outperform its homoleptic cousin **5** in certain applications; a few such cases are known in the literature.^23^ For the lack of good and broadly applicable strategies for the controlled introduction of two or more different (chiral) ligands about the Rh_2_‐core, systematic explorations of dirhodium complexes with mixed ligand spheres remain difficult. In line with this notion, our attempts at partial substitution of the carbonate ligands of Na_4_[Rh_2_(CO_3_)_4_] by **4** basically met with failure, despite considerable experimentation: **5** was the main product independent of the chosen ligand/rhodium ratio and the experimental conditions. The reverse approach, that is partial replacement of the chiral ligands in **5** on reaction with various carbonate sources, was equally unrewarding.

Next, we turned out attention to common dirhodium tetracarboxylates as the point of departure. A first important step was taken when we learnt that only three of the four acetate units of [Rh_2_(OAc)_4_] are substituted by **4** when three equivalents of **4** are employed and the reaction is performed in boiling toluene (Soxhlet method). Unexpectedly, the heteroleptic complex **8** thus formed in 77 % yield proved identical with fraction 4, which is catalytically active but only modestly selective. This result implied that the Na_4_[Rh_2_(CO_3_)_4_] sample used to make **5** must have contained some rhodium acetate impurity.^24^ Three‐fold ligand exchange also worked well with [Rh_2_(tfa)_4_] (**9**, tfa=trifluororacetate), provided that ethyl acetate^25^ or, preferentially, the higher boiling *tert*‐butyl acetate was used as the solvent to furnish **10** in 65 % yield.^26^ Complex **10** was then treated with [Et_4_N][HCO_3_] in MeCN: despite the presumably better leaving group properties of trifluoroacetate, the reaction was again inefficient and furnished *two* new products, which correspond to fraction 2 and the sought‐after fraction 3. The instability of the former in solution (see above) precluded full characterization; yet, a resonance in the ^13^C NMR spectrum at δ_C_=165.2 ppm, a cluster of indicative MS signals,^27^ and the fact that the material responds to treatment with acid/base render the assignment as the heteroleptic mono‐hydrogencarbonate complex **6** highly likely.

The HRMS data of the relevant complex contained in fraction 3 were suggestive: one of the recorded signals at *m*/*z*=1203.20886 matched the composition [C_68_H_55_O_7_NRh_2_] very well, which can be interpreted as {[Rh_2_((*R*)‐TPCP)_3_] + MeCN + OH]}. If traces of MeCN had been contained in the sample of Na_4_[Rh_2_(CO_3_)_4_], it was almost certainly ligated to the axial sites at Rh. As this renders the nitrile group susceptible to base, trace acetamide could have been generated in situ within the first coordination sphere (**A** in Scheme 2), which might replace the trifluoroacetate group of **10** and give rise to the enigmatic “fraction 3”.^28^, ^29^, ^30^ With this idea in mind, we pursued two targeted approaches to the presumed heteroleptic acetamidate complex [Rh_2_((*R*)‐TPCP)_3_(acam)] (**7**): to this end, [Et_4_N][HCO_3_] was replaced by Bu_4_NOH in the reaction with **10** in MeCN, which indeed raised the yield of **7** to 22 % after ordinary flash chromatography. Alternatively, treatment of **10** with acetamide in refluxing chlorobenzene (Soxhlet method)^19^ furnished **7** in 31 % yield.^31^

**Figure 3:**
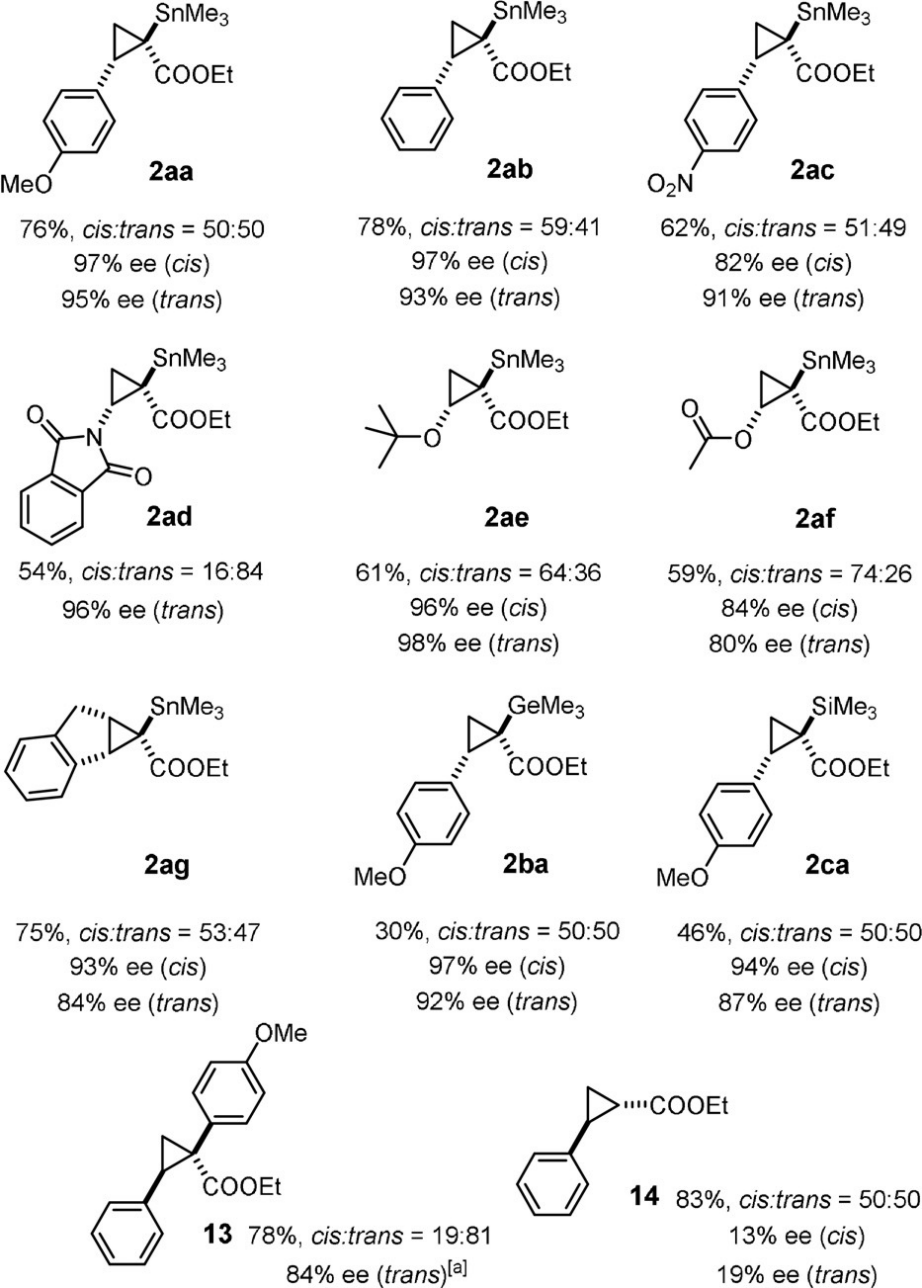
Substrate scope (only the *trans*‐isomer is drawn); unless stated otherwise, all reactions were performed with catalyst **7** (1 mol %) in CH_2_Cl_2_ at ambient temperature; [a] at −78 °C.

**Scope**. Control experiments confirmed that complex **7** is indeed an active and highly enantioselective catalyst for the model cyclopropanation reaction of **1 a** with 4‐methoxystyrene; the missing diastereoselectivity is somehow compensated by the ease of separation of *cis*‐**2 aa** (97 % *ee*) and *trans*‐**2 aa** (95 % *ee*) by flash chromatography. The examples compiled in Figure 3 allow the scope of the reaction to be assessed: styrene derivatives afforded the desired stannylated cyclopropane derivatives in generally excellent optical purity, independent of whether they are electron‐rich or ‐poor. Enamides, enol esters and enol ethers are equally suitable substrates: for the three different functional groups, the resulting products **2 ad**–**2 af** are deemed particularly interesting building blocks. Even though this study was mainly focused on the preparation of stannylated cyclopropanes as our premier candidates for downstream functionalization, it was found that the corresponding silylated and germylated products **2 ba** and **2 ca** are formed with similarly high *ee*’s. The standard donor/acceptor carbene precursor *p*‐MeOC_6_H_4_C(N_2_)COOEt, however, gave cyclopropane **13** with only 57 % *ee*; gratifyingly, the high reactivity of the new catalyst allowed the temperature to be lowered to −78 °C and the outcome to be improved to respectable 84 % *ee*.^32^ The parent ethyl diazoacetate, in contrast, was found to react well but furnished **14** with poor selectivity. These preliminary data suggest that a moderately bulky substituent at the carbene site is mandatory in order to reach high levels of asymmetric induction in reactions catalyzed by **7**;^33^ this aspect is subject to further investigation in our laboratory.

**Figure 4:**
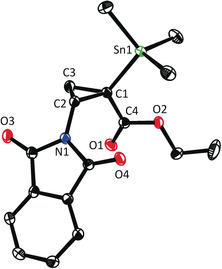
Structure of compound (1*R*,2*R*)‐**2 ad** in the solid state; hydrogen atoms omitted for clarity; only one of four independent molecules in the unit cell is shown; for the whole structure, see the SI.

The sense of induction was rigorously established by X‐ray diffraction for two independent cases. Statistically significant absolute structure parameters were obtained that allowed the configuration of the stannylated cyclopropanes **2 ac** (see the SI) and **2 ad** (Figure 4) to be determined, which derive from an electron‐deficient and an electron‐rich alkene, respectively. In both products the substituents on the three‐membered ring have the same orientation in space, even though the correct denomination is different because of the formalism of the CIP‐notation ((1*R*,2*S*)‐**2 ac** but (1*R*,2*R*)‐**2 ad**). All other compounds were assigned by analogy.^32^

**Mechanistic Aspects**. As yet another important prelude for a mechanistic discussion, the exact role of the acetamidate ligand in **7** was examined. As already mentioned above, the heteroleptic complex **8** carrying an acetate reacts well but is much less enantioselective (Table 1, entry 5). The same disparate behavior was observed for the pair **10** and **11** comprising a trifluoroacetate and a trifluoroacetamidate, respectively: only the latter proved to be highly enantioselective (entries 8/9). Equally relevant is the control experiment with complex **12**, which differs from **7** in that its acetamide ligand is N‐methylated:^34^ the level of asymmetric induction is marginal (entry 10). Taken together, these results suggest that the heteroleptic character accounts for the reactivity of the complexes, but the *protic* ligand plays a quintessential role in the enantiodeterming step.

**Figure 5:**
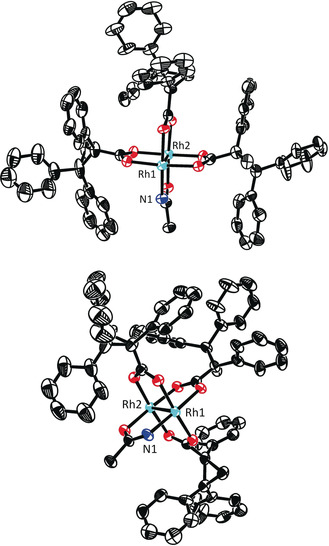
Structure of [Rh_2_((*R*)‐TPCP)_3_(acam)]⋅2 DMF (**7**⋅2 DMF) in the solid state in two different orientations; the axial DMF ligands were removed for a better view onto the binding site about Rh1, to which the N‐atom of the acetamidate ligand is coordinated; hydrogen atoms are omitted for clarity. The full structure is contained in the SI.

This information has to guide the inspection of the structure of [Rh_2_((*R*)‐TPCP)_3_(acam)] (**7**) in the solid state. Crystals of good quality were obtained for an adduct carrying two molecules of DMF at the axial sites (Figure 5). In comparison with the structure of the homoleptic parent complex [Rh_2_((*R*)‐TPCP)_4_] (**5**) (Figure 2), it is apparent that the incorporation of one small ligand leads to a significantly wider binding site. Since the size of an oxygen atom and an ‐NH group are similar, the binding pockets of complexes **8** and **10** are almost certainly akin. Therefore all heteroleptic complexes should be able to accommodate fairly bulky incoming diazo derivatives,^33^ whereas the homoleptic complex **5** (Figure 2) is not; this notion is in accord with the experimental reactivity data.

The ‐NH group constitutes the critical determinant for high selectivity. The effect that it imparts, however, cannot be steric in origin: Figure 5 shows that Rh1 and Rh2 of **7** are both well accessible. A purely electronic argument is equally unlikely: in consideration of the well‐founded trend that amidate ligands tend to render dirhodium catalysts less reactive (but often very selective),^29^ one might assume that carbene formation occurs preferentially or exclusively at Rh2 surrounded by the four O‐atoms. If this were the case, however, complexes **7** and **12** differing only in the substituent on the acetimidate N‐atom (NH versus NMe) should lead to similar levels of asymmetric induction; experimentally, the outcome is dramatically different (Table 1, entries 7/10). The fact that **12** with the more bulky N‐substituent is also chemically somewhat less effective also speaks for diazo‐decomposion occuring at the N‐containing binding site. Moreover, if the reaction takes place at an all‐oxygen coordinated Rh‐center, the (trifluoro)acetate‐containing complexes **8** and **10** comprising two essentially equivalent such binding sites should be highly selective too, which is clearly not the case.

**Figure 6:**
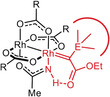
Possible rationale: interligand hydrogen bonding.

These facts and arguments suggest that Rh1 is the relevant reaction center that reigns the asymmetric process. We assume that the NH‐group plays an *active role* that outweighs any electronic handicap: the protic ligand might engage the diazocarbonyl derivative **1** in intermolecular hydrogen bonding and hence recruite the substrate to this site. Once it is bound and nitrogen extruded, the then intramolecular hydrogen bonding array locks the resulting carbene in place within the chiral binding pocket, such that it eclipses the O‐Rh‐N axis (Figure 6).^35^, ^36^ Under this proviso, however, the fairly bulky Me_3_E‐ group (E=Si, Ge, Sn) might force the top phenyl ring, which protrudes over the binding site of **7** (Figure 5), to relocate and change the chiral microenvironment. Therefore it seems prudent at this point not to over‐interpret the structure of the precatalyst **7** in the solid state: in any case, it is non‐obvious from this X‐ray structure which enantiotopic face of the carbene is exposed to the reaction partner and which one is shielded. Moreover, it is unclear in this particular case whether the alkene approaches the electrophilic carbene alongside the R_3_E‐substitutent or the ester;^37^ these and related aspects are subject to ongoing investigations. The largely missing diastereoselectivity, however, means that **7** fails to determine the orientation of the incoming olefin, which is plausible for a catalyst with a fairly wide binding site.

**Scheme 3:**
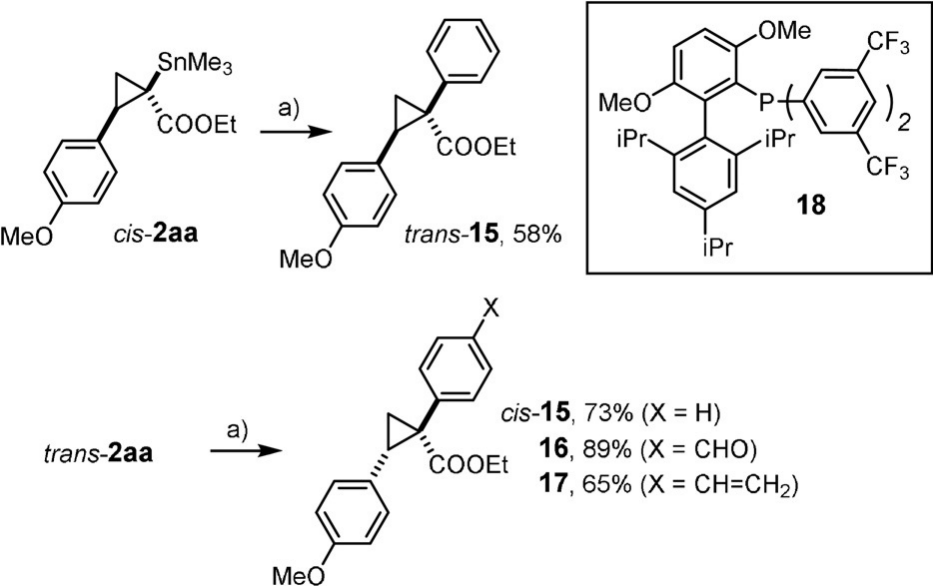
a) Pd(dba)_2_ (10 mol %), JackiePhos (**18**) (20 mol %), CuCl, KF, THF, 60 °C.

**Cross Coupling. Formation of Chiral Quarternay Carbon Centers**. Bifunctional cyclopropanes of type **2** bearing a metalloid center adjacent to an ester open many possibilities for downstream functionalization. Even though Stille coupling of *tert*‐alkylstannanes with formation of stereogenic quarternary carbon had been unknown at the outset of our investigation,^15^, ^38^ it seemed promising to pursue this tantalizing prospect in view of the special bonding situation in cyclopropanes (Walsh orbitals); in case of **2** one can also think of this transformation as an α‐arylation process.^39^ However, the generation of tin enolates by facile C→O migration of the Me_3_Sn‐group with concomitant planarization of the chiral center must be strictly avoided; premature protodestannation is yet another serious threat. The prototype examples shown in Scheme 3 illustrate that these challenges can indeed be met using conditions previously developed for the cross coupling of secondary azastannatranes:^40^ *cis*‐**2 aa** and *trans*‐**2 aa** were coupled with iodobenzene in appreciable yield and perfect integrity of the stereocenter as manifest in a dr >20:1 (NMR, HPLC) in both cases.^41^ While product **15** could certainly be made directly by asymmetric cyclopropanation via a convenitonal donor/acceptor carbene,^1^ the new cross‐coupling approach provides additional opportunities as illustrated by the formation of **16** and **17** comprising a terminal alkene and an aldehyde, respectively: either functionality is incompatible with a transient carbene intermediate. As many more such examples reaching beyond the traditional scope can be envisaged, these promising results mark just the starting point of a more comprehensive study in our laboratory.

## Conclusion

The present report rigorously exemplifies that the switch from a homoleptic to a heteroleptic ligand sphere about a dirhodium core can unlock entirely new reactivity and selectivity in carbene chemistry; the effect per se is known, but equally striking cases are exceedingly rare. For more systematic forays into this promising area, however, innovative new concepts and techniques are deemed vital that allow heteroleptic complexes to be crafted in a (more) rational and productive manner. At the same time, catalyst **7** is thought to showcase the power of interligand hydrogen bonding in catalysis.^36^ Finally, we note that the conclusions of this detective story may arguably be of conceptual relevance in that the success hinges on a ligand that plays an active role rather than being solely a passive divider of (chiral) space.^42^ This notion guides our future investigations in the field.

## Conflict of interest

The authors declare no conflict of interest.

## Supporting information

As a service to our authors and readers, this journal provides supporting information supplied by the authors. Such materials are peer reviewed and may be re‐organized for online delivery, but are not copy‐edited or typeset. Technical support issues arising from supporting information (other than missing files) should be addressed to the authors.

Supplementary
