# Supplementary material for: A Heteroleptic Dirhodium Catalyst for Asymmetric Cyclopropanation with α‐Stannyl α‐Diazoacetate. “Stereoretentive” Stille Coupling with Formation of Chiral Quarternary Carbon Centers
Source: Angew Chem Int Ed Engl. 2020 Jun 4;59(33):13900–7. doi: 10.1002/anie.202004377 (PMC7496581; doi:10.1002/anie.202004377)

Angewandte Chemie
Eine Zeitschrift der Gesellschaft Deutscher Chemiker

Supporting Information

A Heteroleptic Dirhodium Catalyst for Asymmetric Cyclopropanation with α-Stannyl α-Diazoacetate. "Stereoretentive" Stille Coupling with Formation of Chiral Quarternary Carbon Centers

*Fabio P. Caló and Alois Fürstner**

anie_202004377_sm_miscellaneous_information.pdf

SUPPORTING CRYSTALLOGRAPHIC INFORMATION

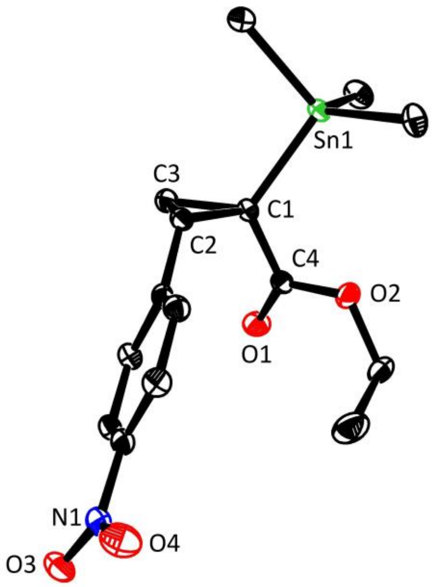

Figure S-1. Structure of compound (1*R*,2*S*)-**2ac** in the solid state; hydrogen atoms omitted for clarity

X-ray Crystal Structure Analysis of Compound 2ac: $C_{15}H_{21}NO_4Sn$, M_r = 398.02 g mol^{-1}, colorless needle, crystal size 0.30 x 0.03 x 0.03 mm^3, orthorhombic, space group $P2_12_12_1$ [No. 19], *a* = a = 6.0363(4) Å, *b* = 15.8101(18) Å, *c* = 17.699(3) Å, *V* = 1689.1(3) Å^3, *T* = 100(2) K, *Z* = 4, D_{calc} = 1.565 g cm^{-3}, λ = 0.71073 Å, μ(*Mo-K$_\alpha$*) = 1.526 mm^{-1}, analytical absorption correction (T_{min} = 0.79, T_{max} = 0.96), Bruker-AXS Kappa Mach3 diffractometer with APEX-II detector and rotating anode X-ray source, 2.638 < θ < 33.139°, 35547 measured reflections, 6432 independent reflections, 6069 reflections with $I > 2\sigma(I)$, R_{int} = 0.0359. The structure was solved by dual space methods (*SHELXT*) and refined by full-matrix least-squares (*SHELXL*) against F^2 to R_1 = 0.021 [$I > 2\sigma(I)$], wR_2 = 0.048, 194 parameters, absolute structure parameter using Flack's method = –0.034(9), residual electron density 0.4 (0.62 Å from C2)/ –1.5 (0.73 Å from Sn1) e · Å^{-3}. **CCDC-1990587**.

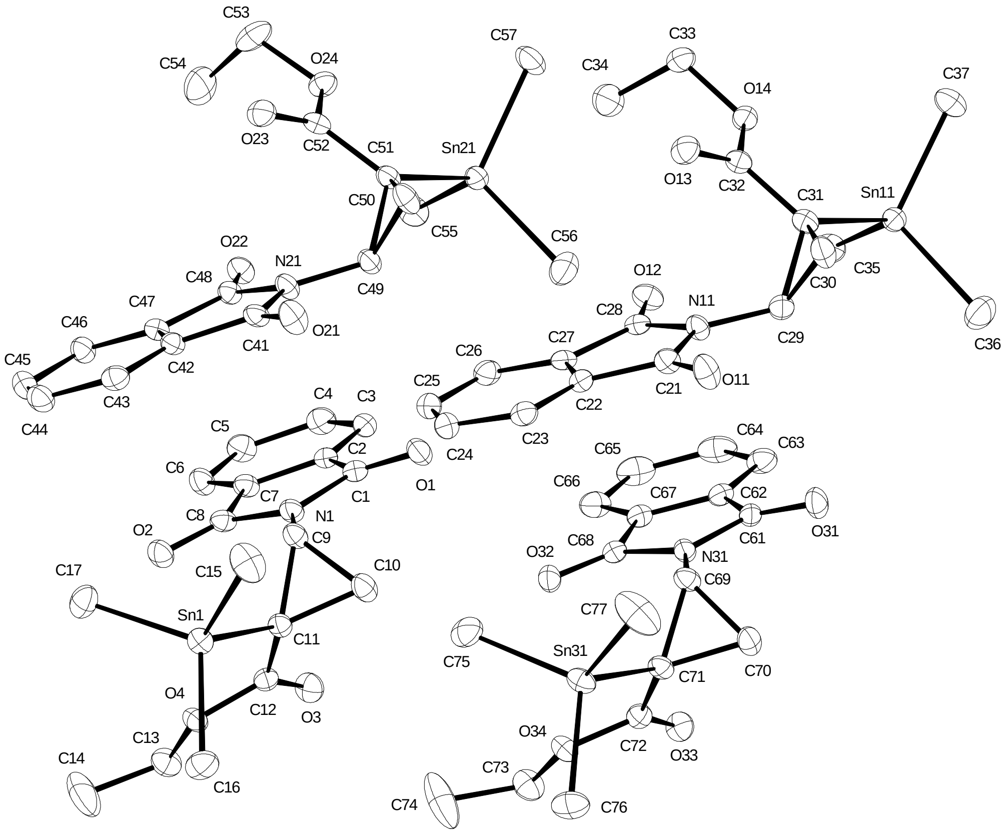

Figure S-2. Structure of compound (1*R*,2*R*)-**2ad** in the solid state; hydrogen atoms omitted for clarity

X-ray Crystal Structure Analysis of Compound 2ad: $C_{17} H_{21} N O_4 Sn$, M_r = 422.04 g mol^{-1}, colorless prism, crystal size 0.22 x 0.07 x 0.06 mm^3, orthorhombic, space group $P2_12_12_1$ [No. 19], a = a = 7.9978(6) Å, b = 29.510(3) Å, c = 30.4972(18) Å, V = 7197.7(10) Å^3, T = 100(2) K, Z = 16, D_{calc} = 1.558 g cm^{-3}, λ = 0.71073 Å, $\mu(Mo\text{-}K_{\alpha})$ = 1.438 mm^{-1}, analytical absorption correction ($T_{\min}$ = 0.76, $T_{\max}$ = 0.92), Bruker-AXS Kappa Mach3 diffractometer with APEX-II detector and rotating anode X-ray source, 2.841 < θ < 33.186°, 92506 measured reflections, 27396 independent reflections, 22278 reflections with $I > 2\sigma(I)$, R_{int} = 0.0489. The structure was solved by dual space methods (*SHELXT*) and refined by full-matrix least-squares (*SHELXL*) against F^2 to R_1 = 0.035 [$I > 2\sigma(I)$], wR_2 = 0.066, 845 parameters, absolute structure parameter using Flack's method = –0.020(7), residual electron density 0.9 (0.54 Å from Sn21)/ –1.1 (0.75 Å from Sn1) e · Å^{-3}. There is a large unit-cell (c-axis > 30.4 Å long) with four independent molecules in the unit cell. Some low-order reflections have been omitted from the final least-squares refinement. **CCDC- 1990588**.

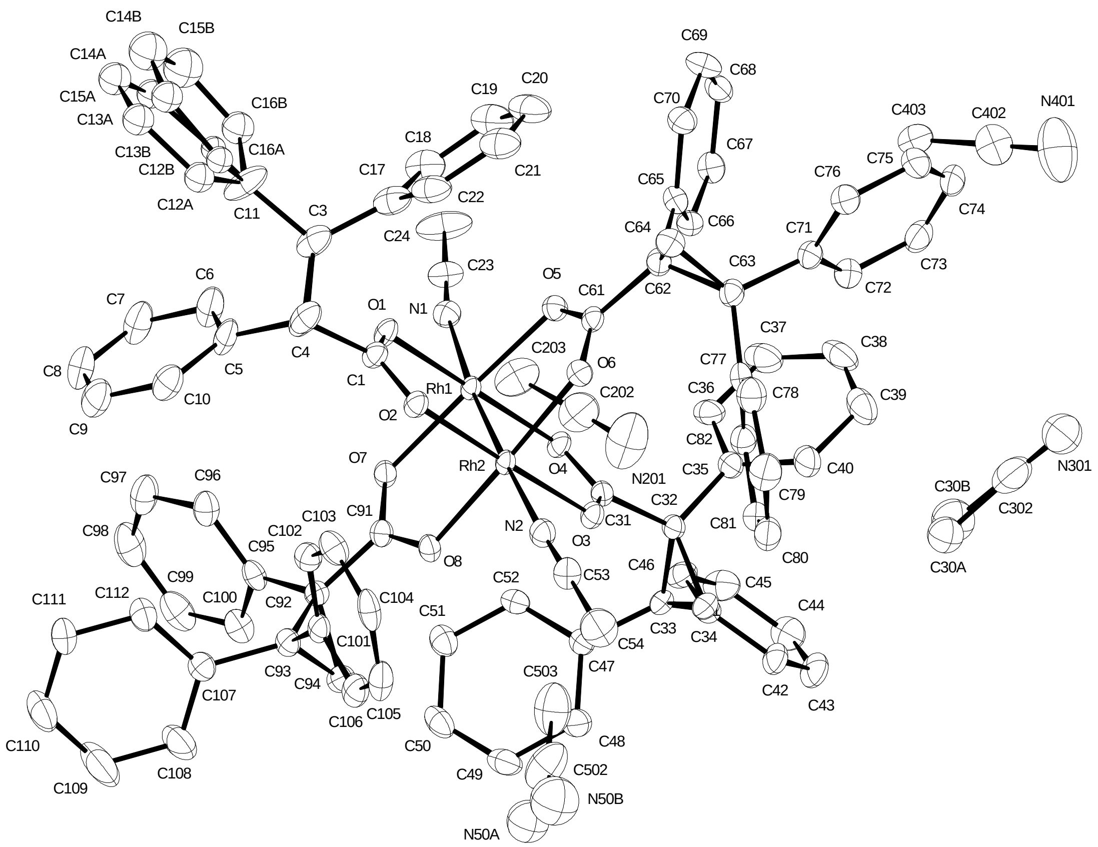

Figure S-3. Structure of complex $[Rh_2((R)\text{-TPCP})_4]\cdot 2MeCN$ (**5**·2MeCN) in the solid state (**orthorhombic, space group $P2_12_12_1$ [No. 19]**); hydrogen atoms omitted for clarity

X-ray Crystal Structure Analysis of Complex [5·2MeCN]: $C_{100}H_{86}N_6O_8Rh_2$, M_r = 1705.56 g mol^{-1}, violet needle, crystal size 0.15 x 0.06 x 0.05 mm^3, orthorhombic, space group $P2_12_12_1$ [No. 19], a = 15.0163(12) Å, b = 22.865(4) Å, c = 27.839(4) Å, V = 9558(2) Å^3, T = 100(2) K, Z = 4, D_{calc} = 1.185 g cm^{-3}, λ = 0.71073 Å, $\mu(Mo\text{-}K_\alpha)$ = 0.400 mm^{-1}, analytical absorption correction ($T_{\min}$ = 0.91, $T_{\max}$ = 0.98), Bruker-AXS Kappa Mach3 diffractometer with APEX-II detector and rotating anode X-ray source, 2.730 < θ < 33.231 °, 200815 measured reflections, 36562 independent reflections, 25384 reflections with $I > 2\sigma(I)$, R_{int} = 0.0960. The structure was solved by dual space methods (*SHELXT*) and refined by full-matrix least-squares (*SHELXL*) against F^2 to R_1 = 0.050 [$I > 2\sigma(I)$], wR_2 = 0.112, 1045 parameters, absolute structure parameter using Flack's method = 0.009(15), residual electron density 0.6 (1.14 Å from Rh2)/ –0.8 (0.73 Å from Rh1) e · Å^{-3}.

The low-angle reflections was shadowed by the beamstop and has been omitted from the data set from the final refinement cycles. **CCDC- 1990590**.

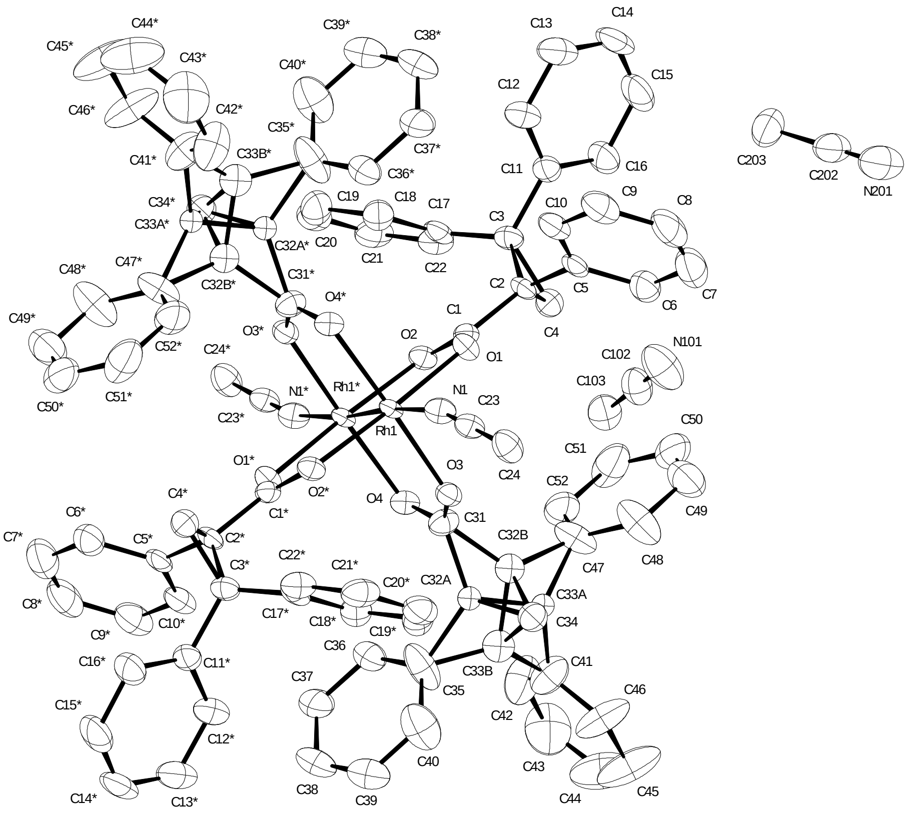

Figure S-4. Structure of complex $[Rh_2((R)\text{-TPCP})_4]\cdot 2MeCN$ (**5**·2MeCN) in the solid state (**triclinic, space group $P\bar{1}$ [No. 2]**); hydrogen atoms omitted for clarity

X-ray Crystal Structure Analysis of Complex [5·2MeCN]: $C_{99}H_{84.50}N_{5.50}O_8Rh_2$, M_r = 1685.04 g mol^{-1}, violet plate, crystal size 0.095 x 0.073 x 0.021 mm^3, triclinic, space group $P\bar{1}$ [No. 2], a = 10.3329(5) Å, b = 14.8860(7) Å, c = 15.1137(8) Å, α = 93.249(3)°, β = 109.844(3)°, γ = 108.573(3)°, V = 2037.08(18) Å^3, T = 100(2) K, Z = 1, D_{calc} = 1.374 g cm^{-3}, λ = 0.71073 Å, $\mu(Mo\text{-}K_\alpha)$ = 0.468 mm^{-1}, analytical absorption correction (T_{min} = 0.97, T_{max} = 0.99), Bruker-AXS Kappa Mach3 diffractometer with APEX-II detector and IµS micro focus, 2.197 < θ < 25.295°, 48368 measured reflections, 7357 independent reflections, 5490 reflections with $I > 2\sigma(I)$, R_{int} = 0.0958. The structure was solved by dual space methods (*SHELXT*) and refined by full-matrix least-squares (*SHELXL*) against F^2 to R_1 = 0.051 [$I > 2\sigma(I)$], wR_2 = 0.112, 524 parameters, residual electron density 0.6 (0.92 Å from Rh1)/ –0.9 (1.01 Å from Rh1) e · Å^{-3}. **CCDC- 1990586**.

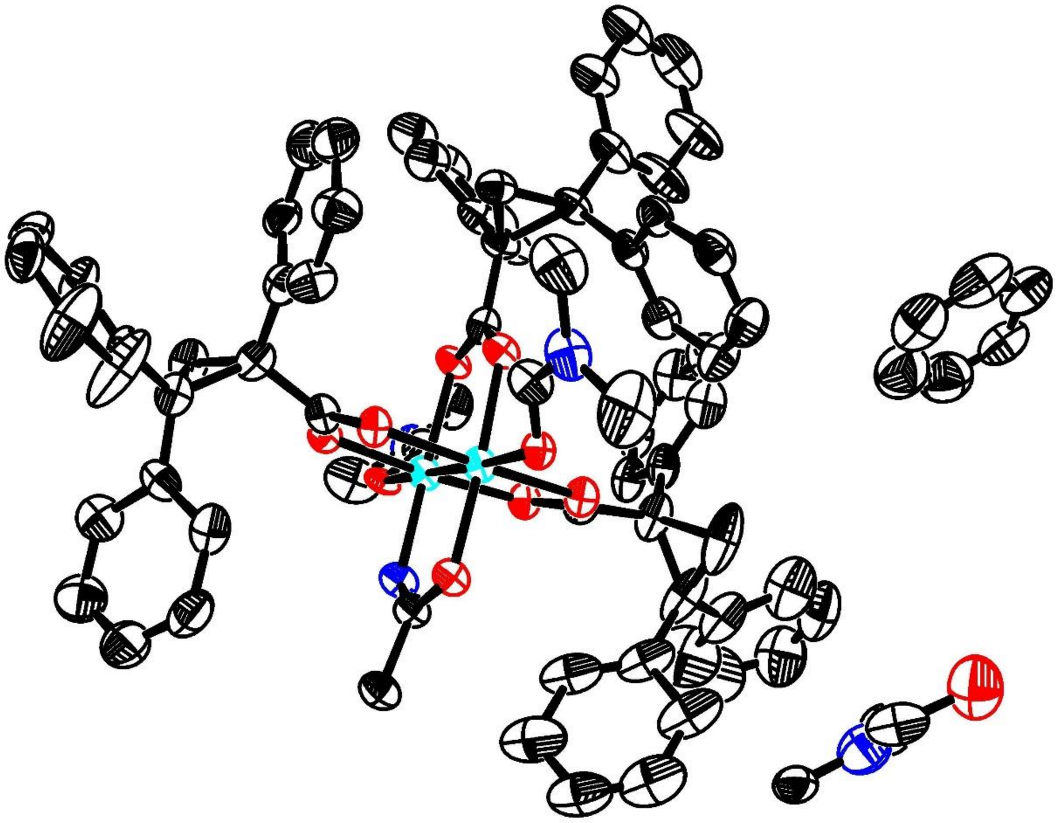

Figure S-5. Structure of [Rh_2((*R*)-TPCP)$_3$(acam)]·2DMF (**7**·2DMF) in the solid state; hydrogen atoms are omitted for clarity.

X-ray Crystal Structure Analysis of Complex [7·2DMF]: $C_{82.25}H_{79.25}N_{3.75}O_{9.75}Rh_2$, M_r = 1482.06 g mol^{-1}, turquoise plate, crystal size 0.200 x 0.062 x 0.040 mm^3, monoclinic, space group $P2_1$ [No. 4], a = 12.700(3) Å, b = 19.823(5) Å, c = 15.550(4) Å, β = 110.780(4)°, V = 3659.9(15) Å^3, T = 100(2) K, Z = 2, D_{calc} = 1.345 g cm^{-3}, λ = 0.71073 Å, μ(Mo-K_α) = 0.512 mm^{-1}, analytical absorption correction (T_{min} = 0.95, T_{max} = 0.99), Bruker-AXS Kappa Mach3 diffractometer with APEX-II detector and and IµS micro focus, 2.717 < θ < 30.508°, 81549 measured reflections, 22180 independent reflections, 12139 reflections with $I > 2\sigma(I)$, R_{int} = 0.1544. The structure was solved by dual space methods (*SHELXT*) and refined by full-matrix least-squares (*SHELXL*) against F^2 to R_1 = 0.064 [$I > 2\sigma(I)$], wR_2 = 0.179, 899 parameters, absolute structure parameter using Flack's method = –0.03(2), residual electron density 1.4 (1.71 Å from O8)/ –1.9 (0.82 Å from Rh2) e · Å^{-3}. **CCDC- 1990589**.

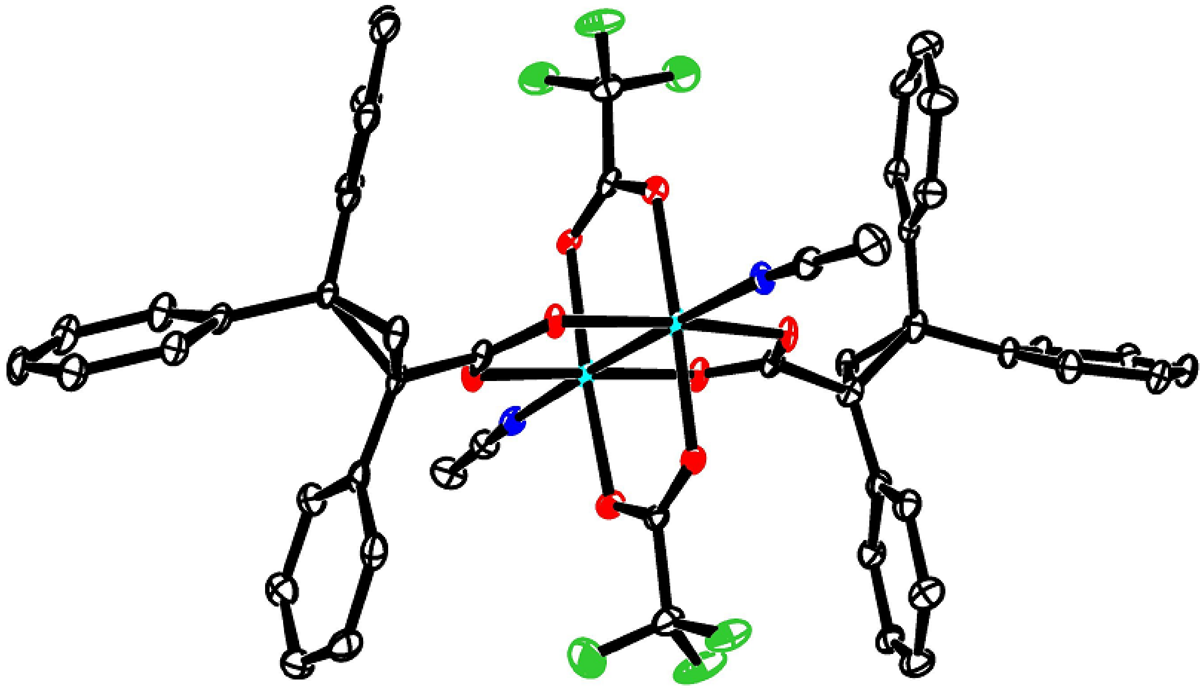

Figure S-6. Structure of *trans*-[$Rh_2(TFA)_2((R)\text{-}TPCP)_2$]·2MeCN in the solid state; hydrogen atoms are omitted for clarity.

X-ray Crystal Structure Analysis of *trans*-[$Rh_2(TFA)_2((R)\text{-}TPCP)_2$]·2MeCN: C_{52} H_{34} D_6 F_6 N_2 O_8 Rh_2, M_r = 1146.71 g mol^{-1}, violet plate, crystal size 0.08 x 0.07 x 0.05 mm^3, monoclinic, space group $P2_1$ [No. 4], a = 12.1320(14) Å, b = 12.5031(3) Å, c = 16.2999(12) Å, β = 106.801(7)°, V = 2367.0(3) Å^3, T = 100(2) K, Z = 2, D_{calc} = 1.609 g cm^{-3}, λ = 0.71073 Å, $\mu(Mo\text{-}K_\alpha)$ = 0.778 mm^{-1}, analytical absorption correction ($T_{\min}$ = 0.97, $T_{\max}$ = 0.98), Bruker-AXS Kappa Mach3 diffractometer with APEX-II detector and IμS micro focus, 3.147 < θ < 27.499°, 34197 measured reflections, 10668 independent reflections, 9835 reflections with $I > 2\sigma(I)$, R_{int} = 0.0570. The structure was solved by dual space methods (*SHELXT*) and refined by full-matrix least-squares (*SHELXL*) against F^2 to R_1 = 0.040 [$I > 2\sigma(I)$], wR_2 = 0.097, 633 parameters, absolute structure parameter using Flack's method = 0.005(19), residual electron density 0.8 (0.88 Å from Rh2)/ –1.2 (0.85 Å from Rh2) e · Å^{-3}. **CCDC- 2003930**.

SCREENING RESULTS

Table S-1. Screening of standard catalysts with **1a**

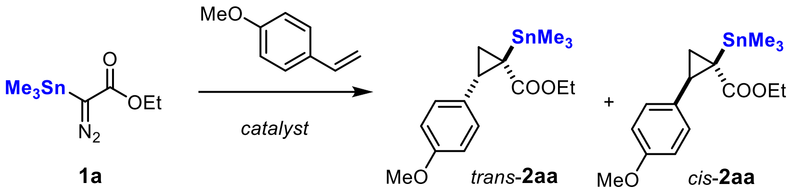

Entry	Catalyst	Solvent	T (°C)	*trans:cis*	ee (*trans*) [%]	ee (*cis*) [%]	Yield (NMR %)
1	[Rh_2((*S*)-PTTL)$_4$]	pentane	RT	85:15	49	n.d.	quant.
2	Rh_2((*S*)-NTTL)$_4$]	pentane	RT	8:20	37	0	45
3	[BiRh((*S*)-NTTL)$_4$]	pentane	RT	---	---	---	NR
4	[Rh_2((*S*)-PTAD)$_4$]	pentane	RT	85:15	35	9	quant.
5	[Rh_2((*S*)-BTPCP)$_4$]	pentane	RT	45:55	–50	–58	69
6	[Rh_2((*S*)-BTPCP)$_4$]	CH_2Cl_2	RT	51:49	–64	–50	11
7	[Rh_2((*S*)-p-PhTPCP)$_4$]	CH_2Cl_2	RT	47:54	–51	–38	traces
**8**	**[Rh_2((*R*)-TPCP)$_4$][a]**	**CH_2Cl_2**	**RT**	**50:50**	***up to* 92**	***up to* 96**	***up to* quant.**

[a] the results proved highly variable, depending on the batch of catalyst, see Text; NR = no reaction; n. d. = not determined

Table S-2. Screening of standard catalysts with the more bulky ethyl α-diazo-α-tributylstannylacetate

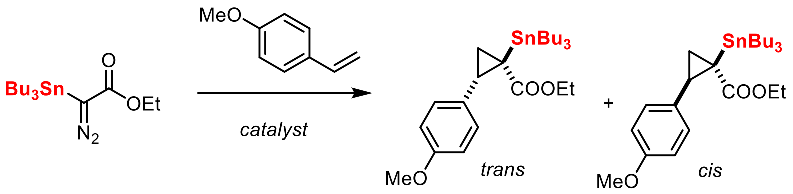

Entry	Catalyst	Solvent	T (°C)	*trans:cis*	ee (*trans*) [%]	ee (*cis*) [%]	Yield (NMR %)
1	[Cu(box)]BF_4	toluene	RT → 80	39:61	–7	17	< 5
2	[Rh_2((*S*)-PTTL)$_4$]	toluene	RT	86:14	28	37	17
3	[Rh_2((*S*)-PTTL)$_4$]	pentane	RT	91:9	37	43	63
4	[Rh_2((*S*)-PTTL)$_4$]	pentane	0	92:8	25	57	58
5	[BiRh((*S*)-PTTL)$_4$]	pentane	RT	---	---	---	NR
6	[Rh_2((*S*)-NTTL)$_4$]	pentane	RT	n. d.	n. d.	n. d.	< 5
7	[Rh_2((*S*)-PTAD)$_4$]	pentane	RT	---	---	---	NR
8	[Rh_2((*R*)-MEPY)$_4$]	pentane	RT → 80	---	---	---	NR
9	[Rh_2((*R*)-DOSP)$_4$]	pentane	RT → 80	68:32	7	–6	< 5
10	**[Rh_2((*R*)-TPCP)$_4$][a]**	**toluene**	**RT → 80**	**58:42**	***up to* 85**	***up to* –72**	**< 5**

[a] the results proved highly variable, depending on the batch of catalyst, see Text; NR = no reaction; n. d. = not determined

Structures of the screened precatalysts (note that complexes with different absolute configuration may have been used):

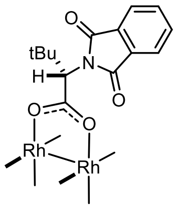

[Rh_2(*S*-PTTL)$_4$]

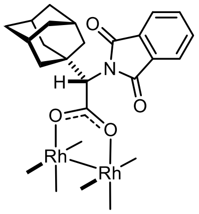

[Rh_2(*S*-PTAD)$_4$]

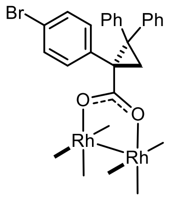

[Rh_2(*R*-BTPCP)$_4$]

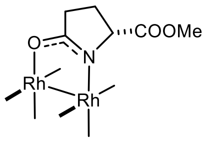

[Rh_2(5*R*-MEPY)$_4$]

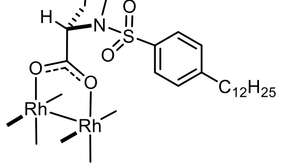

[Rh_2(*S*-DOSP)$_4$]

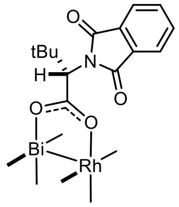

[BiRh(*S*-PTTL)$_4$]

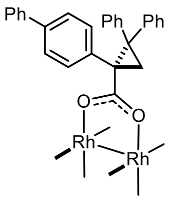

[Rh_2(*R*)-p-PhTPCP)$_4$]

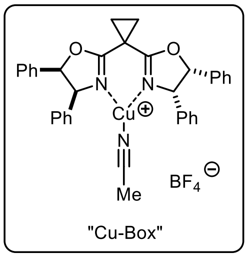

"Cu-Box"

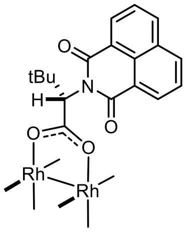

[Rh_2(*S*-NTTL)$_4$]

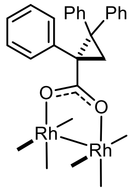

[Rh_2(*R*)-TPCP)$_4$]

General

Unless stated otherwise, all reactions were carried out under argon atmosphere in flame dried Schlenk glassware, ensuring inert conditions. The solvents were purified by distillation over the indicated drying agents under argon: THF, Et_2O (Mg/anthracene); pentane, toluene (Na/K); CH_2Cl_2, chlorobenzene (CaH_2); MeCN was dried by an absorption solvent purification system based on molecular sieves. Flash chromatography: Merck Geduran silica gel 60 (40 – 63 µm).

NMR spectra were recorded on Bruker DPX 300, AV 400, AV 500 or AV III 600 spectrometers in the solvents indicated; chemical shifts are given in ppm relative to TMS, coupling constants (*J*) in Hz. The solvent signals were used as references and the chemical shifts converted to the TMS scale ($CDCl_3$: δ_C = 77.2 ppm; residual $CHCl_3$: δ_H = 7.26 ppm; CD_2Cl_2: δ_C = 54.0 ppm; residual $CHDCl_2$: δ_H = 5.32 ppm; C_6D_6: δ_C = 128.1 ppm; residual C_6HD_5: δ_H = 7.16 ppm; CH_3CN: δ_C = 1.3, 118.3 ppm; residual CH_2DCN: δ_H = 1.94 ppm). Signal assignments were established using HSQC, HMBC and NOESY experiments.

IR: Alpha Platinum ATR (Bruker), wavenumbers ($\tilde{\nu}$) in cm^{-1} Most medium and weak resonances were omitted.

MS (EI): Finnigan MAT 8200 (70 eV), ESI-MS: ESQ 3000 (Bruker) or Thermo Scientific LTQ-FT or Thermo Scientific Exactive Spectrometer. HRMS: Bruker APEX III FT-MS (7 T magnet), MAT 95 (Finnigan), Thermo Scientific LTQ-FT or Thermo Scientific Exactive Spectrometer. GC-MS spectra were measured on a Shimadzu GCMS-QP2010 Ultra instrument.

LC analyses were conducted on a Shimadzu LC 2020 instrument equipped with a Shimadzu SPD-M20A UV/VIS detector. GC analyses were conducted on an Agilent technologies 7890B instrument with a FID detector.

Unless stated otherwise, all commercially available compounds (abcr, Acros, Aldrich, Alfa Aesar, Fluoro Chem, Strem, TCI) were used as received.

Synthesis of α-Metallated Diazoester Derivatives

Ethyl (trimethylstannyl)diazoacetate (1a): A flame dried 50 mL Schlenk flask was charged with commercial N,N,1,1,1-pentamethylstannanamine (1.55 mL, 9.50 mmol) and diethyl ether (20 mL). A solution of ethyl diazoacetate in CH_2Cl_2 (6.4 mol/L, 1.5 mL, 9.60 mmol) was added dropwise via syringe over the course of 1 min at ambient temperature. The resulting orange mixture was stirred for 6 h before all volatile materials were removed in vacuo. The resulting orange greasy solid was purified by sublimation at 60°C and $1{\cdot}10^{-3}$ mbar (*for safety reasons the sublimation was performed behind an additional protective shield even though no problems were encountered in different runs*). CH_2CL_2 (5 mL) to give the title compound as a crystalline orange solid (2.60 g, 99 % yield).

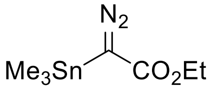

^{1}H-NMR (400 MHz, CD_2Cl_2): δ = 4.11 (q, J = 7.1 Hz, 2H), 1.22 (t, J =7.1 Hz, 3H), 0.37 (s, J_{Sn-H} = 56.4 Hz, 59.0 Hz, 9H) ppm; ^{13}C{^{1}H}-NMR (100 MHz CD_2Cl_2): δ = 61.1, 14.8, −7.7 ppm (C-N_2 not detected, C-O detected in ^{1}H-^{13}C-HMBC at 170.8 ppm);^{119}Sn{^{1}H}-NMR (149 MHz, CD_2Cl_2) = 25.5 ppm; IR (ATR): $\tilde{\nu}$ = 2982, 2109, 2067, 1672, 1464, 1376, 1350, 1275, 1172, 1095, 1046, 772, 738, 534, 508, 468 cm^{-1}; HRMS (EI): *m/z* calcd. for $C_7H_{14}N_2O_2Sn$ [M]: 278.0077; found: 278.0074. The analytical data is consistent with the literature.[1]

The compound rapidly decomposes when exposed to air.

Ethyl (trimethylgermyl)diazoacetate (1b): A flame dried 25 mL Schlenk flask was charged with a solution of dimethylamine (1.2 mol·L^{-1} in THF, 4 mL, 4.80 mmol) and diethyl ether (10 mL). *n*-BuLi (1.6 mol·L^{-1} in hexane, 3 mL, 4.16 mmol) was added slowly at −78°C and the resulting colorless suspension was allowed to warm to ambient temperature. After stirring for 30 min, the mixture was concentrated to dryness under reduced pressure to give a white solid. A solution of Me_3GeCl (496 mg, 3.24 mmol) in diethyl ether (5 mL) was added at 0°C and the resulting mixture was stirred at 50°C (bath temperature) for 1.5 h. The resulting light yellow slurry was transferred into a 25 mL two neck round bottom flask through a cannula-filter and then distilled at ≈ 110°C into a 10 mL Schlenk flask which was cooled in a dry ice/acetone bath to give N,N,1,1,1-pentamethylgermanamine as a colorless oil, which was directly used for the next step.

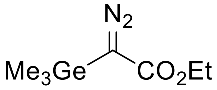

A solution of ethyl diazoacetate in CH_2Cl_2 (6.4 mol·L^{-1}, 0.3 mL, 1.92 mmol) was added via syringe over the course of 1 min to N,N,1,1,1-pentamethylgermanamine (220 mg, 1.13 mmol). The resulting orange

mixture was stirred at 80°C for 2 h in the Schlenk flask, which was connected to a cooling trap at −78°C to capture all volatile materials formed. The resulting orange liquid was purified by distillation at $5{\cdot}10^{-3}$ mbar, collecting the fraction that distilled at ≈70°C to yield ethyl (trimethylgermyl)diazoacetate as a yellow liquid (72 mg, 0.31 mmol, 28 %). ^{1}H-NMR (400 MHz, CD_2Cl_2): δ = 4.14 (q, *J* = 7.1 Hz, 2H), 1.24 (t, *J* = 7.1 Hz, 3H), 0.43 (s, 9H) ppm; ^{13}C{^{1}H}-NMR (100 MHz, CD_2Cl_2): δ = 61.1, 14.8, −1.2. (C-N_2 and C-O detected in ^{1}H-^{13}C-HMBC at 44.2 ppm and 170.5 ppm respectively); IR (ATR): $\tilde{\nu}$ = 2980, 1911, 2075, 1680, 1447, 1399, 1366, 1274, 1203, 1180, 1061, 828, 768, 737, 610, 574, 554, 475 cm^{-1}; HRMS (ESI+): *m/z* calcd. for $C_7H_{14}GeN_2O_2Na$ [M+Na]: 255.0159; found: 255.0160. The analytical data is consistent with the literature.[2]

The compound can be handled for short periods of time in air but was stored under Ar in a refrigerator.

Ethyl (trimethylsilyl)diazoacetate (1c): A flame dried 50 mL Schlenk flask was charged with a solution of

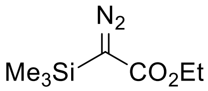

ethyl diazoacetate in CH_2Cl_2 (87 %, 0.5 mL, 4.14 mmol), N-ethyldiisopropylamine (0.9 mL, 4.96 mmol) and diethyl ether (20 mL). The mixture was cooed to −78°C before a solution of TMSOTf (0.8 mL, 4.14 mmol) in diethyl ether (5 mL) was added dropwise at this temperature. The resulting yellow mixture was allowed to warm to ambient temperature and stirring was continued for 20 h, during which time a colorless solid precipitated. The mixture was cannula filtrated into a flame dried 50 mL two neck flask and all volatile materials were evaporated. The resulting oily, orange residue was purified by distillation at $1{\cdot}10^{-3}$ mbar; the fraction that distilled at 27-32°C was collected to give the title compound as an orange oil (562 mg, 73 %). ^{1}H-NMR (400 MHz, $CDCl_3$): δ = 4.19 (q, *J* = 7.0 Hz, 2H), 1.26 (t, *J* = 7.0 Hz, 3H), 0.25 (s, 9H) ppm; ^{13}C{^{1}H}-NMR (100 MHz, $CDCl_3$): δ = 60.8, 14.6, −13 ppm (C-N2 and C-O detected in ^{1}H-^{13}C-HMBC at 44.9 ppm and 171.1 ppm respectively); IR (ATR): $\tilde{\nu}$ = 2096, 1683, 1366, 1211, 1185, 1074, 764, 698, 629, 485, 410 cm^{-1}; HRMS (ESI+): *m/z* calcd. for $C_7H_{14}N_2O_2SiNa$ [M+Na]: 209.0717; found: 209.0718. The analytical data is consistent with the literature.[3]

The compound can be handled for short periods of time in air but was stored under Ar in a refrigerator.

Ligands and Dirhodium Complexes

Methyl *(R)*-1,2,2-triphenylcyclopropane-1-carboxylate (S-1): A solution of methyl

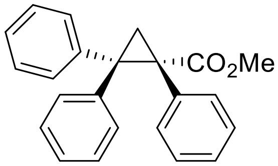

2-diazo-2-(4-methoxy)phenylacetate (2.00 g, 11.35 mmol) in pentane (10 mL) was added with syringe pump over a course of 3 h to a mixture of diphenylethylene (3 mL, 17.03 mmol) and Rh_2(*(R)*-DOSP)$_4$ (100 mg, 0.05 mmol) in pentane (30 mL) at −78°C. The mixture was allowed to warm to room temperature and stirring was continued for 12 h. The solvent was evaporated and the residue purified by flash chromatography (silica, hexanes/ethyl acetate, 99:1) to give the title compound as a colorless solid (2.57 g, 69 %, 98 % *ee*). Recrystallization from refluxing hexanes/ethyl acetate (1:99) gave the title compound as a crystalline solid with >99 % *ee* (2.05 g, 55 %). ^{1}H-NMR (400 MHz, $CDCl_3$): δ = 7.51 (m, 2H), 7.33 (m, 4H), 7.24 (m, 1H), 7.17-7.08 (m, 3H), 6.99-6.91 (m, 5H), 3.36 (s, 3H), 2.69 (d, *J* = 5.6 Hz, 1H), 2.42 (d, *J* = 5.6 Hz, 1H) ppm. The analytical data is consistent with the literature.[4]

The racemic ligand was prepared analogously but with $Rh_2(esp)_2$[5] instead of Rh_2(*(R)*-DOSP)$_4$.

The optical purity was determined by HPLC (250 mm Chiralpak IA, 3µm, 4.6 mm, 10 % 2-propanol in *n*-heptane, 1 mL·min^{-1}, 10 min, UV 220 nm): 5.45 min (major) and 6.63 min (minor) (Figure S-7)

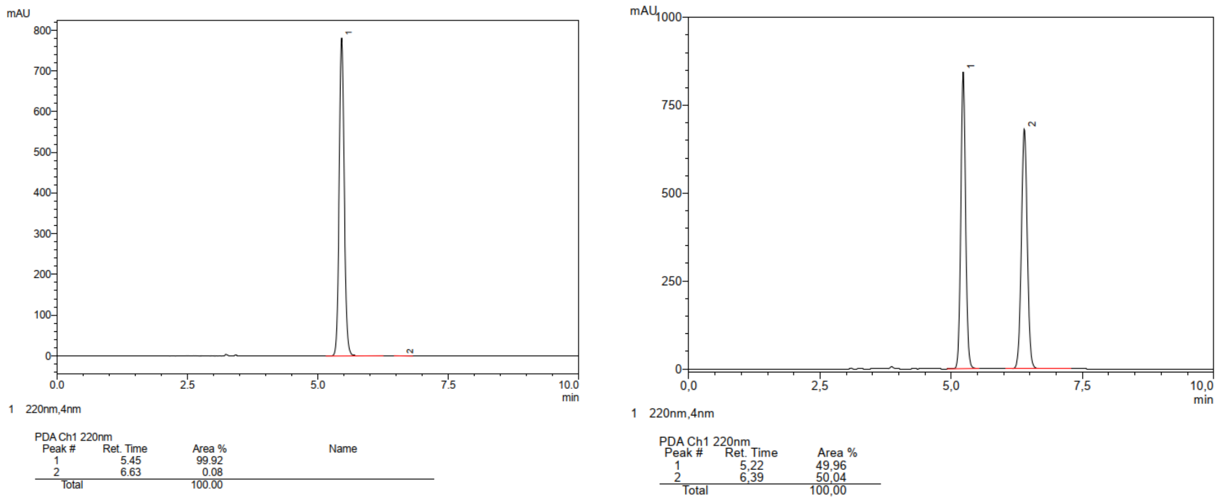

Figure S-7. HPLC traces of methyl *(R)*-1,2,2-triphenylcyclopropane-1-carboxylate (left) and the corresponding racemate (right).

***(R)*-1,2,2-Triphenylcyclopropane-1-carboxylic acid (4):** TMSOK (3.070 g, 21.56 mmol) was added to a solution of ester **S-1** (708 mg, 2,16 mmol) in THF (10 mL) and the resulting mixture was stirred at room temperature for 12 h. The solution was cooled to 0°C before a solution of citric acid in water (3.0 mol·L^{-1}, 1.0 mL) was added. After stirring for 30 min, the mixture was extracted with ethyl acetate (3 x 20 mL). The combined organic extracts were washed with brine (20 mL), dried over Na_2SO_4 and concentrated under reduced pressure. The residue was purified by flash chromatography (silica, hexanes/ethyl acetate, 4:1) to give the title compound as a colorless solid. (543 mg, 80 %, >99 % *ee*). $[\alpha]_D^{20}$ = −306.3° (c = 1.0 g·100 mL^{-1}, $CHCl_3$) [Lit.[4]: $[\alpha]_D^{20}$ = −223.5° (c = 1.0 g·100 mL^{-1}, $CHCl_3$)]; ^{1}H-NMR (400 MHz, CD_2Cl_2): δ = 7.51 (m, 2H), 7.35-7.28 (m, 4H), 7.14 -6.85(m, 9H), 2.54 (d, *J* = 5.6 Hz, 1H), 2.48 (d, *J* = 5.6 Hz, 1H) ppm; ^{13}C-NMR (400 MHz, CD_2Cl_2): δ = 174.4, 142.1, 139.7, 135.4, 131.9, 129.7, 128.9, 128.4, 127.7, 127.4, 127.0, 126.9, 126.3, 45.9, 42.1, 25.6 ppm; HRMS (ESI–): *m/z* calcd. for $C_{22}H_{17}O_2$ [M-H]: 313.1234; found: 313.1236. The analytical data is consistent with the literature.[4]

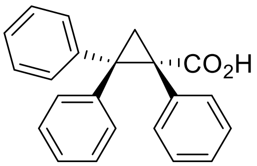

The enantiomeric excess (*ee*) was determined by converting a sample of the acid (10 mg, 0.03 mmol) back into the methyl ester by stirring it in DMF (1 mL) in the presence of methyl iodide (4 µL, 0.06 mmol) and potassium carbonate (8.8 mg, 0.06 mmol) for 12 h followed by extraction into CH_2Cl_2 (2 mL). HPLC of the corresponding methyl ester **S-1** (250 mm Chiralpak IA, 3µm, 4.6 mm, 10 % 2-propanol in n-heptane, 1 mL·min^{-1}, 10 min, UV 220 nm): 5.45 min (major) and 6.63 min (minor) (Figure S-8).

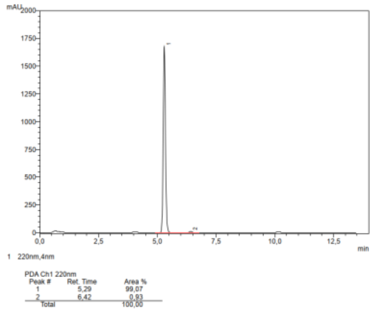

Figure S-8. HPLC trace of methyl *(R)*-1,2,2-triphenylcyclopropane-1-carboxylate, obtained by methylation of acid **4**.

[Rh_2(*(R)*-TPCP)$_4$] (5). Method A. A 25 mL two-neck flask was equipped with Soxhlet extractor (returning-arm frit) which in turn was topped by a reflux condenser. The Soxhlet extractor was filled with an oven-dried mixture of K_2CO_3 and sand (1:1, ca. 3 g).

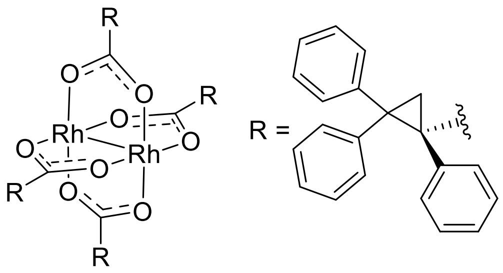

The flask was charged with dirhodium(II) acetate dihydrate (90 mg, 0.19 mmol), *(R)*-**4** (470 mg, 1.50 mmol), and toluene (20 mL). The mixture was stirred at reflux temperature and the refluxing solvent passed through the Soxhlet extractor. After 12 h, the mixture was cooled to room temperature and concentrated under reduced pressure. The green residue was purified by flash chromatography (silica, acetonitrile/toluene, 1:9) to give the title compound as a green solid (205 mg, 0.14 mmol, 75 %).

Method B. Dirhodium(II) acetate dihydrate (500 mg, 1.13 mmol) was suspended in an aqueous solution of sodium carbonate (2 mol·L^{-1}, 20 mL) and the resulting mixture was stirred at reflux temperature for 30 min, during which time the color changed from green to purple. The precipitate was filtered off and washed with water, methanol and *tert*-butyl methyl ether to yield tetrasodium-dirhodium(II)carbonate x 2.5 hydrate as a purple solid, which was directly used for the next step (495 mg, 81 %). IR (ATR): $\tilde{\nu}$ = 3311, 1689, 1478, 1413, 1074, 836, 761, 726 cm^{-1}

A mixture of tetrasodium-dirhodium(II)carbonate x 2.5 hydrate (100 mg, 0.19 mmol) and *(R)*-**4** (500 mg, 1.59 mmol) in water (20 mL) was stirred at reflux temperature for 4 h, during which time the mixture turned from purple to green. Saturated sodium bicarbonate solution (20 mL) was then added and the mixture was extracted with CH_2Cl_2 (3 x 20 mL). The organic phase was washed with saturated sodium bicarbonate solution (2 x 20 mL) until the aqueous phase was clear. After the organic phase had been dried with Na_2SO_4, the solvent was removed *in vacuo* to yield the title complex as a green solid which was purified by flash chromatography (silica, acetonitrile/toluene, 1:9) (96 mg, 35 %); this material appeared to be pure by NMR and was used for the first screenings but actually contained several trace impurities (cf. Figure 1 of the main text). Analytically pure samples of complex **5** (>99%) and three trace impurities were obtained by HPLC (Zorbax 300 SB-C8, 21 x 150 mm, 5 µm, 15 mL·min^{-1},acetonitrile / water 85:15).

Single crystals were obtained by slow evaporation of a concentrated acetonitrile solution at ambient temperature over 7 d.

$[\alpha]_{D}^{20}$ = −23.3° (c = 0.03 g·100 mL^{-1}, $CHCl_3$) [Lit.[4]: $[\alpha]_{D}^{20}$ = –12.8° (c = 1.0 g·100 mL^{-1}, $CHCl_3$)]. ^{1}H-NMR (400 MHz, $CDCl_3$): 7.39 – 6.60 (m, 60H), 2.40 (d, *J* = 5.2 Hz, 2H), 1.92 (d, *J* = 5.2 Hz, 2H) ppm; ^{13}C-NMR (400 MHz, $CDCl_3$): 189.1, 142.5, 141.1, 136.4, 131.1, 129.9, 129.2, 128.0, 127.4, 127.3, 127.2, 127.2, 126.3, 125.7, 46.4, 43.2, 23.4 ppm; The analytical data is consistent with the literature.[4]

[Rh_2(acam)((*R*)-TPCP)$_3$] (7). Method A. A 25 mL two-neck flask was equipped with Soxhlet extractor (returning-arm frit) which in turn was topped by a reflux condenser. The Soxhlet extractor was filled with an oven-dried mixture of K_2CO_3 and sand (1:1, ca. 3 g).

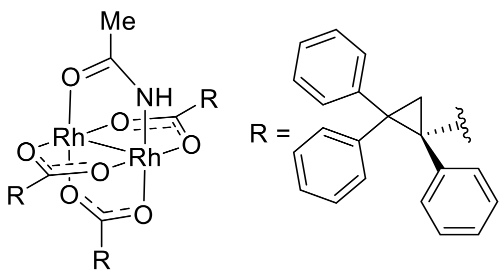

The flask was charged with [Rh_2(TFA)((*R*)-TPCP)$_3$] (**10**) (200 mg, 0.16 mmol), acetamide (10.0 mg, 0.17 mmol), and chlorobenzene (15 mL). The mixture was stirred at reflux temperature and the refluxing solvent passed through the Soxhlet extractor. Reaction progress was checked by HPLC chromatography (Eclipse Plus C-8, 1.8 μm, 3.0 x 50 mm, acetonitrile /water gradient 60:40 – 90:10 in 10 min 0.5 mL·min^{-1}, 308 K, 20 min, 230 nm; retention time of product: 7.14 min). After 12 h, the mixture was cooled to room temperature and concentrated under reduced pressure. The green residue was purified by flash chromatography (silica, acetonitrile/toluene, 1:4) to give the title compound as a green solid (72 mg). Remaining traces of [Rh_2(acam)$_2$((*R*)-TPCP)$_2$] were removed by HPLC (YMC-C18 Triart, 5.0 μm, 30 x 150 mm, acetonitrile/water, 80:20, 35 mL·min^{-1}, 308 K, 20 min, retention time of impurity 7.1 min, retention time of product: 13.6 min; analytical HPLC conditions: Zorbax Eclipse , 1.8 μm, 4.6 x 50 mm, acetonitrile /water, 85:15, 0.8 mL·min^{-1}, 308 K, 8 min, retention time of product: 3.22 min) (Figure S-9). The compound-containing fractions were combined and evaporated, and the residue was dried under vacuum to give the title complex as a green solid (60.0 mg, 31 %). Single crystals for X-ray crystal structure analysis were obtained by layer diffusion of pentane into a solution of the complex in DMF/benzene (1:20) at room temperature over 48 h.

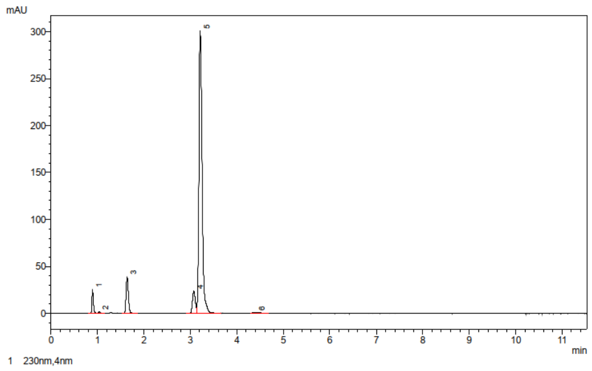

PDA Ch1 230nm

Peak #	Ret. Time	Area %	Name
1	0.90	3.04	
2	1.04	0.24	
3	1.64	7.43	
4	3.07	5.66	
5	3.21	82.94	
6	4.40	0.69	
Total		100.00	

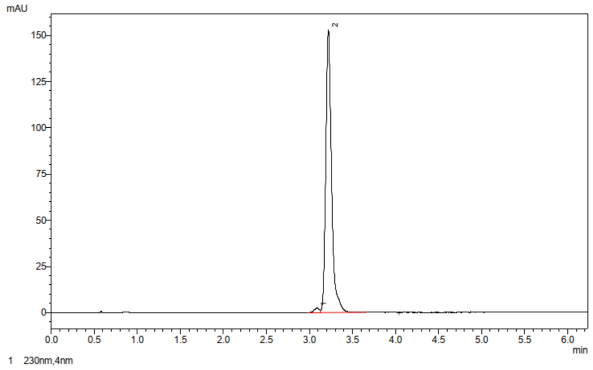

PDA Ch1 230nm

Peak #	Ret. Time	Area %	Name
1	3.09	1.36	
2	3.22	98.64	
Total		100.00	

Figure S-9. HPLC traces of complex **7** prepared according to method A before (left) and after HPLC purification (right).

Method B. A solution of tetrabutylammonium hydroxide (1.0 mol·L^{-1}in MeOH), 90 μL, 0.09 mmol) was added to a solution of [Rh_2(TFA)(*(R)*-TPCP)$_3$] (**10**) (110 mg, 0.09 mmol) in acetonitrile (5 mL). The mixture was stirred at ambient temperature for 12 h, before it was concentrated under reduced pressure. The resdiue was purified by flash chromatography (silica, acetonitrile/toluene, 1:4) and the product dried under high vacuum to give the title complex as a green solid (23 mg, 22 %).

$[\alpha]_D^{20}$ = −218.6° (c = 0.07 g·100 mL^{-1}, $CHCl_3$). ^{1}H-NMR (400 MHz, CD_3CN): δ = 7.61 – 6.61 (m, 45H), 3.88 (s, 1H), 2.35 (d, *J* = 5.4 Hz, 1H), 2.31 (d, *J* = 5.2 Hz, 1H), 2.27 (d, *J* = 5.1 Hz, 1H), 2.04 (d, *J* = 5.2 Hz, 1H), 1.97 (d, *J* = 5.4 Hz, 1H), 1.58 – 1.51 (d, *J* = 5.1 Hz 1H), 1.47 (s, 3H) ppm; ^{13}C{^{1}H}-NMR (100 MHz CD_3CN): δ = 188.8, 188.7, 187.6, 186.1, 144.9, 144.3, 144.2, 143.2, 143.0, 142.9, 138.9, 138.8, 138.6, 132.6, 132.5, 131.8, 131.0, 130.8, 130.6, 130.5, 130.5, 130.4, 128.9, 128.8, 128.7, 128.5, 128.4, 128.2, 128.1, 127.9, 127.5, 127.2, 127.1, 127.1, 126.9, 126.7, 126.6, 126.5, 126.4, 126.3, 47.0, 45.8, 45.0, 44.7, 44.6, 43.1, 25.0, 24.1, 23.1, 22.1 ppm.; IR (solid, ATR) $\tilde{\nu}$ = 3056, 3025, 1592, 1494, 1448, 1383, 1264, 1202, 1077, 1031, 1005, 988, 734, 694, 669, 617, 603, 584, 547, 484 cm^{-1}; HRMS (ESI+): *m/z* calcd. for $C_{66}H_{55}NO_7Rh_2$ [M^+]: 1203.2083; found: 1217.2089.

(Rh_2(OAc)(*(R)*-TPCP)$_3$) (8). A 25 mL two-neck flask was equipped with Soxhlet extractor (returning-arm frit) which in turn was topped by a reflux condenser. The Soxhlet extractor was filled with an oven-dried mixture of K_2CO_3 and sand (1:1, ca.3 g).

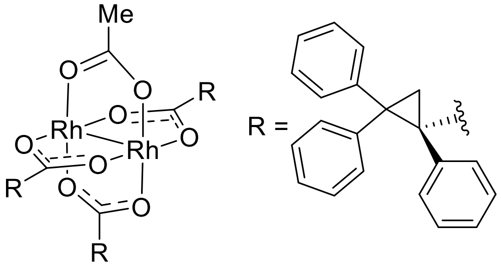

The flask was charged with $Rh_2(OAc)_4·2H_2O$ (99 mg, 0.21 mmol), *(R)*-**4** (193 mg, 0.62 mmol) and toluene (20 mL). The green mixture was stirred at reflux temperature and the refluxing solvent passed through the Soxhlet extractor. After 12 h, the reaction mixture was cooled to room temperature and concentrated under reduced pressure. The green residue was purified by flash chromatography (silica, acetonitrile/toluene, 5:95 – 3:7) and the resulding product dried under high vacuum to give the title complex as a green solid material (191 mg, 77 %). $[\alpha]_D^{20}$ = +252.0° (c = 0.2 g·100 mL^{-1}, $CHCl_3$); ^{1}H-NMR (600 MHz, CD_3CN): δ = 7.44 – 6.59 (m, 45H), 2.37 (d, *J* = 5.4 Hz, 2H), 2.27 – 2.23 (m, 1H), 2.04 (d, *J* = 5.4 Hz, 2H), 1.38 (m, 4H) ppm; ^{13}C{^{1}H}-NMR (100 MHz CD_3CN): δ = 190.7, 190.2, 190.0, 144.6, 144.0, 142.9, 142.7, 138.9, 138.4, 138.1, 132.4, 130.7, 130.5, 130.4, 130.4, 129.9, 129.2, 129.0, 128.8, 128.5, 128.3, 128.1, 127.6, 127.3, 127.1, 126.8, 126.5, 126.5, 126.2, 47.5, 45.5, 44.6, 43.0, 24.3, 24.0, 22.5, 21.4 ppm; IR (solid, ATR) $\tilde{\nu}$ = 1657, 1439, 1189, 1153, 859, 786, 736, 714, 529 cm^{-1}; HRMS (ESI+): *m/z* calcd. for $C_{68}H_{54}O_8Rh_2Na$ [M+Na]: 1227.1821; found: 1227.1819.

[Rh_2(TFA)(*(R)*-TPCP)$_3$] (10). A 50 mL two-neck flask was equipped with Soxhlet extractor (returning-arm frit) which in turn was topped by a reflux condenser. The Soxhlet extractor was filled with an oven-dried mixture of K_2CO_3 and sand (1:1, ca.3 g).

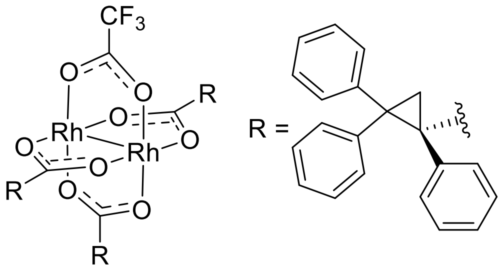

The flask was charged with [$Rh_2(TFA)_4$] dihydrate (250 mg, 0.36 mmol), *(R)*-**4** (339 mg, 1.08 mmol) and *tert*-butyl acetate (15 mL). The green-blue mixture was degassed by bubbling

argon through it for 15 min. The stirred mixture was then heated in an oil bath (100°C bath temperature) so that the refluxing solvent passes through the Soxhlet extractor. Reaction progress was checked by HPLC chromatography (Eclipse Plus C-8, 1.8 μm, 3.0 x 50 mm, acetonitrile/water gradient 60:40 – 90:10 in 10 min, 0.5 mL·min^{-1}, 20 min, 230 nm; retention time of product: 8.79 min). After 44 h, ligand **4** was consumed and the mixture allowed to cool to room temperature before it was concentrated under reduced pressure. The green residue was purified by flash chromatography (silica, acetonitrile/toluene, 1:99) and the resulting product was dried under high vacuum to give the title compound as a green solid (303 mg, 66 %). $[\alpha]_D^{20}$ = –133.0° (c = 0.2 g·100 mL^{-1}, $CHCl_3$); ^{1}H-NMR (400 MHz, CD_3CN): δ = 7.50 – 6.71 (m, 45H), 2.40 (m, 3H), 1.87 (d, *J* = 5.3 Hz, 2H), 1.71 (d, *J* = 5.4 Hz, 1H) ppm; ^{13}C{^{1}H}-NMR (100 MHz, CD_3CN): δ = 192.21, 191.39, 173.1 (q $J_{13C\text{-}19F}$ = 38.2 Hz), 144.2, 144.1, 142.5, 138.9, 137.9, 137.7, 132.4, 130.7, 130.4, 129.9, 129.3, 129.1, 129.0, 128.6, 128.5, 128.4, 128.1, 127.9, 127.4, 127.2, 127.0, 126.8, 126.8, 126.3, 47.3, 46.9, 44.3, 43.6, 24.1, 23.7 ppm; ^{19}F-NMR (470 MHz, CD_3CN): δ = –74.5 ppm; IR (solid, ATR) $\tilde{\nu}$ = 3058, 3026, 1635, 1600, 1548, 1494, 1448, 1383, 1198, 1150, 1077, 1031, 988, 863, 782, 739, 693, 669, 618, 603, 54, 485 cm^{-1}; HRMS (ESI+): *m/z* calcd. for $C_{68}H_{51}F_3O_8Rh_2$ [M]: 1258.1641; found: 1258.2623.

A second fraction collected during flash chromatography contained a mixture of *cis*- and *trans*-[$Rh_2(TFA)_2$((*R*)-TPCP)$_2$] (green solid, 140 mg, 33%). Reaction of this mixture (550 mg, 0.52 mmol) with *(R)*-**4** (163 mg, 0.52 mmol) under the conditions described above also gave the title complex **10** (442 mg, 68 %).

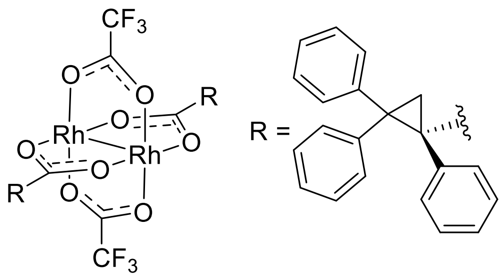

Single crystals of pure *trans*-[$Rh_2(TFA)_2$((*R*)-TPCP)$_2$]·2MeCN could be grown from a concentrated solution of the mixture in MeCN; for the structure of the complex in the solid state, see Figure S-6. ^{1}H-NMR (600 MHz, CD_3CN): δ = 7.32 – 7.21 (m, 4H), 7.20 – 6.98 (m, 20H), 6.95 – 6.90 (m, 4H), 6.88 – 6.82 (m, 2H), 2.37 (d, *J* = 5.4 Hz, 2H), 2.15 (d, *J* = 5.4 Hz, 2H), [2.14 (s, H_2O)] ppm; ^{13}C{^{1}H}-NMR (100 MHz, CD_3CN_3): 193.0, 174.9 (q, J = 39.0 Hz) , 143.8, 142.1, 137.4, 132.1, 130.3, 130.2, 129.0, 128.6, 128.1, 127.5, 127.3, 127.0, 47.0, 44.7, 24.0 ppm; ^{19}F{^{1}H}-NMR (282 MHz, CD_3CN): –75.0 ppm.

With this pure sample of known structure in hand, the HPLC peaks of identical mass corresponding to these two geometric isomers could be assigned. With this additional information, one can deduce from the data recorded during reaction monitoring that (i) *trans*-[$Rh_2(TFA)_2$((*R*)-TPCP)$_2$] is more readily formed than *cis*-[$Rh_2(TFA)_2$((*R*)-TPCP)$_2$], and (ii) the conversion of *trans*-[$Rh_2(TFA)_2$((*R*)-TPCP)$_2$] into **10** is also somewhat faster than that of *cis*-[$Rh_2(TFA)_2$((*R*)-TPCP)$_2$]; representative HPLC chromatograms are shown in Figure S-10. This differential reactivity might provide a handle for a further optimization of the preparation of heteroleptic dirhodium complexes.

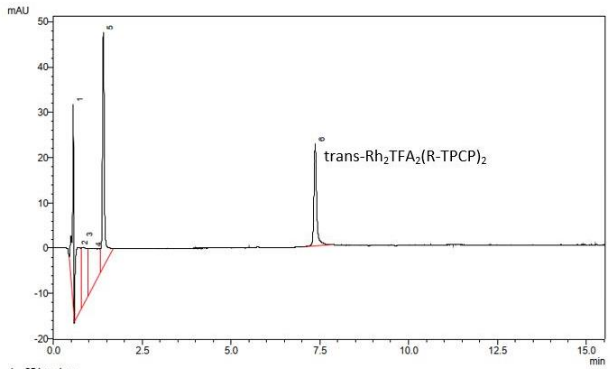

1 254nm,4nm

PDA Ch1 254nm

Peak #	Ret. Time	Area %	Name
1	0.56	13.57	
2	0.69	16.14	
3	0.83	14.65	
4	1.08	18.85	
5	1.40	24.05	
6	7.37	12.73	
Total		100.00	

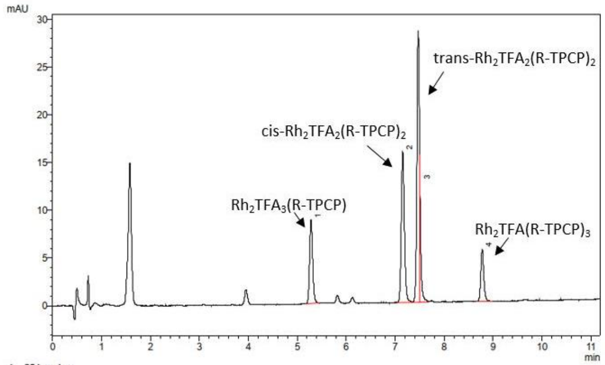

1 254nm,4nm

PDA Ch1 254nm

Peak #	Ret. Time	Area %	Name
1	5.28	14.67	
2	7.16	67.84	
3	7.51	8.40	
4	8.78	9.09	
Total		100.00	

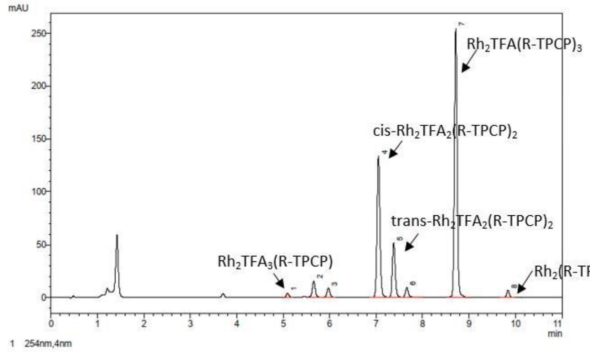

1 254nm,4nm

PDA Ch1 254nm

Peak #	Ret. Time	Area %	Name
1	5.09	0.88	
2	5.66	3.25	
3	5.97	1.87	
4	7.05	28.30	
5	7.38	10.87	
6	7.66	2.07	
7	8.71	51.40	
8	9.84	1.36	
Total		100.00	

Figure S-10. HPLC chromatograms: *trans*-[$Rh_2(TFA)_2((R)\text{-}TPCP)_2$] (top); reaction mixture after 20 h reaction time (middle); reaction mixture after 84 h reaction time (bottom)

Complex 11. A solution of $[Bu_4N][NHOCF_3]$ (9.5 mg, 0.03 mmol)[6] in acetonitrile was added to a stirred solution of $[Rh_2(TFA)((R)\text{-}TPCP)_3]$ (**10**) (33 mg, 0.03 mmol) in acetonitrile. The resulting red mixture was stirred at ambient temperature for 2 h, before it was concentrated under reduced pressure. The residue was purified by HPLC (YMC-Triart C8, 5 µm, 20x150 mm, acetonitrile / water, 75:25, 15 mL·min^{-1}, 308 K, retention time of product: 32.12 min – 33.66 min) to give the title compound as a green solid (3.3 mg, 10 %). ^{1}H-NMR (600 MHz, CD_3CN): δ = 7.66 – 6.46 (m, 45H), 2.37 (m, 2H), 2.32 (d, *J* = 5.2 Hz, 1H), 1.89 (d, *J* = 5.2 Hz, 1H), 1.81 (d, *J* = 5.4 Hz, 1H), 1.72 (d, *J* = 5.2 Hz, 1H) ppm; ^{13}C{^{1}H}-NMR (151 MHz CD_3CN): δ = 188.2, 188.2, 187.5, 168.8 (q, *J* = 34.6 Hz), 142.8, 142.6, 142.3, 141.1, 141.1, 141.0, 136.6, 136.4, 136.3, 130.7, 130.7, 130.1, 128.9, 128.9, 128.7, 128.6, 128.6, 128.5, 127.1, 126.9, 126.7, 126.5, 126.3, 126.1, 125.9, 125.4, 125.3, 125.3, 125.2, 125.1, 124.9, 124.9, 124.9, 124.8, 110.8 (q, *J* = 282.1 Hz), 45.2, 45.0, 44.2, 42.5, 42.3, 41.7, 22.2, 22.0, 20.9 ppm; HRMS (ESI+): *m/z* calcd. for $C_{68}H_{52}F_3NO_7Rh_2Na$ [M·+Na]: 1280.1698; found: 1280.1702.

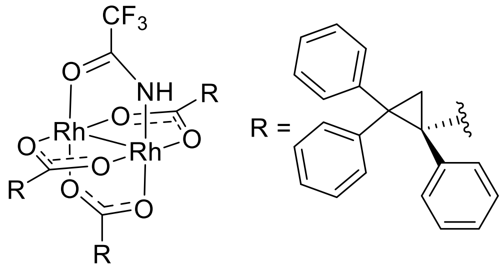

Complex 12. A 10 mL Schlenk flask was charged with N-methylacetamide (188 mg, 2.57 mmol) and complex **10** (20 mg, 0.02 mmol). The flask was immersed into an oil bath at 90°C, causing the formation of a purple melt. After 6 h, the mixture was cooled, the residue was dispersed in a mixture of acetonitrile and toluene (2:8, 10 mL) and the suspension was filtered through a plug of silica. The filtrate was evaporated and the remaining green-blue solid purified by HPLC (YMC Triart-C8, 5.0 µm, 20 x 150 mm, acetonitrile /water, 80:20, 15 mL·min^{-1}, 308 K, 20 min, retention time of product: 14.14-15.80 min) to give the title compound as a green solid (0.6 mg, 3 %). ^{1}H-NMR (600 MHz, CD_3CN): δ = 7.62 – 7.52 (m, 2H), 7.44 – 7.35 (m, 2H), 7.32 (t, *J* = 7.6 Hz, 2H), 7.28 – 7.21 (m, 8H), 7.20 – 7.15 (m, 3H), 7.15 – 7.01 (m, 10H), 6.99 – 6.90 (m, 9H), 6.89 – 6.72 (m, 7H), 6.62 – 6.54 (m, 2H), 2.44 (d, *J* = 5.4 Hz, 1H), 2.34 (s, 3H), 2.28 (d, *J* = 5.1 Hz, 1H), 2.20 (d, *J* = 5.2 Hz, 1H), 2.11 (d, *J* = 5.4 Hz, 1H), 2.10 – 2.05 (d, *J* = 5.2 Hz, 1H), 1.32 (d, *J* = 5.1 Hz, 1H), 1.28 (s, 3H) ppm; ^{13}C{^{1}H}-NMR (151 MHz CD_3CN): δ = 188.9, 188.4, 187.3, 181.6, 145.1, 144.2, 144.1, 143.4, 143.1, 142.6, 139.3, 138.8, 138.7, 132.6, 132.5, 132.2, 130.9, 130.9, 130.5, 130.4, 130.3, 129.1, 128.8, 128.7, 128.5, 128.5, 128.2, 127.9, 127.3, 127.3, 127.3, 127.2, 127.1, 126.7, 126.6, 126.4, 126.3, 126.1, 47.7, 45.2, 45.0, 44.5, 44.3, 42.6, 41.2, 24.3, 22.3, 21.4, 20.9 ppm; HRMS (ESI+): *m/z* calcd. for $C_{69}H_{57}NO_7Rh_2$ [M$^+$]: 1217.2240; found: 1217.2241.

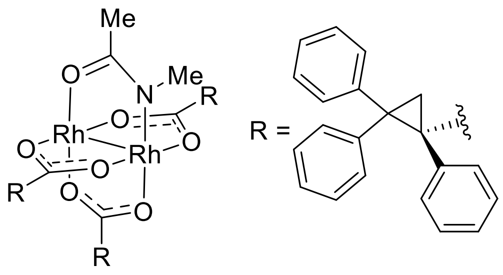

Rhodium Catalyzed Cyclopropanation Reactions

Representative procedure: Ethyl 2-(4-methoxyphenyl)-1-(trimethylstannyl)cyclopropane-1-carboxylate (2aa). A solution of ethyl (trimethylstannyl)diazoacetate **1a** (260 mg, 0.94 mmol,) in CH_2Cl_2 (4 mL) was added over 6 h from a gas-tight Hamilton syringe via syringe pump to a mixture of *p*-methoxystyrene (0.6 mL, 4.66 mmol) and complex **7** (12 mg, 0.01 mmol) in CH_2Cl_2 (5 mL) at ambient temperature. Once the addition was complete, the green mixture was stirred for additional 6 h, before it was concentrated under reduced pressure. The green residue was purified by flash chromatography (silica, hexanes/ethyl acetate, 20:1) to give the two diastereomers (dr = 1:1, NMR) of the title compound (275 mg, 72 %, combined yield). The relative stereochemistry of the diastereomers was assigned via ^1H-^1H-NOESY-NMR.

All racemates, as necessary for determination of the optical purity, were prepared analogously using [$Rh_2(esp)_2$] as catalyst.[5]

***cis*-2aa:** Colorless oil (115 mg, 37 %, 97 % *ee*). $[\alpha]_D^{20}$ = +45.5° (c = 0.38 g·100 mL^{-1}, $CHCl_3$). ^1H-NMR (600 MHz, $CDCl_3$): δ = 7.14 (d, *J* = 8.7 Hz, 2H), 6.80 (d, *J* = 8.7 Hz, 2H) 4.14(m, 2H), 3.79 (s, 3H), 2.68 (dd, *J* = 9.0 Hz, 6.4 Hz, 1H), 1.71 (dd, *J* = 8.8 Hz, 4.0 Hz, 1H), 1.28 (m, 4H), −0.17 (s, 9H) ppm; $^{13}C\{^1H\}$-NMR (100 MHz, $CDCl_3$): δ = 177.4, 158.6, 132.1, 130.7, 113.7, 61.0, 55.4, 30.3, 21.2, 16.7, 14.4, −8.4 ppm; $^{119}Sn\{^1H\}$-NMR (149 MHz, $CDCl_3$): 3.8 ppm; IR (film, ATR) $\tilde{\nu}$ = 2980, 2836, 1706, 1611, 1513, 1441, 1369, 1300, 1224, 1170, 1110, 1031, 968, 893, 833, 805, 764, 676, 560, 526, 512 cm^{-1}; HRMS (ESI+): *m/z* calcd. for $C_{16}H_{24}O_3SnNa$ [M+Na]: 407.0640; found: 407.0643.

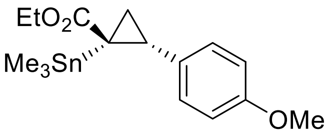

The optical purity was determined by HPLC (Chiralpak IG-3, 4.6 mm, 2 % 2-propanol in n-heptane, 1 mL·min^{-1}, 20 min, UV 230 nm): 3.36 min (major) and 3.79 min (minor) (Figure S-11).

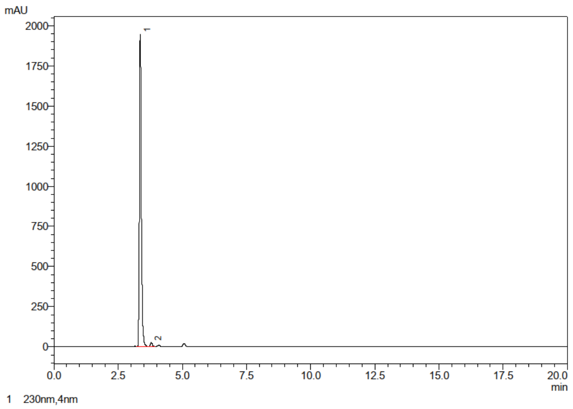

PDA Ch1 230nm

Peak #	Ret. Time	Area %	Name
1	3.36	98.64	
2	3.79	1.36	
Total		100.00	

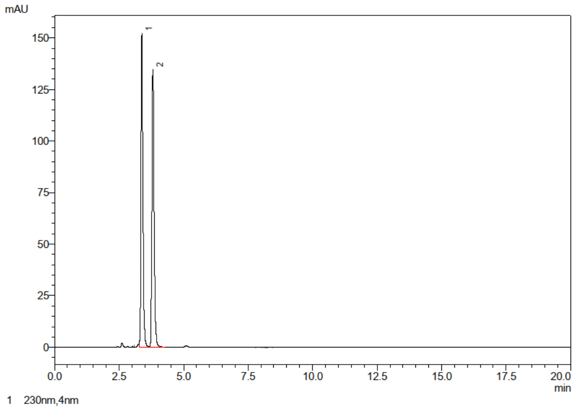

PDA Ch1 230nm

Peak #	Ret. Time	Area %	Name
1	3.38	49.88	
2	3.81	50.12	
Total		100.00	

Figure S-11. HPLC traces of *cis*-**2aa** (left) and the corresponding racemate (right).

***trans*-2aa:** Colorless oil (106 mg, 34 %, 95% *ee*). $[\alpha]_D^{20}$ = −49.6° (c = 1.00 g·100 mL^{-1}, $CHCl_3$); ^{1}H-NMR

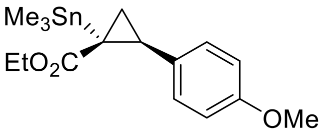

(400 MHz, $CDCl_3$): δ = 7.11 (d, *J* = 8.5 Hz, 2H), 6.80 (d, *J* = 8.5 Hz, 2H), 3.99 – 3.53 (m, 5H), 2.34 (t, *J* = 6.9 Hz, 1H), 1.90 (dd, *J* = 6.9, 4.9, 1H), 1.26 – 1.09 (m, 1H), 0.94 (t, *J* = 7.1, 3H), 0.19 (s, 9H) ppm; ^{13}C{^{1}H}-NMR (100 MHz, $CDCl_3$): δ = 172.1, 158.3, 139.6, 129.8, 128.9, 128.4, 128.2, 127.5, 124.2, 113.5, 60.3, 55.2, 27.7, 24.8, 22.1, 14.3, 14.1, 1.0, −4.1, −4.8 ppm; IR (film, ATR) $\tilde{\nu}$ = 2979, 2908, 2835, 1711, 1611, 1513, 1442, 1282, 1245, 1206, 1176, 1112, 1035, 836, 763, 529 cm^{-1}; HRMS (ESI+): *m/z* calcd. for $C_{16}H_{24}O_3SnNa$ [M+Na]: 407.0640; found: 407.0641.

The optical purity was determined by HPLC (Chiralpak IG-3, 4.6 mm, 2 % 2-propanol in n-heptane, 1 mL·min^{-1}, 20 min, UV 230 nm): 5.05 min (major) and 7.43 min (minor) (Figure S-12).

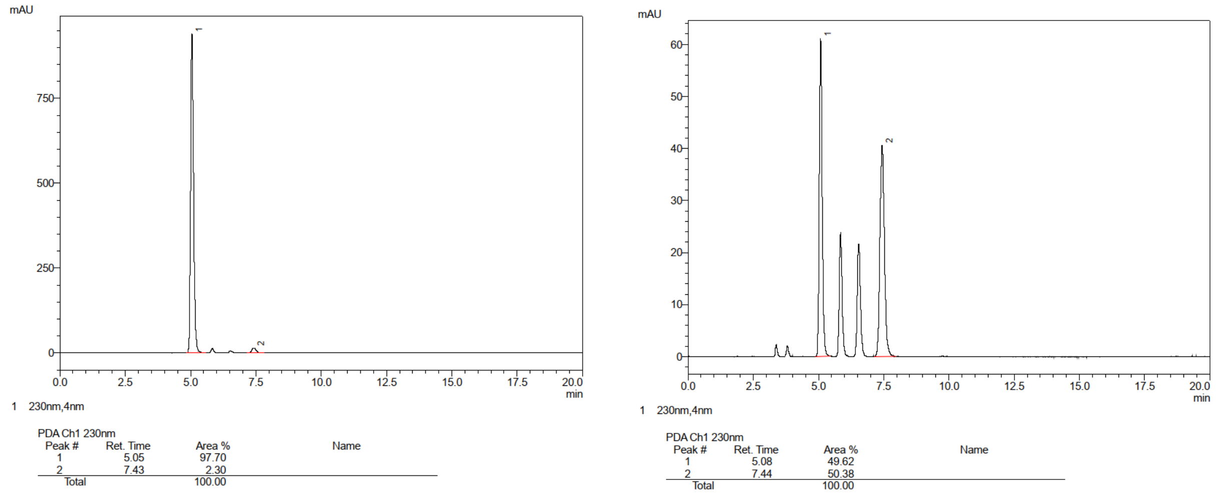

Figure S-12. HPLC traces of *trans*-**2aa** (left) and the corresponding racemate (right).

The following compounds were prepared analogously:

Ethyl 2-(phenyl)-1-(trimethylstannyl)cyclopropane-1-carboxylate (2ab). Prepared according to the representative procedure using ethyl trimethylstannyldiazoacetate **1a** (121 mg, 0.44 mmol), styrene (0.3 mL, 2.17 mmol), and **7** (6 mg, 0.005 mmol). The crude material was purified by flash chromatography (silica, hexanes/ethyl acetate, 19:1) to afford a mixture of the two diastereomers (*cis:trans* = 59:41, combined yield of both diastereomers: 121 mg, 78 %). Separation of the diastereomers was achieved by HPLC (Nucleodur C18 H-Tec, 40 x 250 mm, 10 μm, methanol-water, 85:15, retention time of trans diasteromer: 9.51 min, cis diastereomer: 13.52 min).

***trans*-2ab:** Colorless oil (20 mg, 13 % after HPLC, 93 % *ee*). $[\alpha]_D^{20}$ = −49.0° (c = 2.00 g·100 mL^{-1}, $CHCl_3$). ^{1}H-

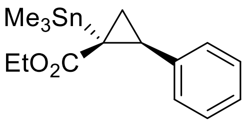

NMR (500 MHz, $CDCl_3$): δ = 7.25 – 7.13 (m, 5H), 3.88 – 3.64 (m, 2H), 2.55 – 2.28 (m, 1H), 1.96 (dd, *J* = 6.2, 5.1 Hz, 1H), 1.19 (dd, *J* = 7.6, 5.1 Hz, 1H), 0.89 (t, *J* = 7.1 Hz, 3H), 0.21 (s, 9H).ppm; ^{13}C{^{1}H}-NMR (100 MHz, $CDCl_3$): δ = 173.8, 137.9, 128.6, 128.0, 126.4, 60.4, 28.5, 22.3, 14.2, 13.9, −9.5 ppm; ^{119}Sn{^{1}H}-NMR (149 MHz, $CDCl_3$):

δ = 24.3 ppm; IR (film, ATR) $\tilde{\nu}$ = 2979, 2915, 1712, 1279, 1205, 1166, 1112, 1030, 764, 695, 528 cm^{-1}**;** HRMS (ESI+): *m/z* calcd. for $C_{15}H_{22}O_2SnNa$ [M+Na]: 377.0534; found: 377.0536.

The optical purity was determined by HPLC (150 mm Cellucoat RP, 4.6 mm, acetonitrile /water 60:40, 1 mL·min^{-1}, 15 min, UV 220 nm): 7.86 min (major) and 6.66 min (minor) (Figure S-13).

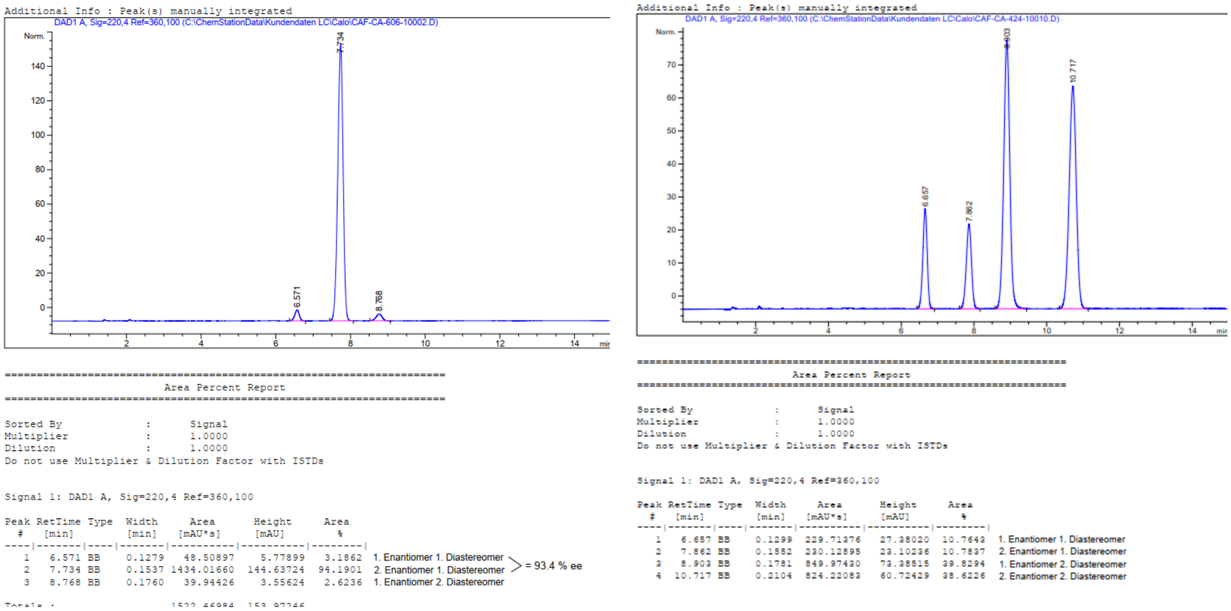

Figure S-13. HPLC traces of *trans*-**2ab** (left) and the corresponding racemate (right, both diastereomers).

***cis*-2ab:** colorless oil (24 mg, 16 % after HPLC, 97 % *ee*). $[\alpha]_D^{20}$ = +10.7° (c = 1.50 g·100 mL^{-1}, $CHCl_3$). ^1H-NMR (500 MHz, $CDCl_3$): δ = 7.31 – 7.17 (m, 5H), 4.35 – 3.97 (m, 2H), 2.97 – 2.52 (m, 1H), 1.74 (dd, *J* = 8.8, 4.1 Hz, 1H), 1.33 (dd, *J* = 6.4, 4.1 Hz, 1H), 1.28 (t, *J* = 7.1 Hz, 3H), −0.19 (s, 9H) ppm; $^{13}C\{^1H\}$-NMR (100 MHz, $CDCl_3$): δ = 177.3, 140.9, 129.6, 128.4, 127.0, 61.0, 30.9, 21.2, 16.5, 14.4, −8.5 ppm; $^{119}Sn\{^1H\}$-NMR (149 MHz, $CDCl_3$): δ = 4.6 ppm; IR (film, ATR) $\tilde{\nu}$ = 2961, 2914, 1711, 1449, 1371, 1227,1126, 765, 699, 529 cm^{-1}**;** HRMS (ESI+): *m/z* calcd. for $C_{15}H_{22}O_2SnNa$ [M+Na]: 377.0534; found: 377.0538;

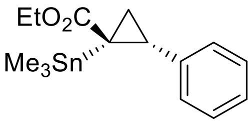

The optical purity was determined by HPLC (150 mm Cellucoat RP, 4.6 mm, acetonitrile /water 60:40, 1 mL·min^{-1}, 15 min, UV 220 nm): 8.90 min (major) and 10.72 min (minor) (Figure S-14).

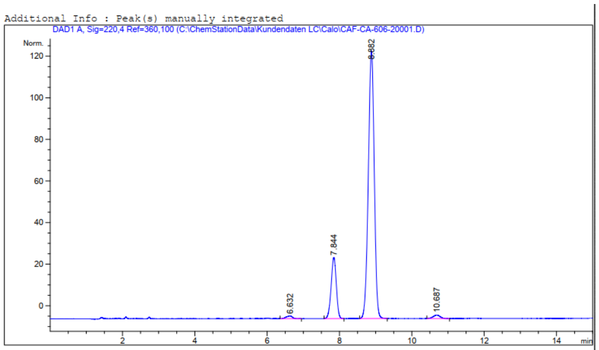

Area Percent Report

Sorted By : Signal
Multiplier : 1.0000
Dilution : 1.0000
Do not use Multiplier & Dilution Factor with ISTDs

Signal 1: DAD1 A, Sig=220,4 Ref=360,100

Peak #	RetTime [min]	Type	Width [min]	Area [mAU*s]	Height [mAU]	Area %		
1	6.632	BB	0.1558	13.75140	1.16446	0.8522	1. Enantiomer 1. Diastereomer	= 90.1 % ee
2	7.844	BB	0.1550	263.92697	26.54049	16.3557	2. Enantiomer 1. Diastereomer	
3	8.882	BB	0.1764	1315.87268	115.95706	81.5452	1. Enantiomer 2. Diastereomer	= 97.0 % ee
4	10.687	BB	0.2077	20.12216	1.48849	1.2470	2. Enantiomer 2. Diastereomer	

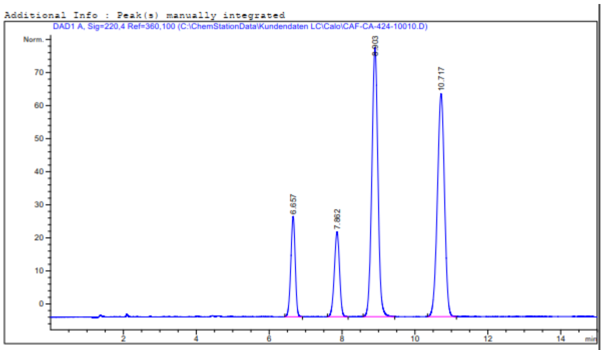

Area Percent Report

Sorted By : Signal
Multiplier : 1.0000
Dilution : 1.0000
Do not use Multiplier & Dilution Factor with ISTDs

Signal 1: DAD1 A, Sig=220,4 Ref=360,100

Peak #	RetTime [min]	Type	Width [min]	Area [mAU*s]	Height [mAU]	Area %	
1	6.657	BB	0.1299	229.71376	27.38020	10.7643	1. Enantiomer 1. Diastereomer
2	7.862	BB	0.1552	230.12895	23.10236	10.7837	2. Enantiomer 1. Diastereomer
3	8.903	BB	0.1781	849.97430	73.38515	39.8294	1. Enantiomer 2. Diastereomer
4	10.717	BB	0.2104	824.22083	60.72429	38.6226	2. Enantiomer 2. Diastereomer

Figure S-14. HPLC traces of *cis*-**2ab** (left) and the corresponding racemate (right, both diastereomers).

Ethyl 2-(4-nitrophenyl)-1-(trimethylstannyl)cyclopropane-1-carboxylate (2ac). Prepared according to the representative procedure; *cis:trans* = 51:49 (crude, NMR)

***trans*-2ac:** Off-white, crystalline solid (71 mg, 31 %, 91 % *ee*). $[\alpha]_D^{20}$ = −39.0° (c = 0.20 g·100 mL^{-1}, $CHCl_3$)

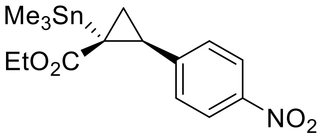

^{1}H-NMR (500 MHz, $CDCl_3$): δ = 8.10 (d, *J* = 8.8 Hz, 2H), 7.31 (d, *J* = 8.8 Hz, 2H), 3.90 – 3.69 (m, 2H), 2.49 – 2.32 (m, 1H), 2.09 – 1.93 (m, 1H), 1.37 – 1.23 (m, 1H), 0.93 (t, *J* = 7.1 Hz, 3H), 0.23 (s, 9H) ppm; ^{13}C{^{1}H}-NMR (100 MHz, $CDCl_3$): δ = 173.2, 146.4, 129.1, 123.3, 60.8, 28.2, 24.0, 15.1, 14.3, −9.4 ppm; ^{119}Sn{^{1}H}-NMR (149 MHz, $CDCl_3$): δ = 28.1 ppm; IR (film, ATR) $\tilde{\nu}$ = 2981, 2924, 2854, 1711, 1599, 1519, 1344, 1282, 1207, 1170, 1111, 858, 772, 533 cm^{-1}; HRMS (ESI+): *m/z* calcd. for $C_{15}H_{21}NO_4SnNa$ [M+Na]: 422.0385; found: 422.03877.

Single crystals for X-Ray structure determination were obtained by cooling a concentrated solution of the compound in CH_2Cl_2 to −20°C for 7 days.

The optical purity was determined by HPLC (Chiralpak IA, 4.6 mm, 5 % 2-propanol in n-heptane, 1 mL·min^{-1}, 10 min, UV 330 nm): 5.45min (major) and 7.00 min (minor) (Figure S-15).

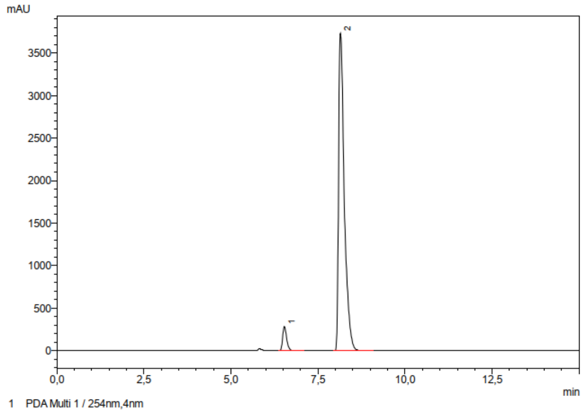

PDA Ch1 254nm

Peak #	Ret. Time	Area %	Theoretical Plate#	HETP	Tailing Factor
1	6,53	4,99	13920	0,0	1,37
2	8,14	95,01	13645	0,0	2,06
Total		100,00			

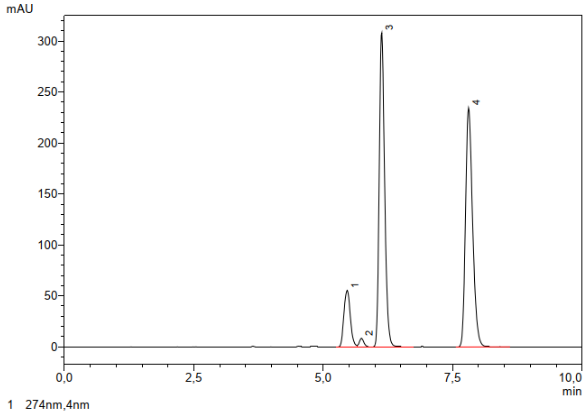

PDA Ch1 274nm

Peak #	Ret. Time	Area %	Name
1	5,46	9,55	Diatereomer 1 Enantiomer 1 u. 2
2	5,73	1,04	
3	6,12	44,73	Diastereomer 2 Enantiomer 1
4	7,80	44,69	Diastereomer 2 Enantiomer 2
Total		100,00	

Figure S-15. HPLC traces of *trans*-**2ac** (left) and the corresponding racemate (right).

***cis*-2ac:** Off-white gel (71 mg, 31 %, 82 % *ee*). $[\alpha]_D^{20}$ = +36.5° (c = 1.00 g·100 mL^{-1}, $CHCl_3$). ^{1}H-NMR (500 MHz, $CDCl_3$): δ = 8.19 – 8.08 (m, 2H), 7.44 – 7.35 (m, 2H), 4.16 (q, *J* = 7.2 Hz, 2H), 2.79 (dd, *J* = 8.7, 6.4 Hz, 1H), 1.83 (dd, *J* = 8.7, 4.3 Hz, 1H), 1.38 (dd, *J* = 6.4, 4.3 Hz, 1H), 1.29 (t, *J* = 7.1 Hz, 3H), −0.15 (s, 9H) ppm; ^{13}C{^{1}H}-NMR (100 MHz, $CDCl_3$): δ = 176.5, 148.0, 146.9, 130.4, 123.6, 61.4, 30.2, 21.6, 16.7, 14.4, −8.2 ppm; ^{119}Sn{^{1}H}-NMR (149 MHz, $CDCl_3$): δ = 7.0 ppm; IR (film, ATR) $\tilde{\nu}$ = 2982, 2911, 1708, 1600, 1519, 1344, 1226, 1108, 859, 759, 697, 529 cm^{-1}**;** HRMS (ESI+): *m/z* calcd. for $C_{15}H_{21}NO_4SnNa$ [M+Na]: 422.0385; found: 422.0385.

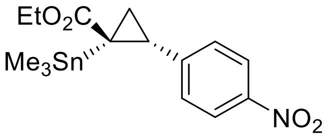

The optical purity was determined by HPLC (ChiralpakIB, 4.6 mm, 2 % 2-propanol in *n*-heptane, 1 mL·min^{-1}, 10 min, UV 254 nm): 8.14 min (major) and 6.53 min (minor) (Figure S-16).

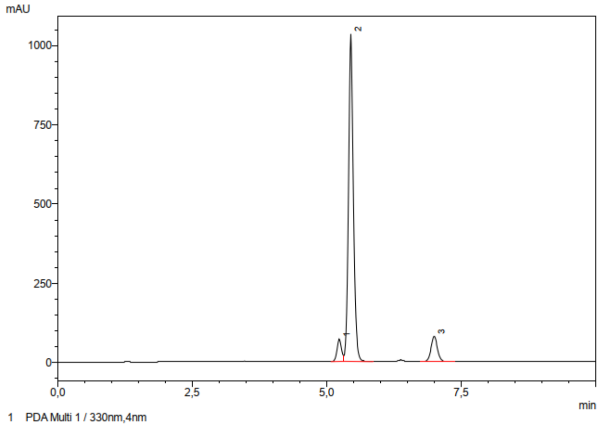

PDA Ch1 330nm

Peak #	Ret. Time	Area %	Theoretical Plate#	HETP	Tailing Factor
1	5,23	5,58	12697	0,0	0,00
2	5,45	85,89	13843	0,0	1,19
3	7,00	8,53	14745	0,0	1,10
Total		100,00			

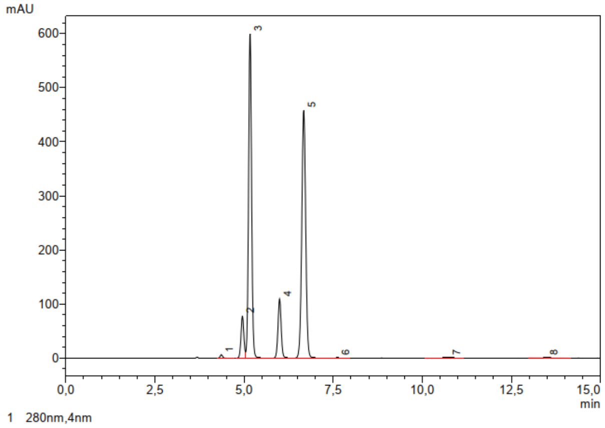

PDA Ch1 280nm

Peak #	Ret. Time	Area %	Name
1	4,36	0,37	
2	4,95	5,33	Diastereomer 2 Enantiomer 1 u. 2
3	5,16	42,73	Diastereomer 1 Enantiomer 1
4	5,99	8,32	
5	6,67	42,63	Diastereomer 1 Enantiomer 2
6	7,62	0,16	
7	10,73	0,24	
8	13,47	0,23	
Total		100,00	

Figure S-16. HPLC traces of *cis* xx (left) and the corresponding racemate (right).

Ethyl 2-(1,3-dioxoisoindolin-2-yl)-1-(trimethylstannyl)cyclopropane-1-carboxylatecarboxylate (2ad): Prepared according to the representative procedure; *cis:trans* = 16:84 (NMR); combined yield of both diastereomers: 131 mg, 0.31 mmol, 54 %,

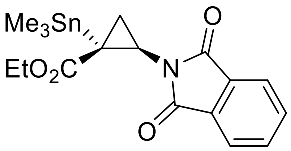

***trans*-2ad:** Colorless crystalline solid (110 mg, 45%, 96% *ee*). $[\alpha]_D^{20}$ = −54.6° (c = 1.16 g·100 mL^{-1}, $CHCl_3$). ^{1}H-NMR (400 MHz, $CDCl_3$): δ = 7.79 (dd, *J* = 5.5, 3.0 Hz, 2H), 7.68 (dd, *J* = 5.5, 3.0 Hz, 2H), 4.14 – 3.75 (m, 2H), 2.87 (dd, *J* = 6.9, 5.0 Hz, 1H), 2.21 (dd, *J* = 5.8, 5.0 Hz, 1H), 1.48 (dd, *J* = 6.9, 5.8 Hz, 1H), 1.11 (t, *J* = 7.1 Hz, 3H), 0.25 (s, 9H) ppm; ^{13}C{^{1}H}-NMR (100 MHz, $CDCl_3$): δ = 173.5, 168.9, 134.1, 131.9, 123.3, 61.2, 31.2, 16.8, 16.1, 14.1, −9.5 ppm; ^{119}Sn{^{1}H}-NMR (141 MHz, $CDCl_3$): 17.0 ppm; IR (film, ATR) $\tilde{\nu}$ = 2981, 2913, 1780, 1715, 1396, 1281, 1177, 1113, 882, 767, 715 cm^{-1}**;** HRMS (ESI+): *m/z* calcd. for $C_{17}H_{21}NO_4SnNa$ [M+Na]: 446.0385; found: 446.0389.

Single crystals for X-ray structure determination were obtained by dissolving the compound in a 9:1 mixture of hexanes/ethyl acetate and evaporating the solvent at room temperature overnight.

The optical purity was determined by HPLC (Chiralcel OJ-3R, 4.6 mm, acetonitrile/water, 85:15, 0.5 mL·min^{-1}, 25 min, UV 220 nm): 10.73 min (major) and 14.29 min (minor) (Figure S-17).

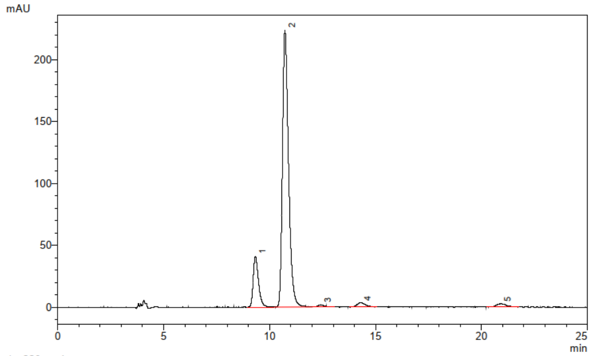

1 220nm,4nm

PDA Ch1 220nm

Peak #	Ret. Time	Area %	Name
1	9,33	13,19	Enantiomer 1 Distereomer 2
2	10,72	82,63	Enantiomer 1 Diastereomer 1 95.9 % ee
3	12,41	0,74	Enantiomer 2 Distereomer 2
4	14,29	1,75	Enantiomer 2 Diastereomer 1
5	20,90	1,68	
Total		100,00	

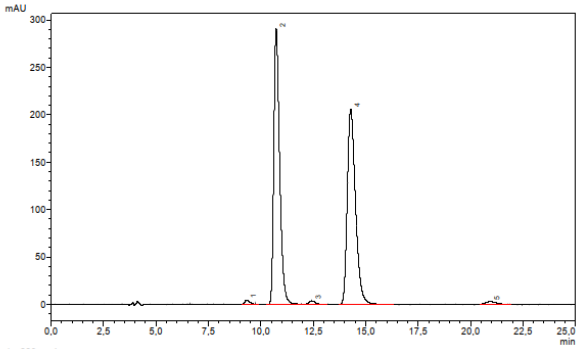

1 220nm,4nm

PDA Ch1 220nm

Peak #	Ret. Time	Area %	Name
1	9,34	0,67	Enantiomer 1 Diastereomer 2
2	10,73	48,95	Enantiomer 1 Diastereomer 1
3	12,43	0,70	Enantiomer 2 Diastereomer 2
4	14,29	48,76	Enantiomer 2 Diastereomer 1
5	20,95	0,91	
Total		100,00	

Figure S-17. HPLC traces of *trans*-**2ad** (left) and the corresponding racemate (right).

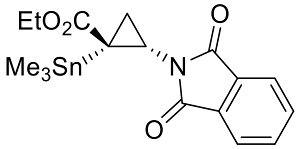

***cis*-2ad:** Colorless solid; ^{1}H-NMR (400 MHz, $CDCl_3$): δ = 7.84 (dd, *J* = 5.5, 3.0 Hz, 2H), 7.72 (dd, *J* = 5.5, 3.1 Hz, 2H), 4.16 (q, *J* = 7.1 Hz, 2H), 3.26 (dd, *J* = 8.0, 5.0 Hz, 1H), 1.85 (dd, *J* = 8.1, 5.3 Hz, 1H), 1.72 (t, *J* = 5.1 Hz, 1H), 1.29 (t, *J* = 7.1 Hz, 3H), 0.01 (s, 9H) ppm; ^{13}C{^{1}H}-NMR (100 MHz, $CDCl_3$): δ = 175.5, 168.4, 134.3, 131.6, 123.3, 61.3, 33.0, 18.8, 16.8, 14.2, −8.5 ppm; ^{119}Sn{^{1}H}-NMR (141 MHz, $CDCl_3$): 6.2 ppm; HRMS (ESI+): *m/z* calcd. for $C_{17}H_{21}NO_4SnNa$ [M+Na]: 446.0385; found: 446.0387.

Ethyl 2-(*tert*-butoxy)-1-(trimethylstannyl)cyclopropane-1-carboxylate (2ae). Prepared according to the representative procedure; *cis:trans* = 64:36 (crude, NMR)

***cis*-2ae:** Colorless oil (40 mg, 45 %, 96 % *ee*). $[\alpha]_D^{20}$ = +6.0° (c = 1.00 g·100 mL^{-1}, $CHCl_3$). ^{1}H-NMR (400 MHz, $CDCl_3$): δ = 4.35 – 3.91 (m, 2H), 3.51 (dd, *J* = 6.0, 3.8 Hz, 1H), 1.42 (dd, *J* = 6.0, 4.7 Hz, 1H), 1.24 (t, *J* = 7.1 Hz, 3H), 1.21 (s, 9H), 0.93 (dd, *J* = 4.7, 3.8 Hz, 1H), 0.14 (s, 9H) ppm; ^{13}C{^{1}H}-NMR (100 MHz, $CDCl_3$): δ = 176.4, 75.2, 60.8, 57.0, 28.2, 20.0, 14.4, −7.8 ppm; ^{119}Sn{^{1}H}-NMR (141 MHz, $CDCl_3$): δ = 4.7 ppm; IR (film, ATR) $\tilde{\nu}$ = 2977, 2932, 1711, 1365, 1252, 1191, 1125, 1067, 907, 768, 530 cm^{-1}**;** HRMS (ESI+): *m/z* calcd. for $C_{13}H_{26}O_3SnNa$ [M+Na]: 373.0796; found: 373.0796.

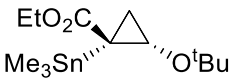

The optical purity was determined by chiral GC (BGB-176/BGB-15 0.25/0.25df G/618, 0.60 bar H_2, FID): 798,49 min (major) and 777.59 min (minor) (Figure S-18).

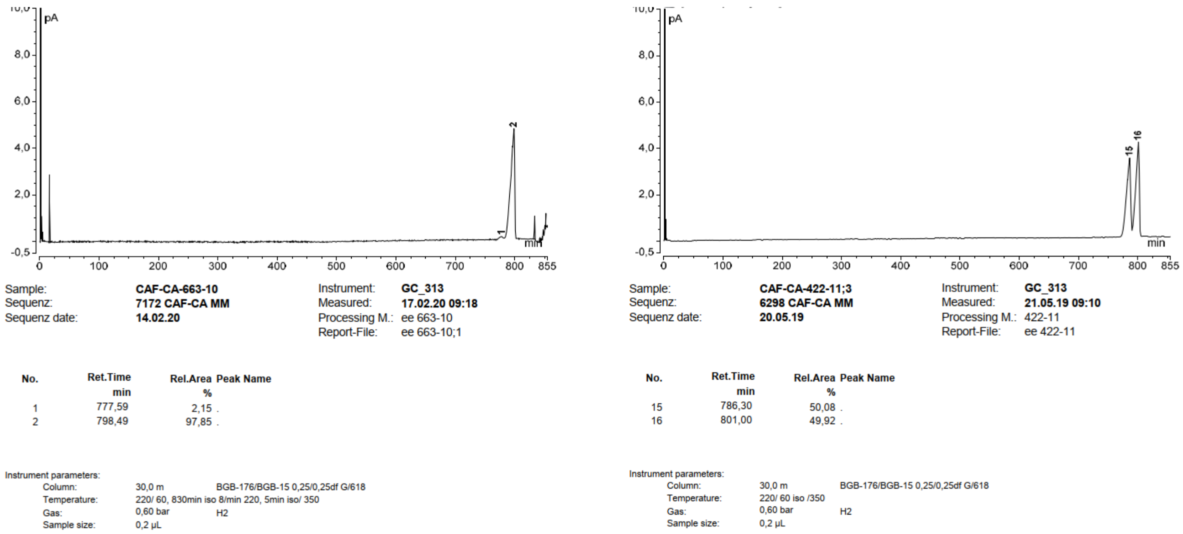

Figure S-18. GC traces of *cis*-**2ae** (left) and the corresponding racemate (right).

***trans*-2ae:** Colorless oil (13 mg, 15 %, 98 % *ee*). $[\alpha]_D^{20}$ = +16.4° (c = 0.90 g·100 mL^{-1}, $CHCl_3$). ^{1}H-NMR (500 MHz, $CDCl_3$): δ = 4.11 (ddq, *J* = 43.1, 10.8, 7.1 Hz, 2H), 3.44 (dd, *J* = 6.2, 4.1 Hz, 1H), 1.65 (dd, *J* = 5.7, 4.0 Hz, 1H), 1.25 (t, *J* = 7.1 Hz, 3H), 1.21 (s, 9H), 0.91 (m, 1H), 0.12 (s, 9H); ^{13}C{^{1}H}-NMR (100 MHz, $CDCl_3$): δ = 173.3, 75.2, 60.6, 56.2, 31.1, 28.2, 15.2, −9.6 ppm; ^{119}Sn{^{1}H}-NMR (141 MHz, $CDCl_3$): δ = 15.2 ppm; IR (film, ATR) $\tilde{\nu}$ = 2977, 2932, 1719, 1363, 1280, 1188, 1147, 1102, 1070, 991, 932, 767, 530 cm^{-1}; HRMS (ESI+): *m/z* calcd. for $C_{13}H_{26}O_3SnNa$ [M+Na]: 373.0796; found: 373.0797.

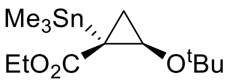

The optical purity was determined by chiral GC (Ivadex-1/PS086 0.25/0.25df G/662, 0.50 bar H_2, FID): 239.21 min (major) and 231.68 min (minor) (Figure S-19).

.

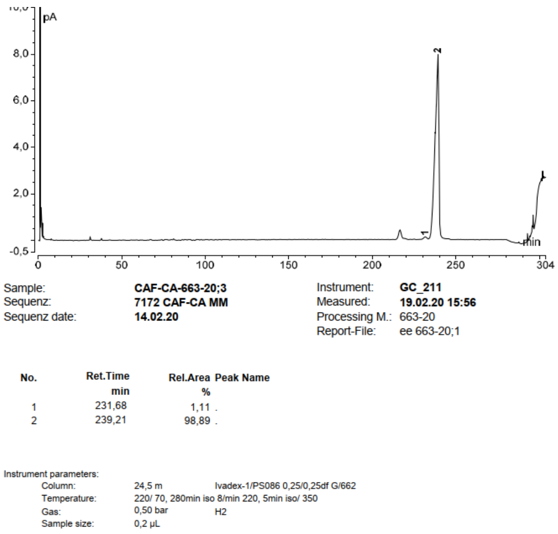

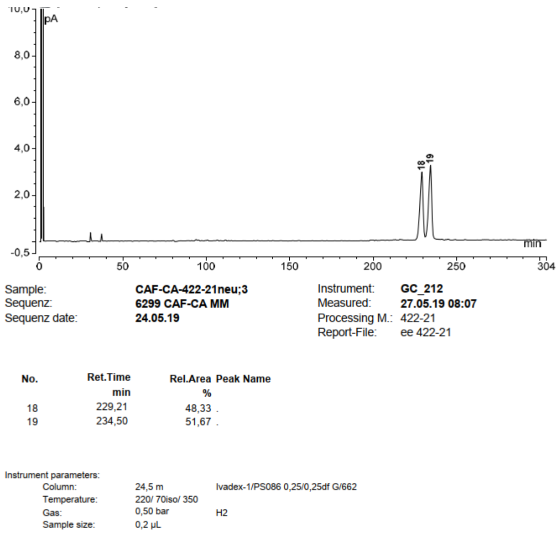

Figure S-19. GC traces of *trans*-**2ae** (left) and the corresponding racemate (right).

Ethyl 2-acetoxy-1-(trimethylstannyl)cyclopropane-1-carboxylate (2af). Prepared according to the representative procedure; *cis:trans* = 74:26 (crude, NMR)

***cis*-2af:** Colorless oil (92 mg, 47 %, 84 % *ee*). $[\alpha]_D^{20}$ = −13.5° (c = 1.09 g·100 mL^{-1}, $CHCl_3$). ^{1}H-NMR (400 MHz, $CDCl_3$): δ = 4.52 (dd, *J* = 6.9, 3.7 Hz, 1H), 4.35 – 3.81 (m, 2H), 2.01 (s, 3H), 1.70 – 1.55 (m, 1H), 1.23 (t, *J* = 7.1 Hz, 3H), 1.07 (dd, *J* = 5.5, 3.7 Hz, 1H), 0.18 (s, 9H) ppm; ^{13}C{^{1}H}-NMR (100 MHz, $CDCl_3$): δ = 173.2, 146.4, 129.1, 123.2, 60.8, 28.2, 24.0, 15.1, 14.3, −9.4 ppm; ^{119}Sn{^{1}H}-NMR (149 MHz, $CDCl_3$): δ = 10.8 ppm; IR (film, ATR) $\tilde{\nu}$ = 2983, 2913, 1754, 1711, 1365, 1211, 1118, 1058, 773, 532 cm^{-1}**;** HRMS (ESI+): *m/z* calcd. for $C_{11}H_{20}O_4SnNa$ [M+Na]: 359.0276; found: 359.0278.

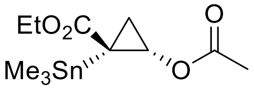

The optical purity was determined by HPLC (Chiralpak IC-3, 4.6 x 150 mm, 10 % 2-propanol in n-heptane, 12 min, UV 205 nm): 5.91min (major) and 7.58 min (minor) (Figure S-20).

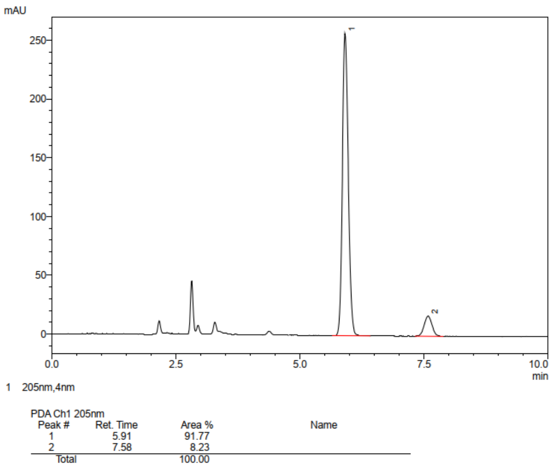

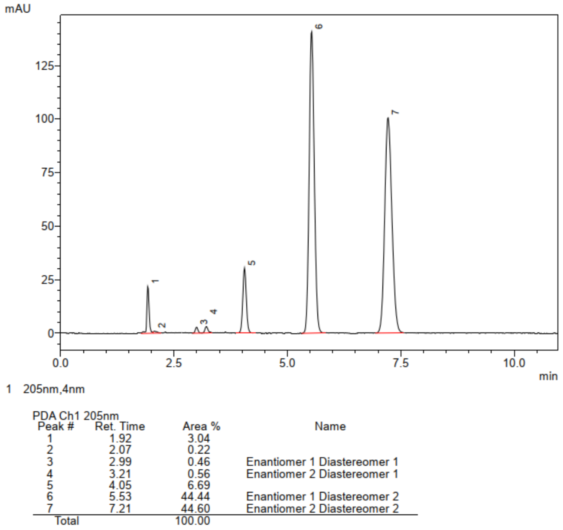

Figure S-20. HPLC traces of *cis*-**2af** (left) and the corresponding racemate (right).

***trans*-2af:** Colorless oil (24 mg, 13 %, 80 % *ee*). $[\alpha]_D^{20}$ = +25.9° (c = 0.90 g·100 mL^{-1}, $CHCl_3$). ^{1}H-NMR

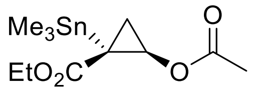

(400 MHz, $CDCl_3$): δ = 4.20 – 4.02 (m, 3H), 2.01 (s, 3H), 1.67 (dd, *J* = 6.1, 3.6 Hz, 1H), 1.23 (t, *J* = 7.1 Hz, 3H), 1.06 (t, *J* = 6.0 Hz, 1H), 0.18 (s, 9H) ppm; ^{13}C{^{1}H}-NMR (100 MHz, $CDCl_3$): δ = 172.5, 171.6, 60.9, 55.6, 31.1, 20.9, 14.6, 14.2, −9.6 ppm; ^{119}Sn{^{1}H}-NMR (149 MHz, $CDCl_3$): δ = 22.6 ppm; IR (film, ATR) $\tilde{\nu}$ = 2982, 2917, 1751, 1716, 1364, 1285, 1226, 1184, 1105, 771, 532 cm^{-1}**;** HRMS (ESI+): *m/z* calcd. for $C_{11}H_{20}O_4SnNa$ [M+Na]: 359.0276; found: 359.0274.

The optical purity was determined by HPLC (Chiralpak IC-3, 4.6 x 150 mm, 10 % 2-propanol in n-heptane,, 12 min, UV 205 nm): 3.27min (major) and 3.49 min (minor) (Figure S-21).

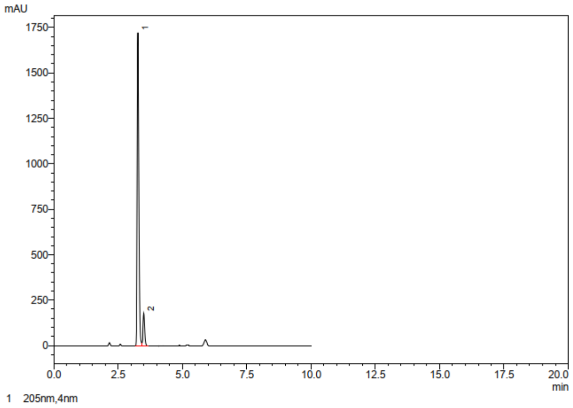

PDA Ch1 205nm

Peak #	Ret. Time	Area %	Name
1	3.27	89.99	
2	3.49	10.01	
Total		100.00	

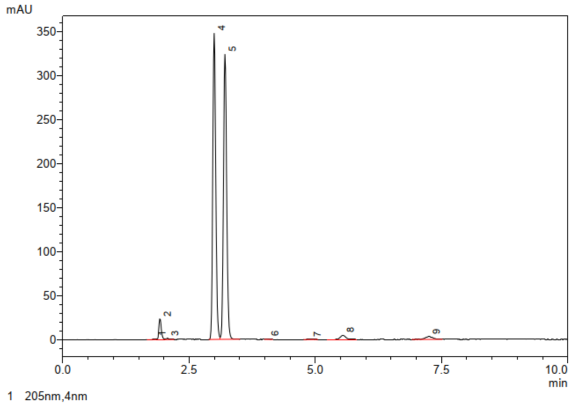

PDA Ch1 205nm

Peak #	Ret. Time	Area %	Name
1	1.81	0.14	
2	1.92	2.66	
3	2.07	0.25	
4	2.99	46.88	Enantiomer 1 Diastereomer 1
5	3.21	47.41	Enantiomer 2 Diastereomer 1
6	4.05	0.10	
7	4.89	0.12	
8	5.55	1.22	Enantiomer 1 Diastereomer 2
9	7.25	1.22	Enantiomer 2 Diastereomer 2
Total		100.00	

Figure S-21. HPLC traces of *trans*-**2af** (left) and the corresponding racemate (right).

Ethyl 1-(trimethylstannyl)-1,1a,6,6a-tetrahydrocyclopropa[a]indene-carboxylate (2ag). Prepared according to the representative procedure; *cis:trans* = 53:47 (crude, NMR).

***cis*-2ag:** Colorless oil (19 mg, 31 %, 93 % *ee*). $[\alpha]_D^{20}$ = +89.0° (c = 0.70 g·100 mL^{-1}, $CHCl_3$). ^{1}H-NMR (500 MHz,

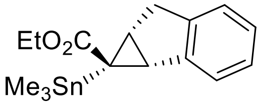

$CDCl_3$): δ = 7.39 – 7.32 (m, 1H), 7.19 – 7.10 (m, 3H), 4.13 (q, *J* = 7.2, 2H), 3.30 (dd, *J* = 17.9, 6.8, 1H), 3.02 (dd, *J* = 6.6, 1.6, 1H), 2.97 – 2.89 (m, 1H), 2.60 (td, *J* = 6.7, 0.9, 1H), 1.29 (t, *J* = 7.2, 3H), −0.17 (s, 9H) ppm; ^{13}C{^{1}H}-NMR (100 MHz, $CDCl_3$): δ = 175.8, 144.0, 143.8, 126.5, 126.5, 125.8, 124.6, 60.9, 37.2, 35.6, 30.1, 29.9, 14.3, −7.5 ppm; ^{119}Sn{^{1}H}-NMR (149 MHz, $CDCl_3$): δ = −5.8 ppm; IR (film, ATR) $\tilde{\nu}$ = 3043, 3023, 2980, 2908, 2841, 1706, 1234, 1210, 1193, 1053, 767, 735, 528 cm^{-1}; HRMS (ESI+): *m/z* calcd. for $C_{16}H_{22}O_2SnNa$ [M+Na]: 389.0534 found: 389.0536.

The optical purity was determined by HPLC (Chiralcel OJ-3R, 4.6 x 150 mm, methanol-water 90:10, 0.5 mL·min^{-1}, 25 min, UV 220 nm): 22.18 min (major) and 10.40 min (minor) (Figure S-22).

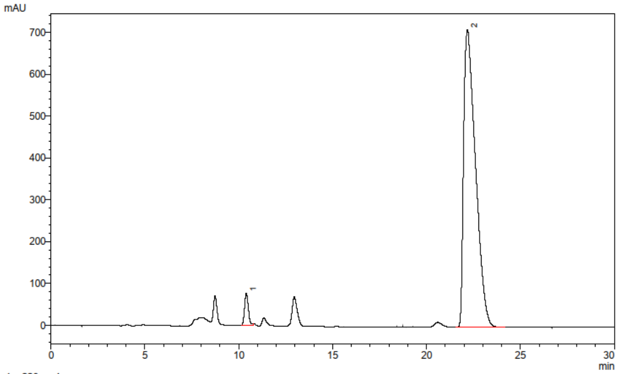

1 220nm,4nm

PDA Ch1 220nm

Peak #	Ret. Time	Area %	Name
1	10.40	3.45	
2	22.18	96.55	
Total		100.00	

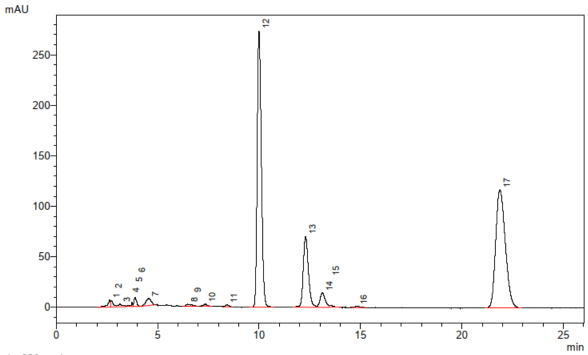

1 220nm,4nm

PDA Ch1 220nm

Peak #	Ret. Time	Area %	Name
1	2.63	0.66	
2	2.72	0.62	
3	3.14	0.45	
4	3.59	0.10	
5	3.74	0.19	
6	3.88	0.81	
7	4.55	1.33	
8	6.47	0.26	
9	6.63	0.15	
10	7.34	0.18	Enantiomer 1 Diastereomer 1
11	8.41	0.24	Enantiomer 2 Diastereomer 1
12	9.99	39.65	Enantiomer 1 Diastereomer 2
13	12.29	12.69	
14	13.12	2.60	
15	13.44	0.34	
16	14.81	0.23	
17	21.87	39.50	Enantiomer 2 Diastereomer 2
Total		100.00	

Figure S-22. HPLC traces of *cis*-**2ag** (left) and the corresponding racemate (right).

***trans*-2ag:** Colorless oil (23 mg, 44 %, 84 % *ee*). $[\alpha]_D^{20}$ = −101.3° (c = 0.30 g·100 mL^{-1}, $CHCl_3$). ^{1}H-NMR (500 MHz, $CDCl_3$): δ = 7.34 – 7.29 (m, 1H), 7.15 – 7.03 (m, 3H), 3.75 – 3.61 (m, 2H), 3.35 (dd, *J* = 17.1, 1.2, 1H), 3.17 (dd, *J* = 17.1, 6.5, 1H), 2.69 (dd, *J* = 5.3, 1.6, 1H), 2.12 (ddd, *J* = 6.4, 5.3, 0.9, 1H), 0.76 (t, *J* = 7.1, 3H), 0.24 (s, 9H); ^{13}C{^{1}H}-NMR (100 MHz, $CDCl_3$): δ = 172.2, 143.8, 141.8, 125.9, 125.9, 125.2, 124.76, 59.4, 33.2, 33.0, 27.9, 24.8, 14.1, −9.7 ppm; ^{119}Sn{^{1}H}-NMR (149 MHz, $CDCl_3$): δ = 33.9 ppm; IR (film, ATR) $\tilde{\nu}$ = 3021, 2978, 2925, 1703, 1461, 1280, 1176, 1139, 771, 723, 532 cm^{-1}; HRMS (ESI+): *m/z* calcd. for $C_{16}H_{22}O_2SnNa$ [M+Na]: 389.0534 found: 389.0532.

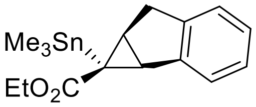

The optical purity was determined by HPLC (Chiralcel OJ-3R, 4.6 x 150 mm, methanol-water 90:10, 0.5 mL·min^{-1}, 25 min, UV 220 nm): 8.68 min (major) and 7.59 min (minor) (Figure S-23).

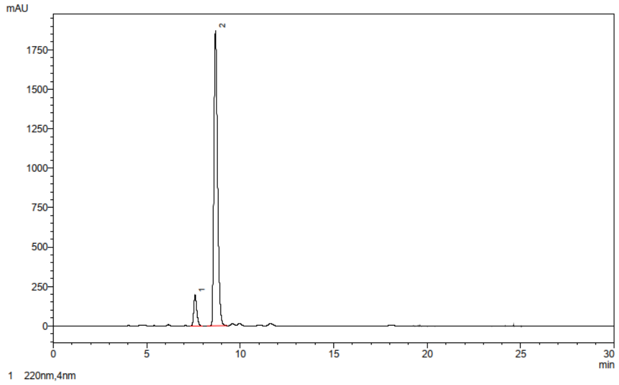

1 220nm,4nm

PDA Ch1 220nm

Peak #	Ret. Time	Area %	Name
1	7.59	7.97	
2	8.68	92.03	
Total		100.00	

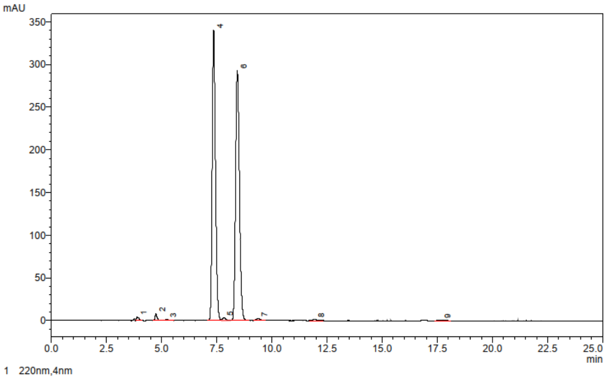

1 220nm,4nm

PDA Ch1 220nm

Peak #	Ret. Time	Area %	Name
1	3.89	0.42	
2	4.74	0.68	
3	5.23	0.15	
4	7.36	48.68	Enantiomer 1 Diastereomer 1
5	7.81	0.49	
6	8.43	48.69	Enantiomer 2 Diastereomer 1
7	9.37	0.44	
8	11.94	0.25	
9	17.67	0.20	
Total		100.00	

Figure S-23. HPLC traces of *trans*-**2ag** (left) and the corresponding racemate (right).

Ethyl 2-(4-methoxyphenyl)-1-(trimethylgermyl)cyclopropane-1-carboxylate (2ba): Prepared according to the representative procedure but using ethyl trimethylgermyldiazoacetate **1b**; dr = 1:1 (crude, NMR).

cis-2ba: Colorless oil (8 mg, 15 %, 97 % *ee*): ^{1}H-NMR (500 MHz, $CDCl_3$): δ = 7.19 – 7.13 (m, 2H), 6.88 – 6.70 (m, 2H), 4.25 – 4.07 (m, 2H), 3.79 (s, 3H), 2.70 (dd, *J* = 8.9, 6.7 Hz, 1H), 1.67 (dd, *J* = 9.0, 4.0 Hz, 1H), 1.29 (t, *J* = 7.1 Hz, 3H), 1.26 – 1.19 (m, 1H), −0.08 (s, 9H) ppm; ^{13}C{^{1}H}-NMR (100 MHz, $CDCl_3$): δ = 176.5, 158.7, 131.1, 130.6, 113.5, 60.8, 55.4, 31.2, 22.1, 15.9, 14.4, −11 ppm; IR (film, ATR) $\tilde{\nu}$ = 2927, 1710, 1515, 1235, 834, 603, 516, 467, 418 cm^{-1}; HRMS (ESI+): *m/z* calcd. for $C_{16}H_{24}O_3GeNa$ [M+Na]: 361.0829; found: 361.0832.

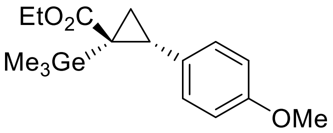

The optical purity was determined by HPLC (Chiralpak IG-3, 4.6 mm, 2 % 2-propanol in n-heptane, 1 mL·min^{-1}, 10 min, UV 230 nm): 4.06 min (major) and 4.75 min (minor) (Figure S-24).

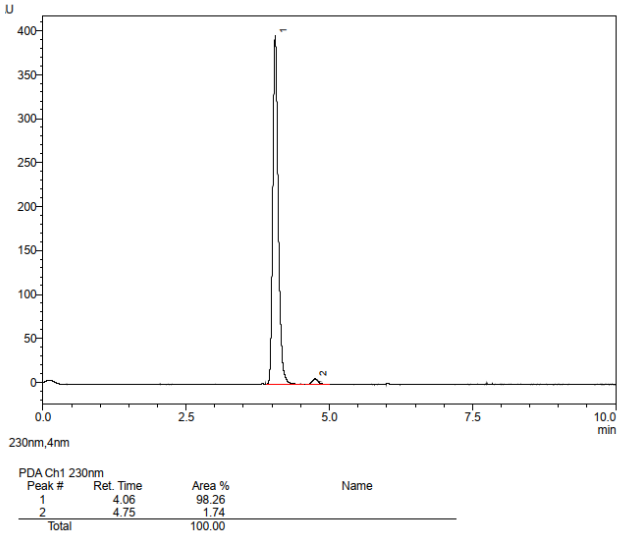

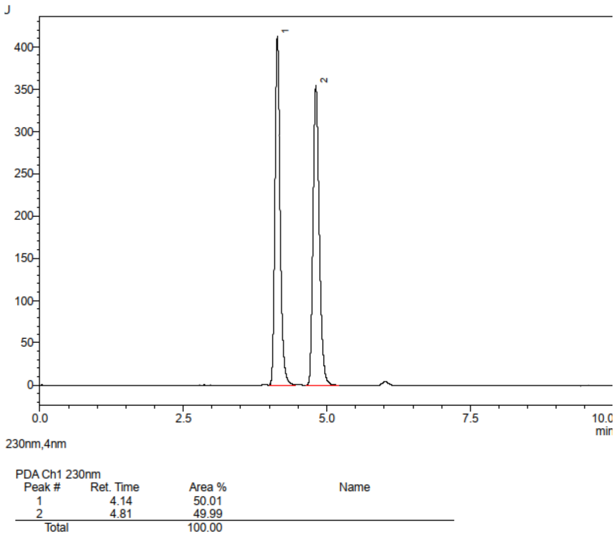

Figure S-24. HPLC traces of *cis*-**2ba** (left) and the corresponding racemate (right).

***trans*-2ba:** Colorless oil (8 mg, 14 %, 92 % *ee*): ^{1}H-NMR (500 MHz, $CDCl_3$): δ = 7.17 – 7.04 (m, 2H), 6.86 – 6.68 (m, 2H), 3.89 – 3.77 (m, 2H), 3.77 (s, 3H), 2.31 – 2.23 (m, 1H), 1.86 (dd, *J* = 6.5, 5.0 Hz, 1H), 1.14 (dd, *J* = 7.9, 5.0 Hz, 1H), 0.95 (t, *J* = 7.1 Hz, 3H), 0.26 (s, 9H).; ^{13}C{^{1}H}-NMR (100 MHz, $CDCl_3$): δ = 173.0, 158.3, 131.1, 129.8, 113.5, 113.5, 60.3, 55.4, 27.5, 14.3, 13.9, −2.6 ppm**;** IR (film, ATR) $\tilde{\nu}$ = 2956, 2923, 2853, 1708, 1657, 1515, 1465, 1377, 1287, 1249, 1040, 847, 605, 546, 478, 450 cm^{-1}**;** HRMS (ESI+): *m/z* calcd. for $C_{16}H_{24}O_3GeNa$ [M+Na]: 361.0829; found: 361.0831.

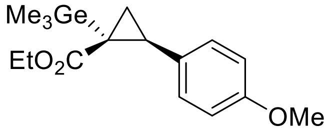

The optical purity was determined by HPLC (Chiralpak IG-3, 4.6 mm, 2 % 2-propanol in n-heptane, 1 mL·min^{-1}, 10 min, UV 230 nm): 7.27 min (major) and 9.55 min (minor) (Figure S-25).

.

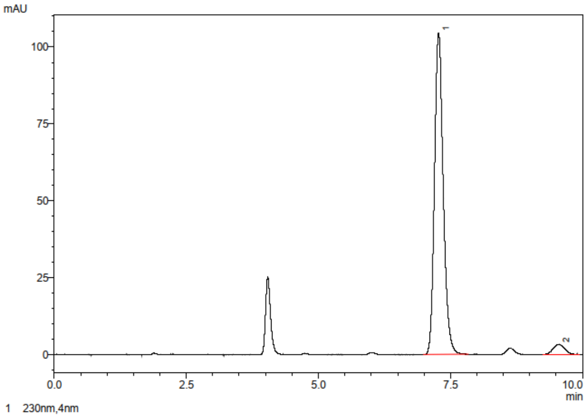

PDA Ch1 230nm

Peak #	Ret. Time	Area %	Name
1	7.27	96.01	
2	9.55	3.99	
Total		100.00	

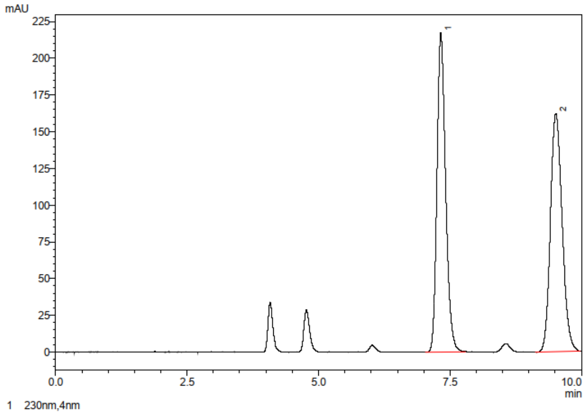

PDA Ch1 230nm

Peak #	Ret. Time	Area %	Name
1	7.33	50.16	
2	9.51	49.84	
Total		100.00	

Figure S-25. HPLC traces of *trans*-**2ba** (left) and the corresponding racemate (right).

Ethyl 2-(4-methoxyphenyl)-1-(trimethylsilyl)cyclopropane-1-carboxylate (2ca). Prepared according to the representative procedure but using ethyl trimethylsilyldiazoacetate (**1c**); dr = 1:1 (crude, NMR).

***cis*-2ca:** Colorless oil (26 mg, 24 %, 94 % *ee*). $[\alpha]_D^{20}$ = +28.3° (c = 1.00 g·100 mL^{-1}, $CHCl_3$). ^{1}H-NMR (400 MHz, $CDCl_3$): δ = 7.18 (d, *J* = 8.7 Hz, 2H), 6.80 (d, *J* = 8.7 Hz, 2H), 4.23 – 4.10 (m, 2H), 3.79 (s, 3H), 2.93 – 2.63 (m, 1H), 1.66 (dd, *J* = 8.8, 3.9 Hz, 1H), 1.30 (m, 4H), −0.20 (s, 9H) ppm; ^{13}C{^{1}H}-NMR (100 MHz, $CDCl_3$): δ = 176.3, 158.7, 131.2, 129.9, 113.5, 60.8, 55.4, 32.2, 20.1, 16.3, 14.4, −0.8 ppm; IR (film, ATR) $\tilde{\nu}$ = 2955, 2903, 2837, 1708, 1612, 1514, 1229, 1173, 1139, 1032, 833, 563 cm^{-1}; HRMS (ESI): *m/z* calcd. for $C_{16}H_{24}O_3SiNa$ [M+Na]: 315.1387; found: 315.1390.

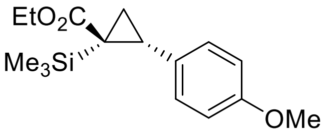

The optical purity was determined by HPLC (Chiralpak IG, 3.0 mm, 2 % 2-propanol in n-heptane, 1 mL·min^{-1}, 15 min, UV 230 nm): 3.19 min (major) and 3.57min (minor) (Figure S-26).

.

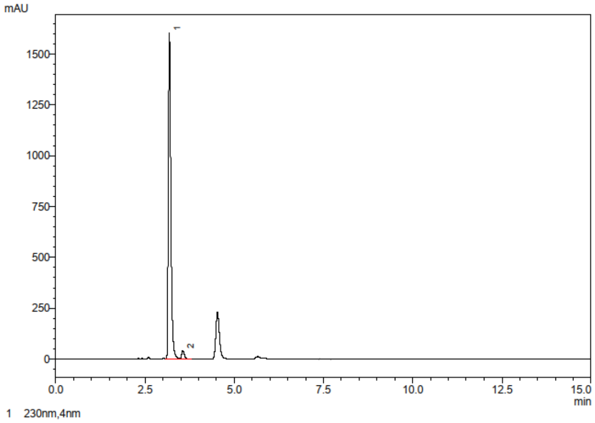

PDA Ch1 230nm

Peak #	Ret. Time	Area %	Name
1	3.19	97.08	
2	3.57	2.92	
Total		100.00	

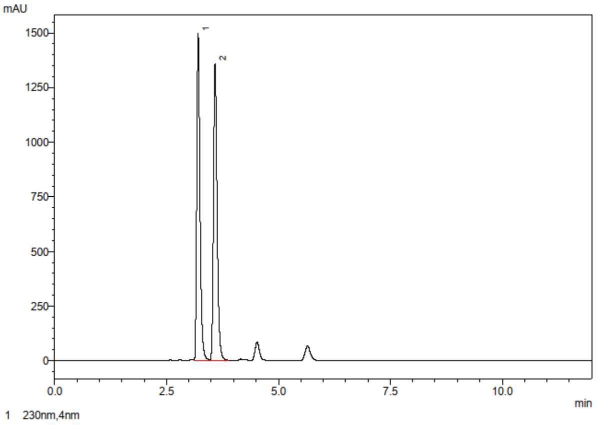

PDA Ch1 230nm

Peak #	Ret. Time	Area %	Name
1	3.21	49.74	
2	3.59	50.26	
Total		100.00	

Figure S-26. HPLC traces of *cis*-**2ca** (left) and the corresponding racemate (right).

***trans*-2ca:** colorless oil (24 mg, 22 %, 87 % *ee*). $[\alpha]_D^{20}$ = −41.8° (c = 0.60 g·100 mL^{-1}, $CHCl_3$). ^{1}H-NMR (500 MHz, $CDCl_3$): δ = 7.18 – 7.06 (m, 2H), 6.78 (d, *J* = 8.7 Hz, 2H), 4.02 – 3.54 (m, 5H), 2.32 (dd, *J* = 7.8, 6.6 Hz, 1H), 1.88 (dd, *J* = 6.6, 4.8 Hz, 1H), 1.16 (dd, *J* = 7.8, 4.8 Hz, 1H), 0.95 (t, *J* = 7.1 Hz, 3H), 0.13 (s, 9H) ppm; ^{13}C{^{1}H}-NMR (100 MHz, $CDCl_3$): δ = 172.6, 158.4, 129.9, 129.4, 113.4, 60.3, 55.4, 27.8, 23.1, 14.4, 14.2, −2.2 ppm. IR (film, ATR) $\tilde{\nu}$ = 2979, 2908, 2835, 1711, 1611, 1513, 1442, 1282, 1245, 1206, 1176, 1112, 1035, 836, 763, 529 cm^{-1}; HRMS (ESI): *m/z* calcd. for $C_{16}H_{24}O_3SiNa$ [M+Na]: 315.1387.

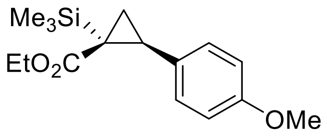

The optical purity was determined by HPLC (Chiralpak IG, 3.0 mm, 2 % 2-propanol in *n*-heptane, 1 mL·min^{-1}, 15 min, UV 230 nm): 4.52 min (major) and 5.65min (minor) (Figure S-27).

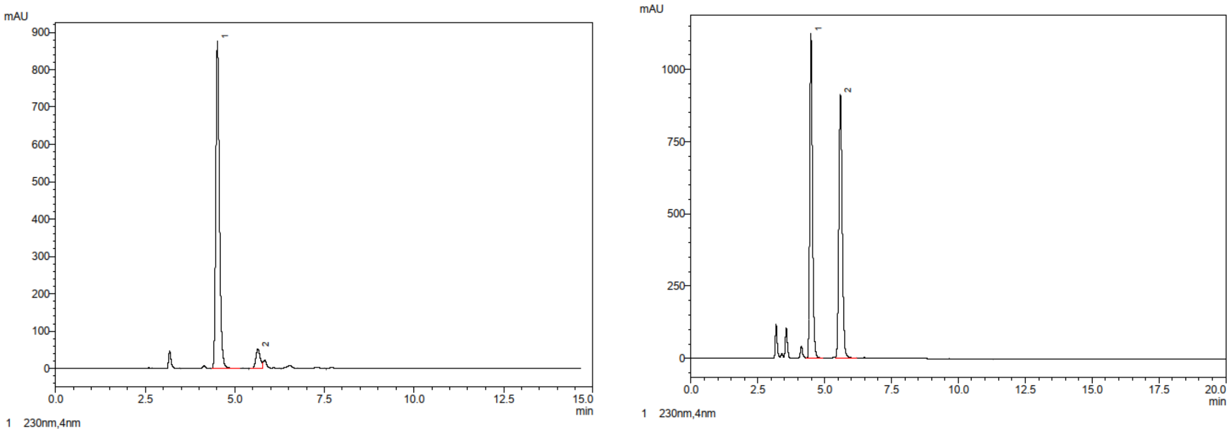

PDA Ch1 230nm

Peak #	Ret. Time	Area %	Name
1	4.52	93.41	
2	5.65	6.59	
Total		100.00	

PDA Ch1 230nm

Peak #	Ret. Time	Area %	Name
1	4.49	49.69	
2	5.59	50.31	
Total		100.00	

Figure S-27. HPLC traces of *trans*-**2ca** (left) and the corresponding racemate (right).

Ethyl 2-(4-methoxyphenyl)cyclopropane-1-carboxylate (14). Prepared according to the representative procedure; *cis:trans* = 1:1 (crude, NMR).

***trans*-14: C**olorless solid (45 mg, 43 %, 19 % *ee*). ^{1}H-NMR (400 MHz, $CDCl_3$): δ = 7.10 – 6.99 (m, 2H), 6.86 – 6.76 (m, 2H), 4.16 (q, *J* = 7.1 Hz, 2H), 3.78 (s, 3H), 2.48 (ddd, *J* = 9.1, 6.5, 4.1 Hz, 1H), 1.82 (ddd, *J* = 8.4, 5.2, 4.1 Hz, 1H), 1.60 – 1.50 (m, 1H), 1.28 (m, 4H) ^{13}C{^{1}H}-NMR (100 MHz, $CDCl_3$): δ = 173.6, 158.4, 132.2, 127.5, 114.0, 60.7, 55.4, 25.7, 24.0, 16.8, 14.4 ppm**.**

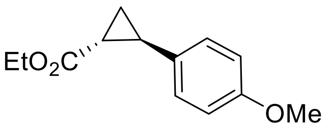

The optical purity was determined by HPLC (Chiralcel IA, 4.6 x 250 mm, 2 % isopropanol in *n*-heptane, 1 mL·min^{-1}, 10 min, UV 230 nm): 6.92 min (major) and 7.20 min (minor) (Figure S-28). The analytical data are consistent with those reported in the literature.[7]

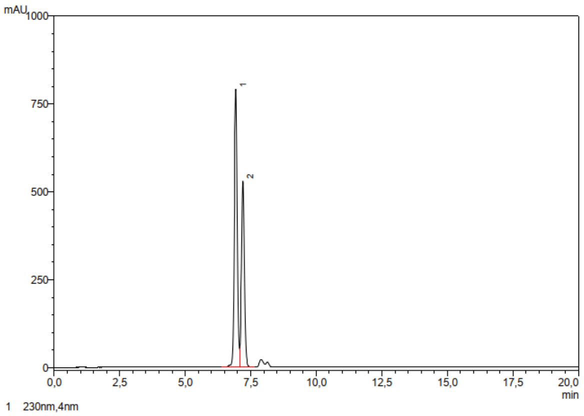

PDA Ch1 230nm

Peak #	Ret. Time	Area %
1	6,92	59,50
2	7,20	40,50
Total		100,00

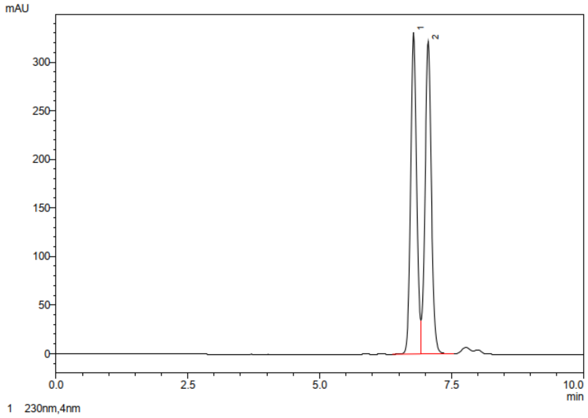

PDA Ch1 230nm

Peak #	Ret. Time	Area %	Name
1	6.78	49.68	
2	7.05	50.32	
Total		100.00	

Figure S-28. HPLC traces of *trans*-**14** (left) and the corresponding racemate (right).

***cis*-14:** Colorless liquid (43 mg, 41 %, 13 % *ee*). ^{1}H-NMR (400 MHz, $CDCl_3$): δ = 7.22 – 7.14 (m, 2H), 6.84 – 6.77 (m, 2H), 3.89 (q, *J* = 7.1 Hz, 2H), 3.77 (s, 3H), 2.52 (td, *J* = 8.8, 7.4 Hz, 1H), 2.03 (ddd, *J* = 9.1, 7.8, 5.6 Hz, 1H), 1.66 (ddd, *J* = 7.4, 5.6, 5.0 Hz, 1H), 1.37 – 1.22 (m, 1H), 1.02 (t, *J* = 7.1 Hz, 3H) ppm; $^{13}C\{^1H\}$-NMR (100 MHz, $CDCl_3$): δ = 171.2, 158.4, 130.4, 128.7, 113.4, 60.3, 55.3, 25.0, 21.8, 14.2, 11.4 ppm.

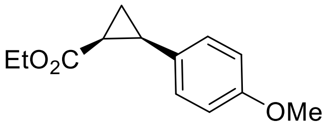

The optical purity was determined by HPLC (Chiralcel IA, 4.6 x 250 mm, 2 % isopropanol in *n*-heptane, 1 mL·min^{-1}, 10 min, UV 230 nm): 7.80 min (major) and 8.05 min (minor) (Figure S-29). The analytical data are consistent with those reported in the literature.[7]

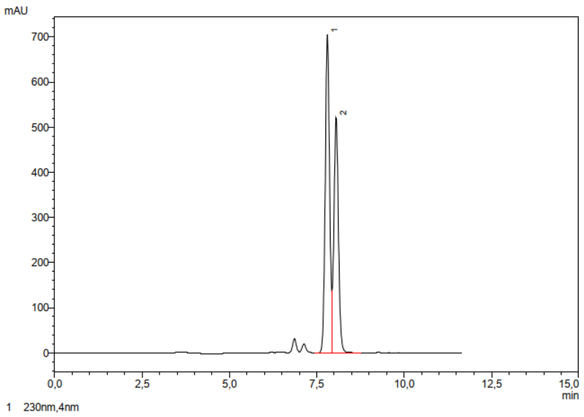

PDA Ch1 230nm

Peak #	Ret. Time	Area %	Name
1	7,80	56,74	
2	8,05	43,26	
Total		100,00	

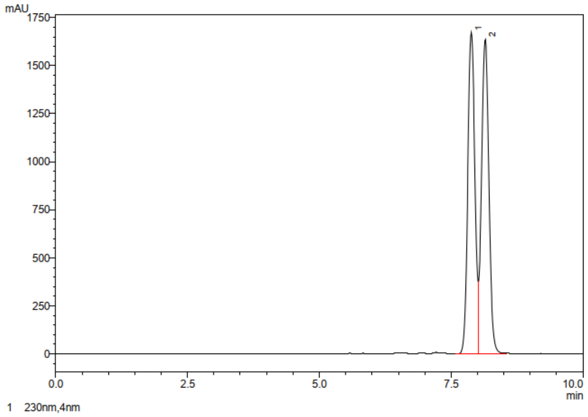

PDA Ch1 230nm

Peak #	Ret. Time	Area %	Name
1	7.88	49.18	
2	8.14	50.82	
Total		100.00	

Figure S-29. HPLC traces of *cis*-**14** (left) and the corresponding racemate (right).

Methyl (1S,2R)-1-(4-methoxyphenyl)-2-phenylcyclopropane-1-carboxylate (13): A solution of methyl 2-diazo-2-(4-methoxyphenyl)acetate (260 mg, 0.94 mmol) in CH_2Cl_2 (5 mL) was added over 6 h via syringe pump to a mixture of styrene (0.6 mL, 4.66 mmol) and **7** (12 mg, 0.01 mmol) in CH_2Cl_2 (5 mL) at the indicated temperature. Once the addition was complete, the green mixture was stirred for additional 6 h at this temperature (before it was warmed up to room temperature when the reaction was performed under cryogenic conditions). The mixture was concentrated under reduced pressure and the residue purified by flash chromatography (silica, hexanes/ethyl acetate, 20:1) to give the title compound as a colorless solid. Reaction at ambient temperature: 48 mg, 70 %, *trans:cis* = 88:12, 57% *ee*; reaction at –78°C: 53 mg, 78 %, *trans:cis* = 81:19, 84% *ee*. Analytical data is given for the major diastereomer (@ RT): $[\alpha]_D^{20}$ = +10.2° (c = 0.93 g·100 mL^{-1}, $CHCl_3$) [Lit.[8]: $[\alpha]_D^{20}$ = +5.1° (c = 1.00 g·100 mL^{-1}, $CHCl_3$, 96 % *ee*]. ^1H-NMR (400 MHz, $CDCl_3$): δ = 7.07 (m, 3H), 7.00 – 6.87 (m, 2H), 6.84 – 6.74 (m, 2H), 6.73 – 6.61 (m, 2H), 3.72 (s, 3H), 3.66 (s, 3H), 3.07 (dd, *J* = 9.3, 7.3 Hz, 1H), 2.12 (dd, *J* = 9.3, 4.8 Hz, 1H), 1.82 (dd, *J* = 7.3, 4.8 Hz, 1H) ppm; $^{13}C\{^1H\}$-NMR (100 MHz, $CDCl_3$): δ = 174.8, 158.5, 136.6, 133.1, 128.2, 127.9, 126.9, 126.4, 113.3, 55.2, 52.8, 36.8, 33.4, 20.9 ppm;

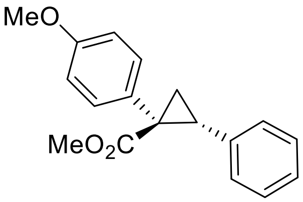

The optical purity was determined by HPLC (Chiralcel IB-N, 3 μm, 4.6 x 150 mm, 2 % isopropanol in *n*-heptane, 1 mL·min^{-1}, UV 225nm): 5.51 min (major) and 6.31 min (minor) (Figure S-30). The analytical data are consistent with those reported in the literature.[8]

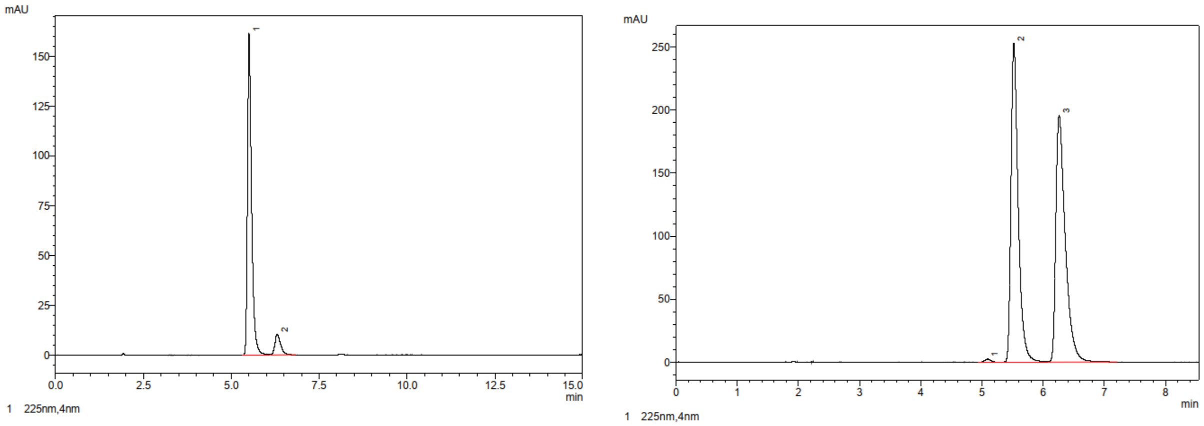

PDA Ch1 225nm

Peak #	Ret. Time	Area %	Name
1	5.51	91.76	
2	6.31	8.24	
Total		100.00	

PDA Ch1 225nm

Peak #	Ret. Time	Area %	Name
1	5,09	0,39	
2	5,52	49,52	1. Enantiomer
3	6,26	50,09	2. Enantiomer
Total		100,00	

Figure S-30. HPLC traces of *trans*-**13** prepared at −78°C (left) and the corresponding racemate (right).

Cross Coupling of Cyclopropylstannanes

Representative Procedure: Ethyl *cis*-2-(4-methoxyphenyl)-1-(phenyl)cyclopropane-1-carboxylate (*cis*-15):

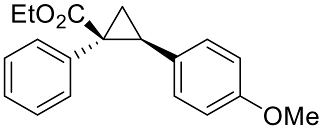

An oven-dried 10 mL glass vial was charged with $Pd(dba)_2$ (5 mg, 0.008 mmol), JackiePhos (**18**) (12 mg, 0.016 mmol), KF (9 mg, 0.16 mmol), and CuCl (16 mg, 0.16 mmol). The vial was closed with a septum cap and evacuated. After 15 min the vial was refilled with Ar, followed by two additional vacuum/Ar cycles before THF (1 mL) was added.

Another oven-dried 10 mL glass vial was charged with *trans*-**2aa** (30 mg, 0.08 mmol), iodobenzene (18 μL, 0.16 mmol) and THF (1.0 mL) under Ar atmosphere. The resulting solution was transferred via syringe into the vial containing the catalyst-solution and the resulting red-brown reaction mixture was stirred at 60°C for 18 h. After GC-MS indicated that all stannylcyclopropane had been consumed, the mixture was cooled to room temperature before it was diluted with *tert*-butyl methyl ether (2 mL). The mixture was filtered through a pipette with a glass fibre filter, the filter was washed with additional *tert*-butyl methyl ether (1 mL) and the combined filtrates were concentrated under reduced pressure. The residue was purified by flash chromatography (silica, hexanes/ethyl acetate 49:1) to afford the title compound as a yellow oil (17 mg, 73%). $[\alpha]_D^{20}$ = −65.5° (c = 1.0 g·100 mL^{-1}, $CHCl_3$). ^{1}H-NMR (400 MHz, $CDCl_3$): δ = 7.60 – 7.46 (m, 2H), 7.39 – 7.32 (m, 2H), 7.32 – 7.25 (m, 3H), 6.92 – 6.75 (m, 2H), 3.84 (m, 1H), 3.80 (s, 3H), 3.71 (m, 1H), 2.93 – 2.65 (m, 1H), 2.29 (dd, *J* = 7.4, 5.0 Hz, 1H), 1.58 (dd, *J* = 9.0, 5.0 Hz, 1H), 0.84 (t, *J* = 7.1 Hz, 3H) ppm; ^{13}C{^{1}H}-NMR (100 MHz, $CDCl_3$): δ = 170.8, 158.6, 140.6, 130.3, 129.1, 128.5, 128.4, 127.3, 113.6, 60.8, 55.4, 32.5, 18.2, 14.0 ppm; IR (film, ATR) $\tilde{\nu}$ = 2982, 2933, 2836, 1719, 1613, 1515, 1447, 1302, 1246, 1211, 1180, 1101, 1033, 837, 769, 699, 551 cm^{-1}; HRMS (ESI+): *m/z* calcd. for $C_{19}H_{21}O_3$ [M+H]: 297.1485; found: 297.1490; The analytical data are consistent with those reported in the literature.[9]

Ethyl *trans*-2-(4-methoxyphenyl)-1-(phenyl)cyclopropane-1-carboxylate (*trans*-15):

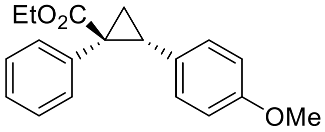

Prepared analogously from *cis*-**2aa** (20 mg, 0.05 mmol) as a yellow oil (9 mg, 58%). After flash chromatography, the material contained traces of dba derived from the palladium precatalyst; an analytically pure sample was ontained by preparative HPLC (YMC-C18 Triart, 5.0 μm, 30 x 150 mm, methanol/water, 70:20, 35 mL·min^{-1}, 308 K, 30 min), which analyzed as follows: $[\alpha]_D^{20}$ = +12.0° (c = 0.9 g·100 mL^{-1}, $CHCl_3$). ^{1}H-NMR (400 MHz, $CDCl_3$): δ = 7.16 – 7.10 (m, 3H), 7.06 – 7.00 (m, 2H), 6.77 – 6.65 (m, 2H), 6.65 – 6.55 (m, 2H), 4.34 – 4.01 (m, 2H), 3.69 (s, 3H), 3.05 (dd, *J* = 9.4, 7.3 Hz, 1H), 2.11 (dd, *J* = 9.4, 4.8 Hz, 1H), 1.80 (dd, *J* = 7.3, 4.9 Hz, 1H), 1.18 (t, *J* = 7.1 Hz, 3H) ppm; ^{13}C{^{1}H}-NMR (100 MHz, $CDCl_3$): 174.0, 158.2, 135.2, 132.1, 129.1, 128.6, 127.7, 127.0, 113.3, 61.3, 55.3, 37.4, 32.6, 20.4, 14.3 ppm; IR (film, ATR) $\tilde{\nu}$ = 2979, 2932, 1710, 1613, 1515, 1447, 1248, 1173, 1159, 1029, 830, 698, 549 cm^{-1}; HRMS (ESI+): *m/z* calcd. for $C_{19}H_{21}O_3$ [M+H]: 297.1485 ; found: 297.1490; The analytical data are consistent with those reported in the literature.[9]

Ethyl *cis*-2-(4-methoxyphenyl)-1-(4-formylphenyl)cyclopropane-1-carboxylate (*cis*-16): Prepared according to the representative procedure from *trans*-**2aa** (20 mg, 0.05 mmol) and 4-iodobenzaldehyde (24 mg, 0.1 mmol) as a yellow oil (15 mg, 89%). $[\alpha]_D^{20}$ = –108.5° (c = 1.0 g·100 mL^{-1}, $CDCl_3$). ^{1}H-NMR (400 MHz, $CDCl_3$): δ = 10.02 (s, 1H), 7.91 – 7.84 (m, 2H), 7.69 – 7.62 (m, 2H), 7.32 – 7.24 (m, 2H), 6.90 – 6.80 (m, 2H), 3.80 (s, 3H), 3.79 (m, 2H), 2.99 – 2.76 (m, 1H), 2.36 (dd, *J* = 7.5, 5.2 Hz, 1H), 1.62 (dd, *J* = 9.1, 5.2 Hz, 1H), 0.85 (t, *J* = 7.1 Hz, 3H) ppm; ^{13}C{^{1}H}-NMR (100 MHz, $CDCl_3$): δ = 192.0, 169.9, 158.8, 147.4, 135.4, 130.9, 130.3, 129.6, 128.0, 113.7, 61.1, 38.1, 32.9, 21.2, 18.5, 14.0 ppm; IR (film, ATR) $\tilde{\nu}$ = 2959, 2930, 2837, 1701, 1606, 1515, 1443, 1303, 1246, 1210, 1179, 1100, 1032, 835, 554 cm^{-1}; HRMS (GC-CI): *m/z* calcd. for $C_{20}H_{21}O_4$ [M+H]: 325.1434; found: 325.1436

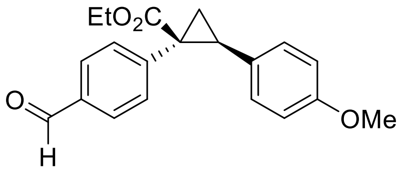

***cis*-17:** Prepared according to the representative procedure from *trans*-**2aa** (20 mg, 0.05 mmol) and 4-iodostyrene (24 mg, 0.1 mmol) as a yellow oil (11 mg, 65%); after flash chromatography, the material contained traces of dba derived from the palladium precatalyst; an analytically pure sample was ontained by preparative HPLC (YMC-C18 Triart, 5.0 μm, 30 x 150 mm, methanol/water, 80:20, 35 mL·min^{-1}, 308 K, 20 min), which analyzed as follows: $[\alpha]_D^{20}$ = –161.7° (c = 1.1 g·100 mL^{-1}, $CDCl_3$). ^{1}H-NMR (400 MHz, $CDCl_3$): δ = 7.49 – 7.37 (m, 4H), 7.33 – 7.22 (m, 2H), 6.94 – 6.82 (m, 2H), 6.73 (dd, *J* = 17.6, 10.8 Hz, 1H), 5.76 (dd, *J* = 17.6, 1.0 Hz, 1H), 5.26 (dd, *J* = 10.8, 1.0 Hz, 1H), 3.85 (m, 1H), 3.80 (s, 3H), 3.77 – 3.62 (m, 1H), 3.11 – 2.58 (m, 1H), 2.29 (dd, *J* = 7.4, 5.0 Hz, 1H), 1.56 (dd, *J* = 9.0, 5.0 Hz, 1H), 0.85 (t, *J* = 7.1 Hz, 3H) ppm; ^{13}C{^{1}H}-NMR (100 MHz, $CDCl_3$): δ = 170.7, 158.6, 140.2, 136.7, 136.4, 130.4, 130.3, 128.7, 126.2, 114.1, 113.6, 60.9, 55.4, 37.9, 32.7, 18.3, 14.0 ppm; IR (film, ATR) $\tilde{\nu}$ = 2979, 2958, 2931, 1718, 1613, 1513, 1447, 1302, 1245, 1210, 1176, 1098, 1031, 987, 836, 767, 698, 531 cm^{-1}; HRMS (ESI+): *m/z* calcd. for $C_{21}H_{23}O_3$[M+H]: 323.1542; found: 323.1647.

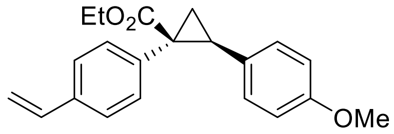

References

1. Lorberth, J., Organometall-substituierte Diazoalkane. II. *J. Organomet. Chem.* **1968,** *15* (1), 251-253.
2. Lorberth, J. r., Metallorganische Diazoalkane III. Diazoalkane mit Germanium, Bblei und den iib-elementen Zink, Cadmium und Quecksilber als Substituenten. *J. Organomet. Chem.* **1971,** *27* (3), 303-325.
3. Schöllkopf, U.; Rieber, N., Triphenylstannyl- and Trimethylsilyl-ethoxycarbonylcarbene from Ethyl Diazo-(triphenylstannyl)- and -(trimethylsilyl)-acetate. **1967,** *6* (10), 884-884.
4. Qin, C.; Davies, H. M. L., Rh2(R-TPCP)4-Catalyzed Enantioselective [3+2]-Cycloaddition between Nitrones and Vinyldiazoacetates. *J. Am. Chem. Soc.* **2013,** *135* (39), 14516-14519.
5. Espino, C. G.; Fiori, K. W.; Kim, M.; Du Bois, J., Expanding the Scope of C−H Amination through Catalyst Design. *J. Am. Chem. Soc.* **2004,** *126* (47), 15378-15379.
6. Carter, S. M.; Sia, A.; Shaw, M. J.; Heyduk, A. F., Isolation and Characterization of a Neutral Imino-semiquinone Radical. *J. Am. Chem. Soc.* **2008,** *130* (18), 5838-5839.
7. Bachmann, S.; Mezzetti, A., Strong Electronic Effects in the cis-Selective Asymmetric Cyclopropanation of Olefins Catalyzed by [RuCl(PNNP)]+. **2001,** *84* (10), 3063-3074.
8. Chepiga, K. M.; Qin, C.; Alford, J. S.; Chennamadhavuni, S.; Gregg, T. M.; Olson, J. P.; Davies, H. M. L., Guide to enantioselective dirhodium(II)-catalyzed cyclopropanation with aryldiazoacetates. *Tetrahedron* **2013,** *69* (27), 5765-5771.
9. Li, Y.; Huang, J.-S.; Zhou, Z.-Y.; Che, C.-M.; You, X.-Z., Remarkably Stable Iron Porphyrins Bearing Nonheteroatom-Stabilized Carbene or (Alkoxycarbonyl)carbenes: Isolation, X-ray Crystal Structures, and Carbon Atom Transfer Reactions with Hydrocarbons. *J. Am. Chem. Soc.* **2002,** *124* (44), 13185-13193.

Ethyl (trimethylstannyl)diazoacetate (1a): ^{1}H-NMR (400 MHz, CD_2Cl_2)

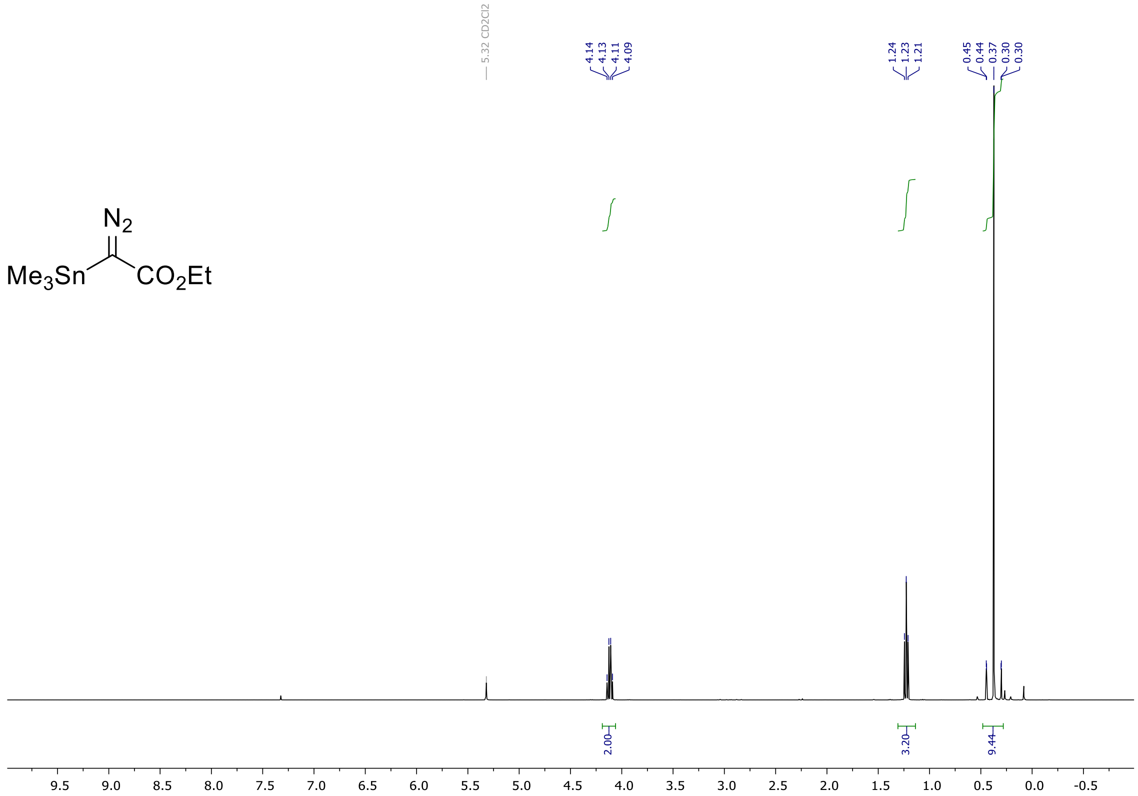

^{13}C{^{1}H}-NMR (100 MHz CD_2Cl_2)

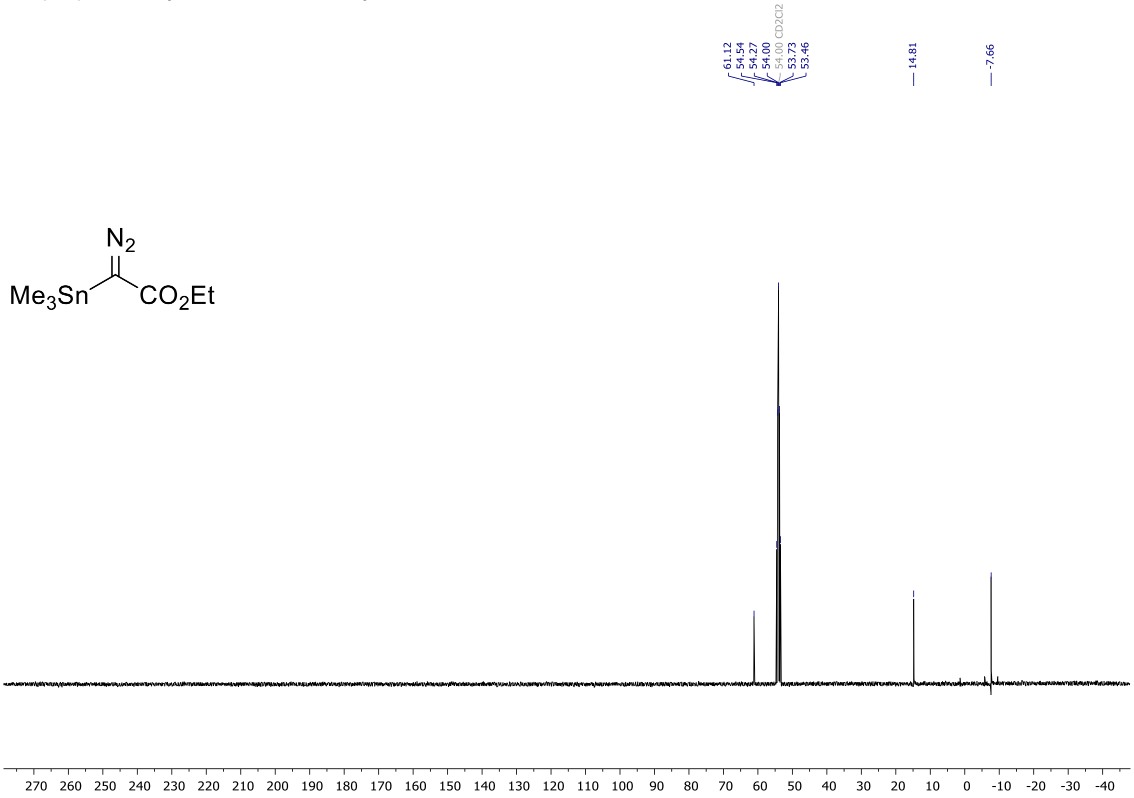

$^{119}Sn\{^{1}H\}$-NMR (149 MHz, CD_2Cl_2)

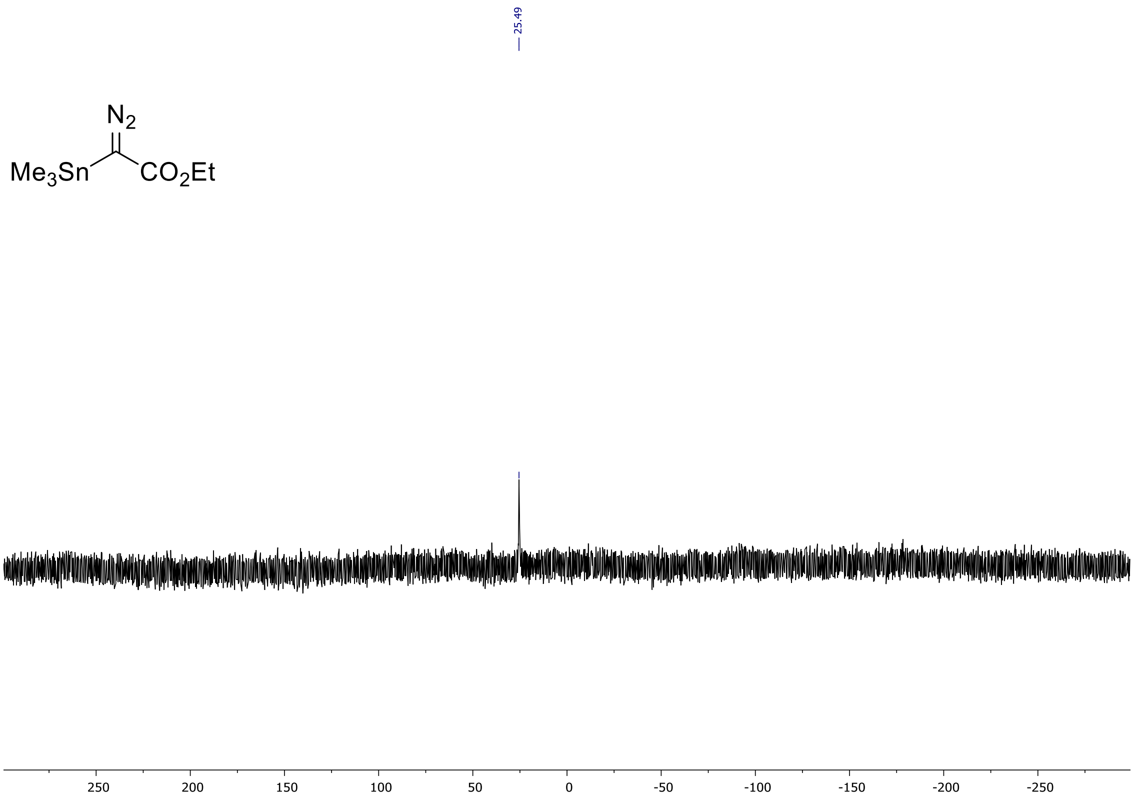

^{1}H-^{13}C-HMBC (CD_2Cl_2)

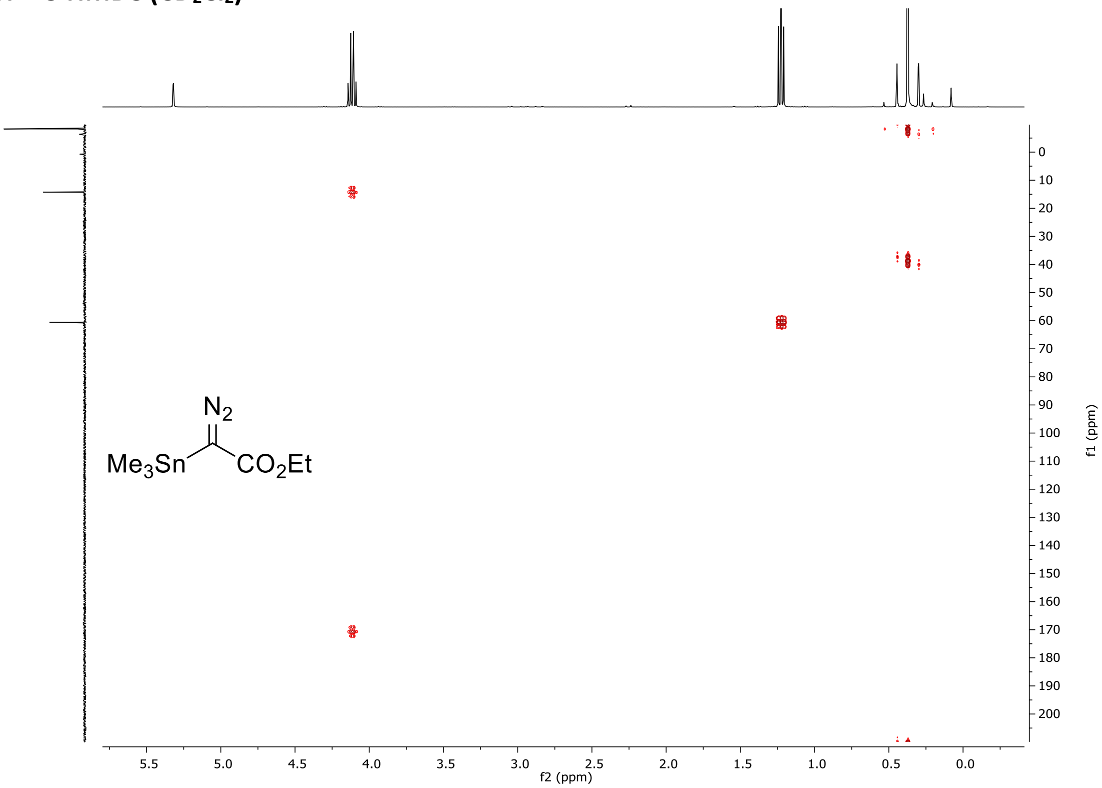

Ethyl (trimethylgermanyl)diazoacetate (1b): ^{1}H-NMR (400 MHz, CD_2Cl_2)

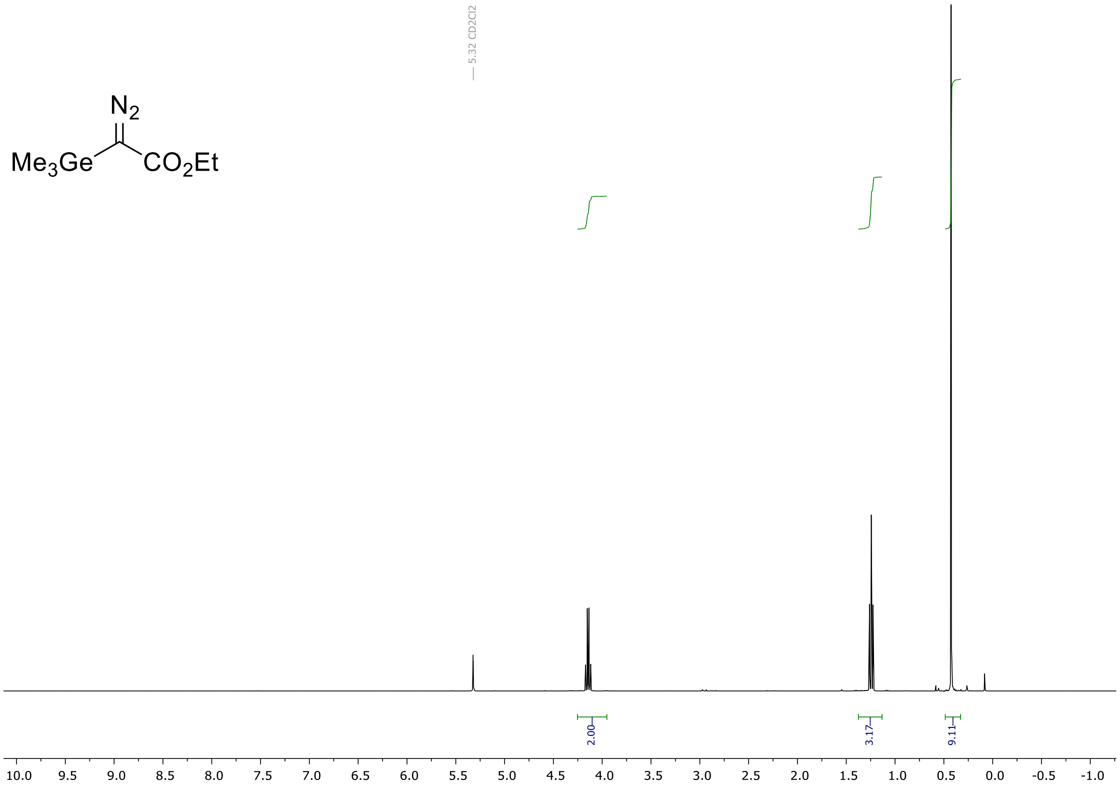

^{13}C-NMR (100 MHz, CD_2Cl_2)

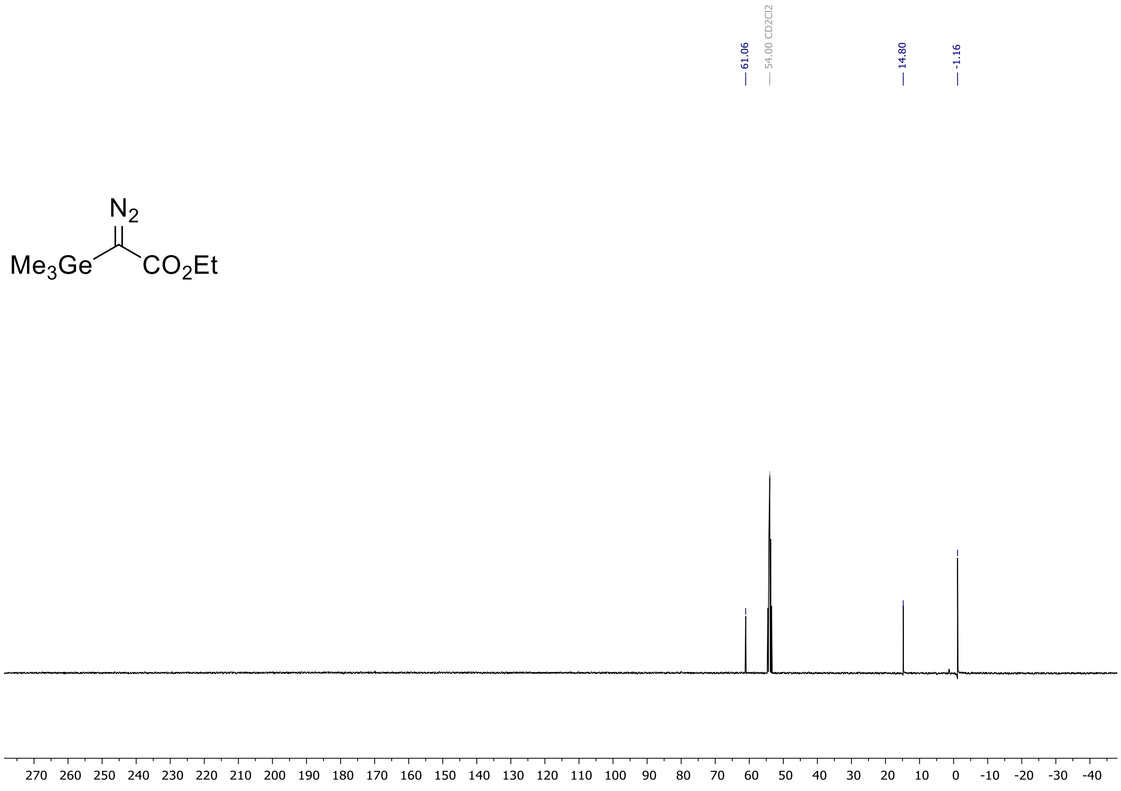

^1H-^{13}C-HMBC (CD_2Cl_2)

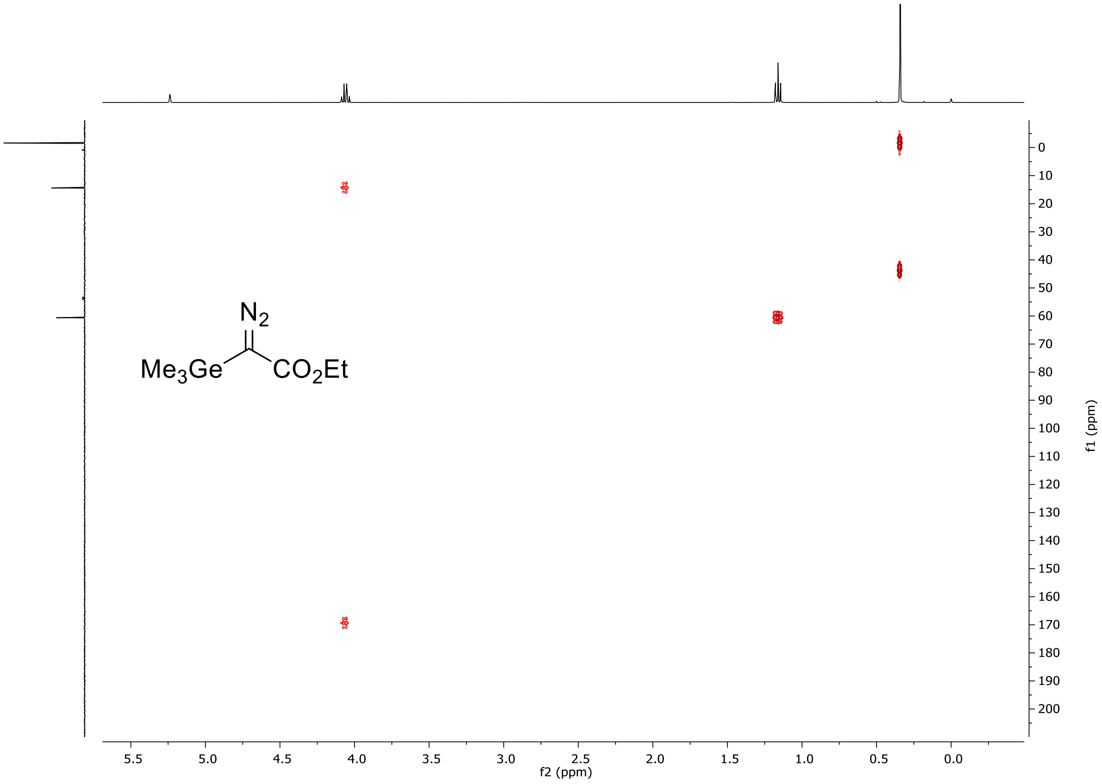

Ethyl (trimethylsilyl)diazoacetate (1c): ^{1}H-NMR (400 MHz, $CDCl_3$)

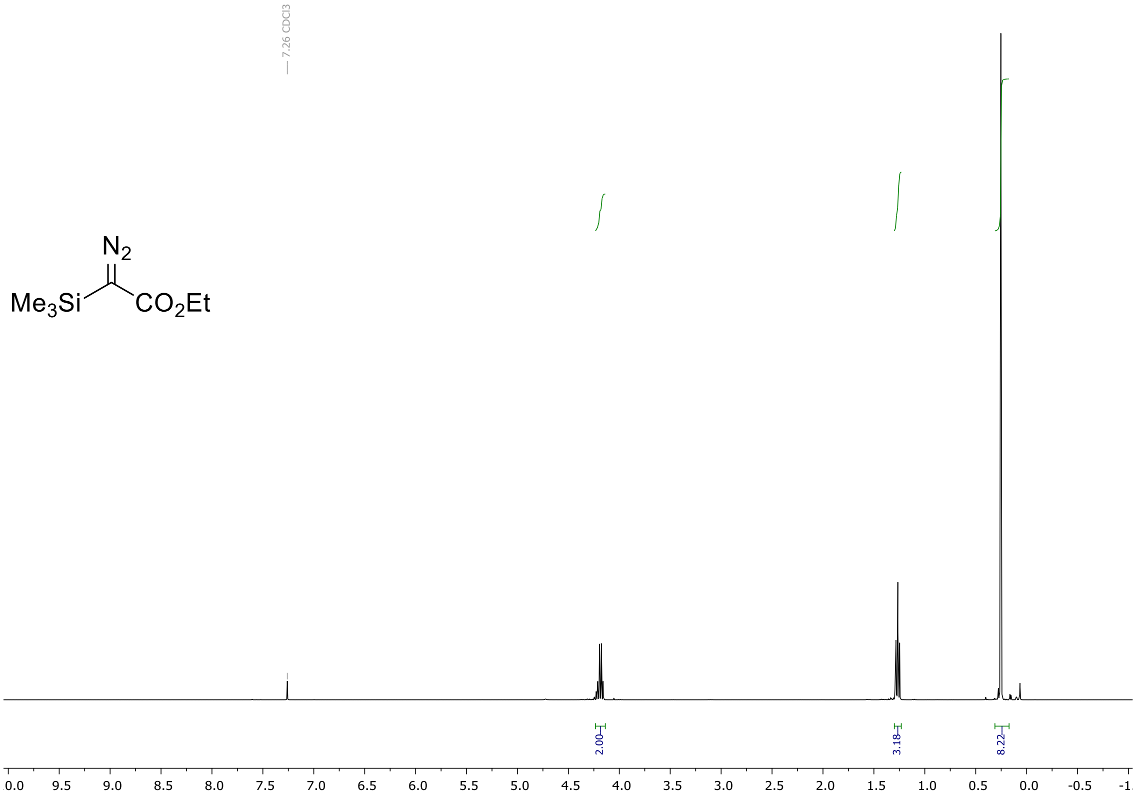

^{13}C-NMR (100 MHz, $CDCl_3$)

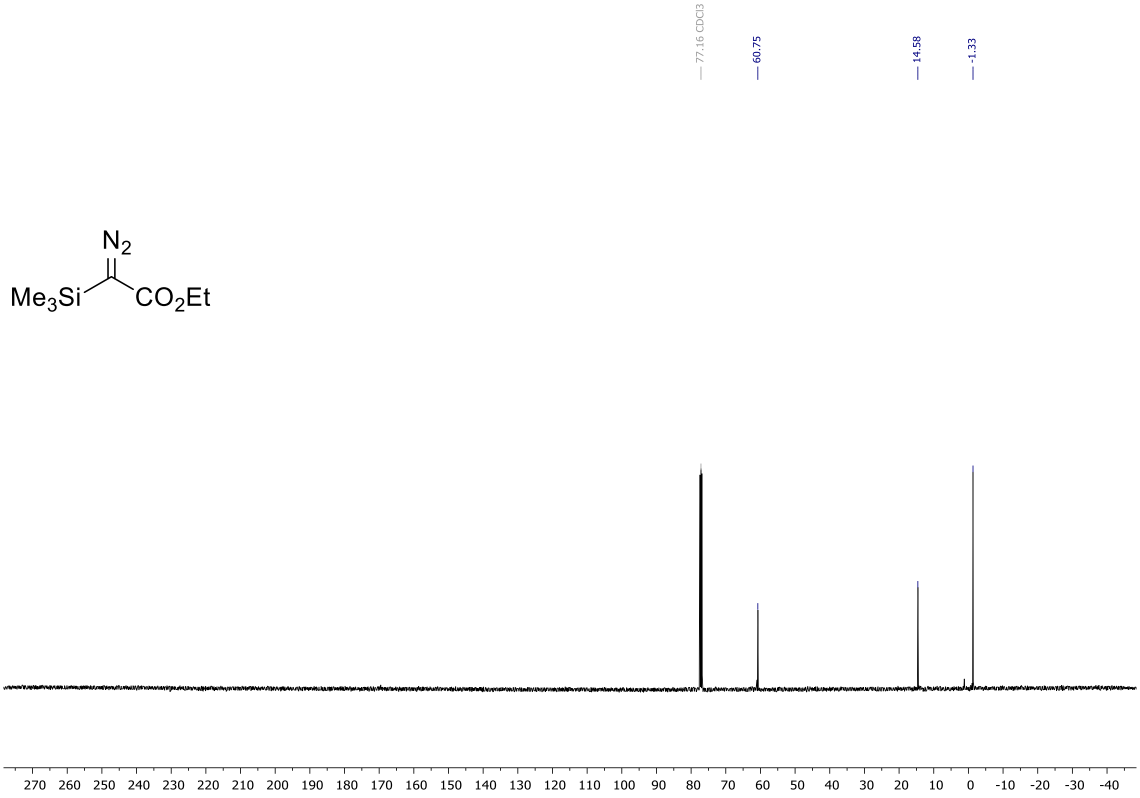

^{1}H-^{13}C-HMBC ($CDCl_3$)

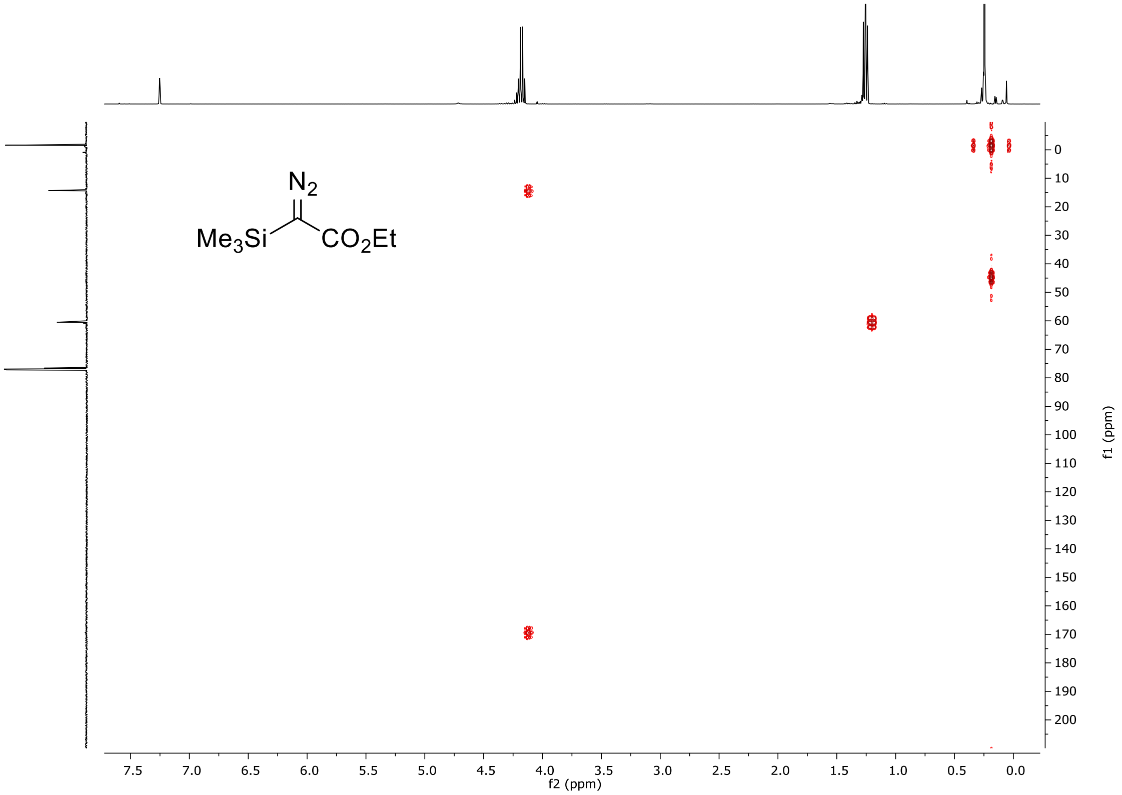

Rh_2(acam)(*(R)*-TPCP)$_3$ (7): ^{1}H-NMR (400 MHz, CD_3CN)

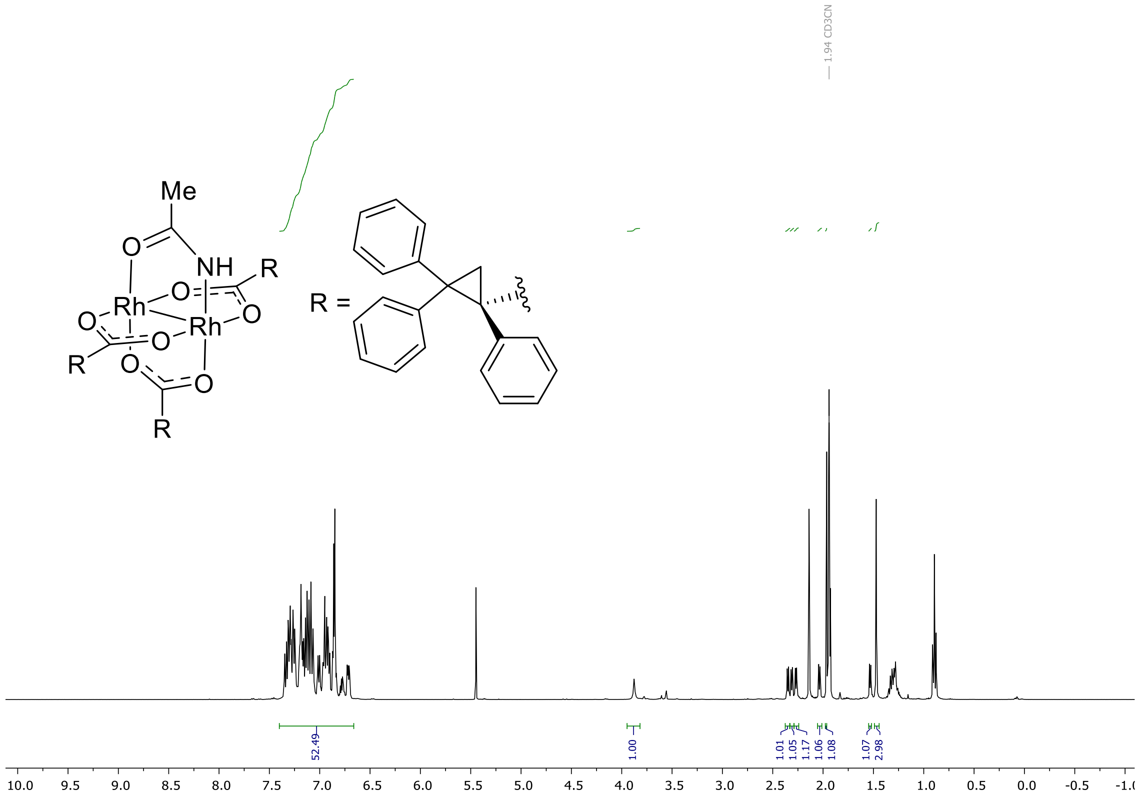

^{13}C-NMR (100 MHz, CD_3CN)

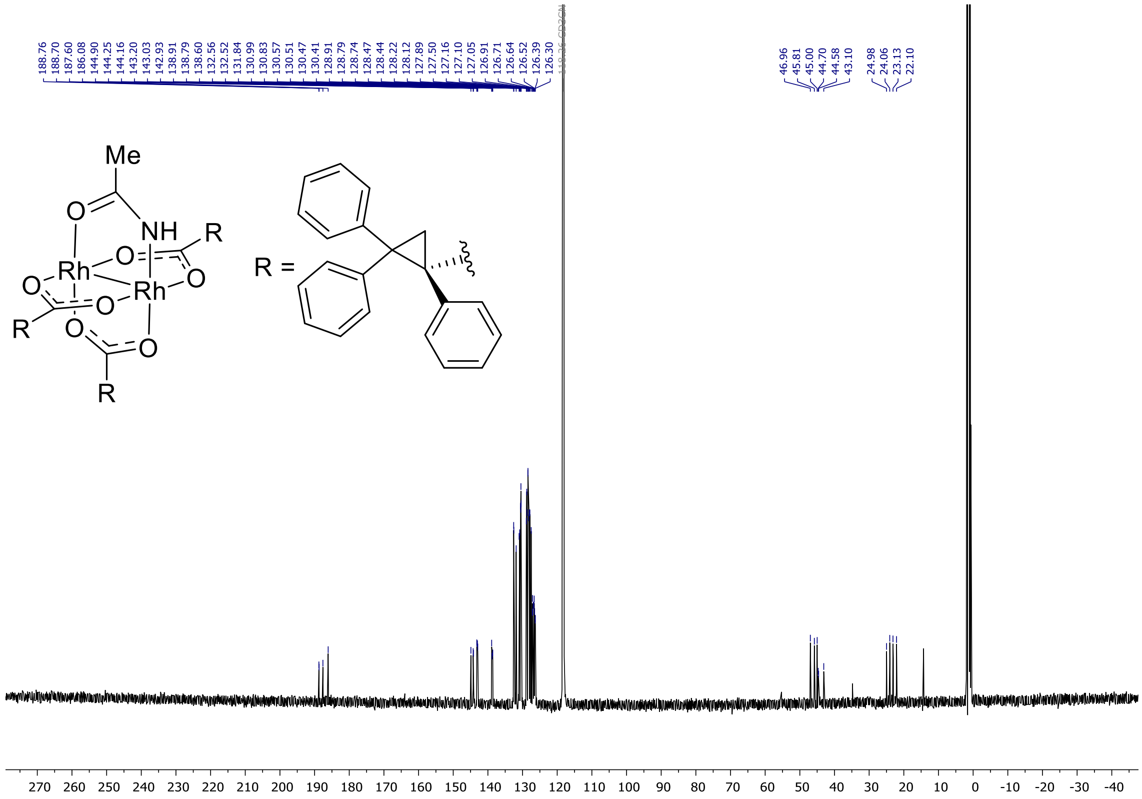

^{1}H-^{13}C-HMBC (CD_3CN)

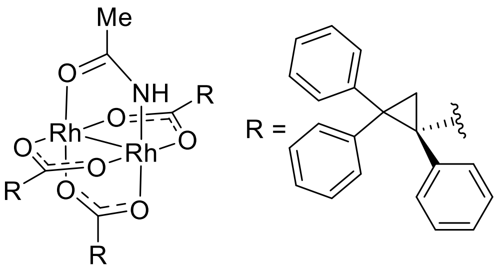

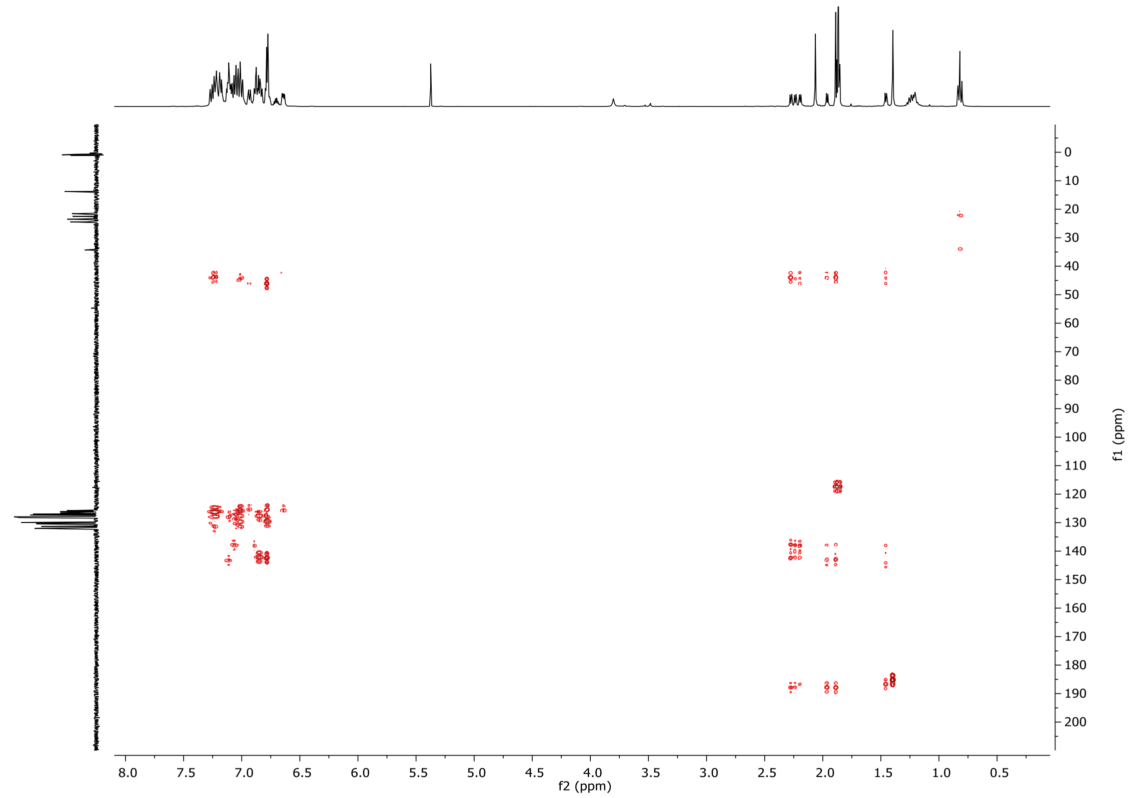

$Rh_2(OAc)((R)\text{-}TPCP)_3$ (8): ^{1}H-NMR (400 MHz, CD_3CN)

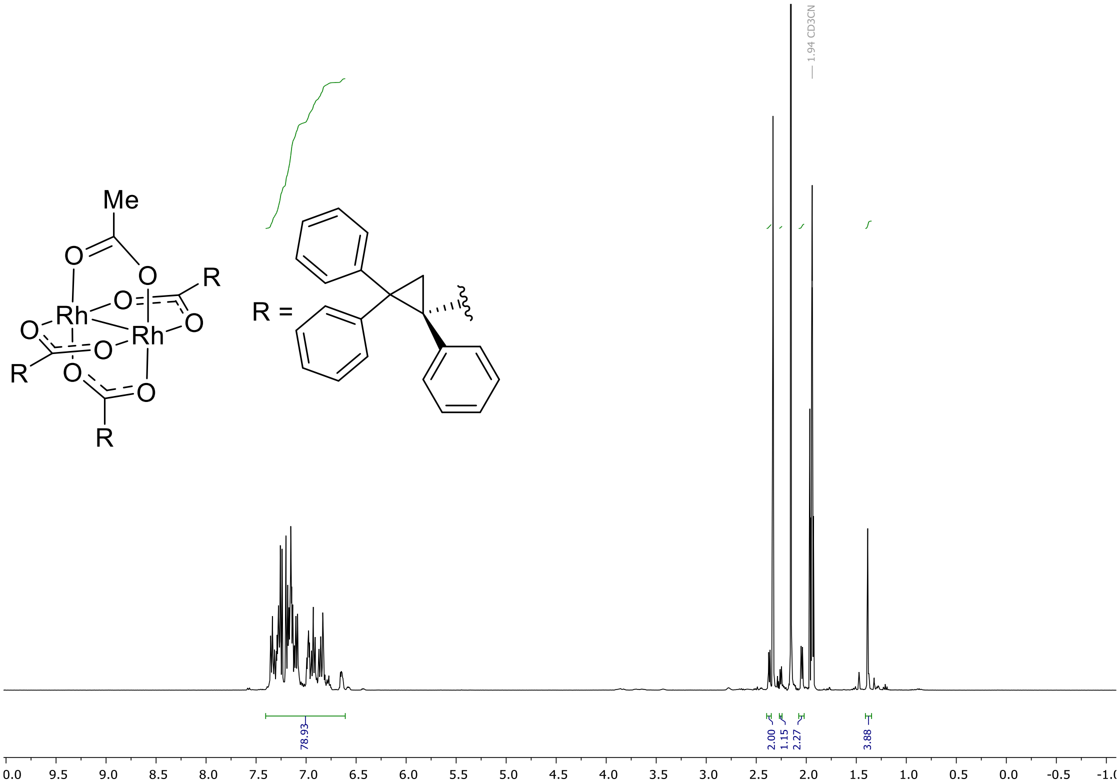

^{13}C-NMR (100 MHz, CD_3CN)

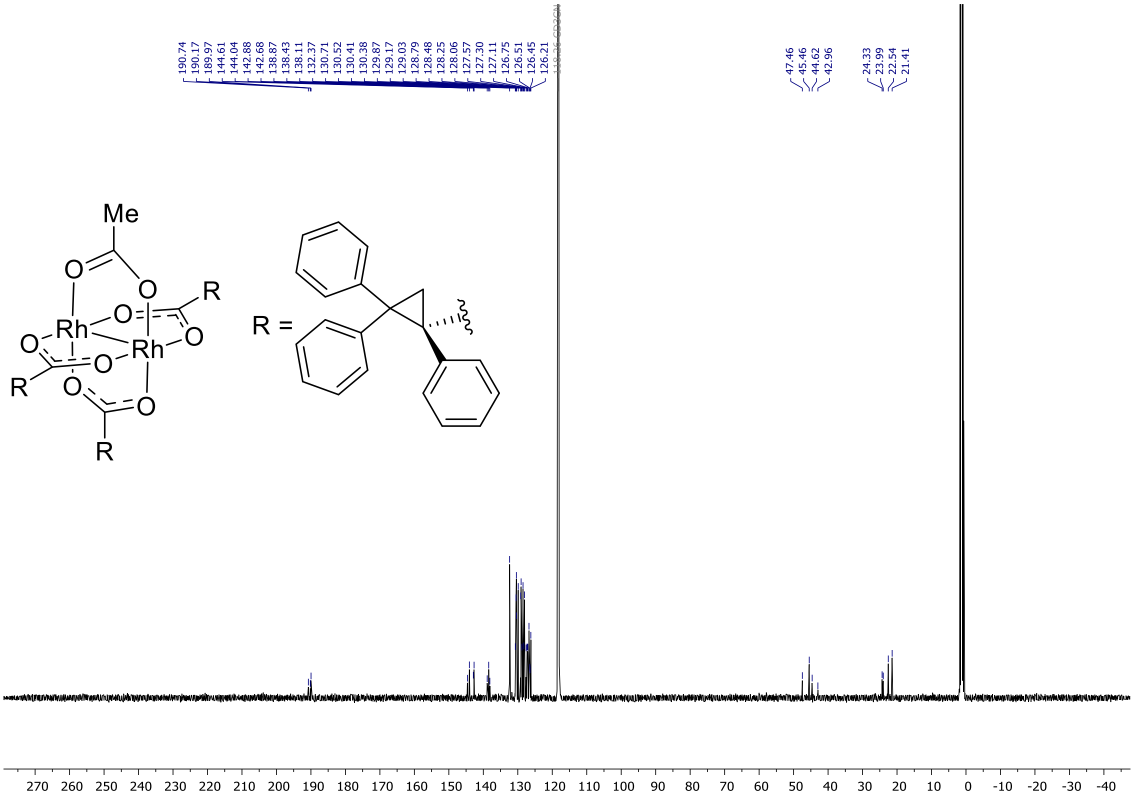

Rh_2(TFA)(*(R)*-TPCP)$_3$ (10): ^{1}H-NMR (400 MHz, CD_3CN)

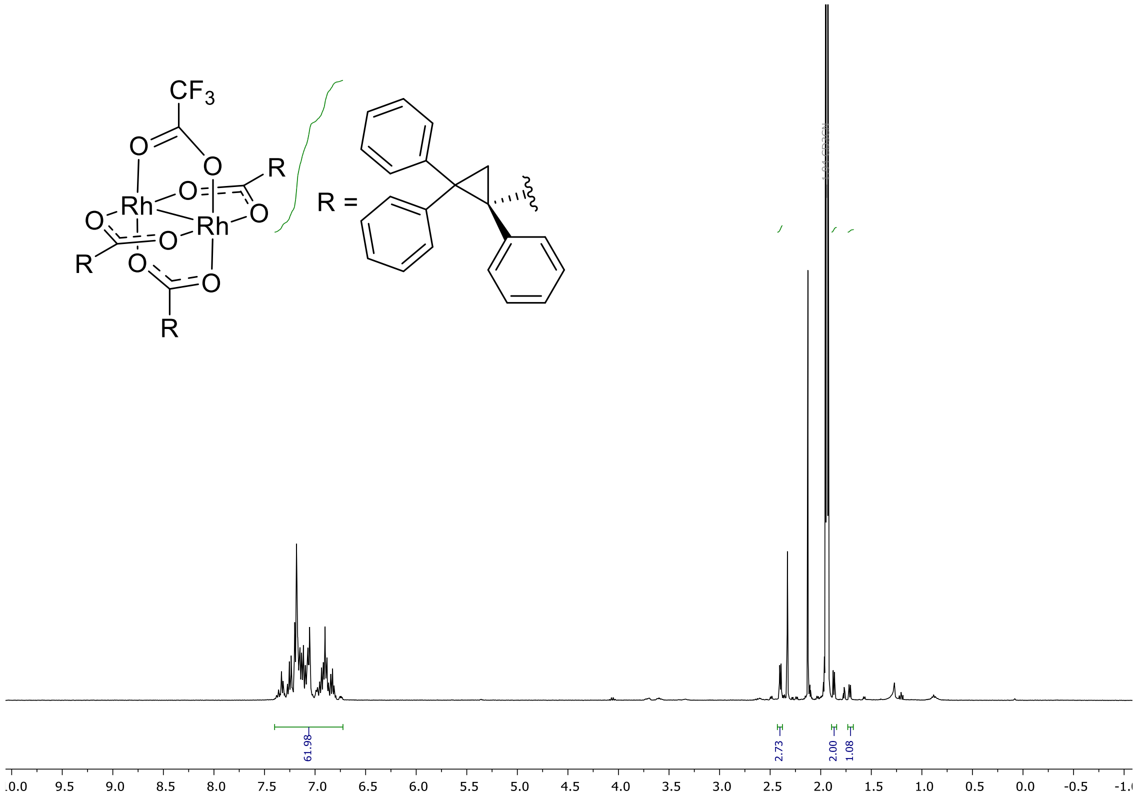

^{13}C-NMR (100 MHz, CD_3CN)

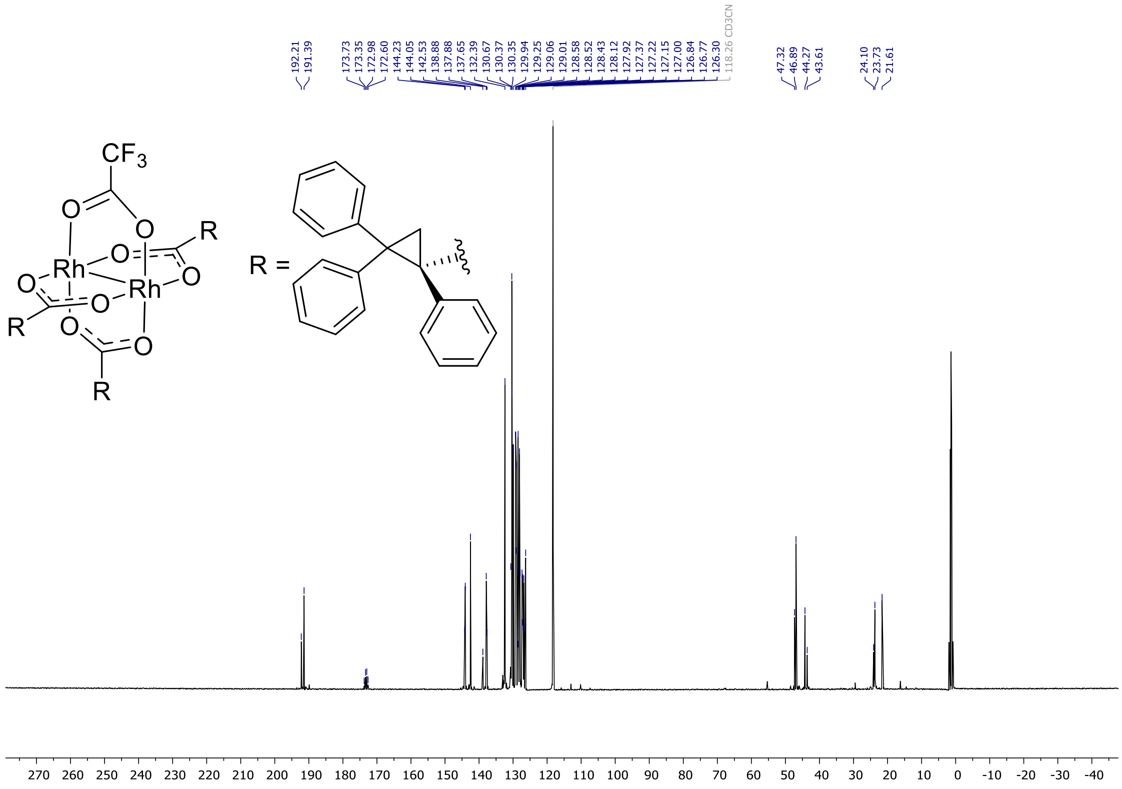

^{19}F-NMR (470 MHz, CD_3CN)

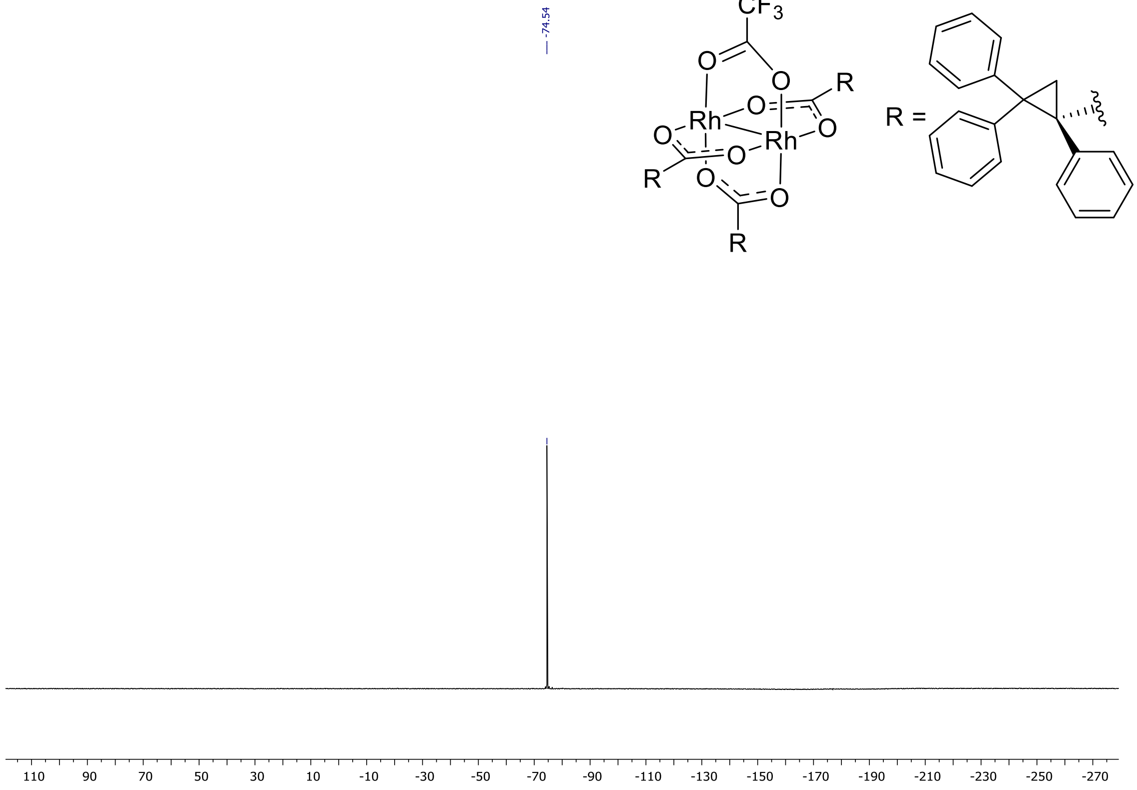

^{1}H-NMR (600 MHz, CD_3CN)

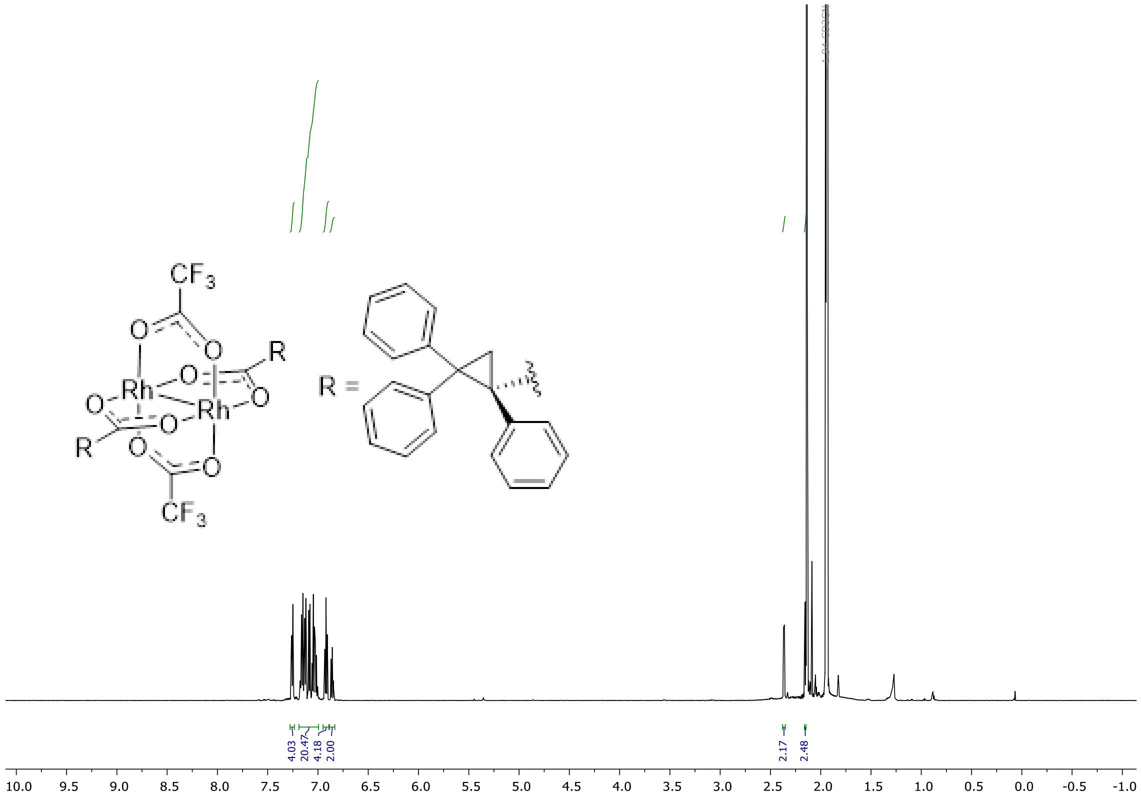

^{13}C-NMR (151 MHz, CD_3CN)

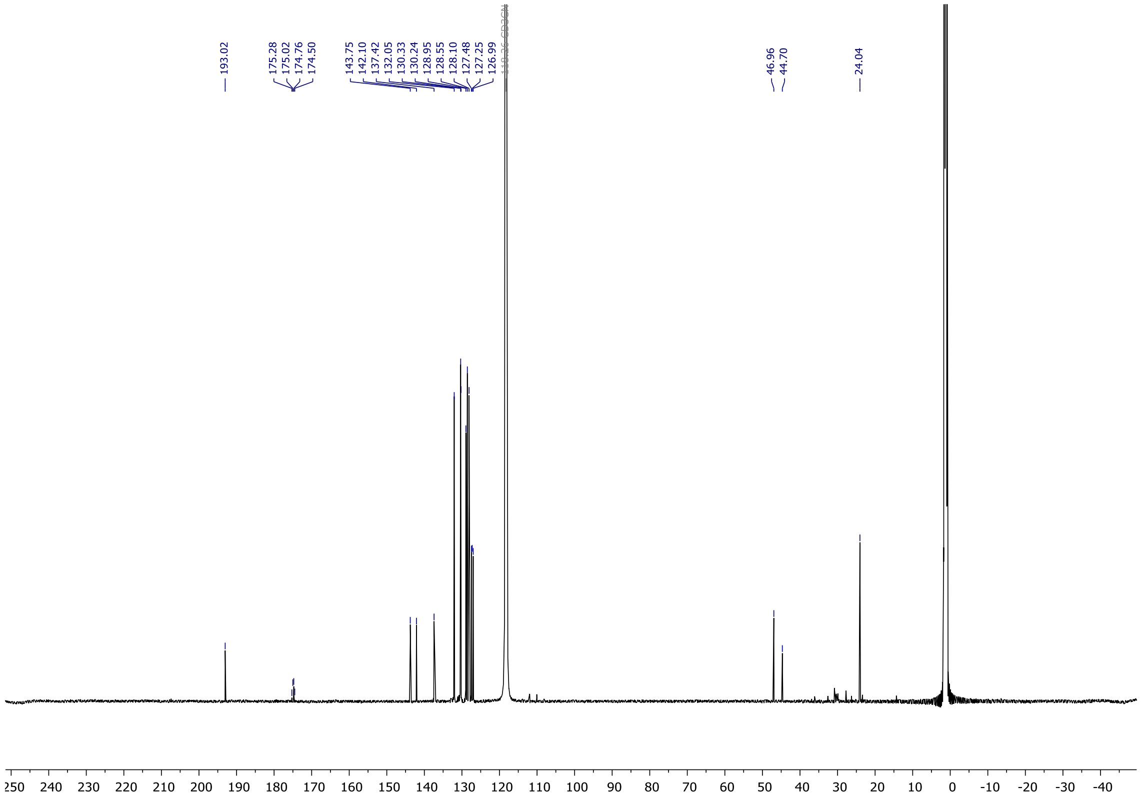

^{19}F-NMR (282 MHz, CD_3CN)

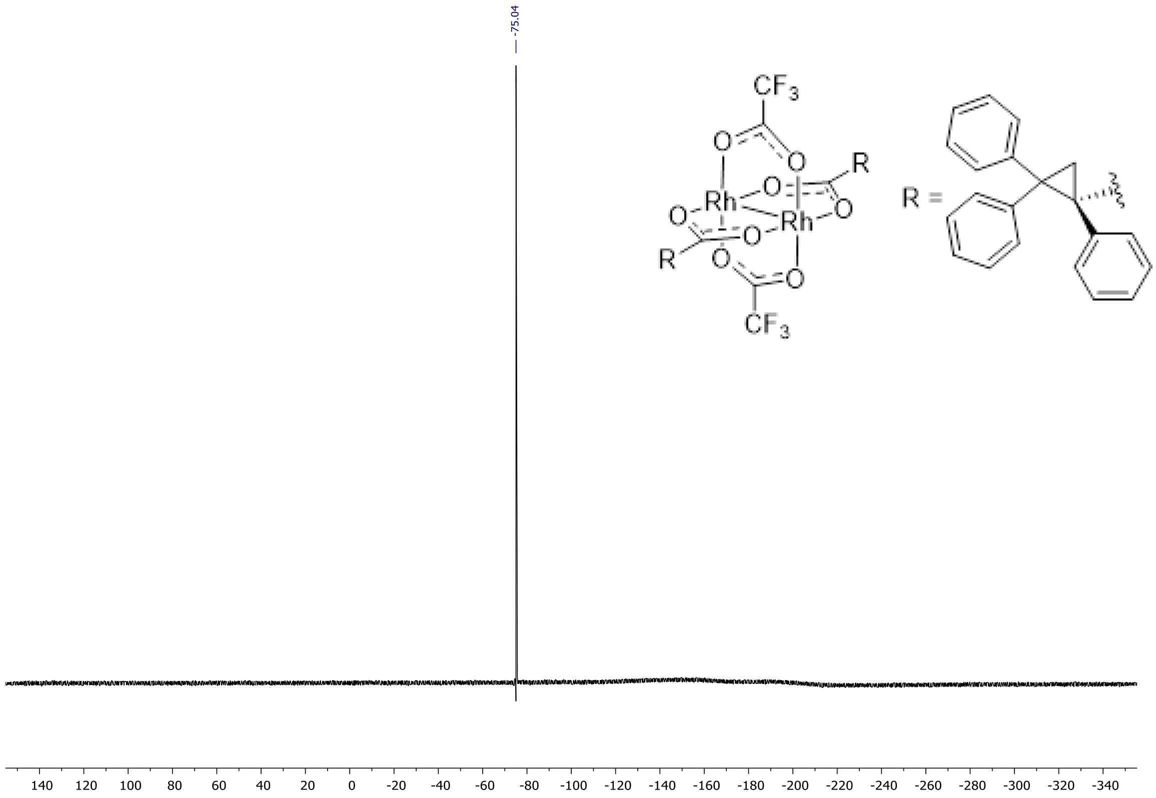

Rh$_2$(trifluoroacetamidate)(*(R)*-TPCP)$_3$ (11): ^{1}H-NMR (600 MHz, CD$_3$CN)

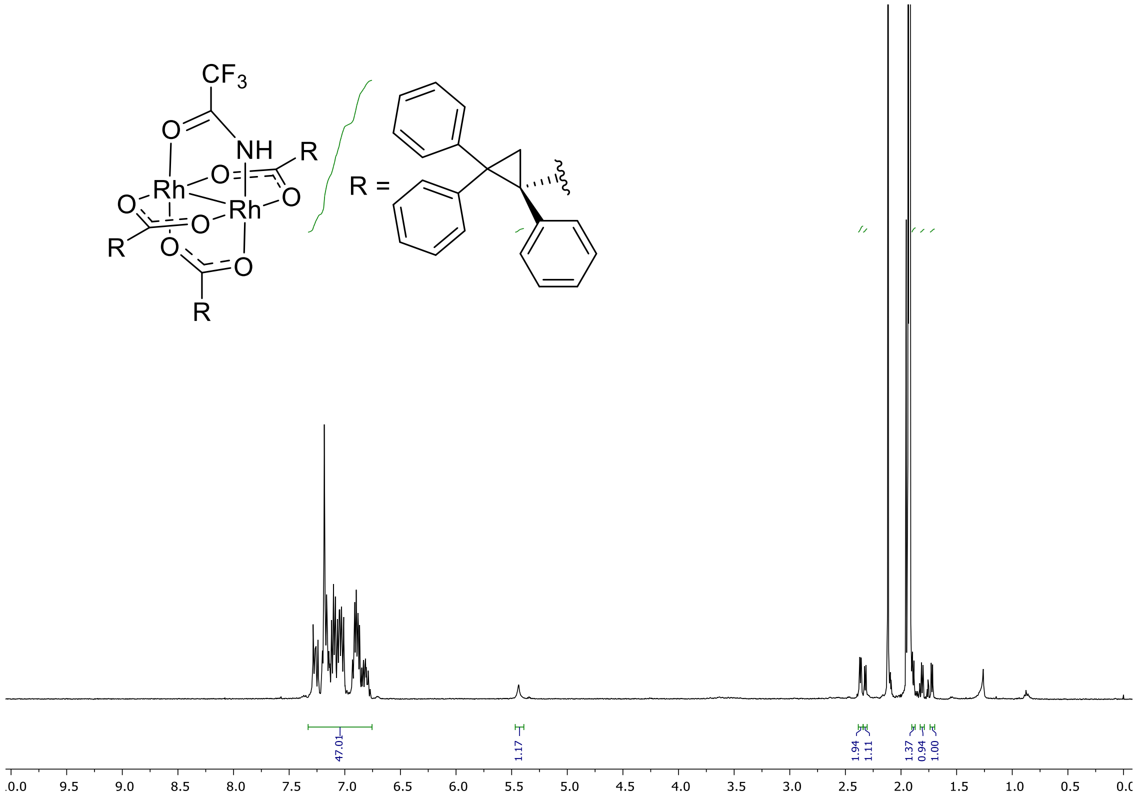

^{13}C{^{1}H}-NMR including ^{13}C{^{19}F}- NMR (470MHz, CD$_3$CN)

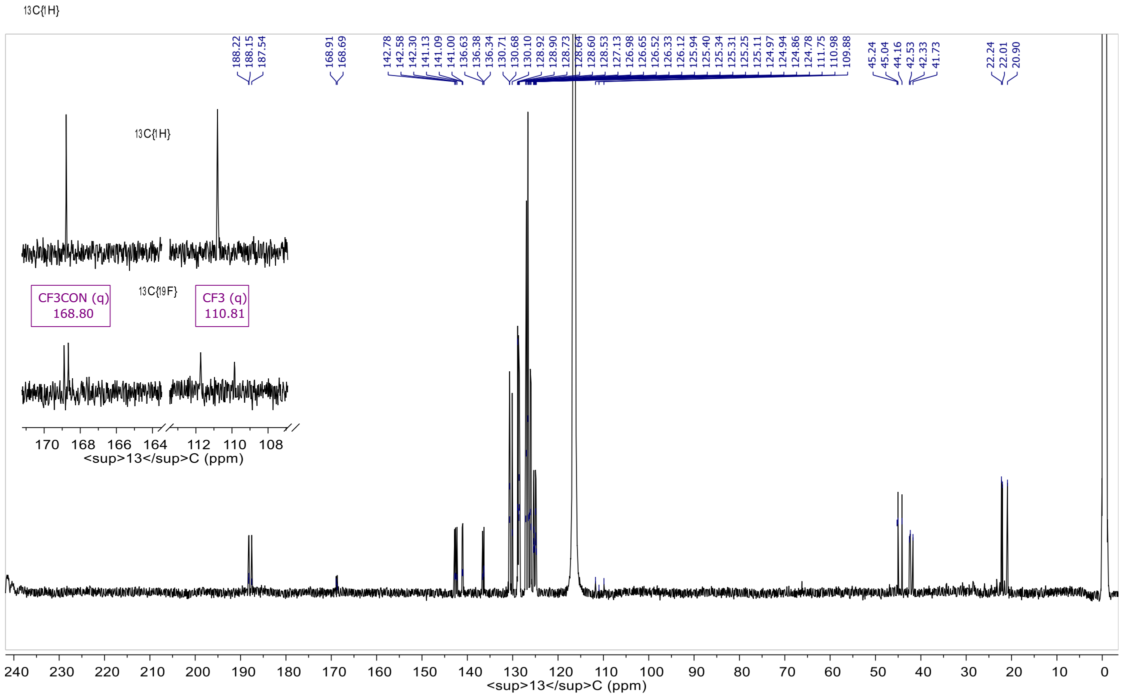

$^{19}F\{^1H\}$-NMR (565 MHz, CD_3CN)

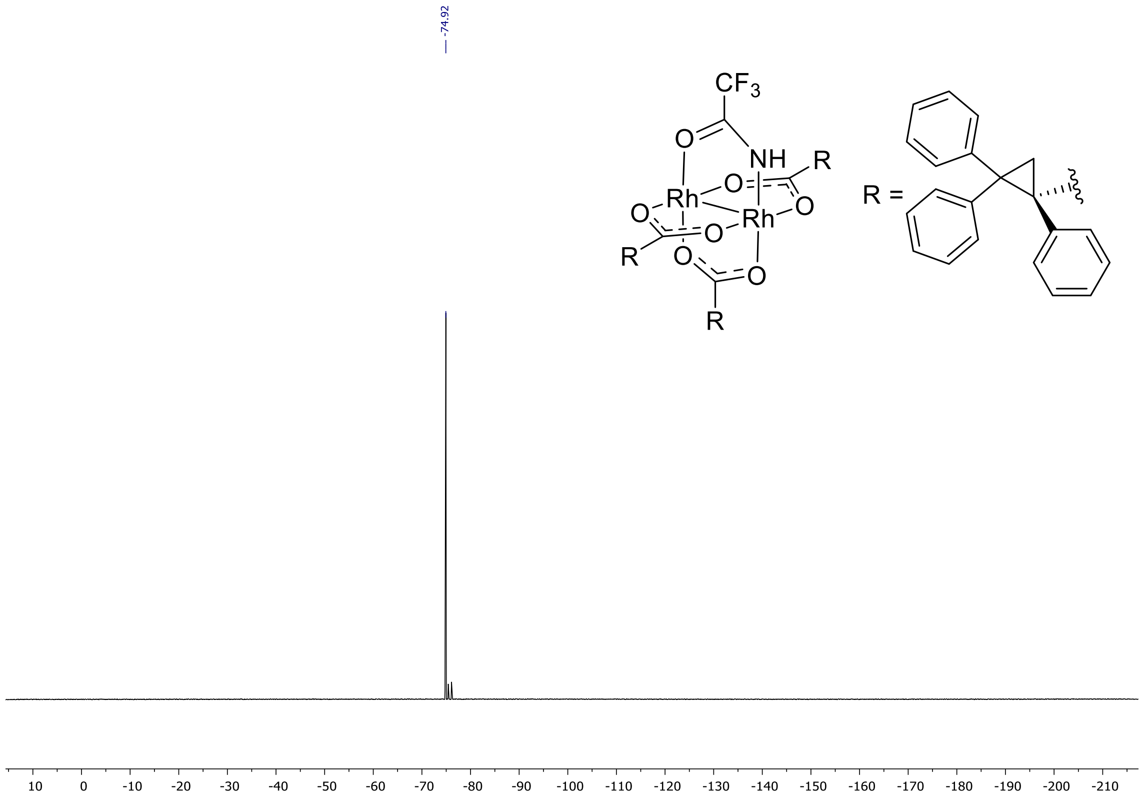

Rh$_2$(N-methylacam)(*(R)*-TPCP)$_3$ (12): ^{1}H-NMR (600 MHz, CD$_3$CN)

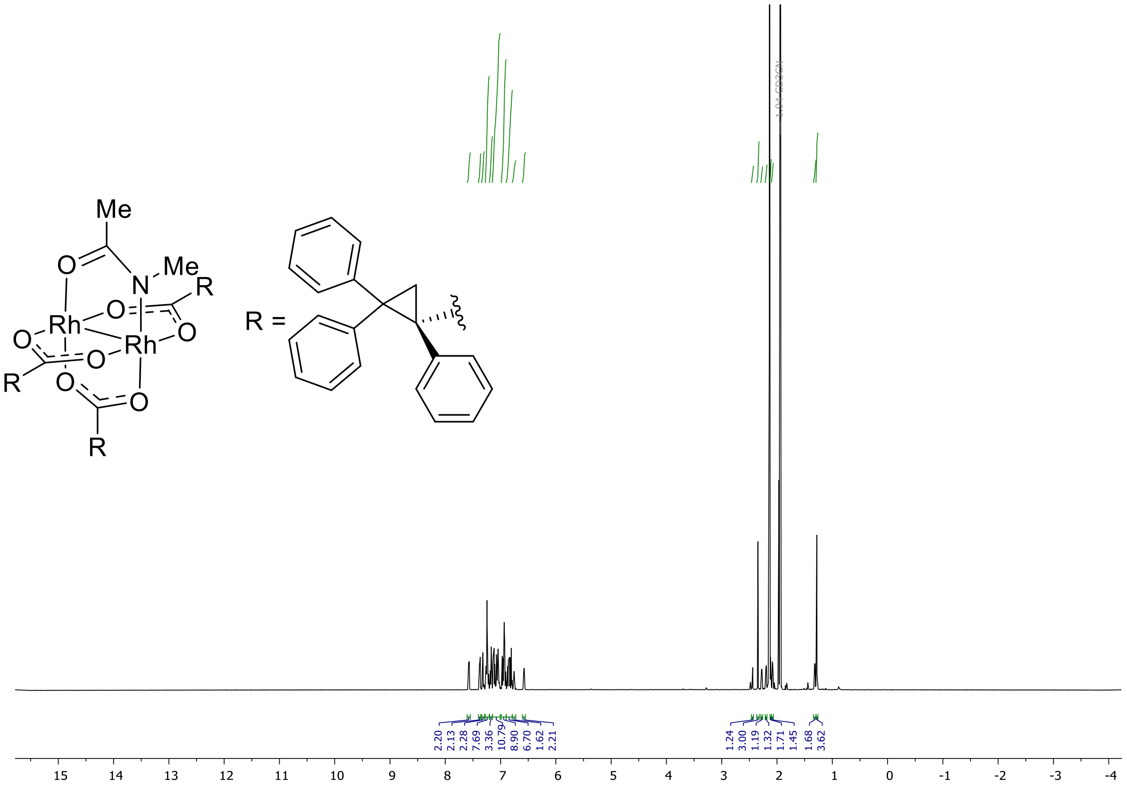

^{13}C-NMR (151 MHz, CD$_3$CN)

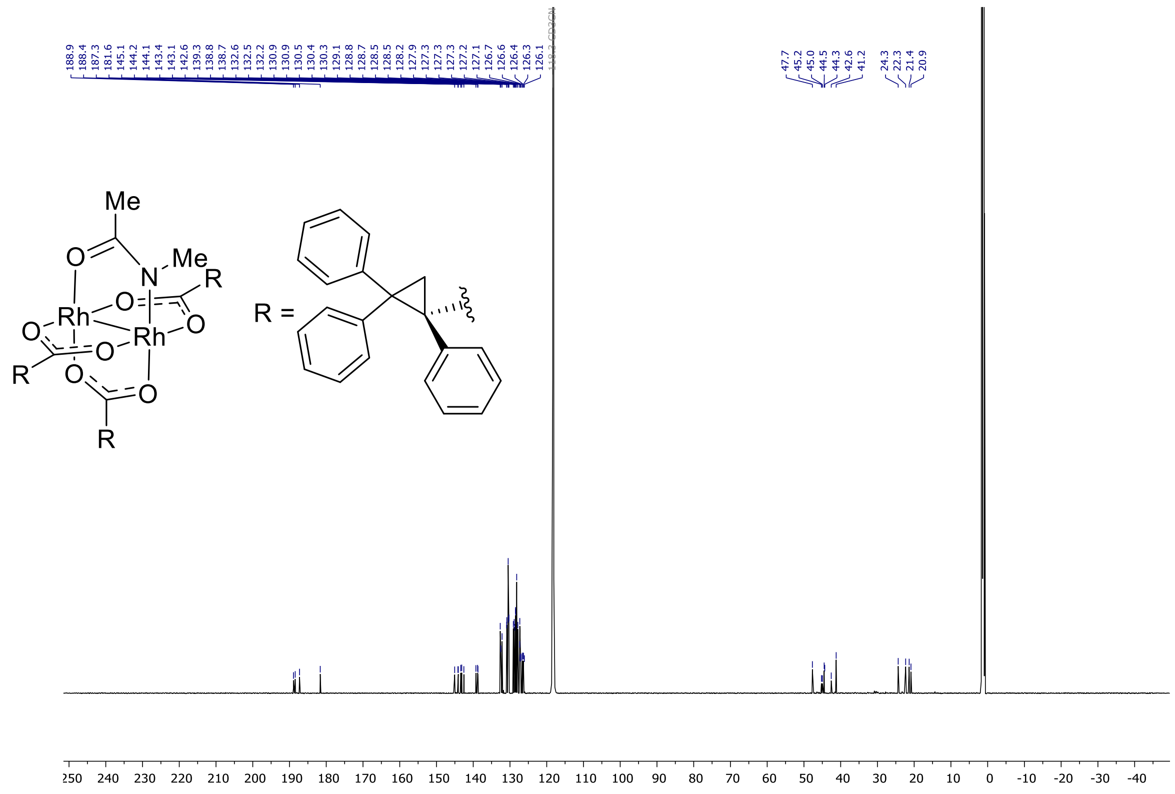

cis-2aa: ^{1}H-NMR (600 MHz, $CDCl_3$)

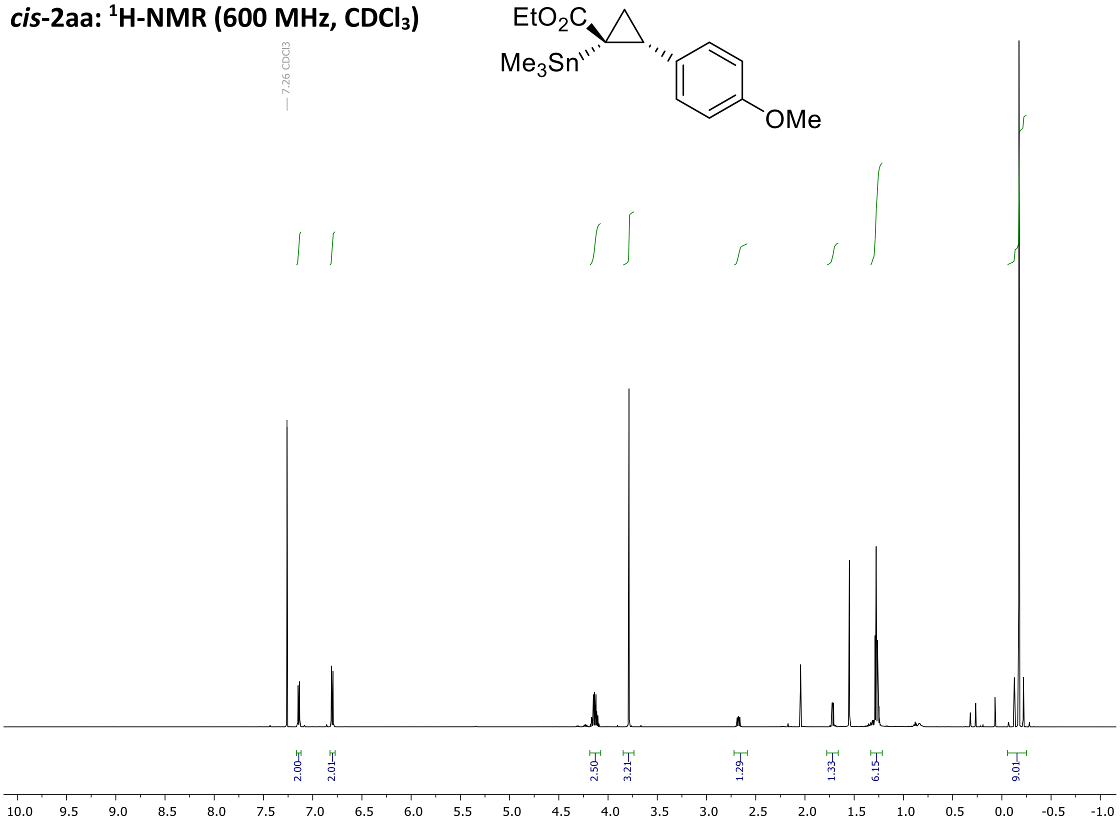

^{13}C-NMR (100 MHz, $CDCl_3$)

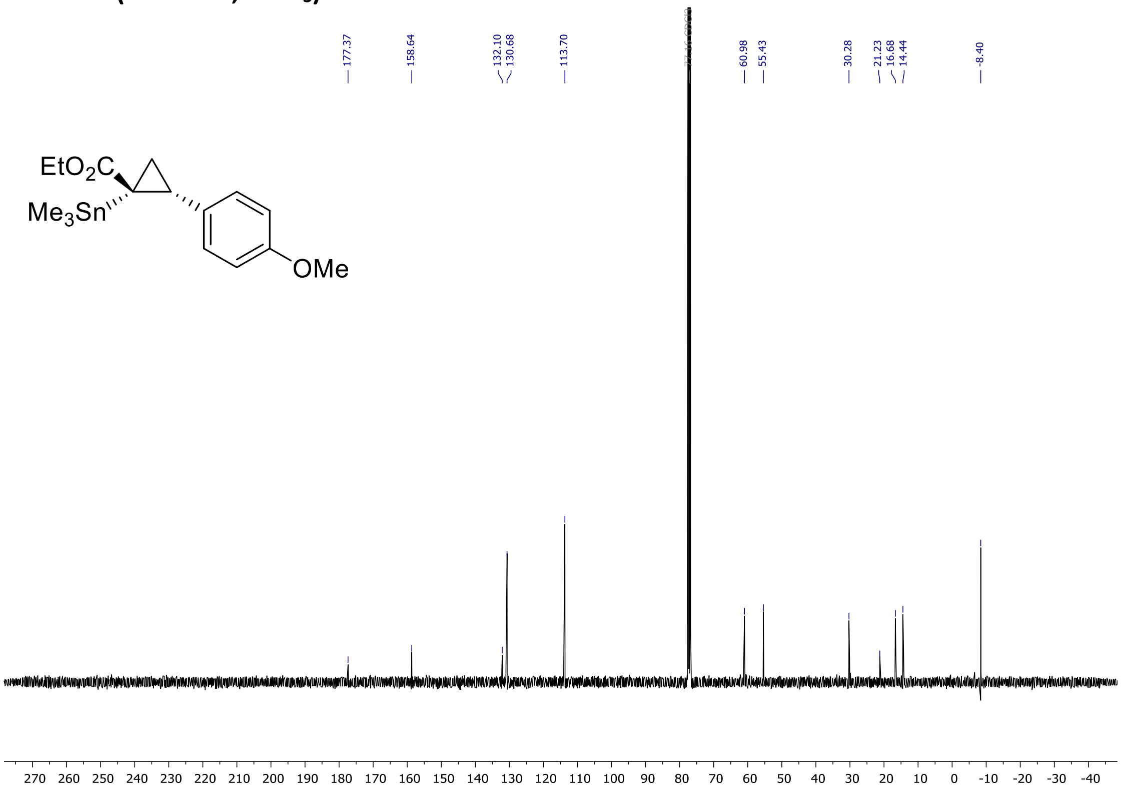

^{119}Sn-NMR (149 MHz, $CDCl_3$)

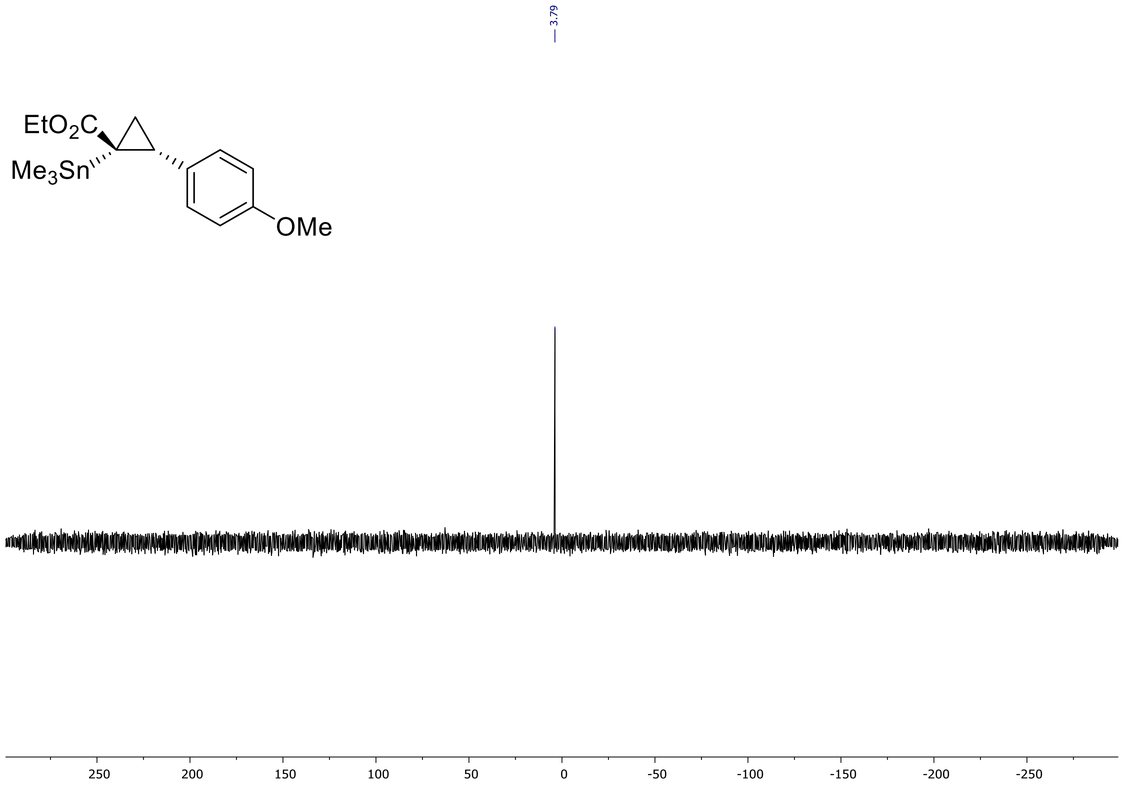

^{1}H-^{1}H-NOESY ($CDCl_3$)

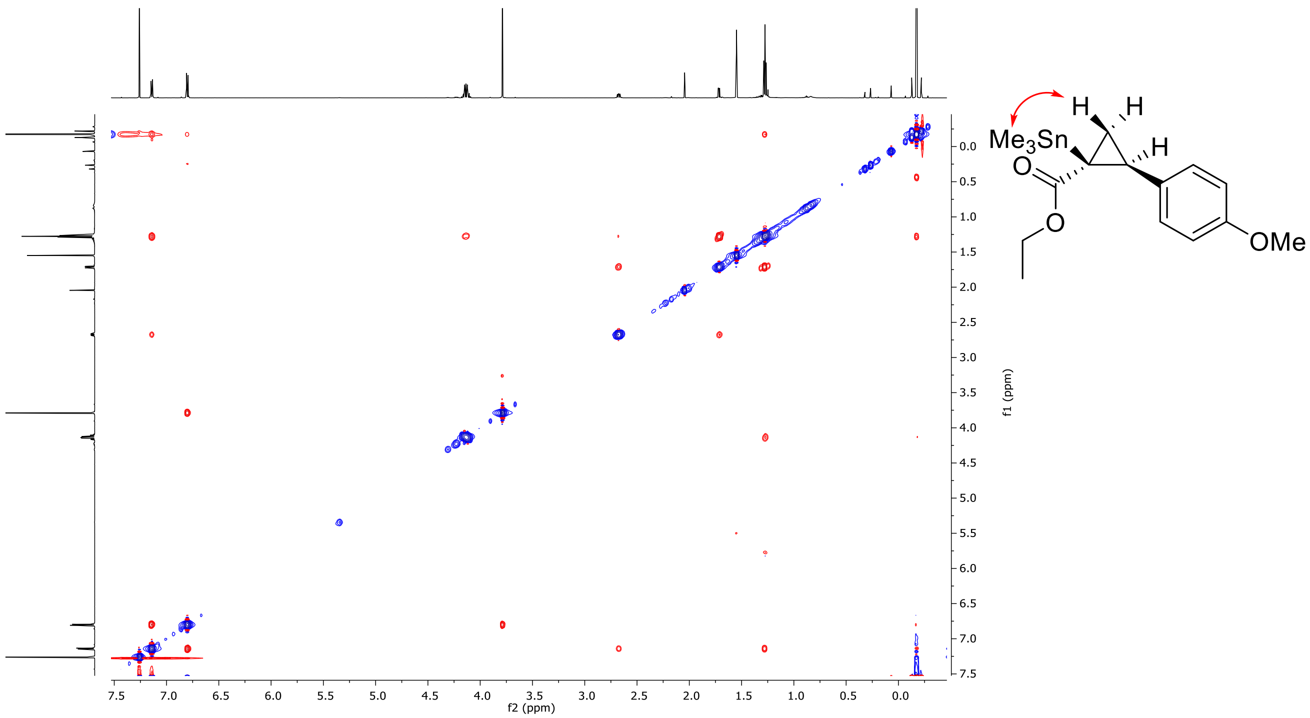

trans-2aa: ^{1}H-NMR (400 MHz, $CDCl_3$)

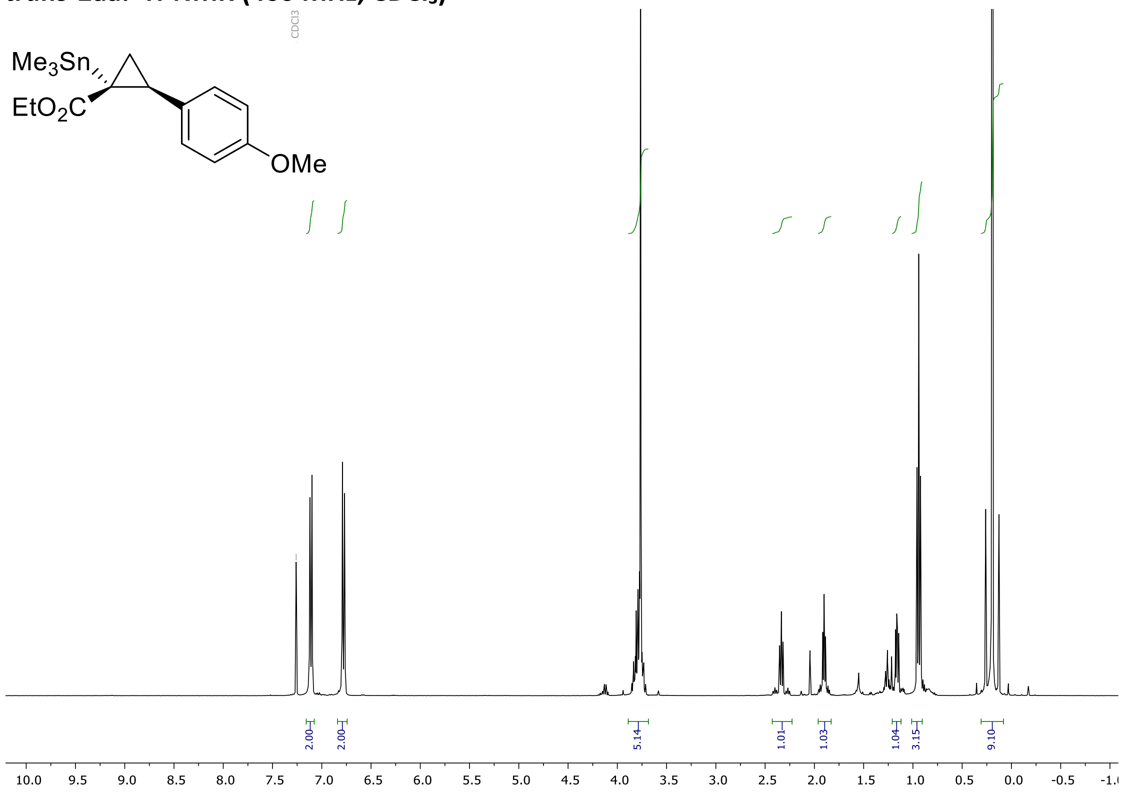

^{13}C-NMR (100 MHz, $CDCl_3$)

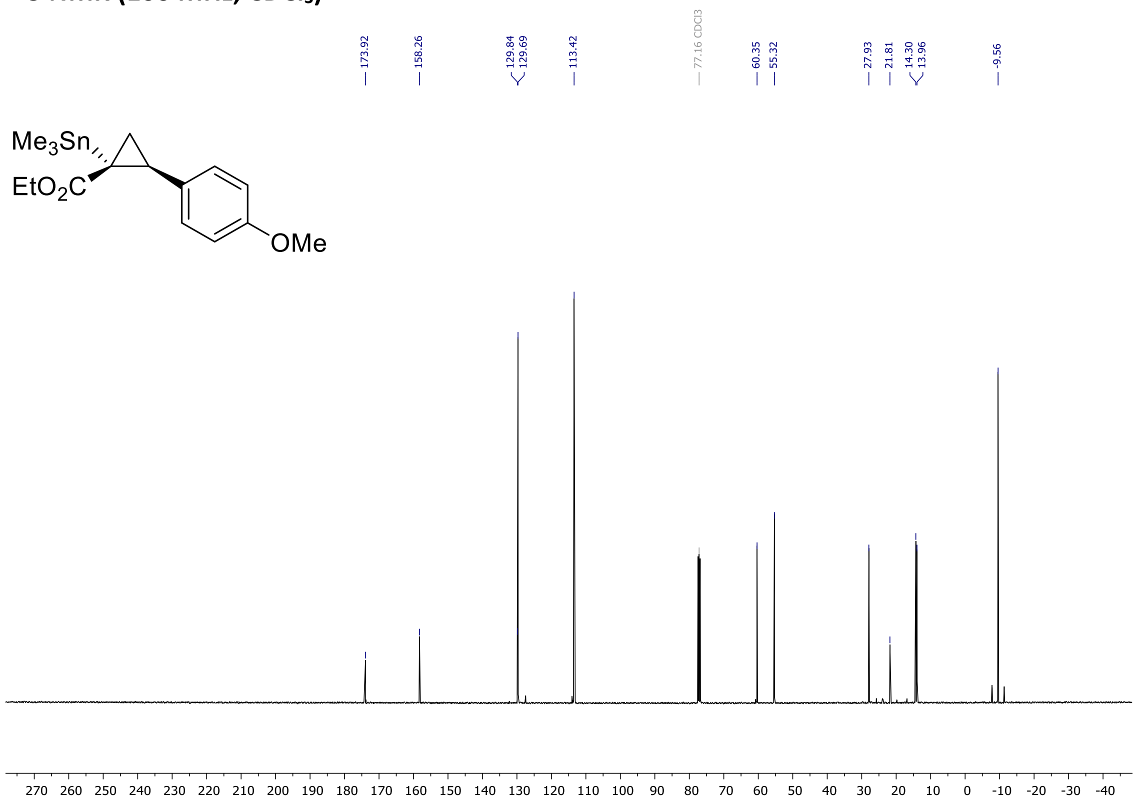

^{119}Sn-NMR (149 MHz, $CDCl_3$)

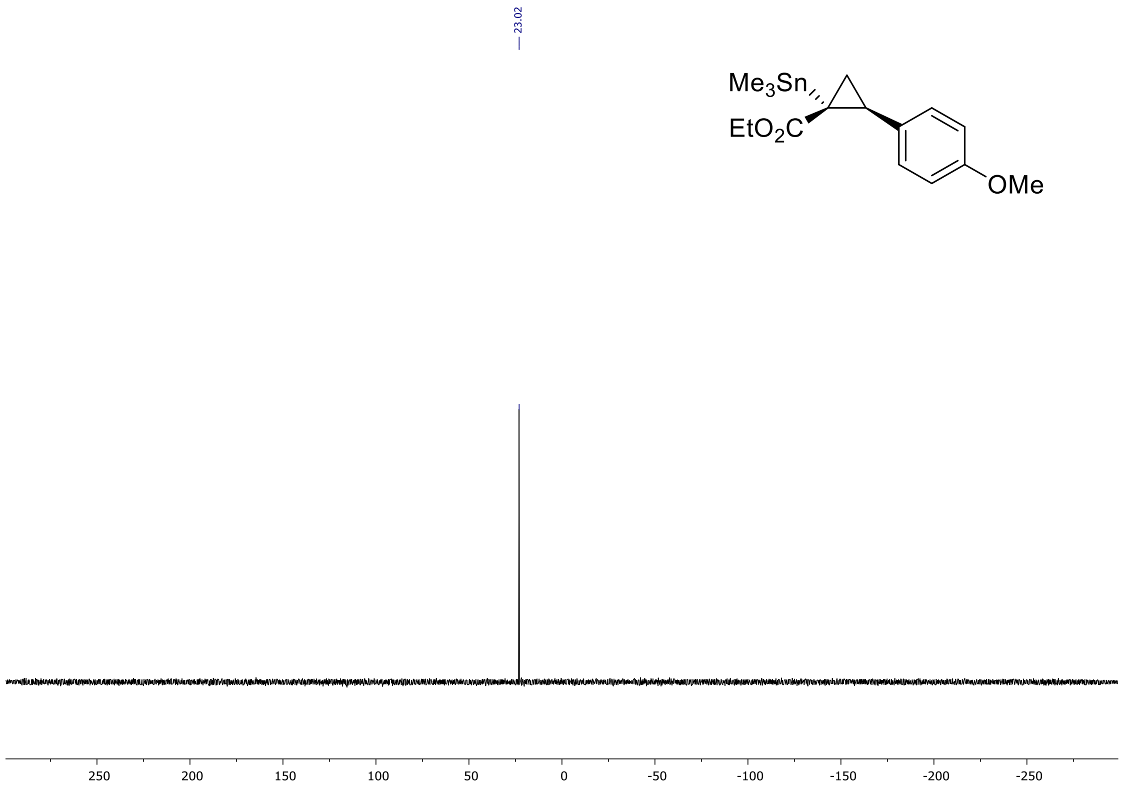

^{1}H-^{1}H-NOESY ($CDCl_3$)

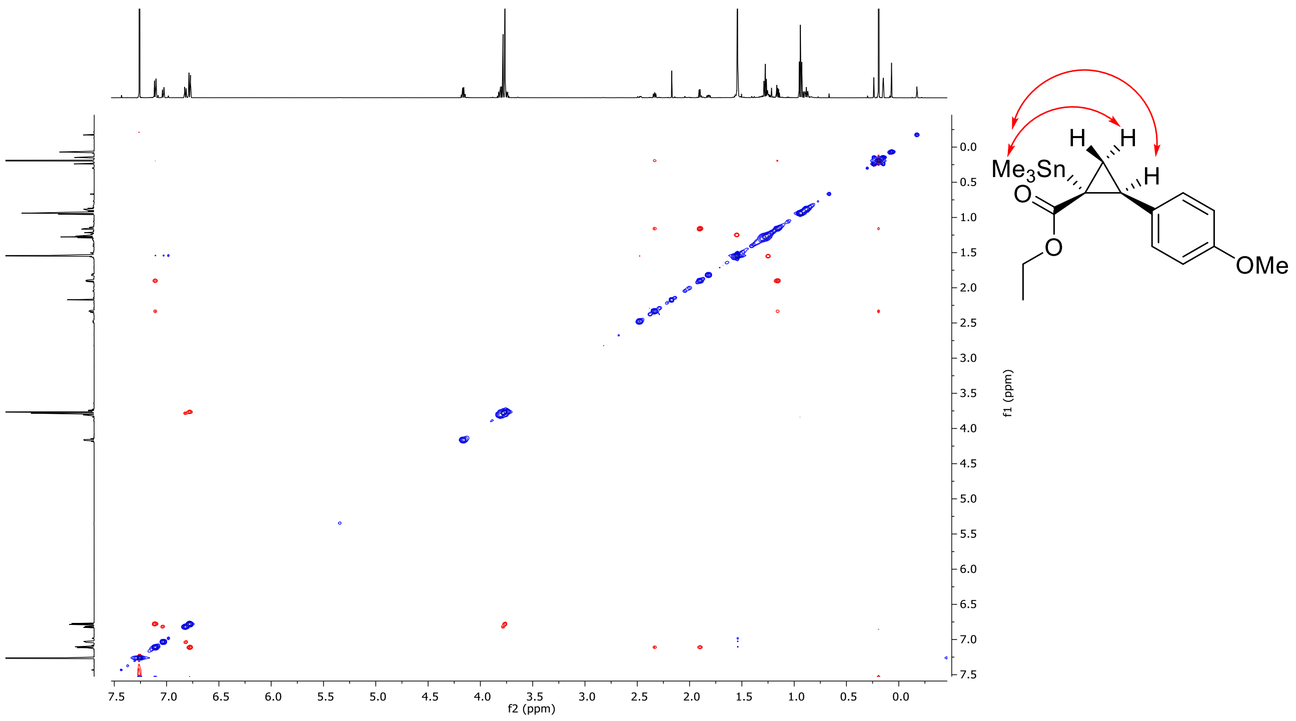

***cis*-2ba: ^{1}H-NMR (500 MHz, $CDCl_3$)**

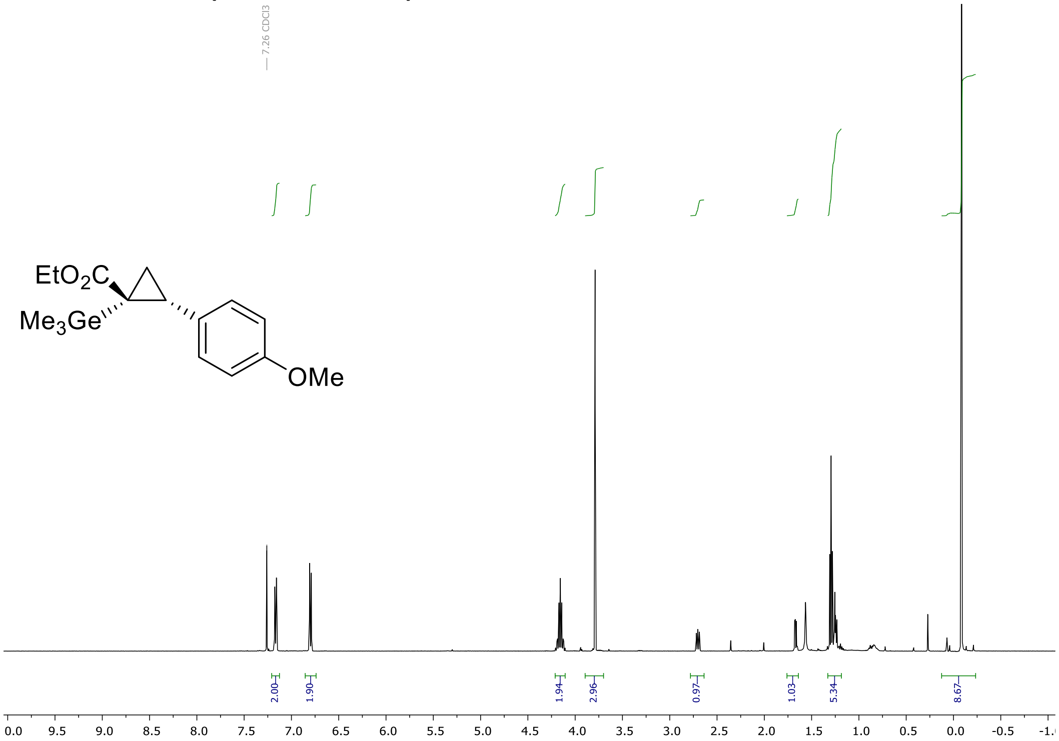

^{13}C-NMR (100 MHz, $CDCl_3$)

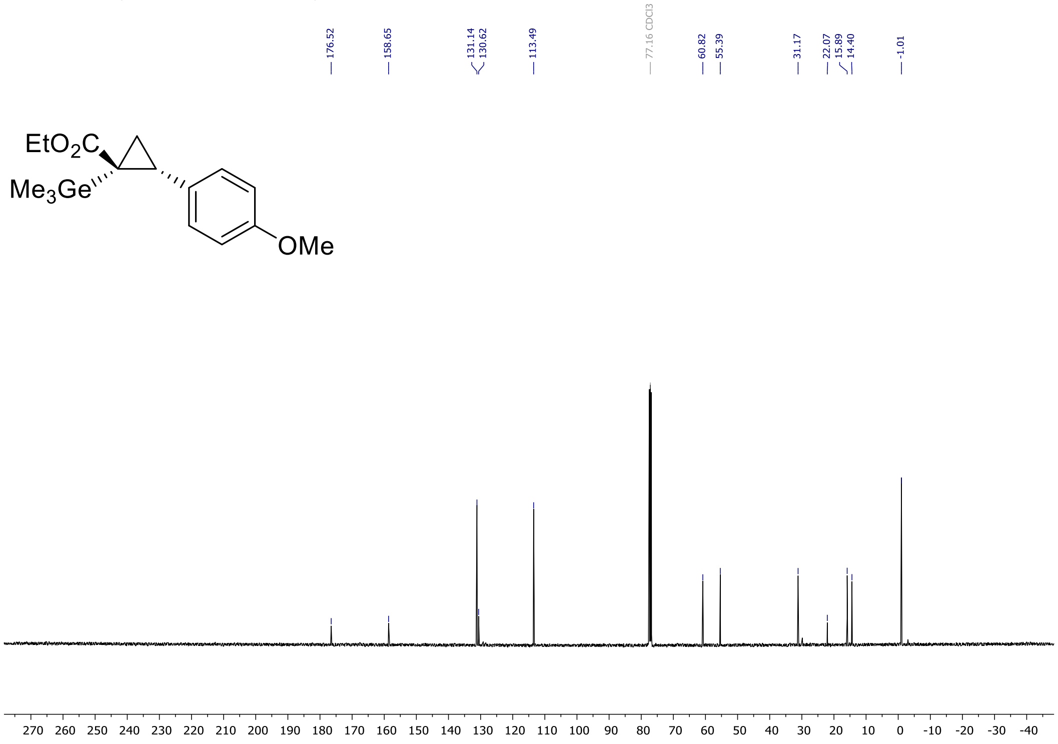

^{1}H-^{1}H-NOESY ($CDCl_3$)

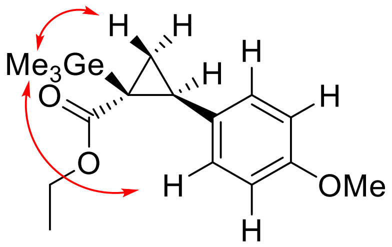

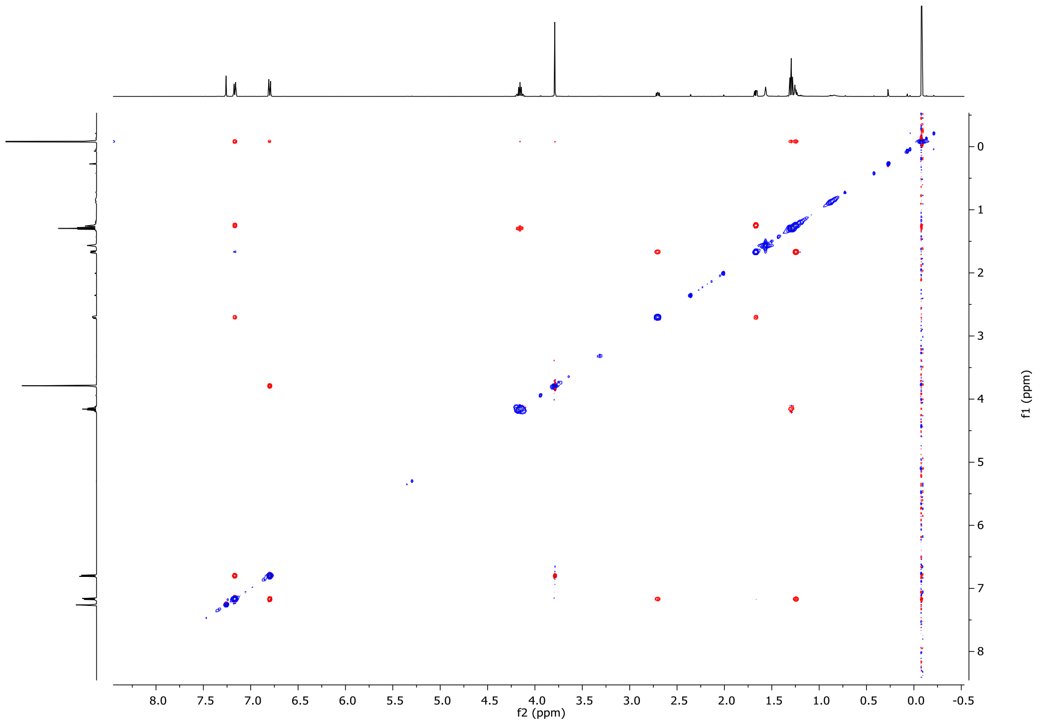

***trans*-2ba:** ^{1}**H-NMR (500 MHz, CDCl$_3$)**

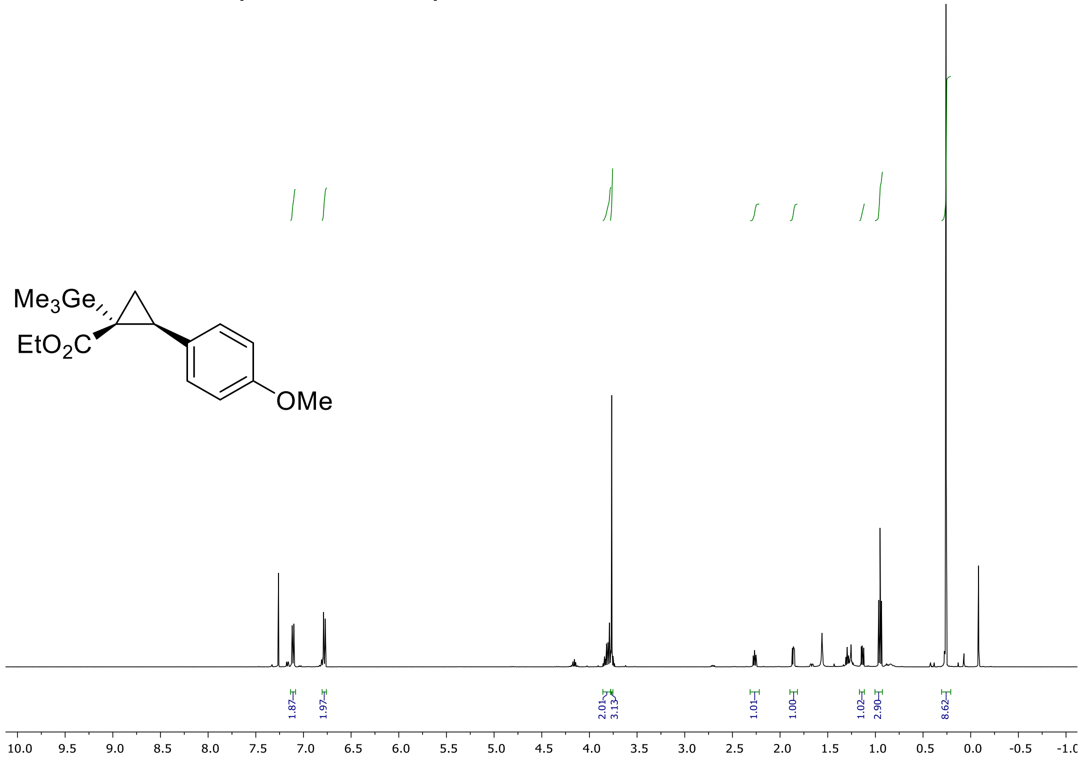

^{13}**C-NMR (100 MHz, CDCl$_3$)**

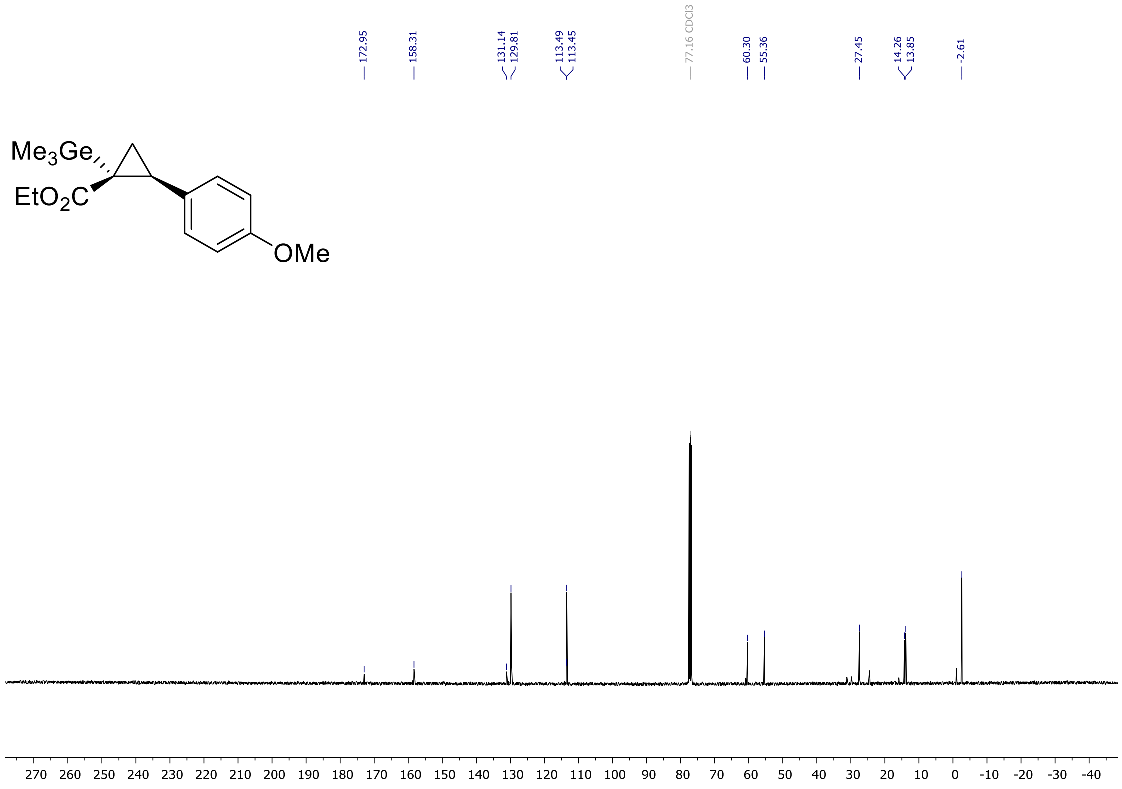

^{1}H-^{1}H-NOESY ($CDCl_3$)

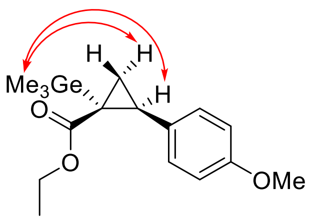

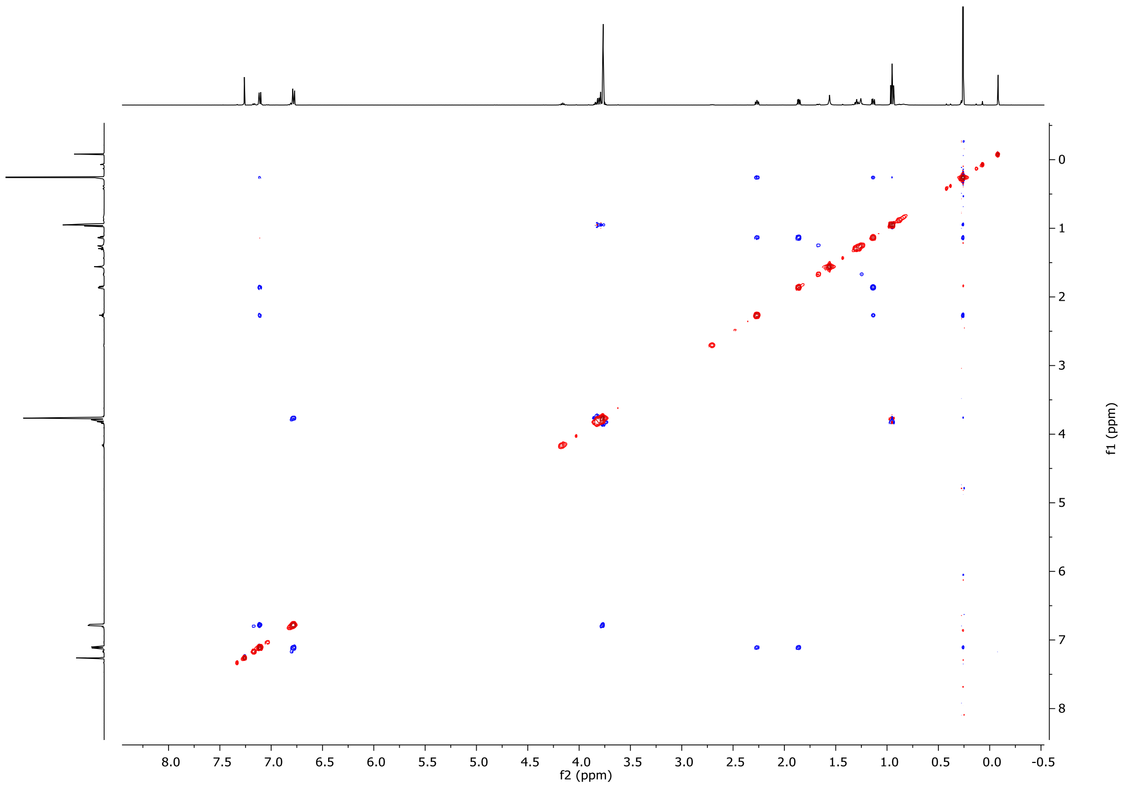

***cis*-2ca:**[1]**H-NMR (500 MHz, $CDCl_3$)**

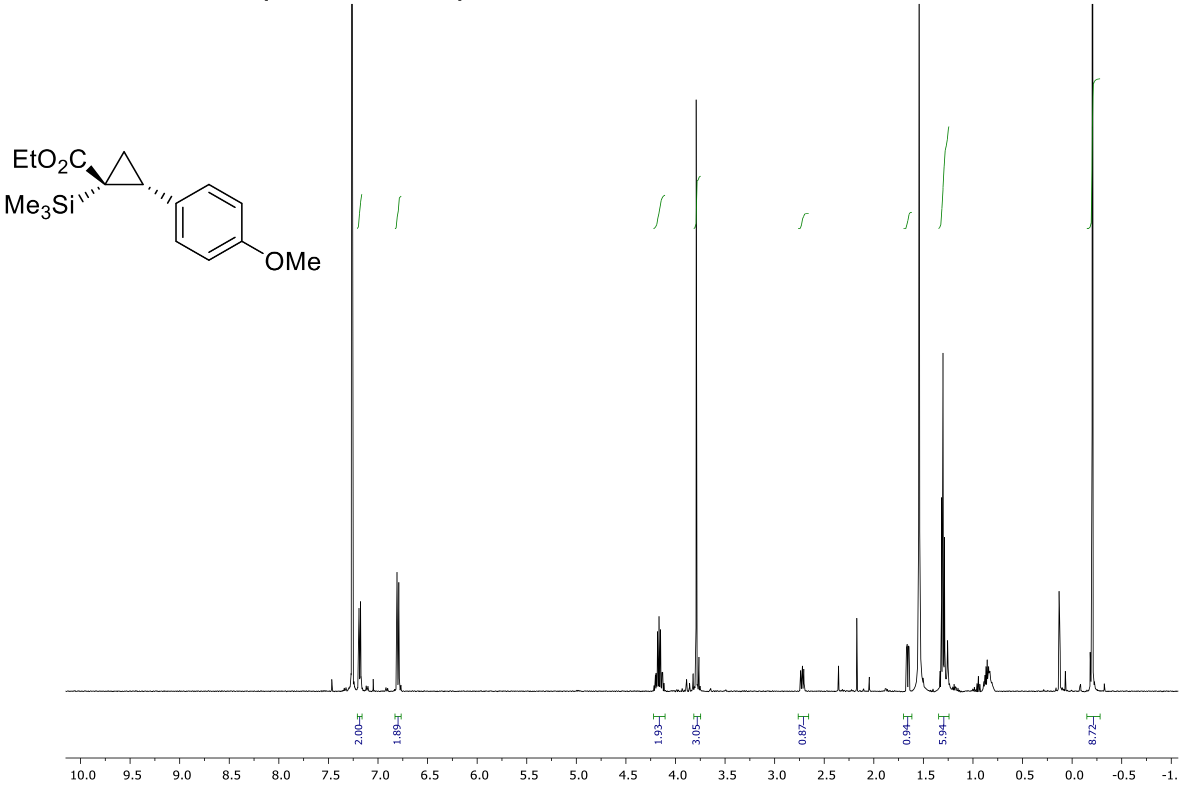

^{13}C-NMR (100 MHz, $CDCl_3$)

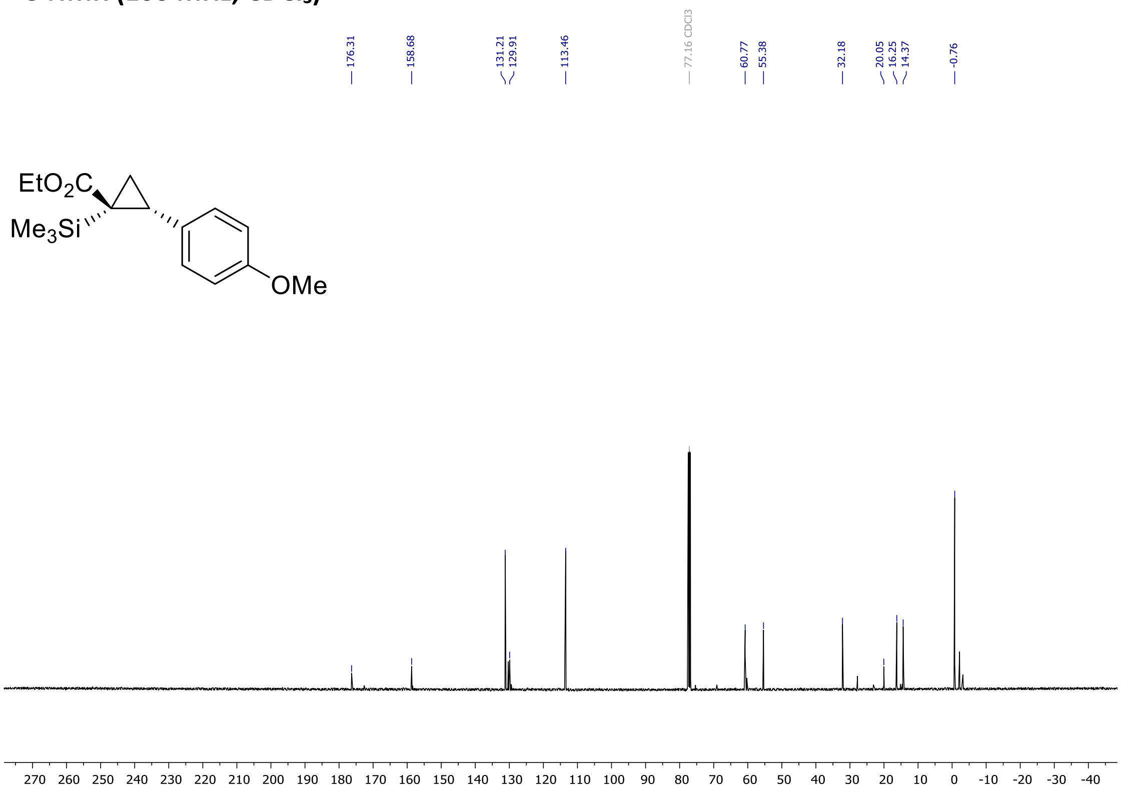

^{1}H-^{1}H-NOESY ($CDCl_3$)

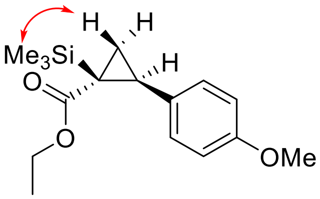

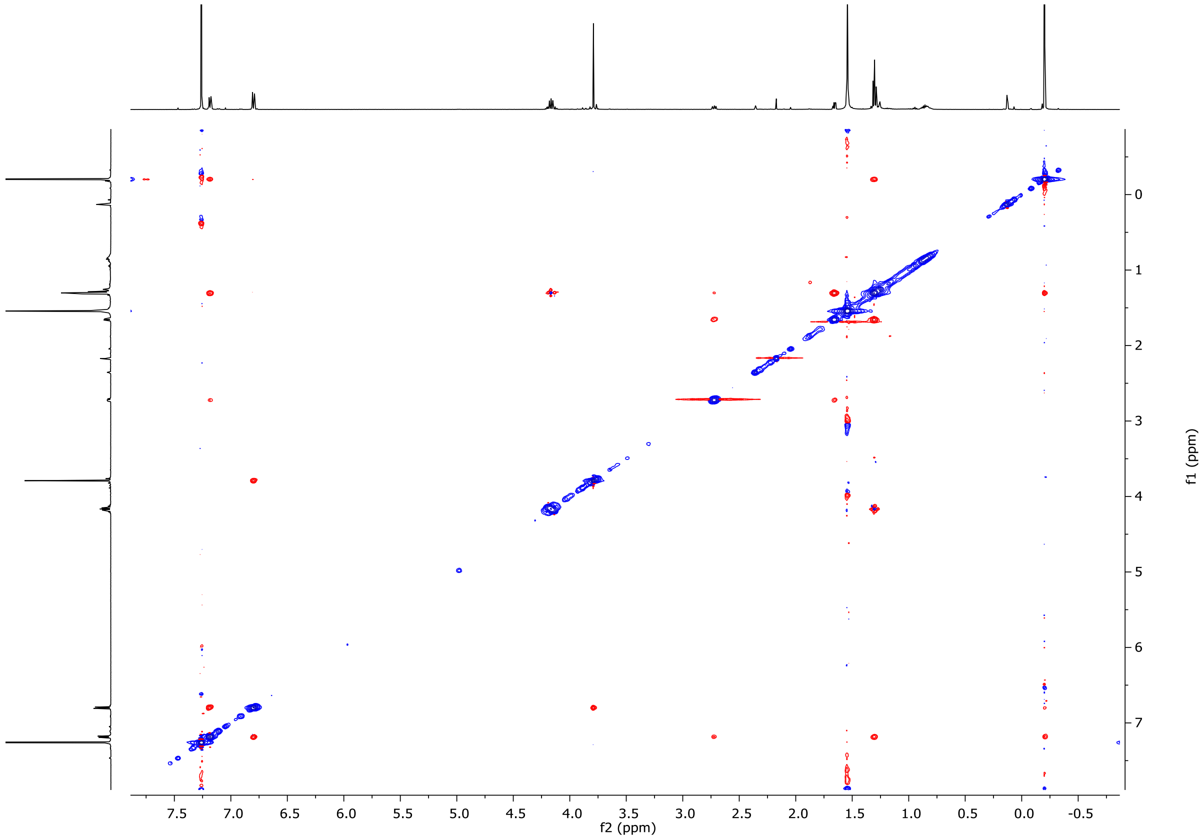

***trans*-2ca:** ^{1}**H-NMR (500 MHz, $CDCl_3$)**

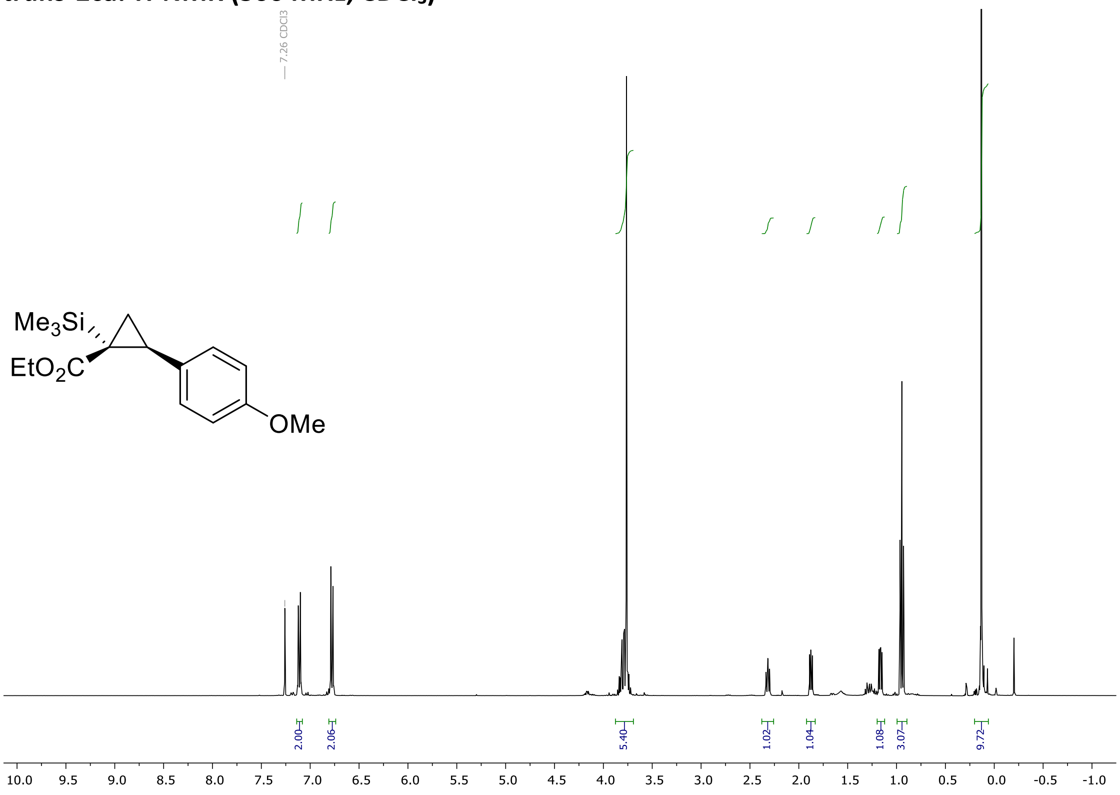

^{13}**C-NMR (100 MHz, $CDCl_3$)**

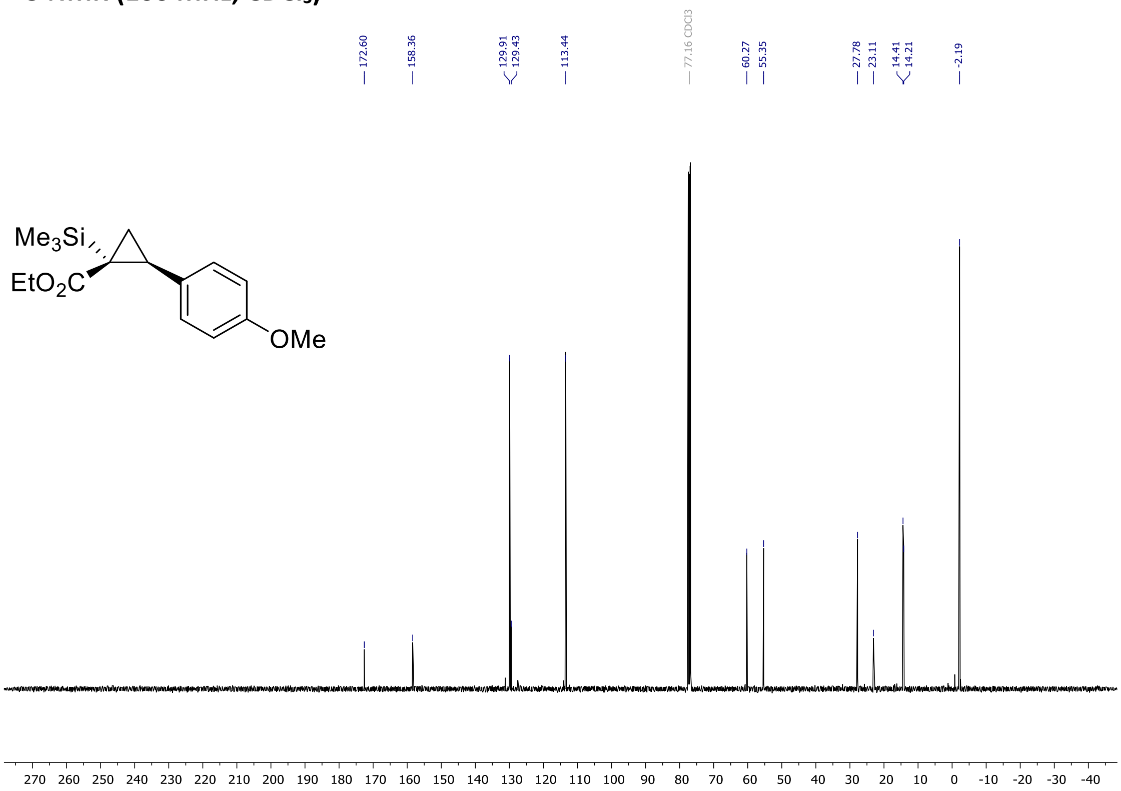

^{1}H-^{1}H-NOESY ($CDCl_3$)

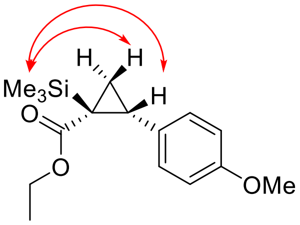

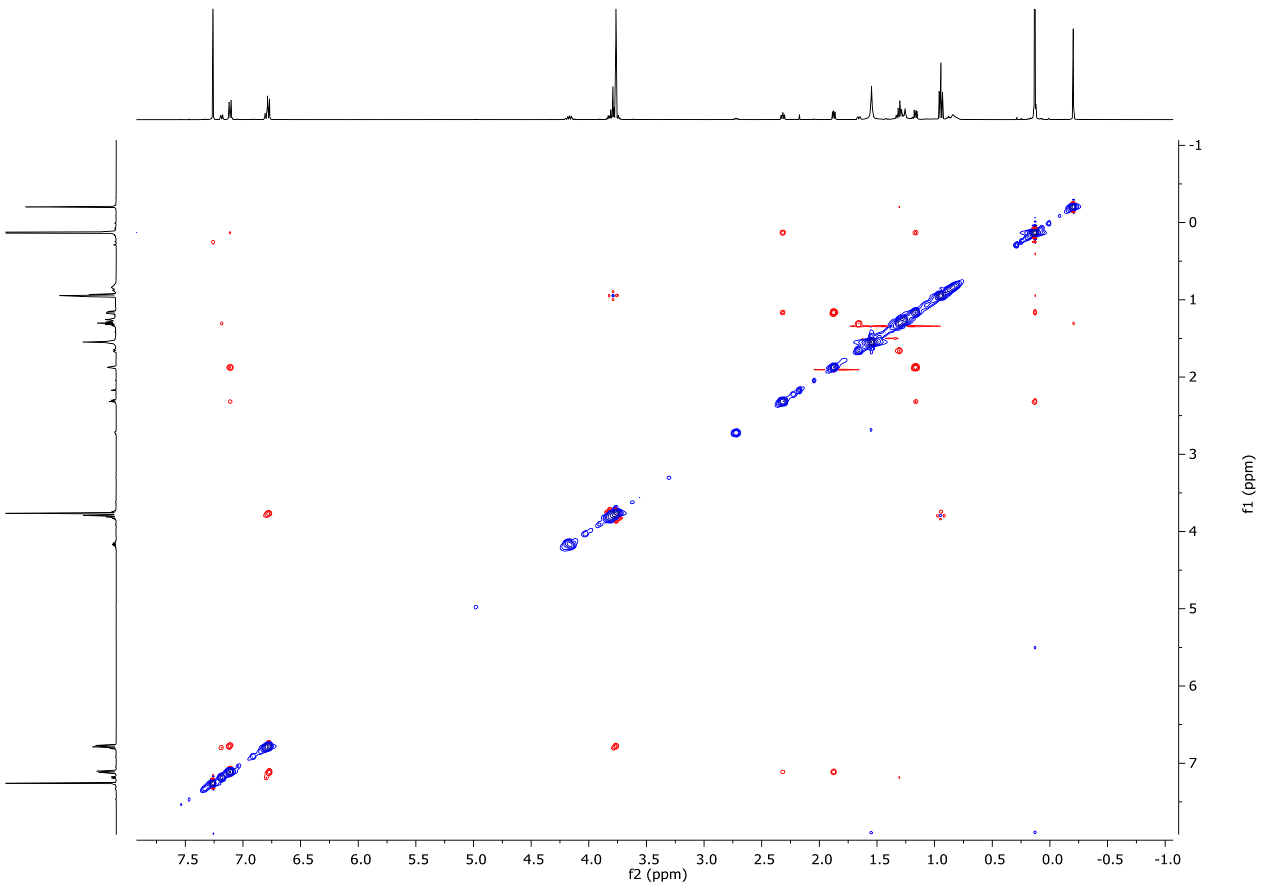

trans-2ab: ^{1}H-NMR (500 MHz, $CDCl_3$)

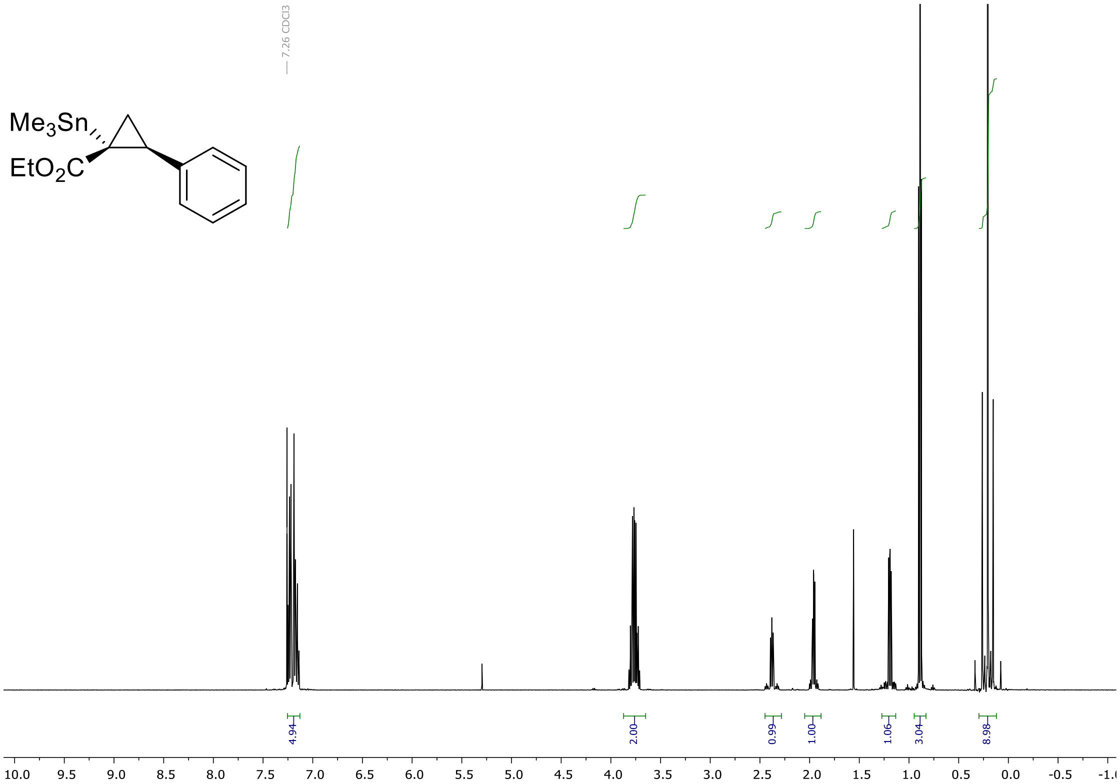

^{13}C-NMR (400 MHz, $CDCl_3$)

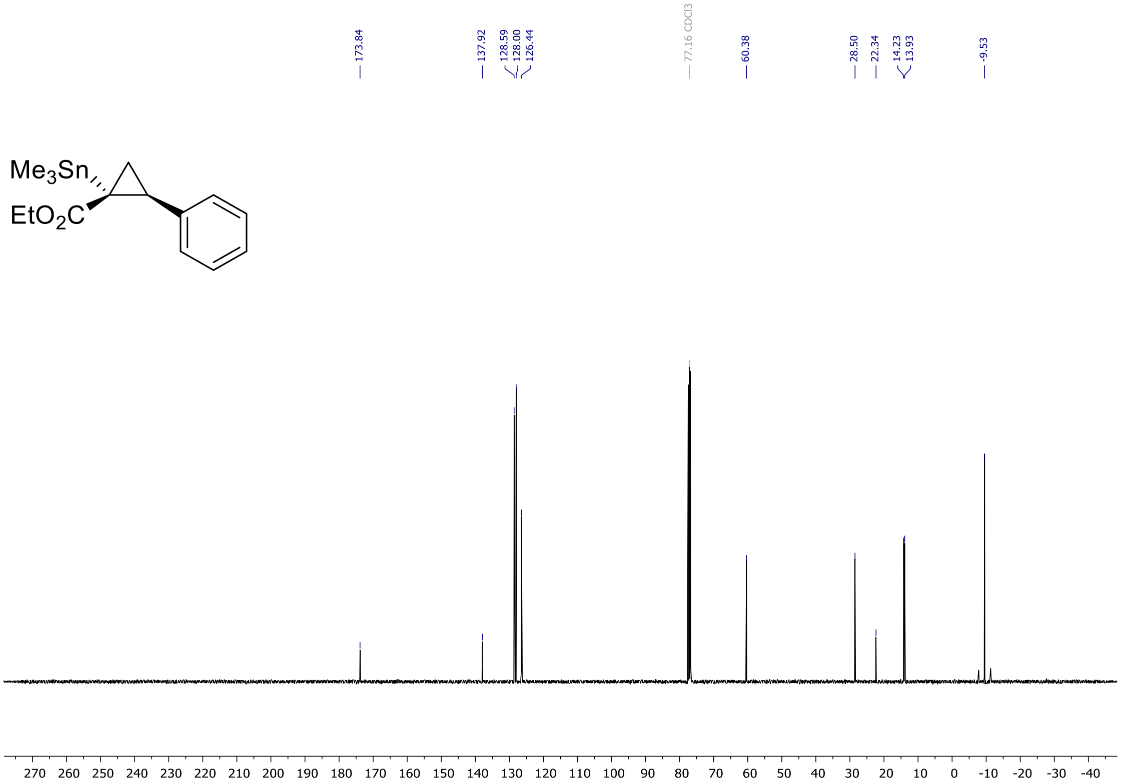

^{119}Sn-NMR (149 MHz, CDCl$_3$)

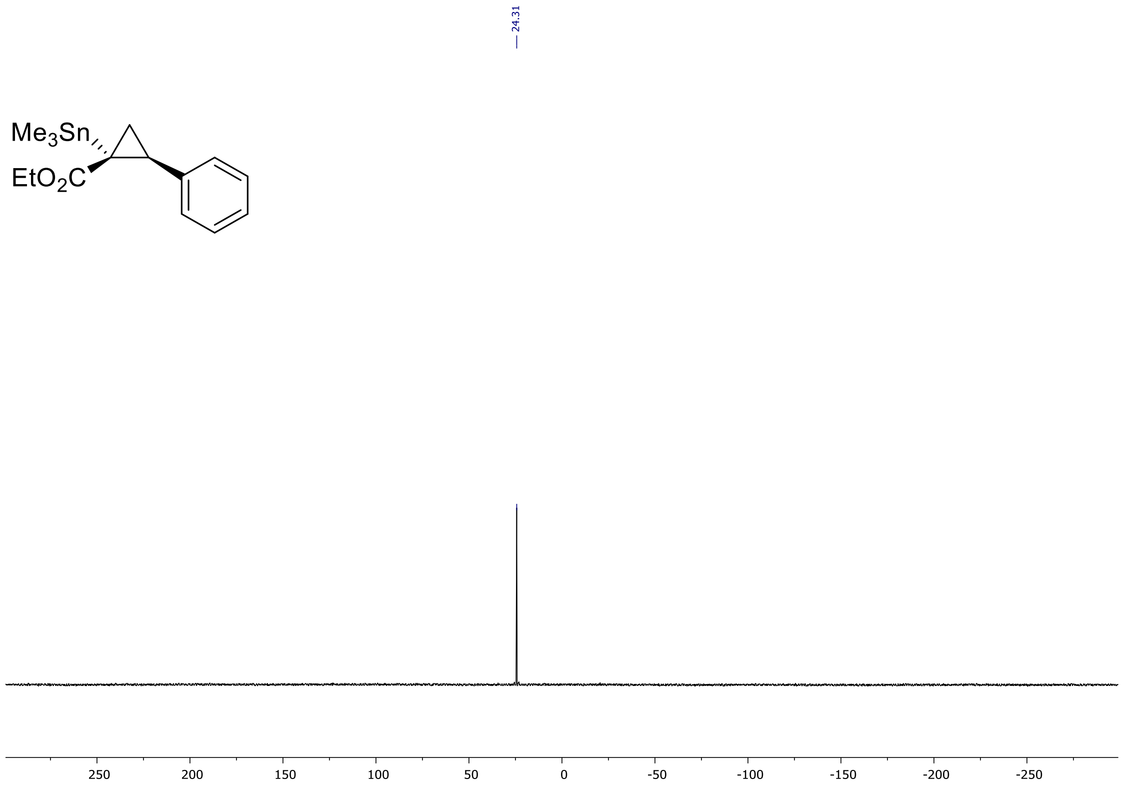

^{1}H-^{1}H NOESY (CDCl$_3$)

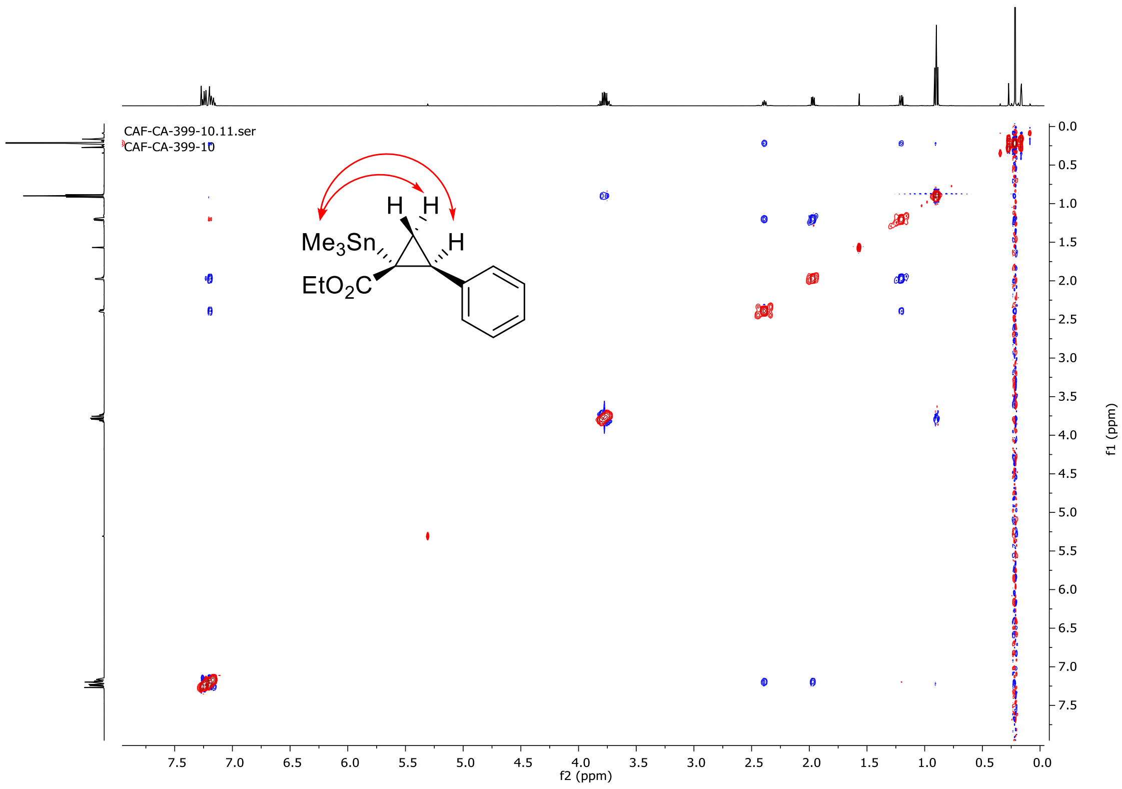

cis-2ab: ^{1}H-NMR (500 MHz, $CDCl_3$)

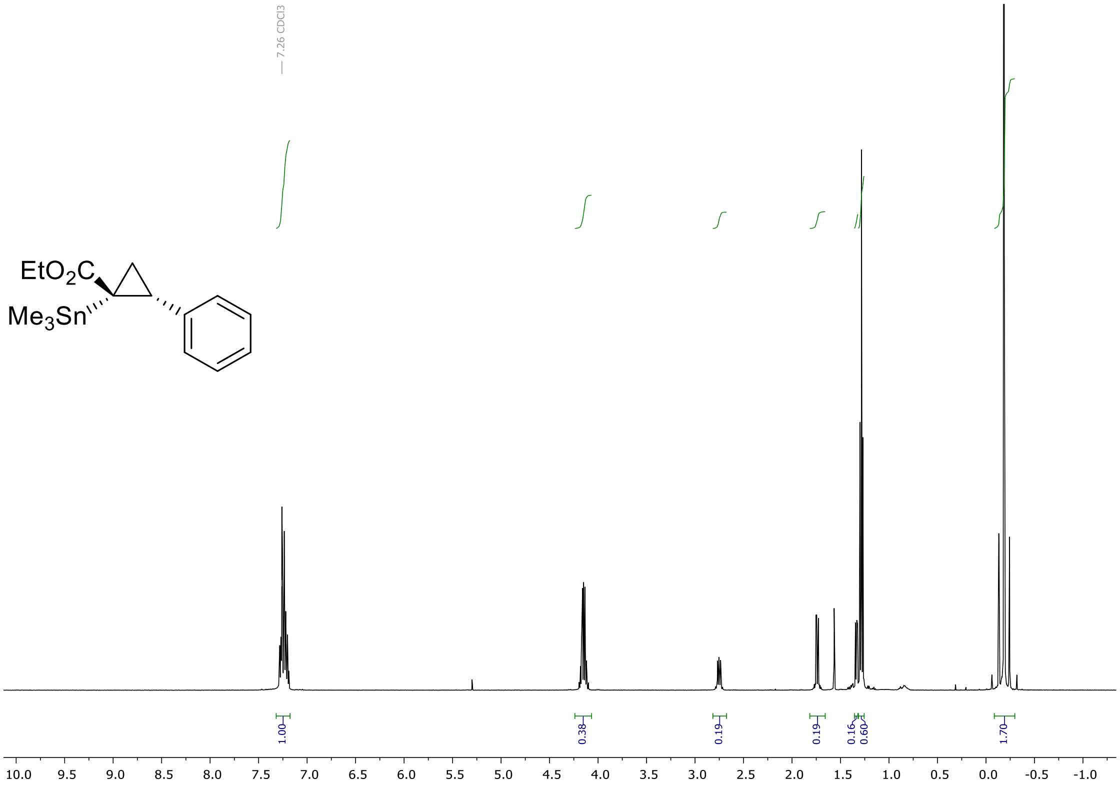

^{13}C-NMR (400 MHz, $CDCl_3$)

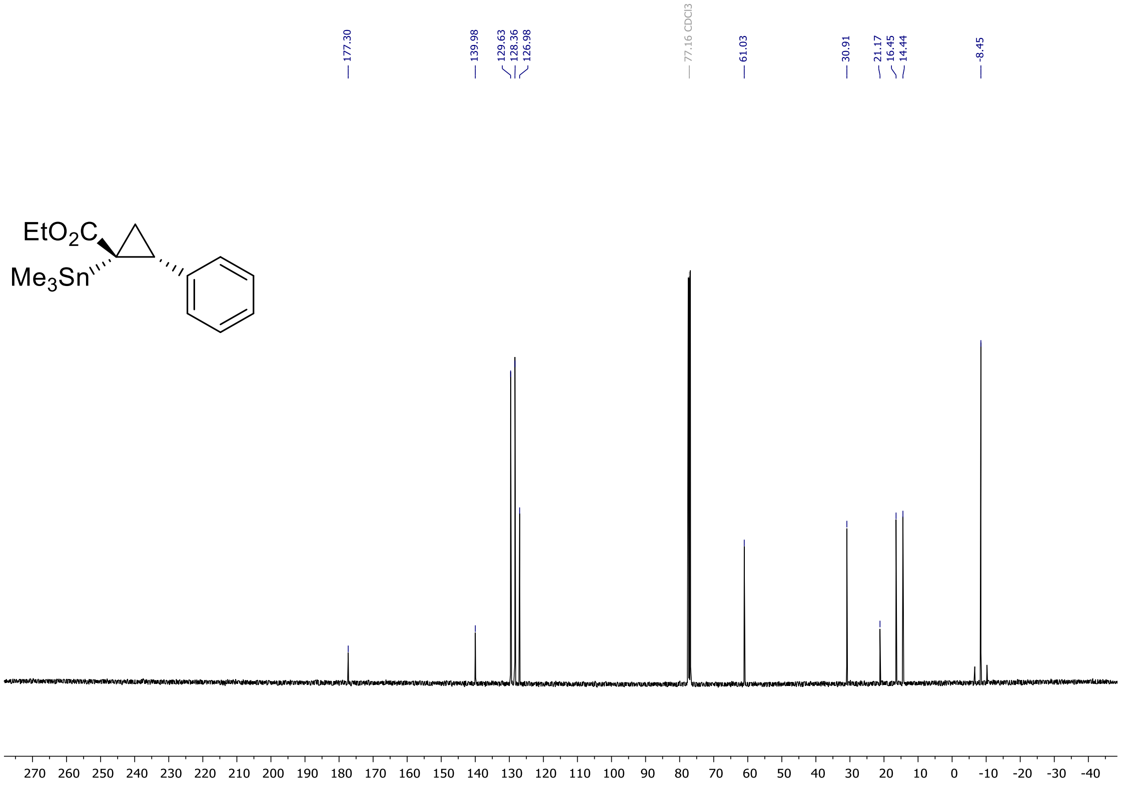

^{119}Sn-NMR (149 MHz, CDCl$_3$)

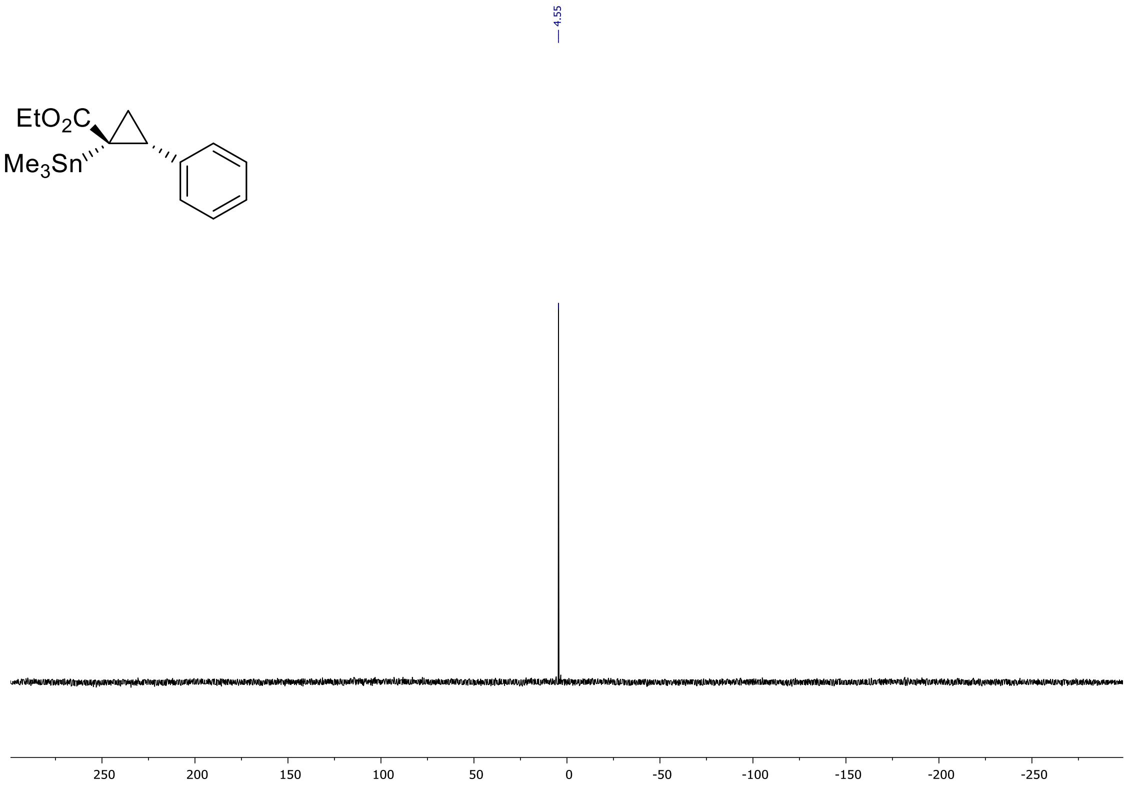

^{1}H-^{1}H NOESY (CDCl$_3$)

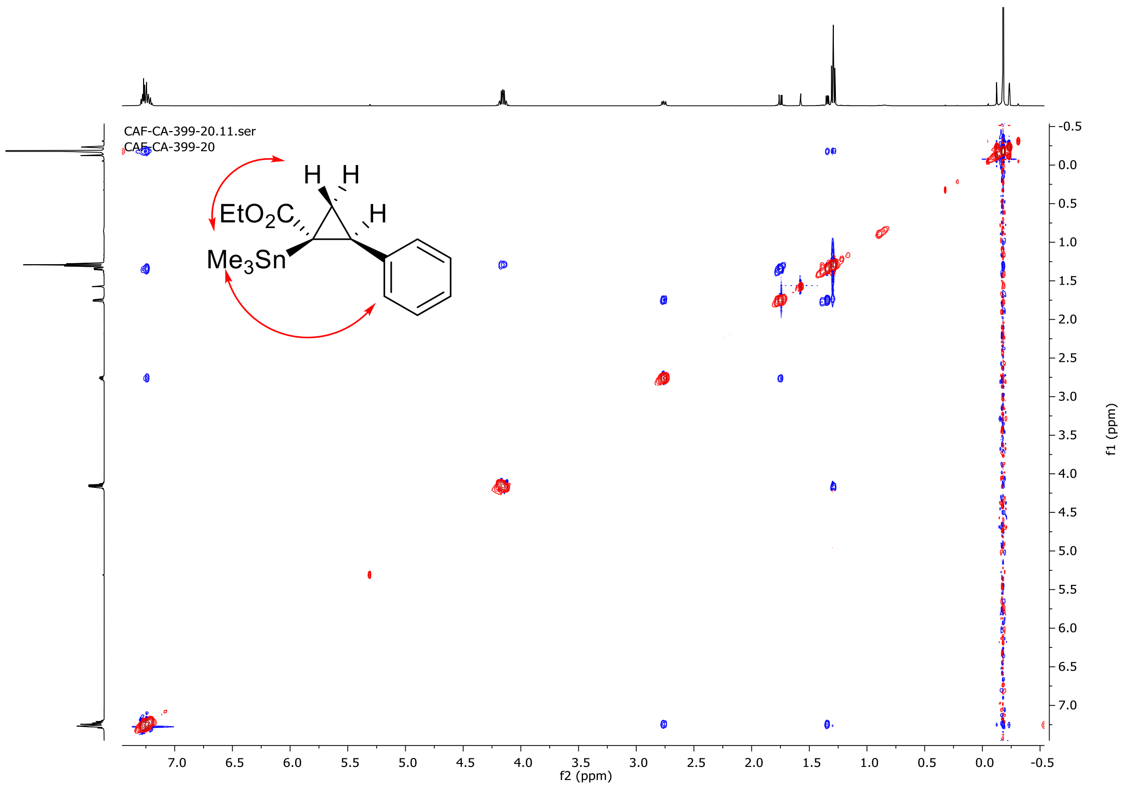

***trans*-2ac: ^{1}H-NMR (400 MHz, $CDCl_3$)**

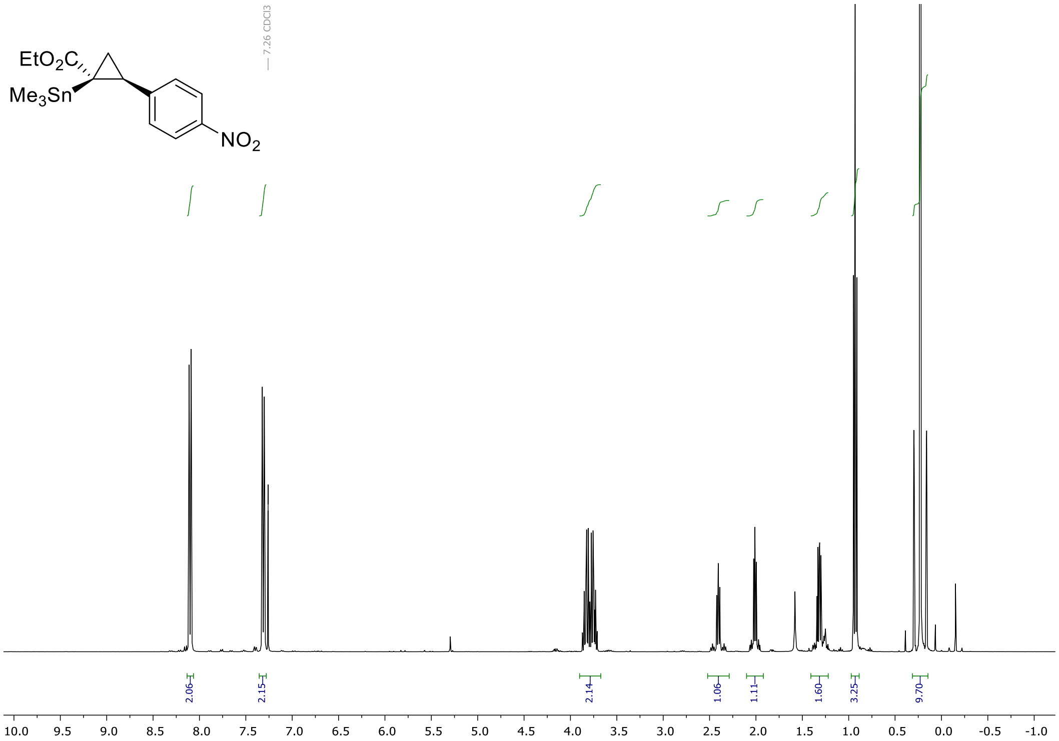

^{13}C-NMR (100 MHz, $CDCl_3$)

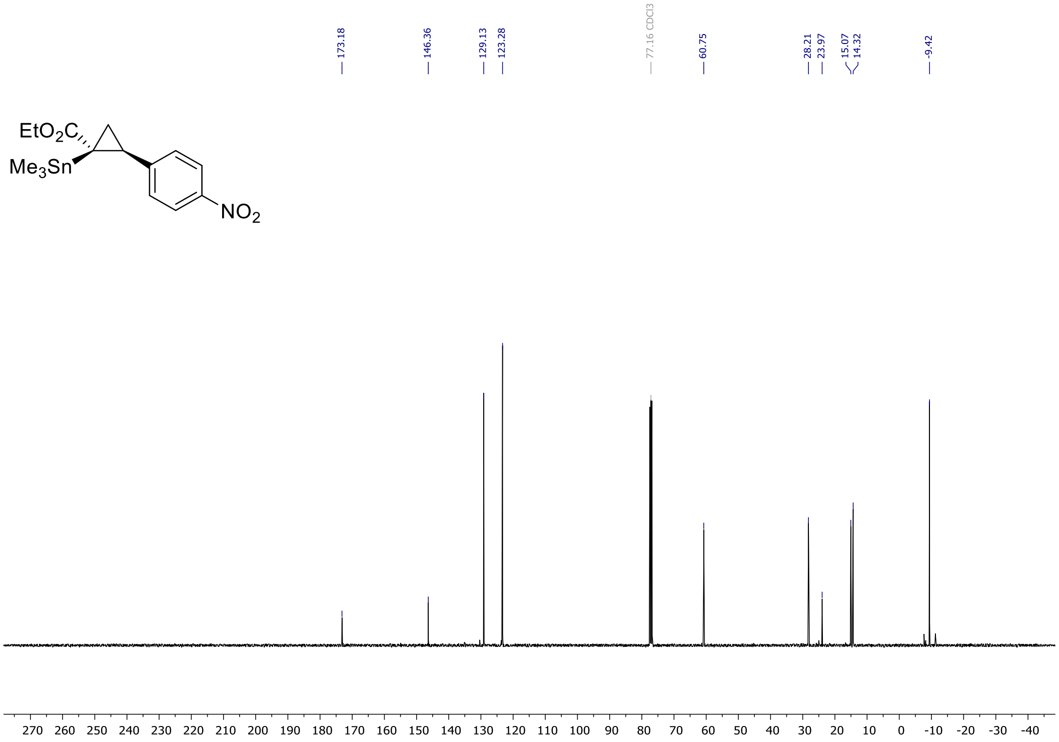

^{119}Sn-NMR (149 MHz, $CDCl_3$)

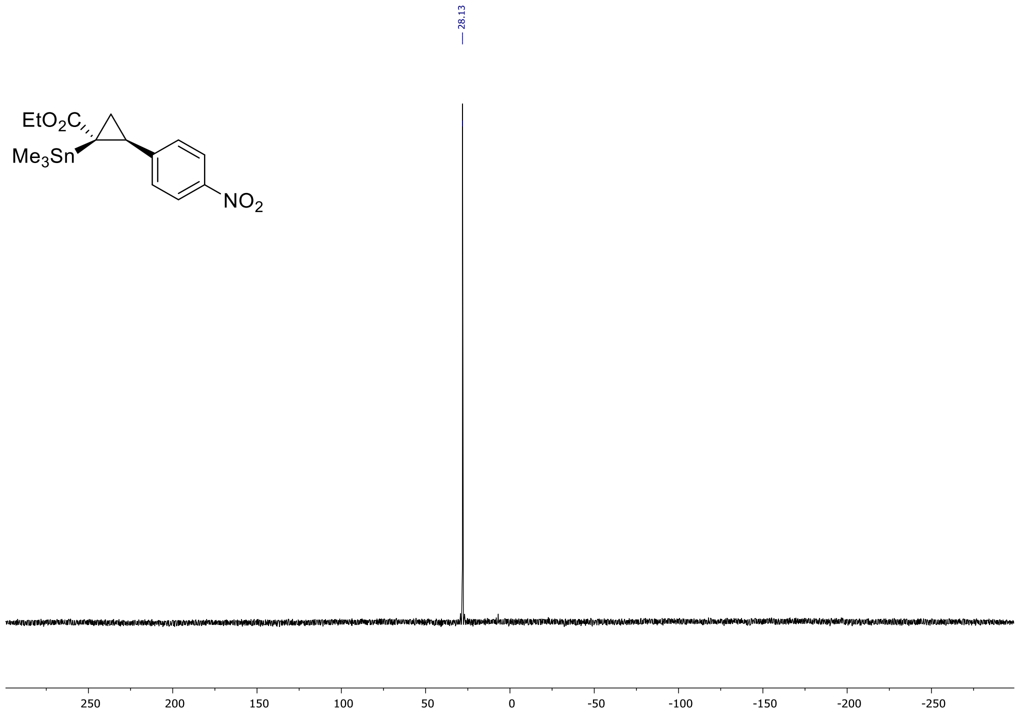

^{1}H-^{1}H NOESY ($CDCl_3$)

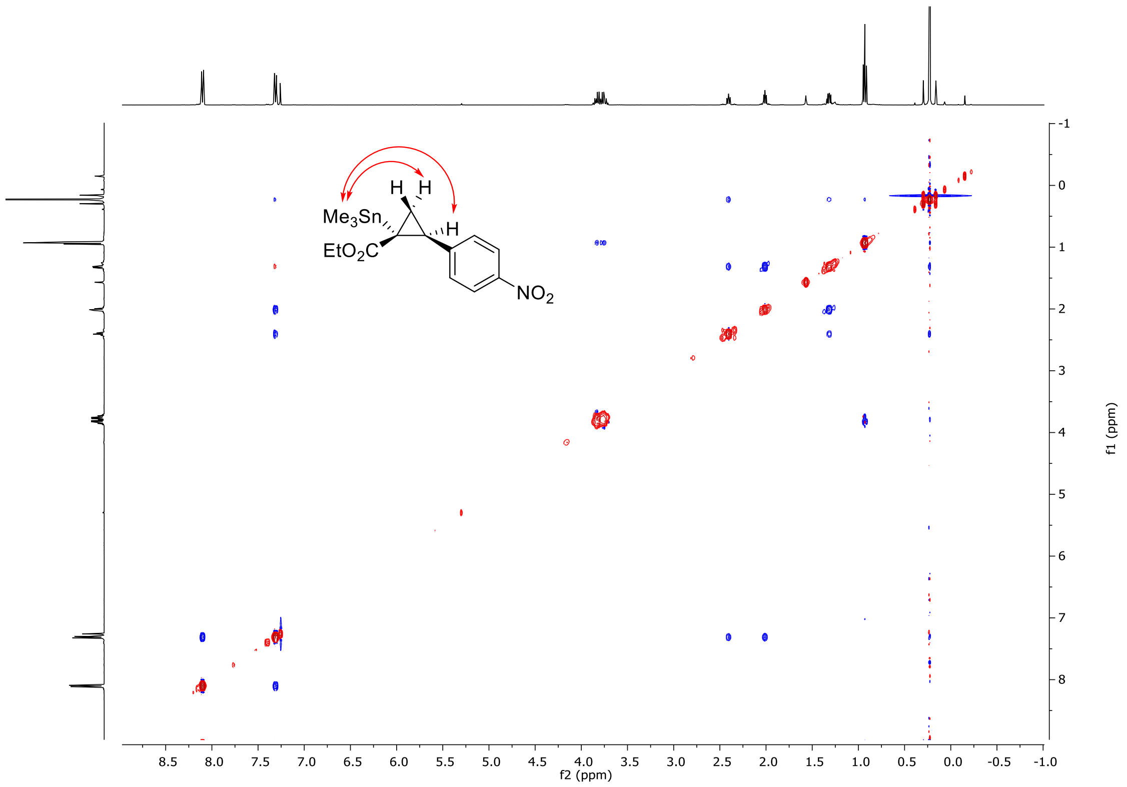

***cis*-2ac: ^{1}H-NMR (400 MHz, $CDCl_3$)**

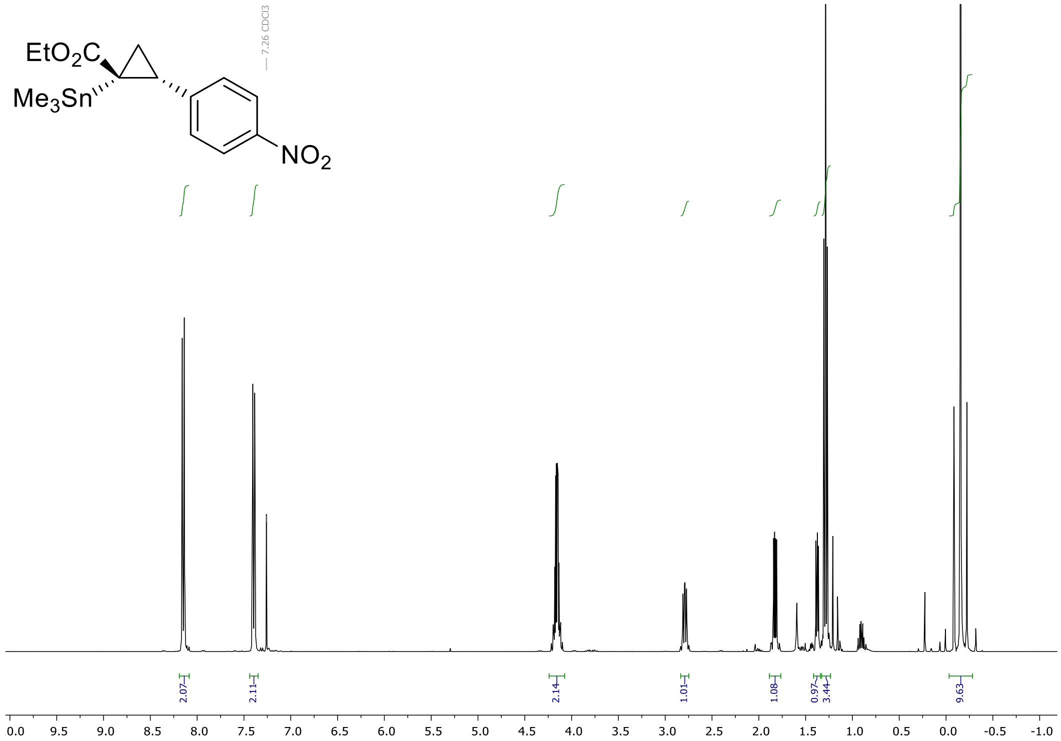

^{13}C-NMR (100 MHz, $CDCl_3$)

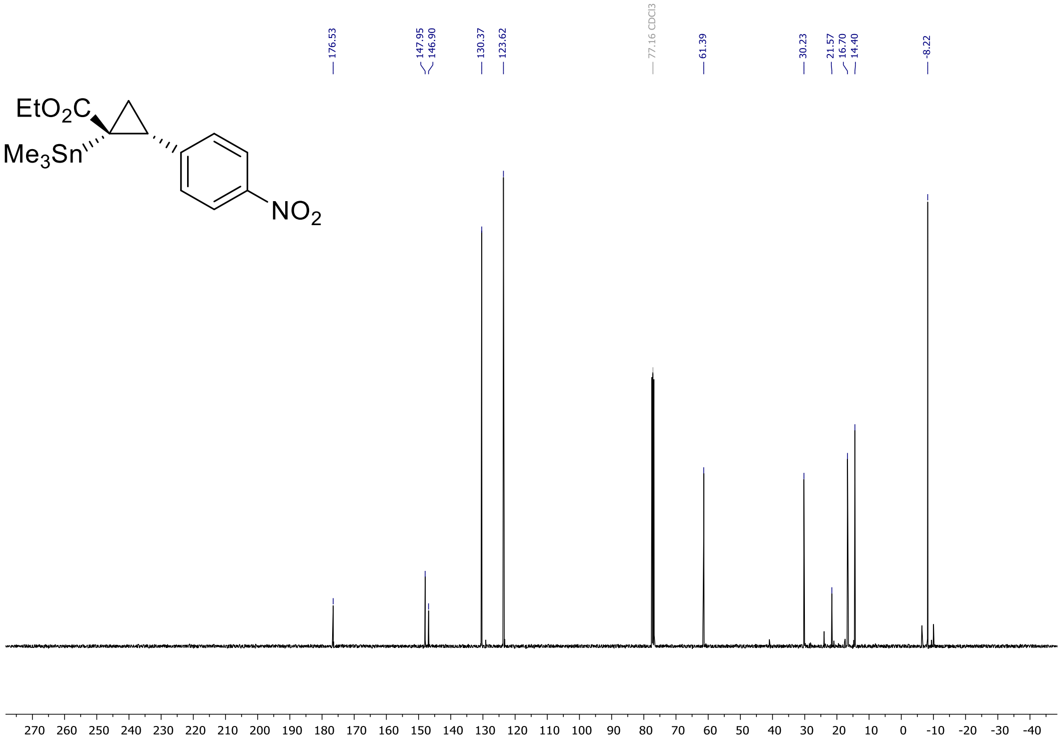

^{119}Sn-NMR (149 MHz, $CDCl_3$)

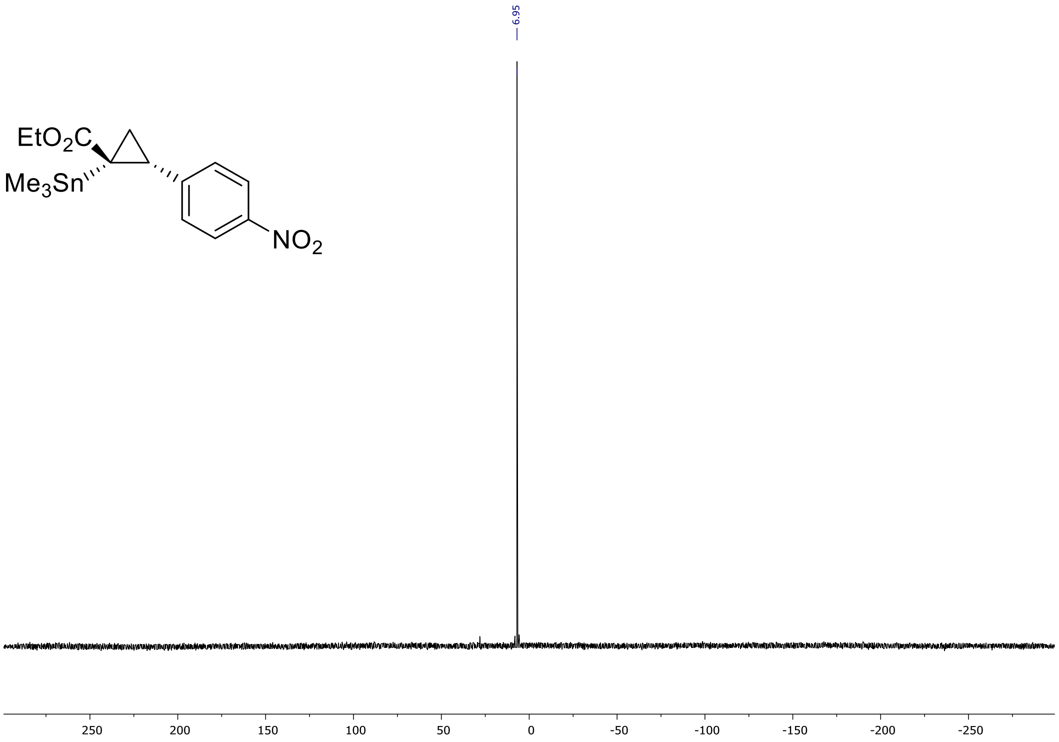

^{1}H-^{1}H NOESY ($CDCl_3$)

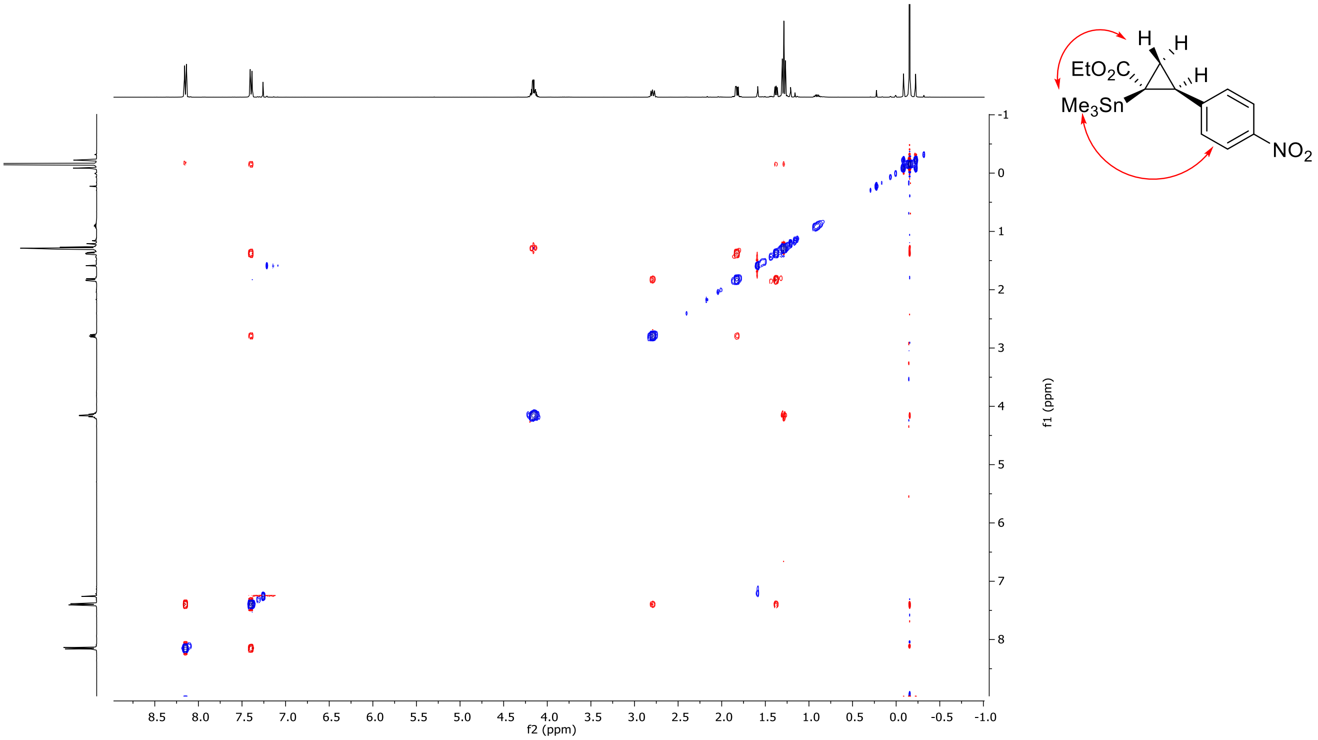

trans-2ad:^{1}H-NMR (400 MHz, $CDCl_3$)

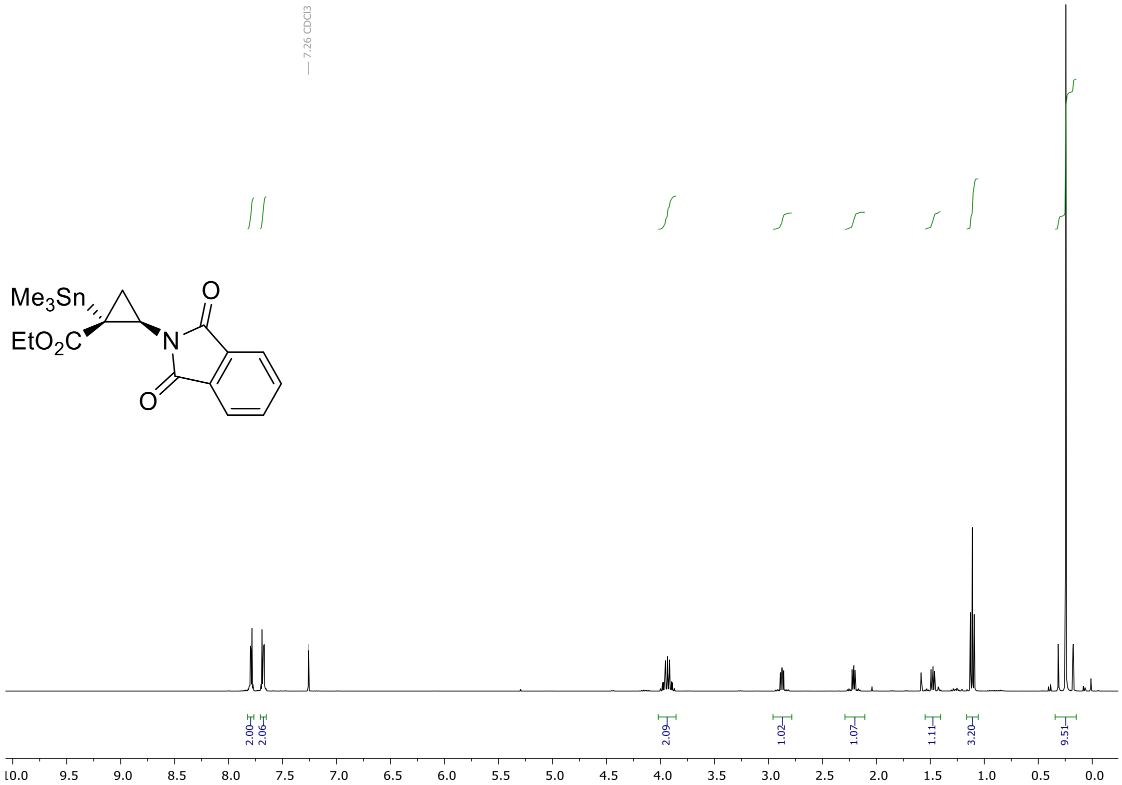

^{13}C-NMR (100 MHz, $CDCl_3$)

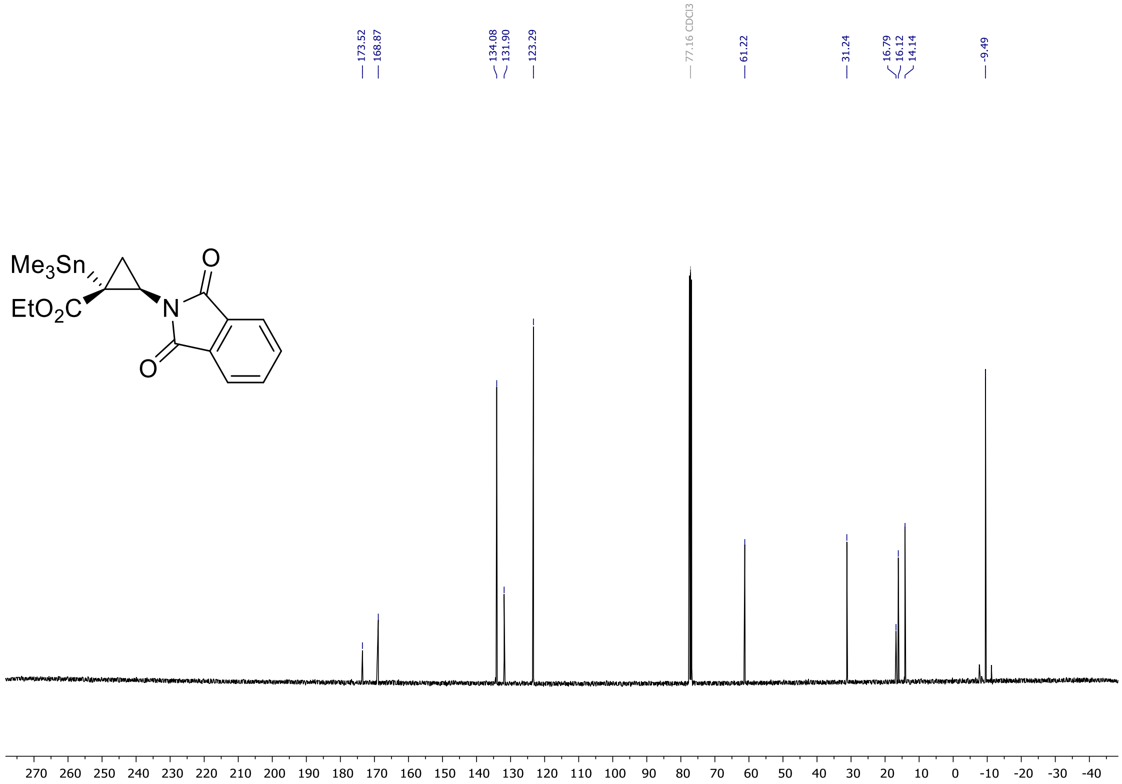

^{119}Sn-NMR (149 MHz, CDCl$_3$)

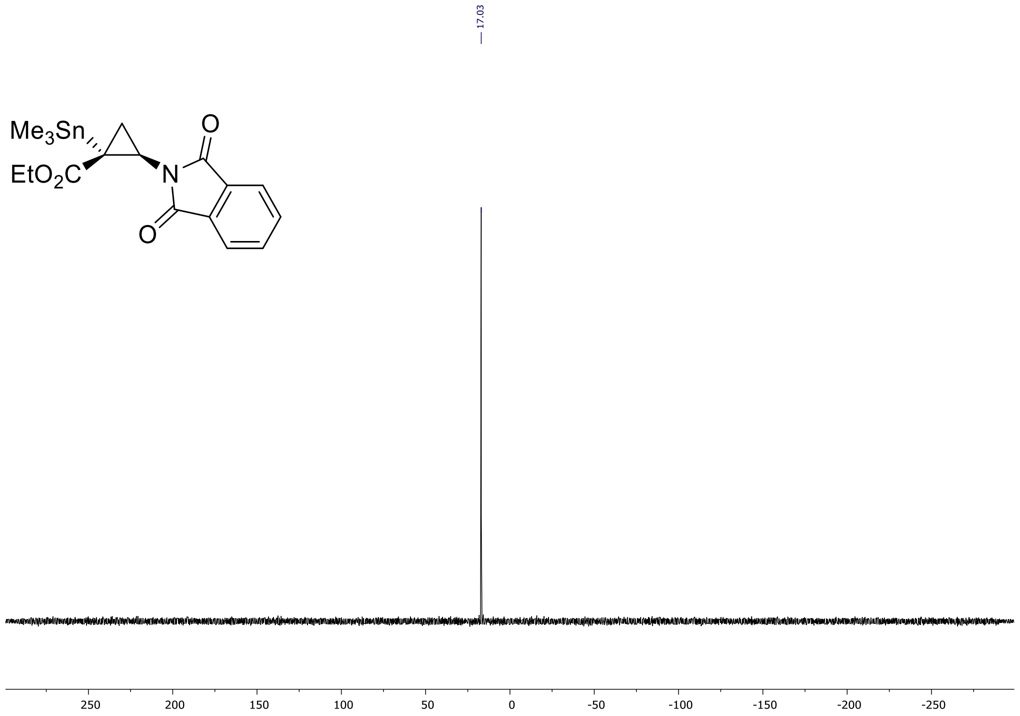

^{1}H-^{1}H-NOESY (CDCl$_3$)

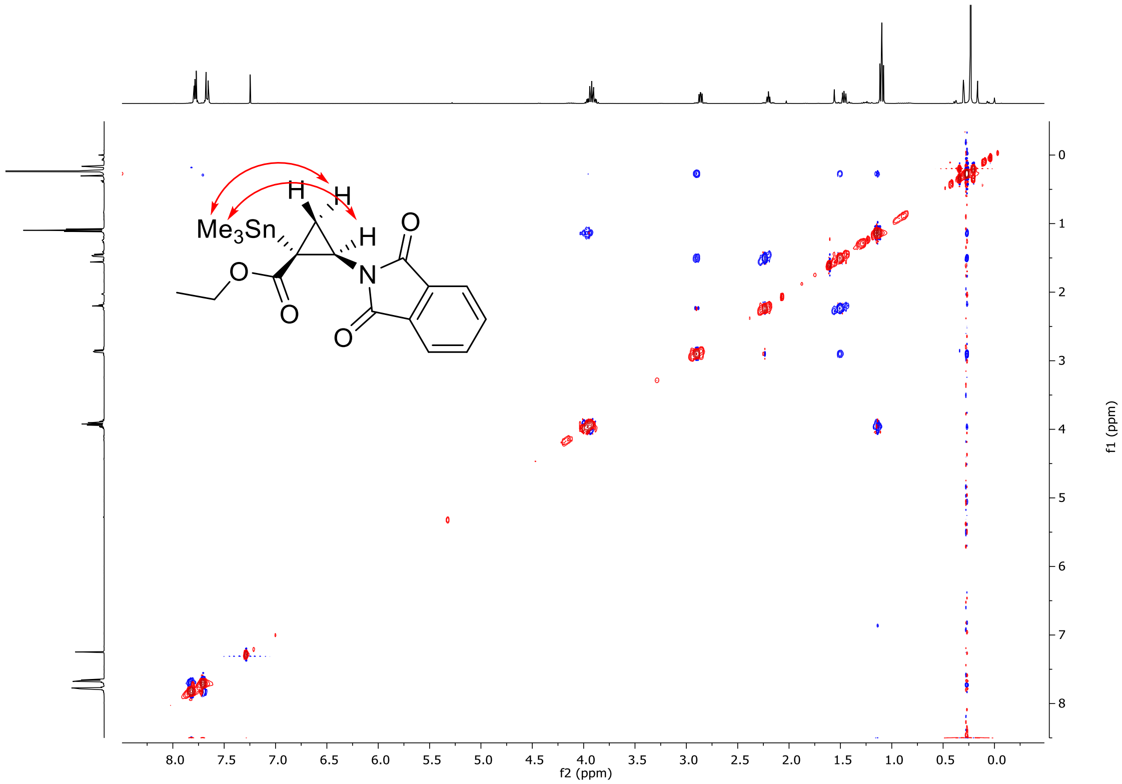

***cis*-2ad: ^{1}H-NMR (400 MHz, $CDCl_3$)**

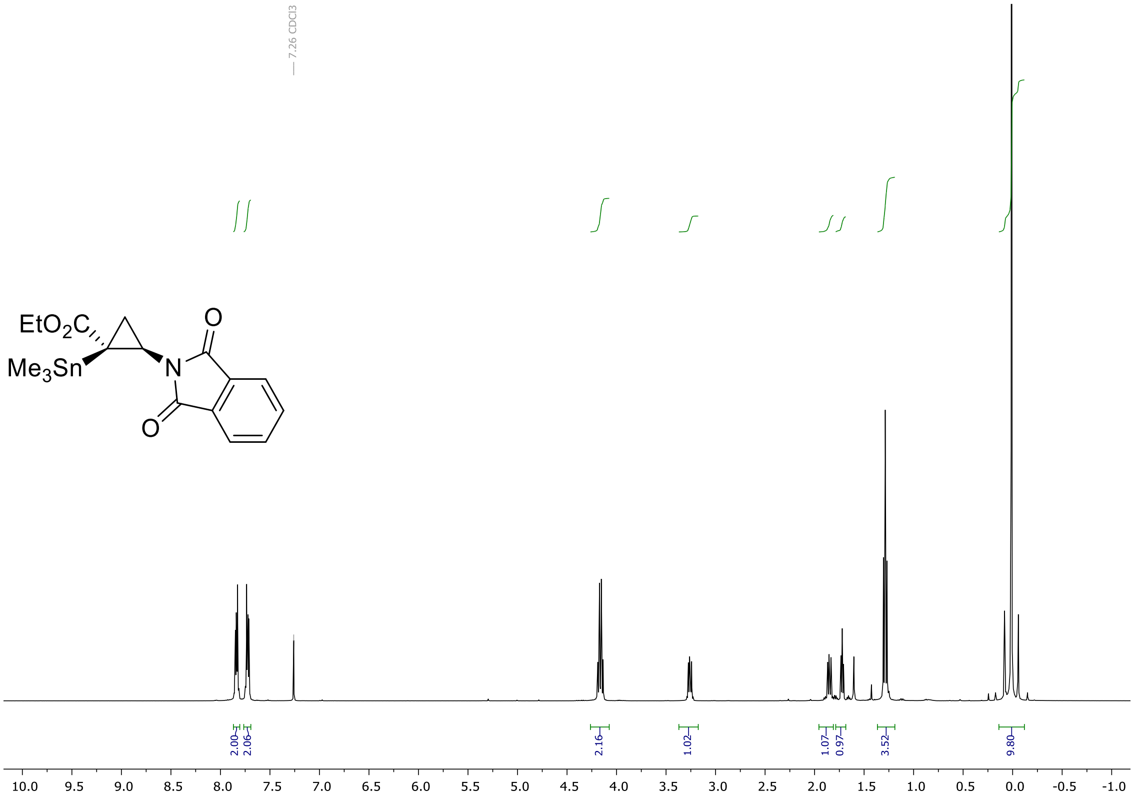

^{13}C-NMR (100 MHz, $CDCl_3$)

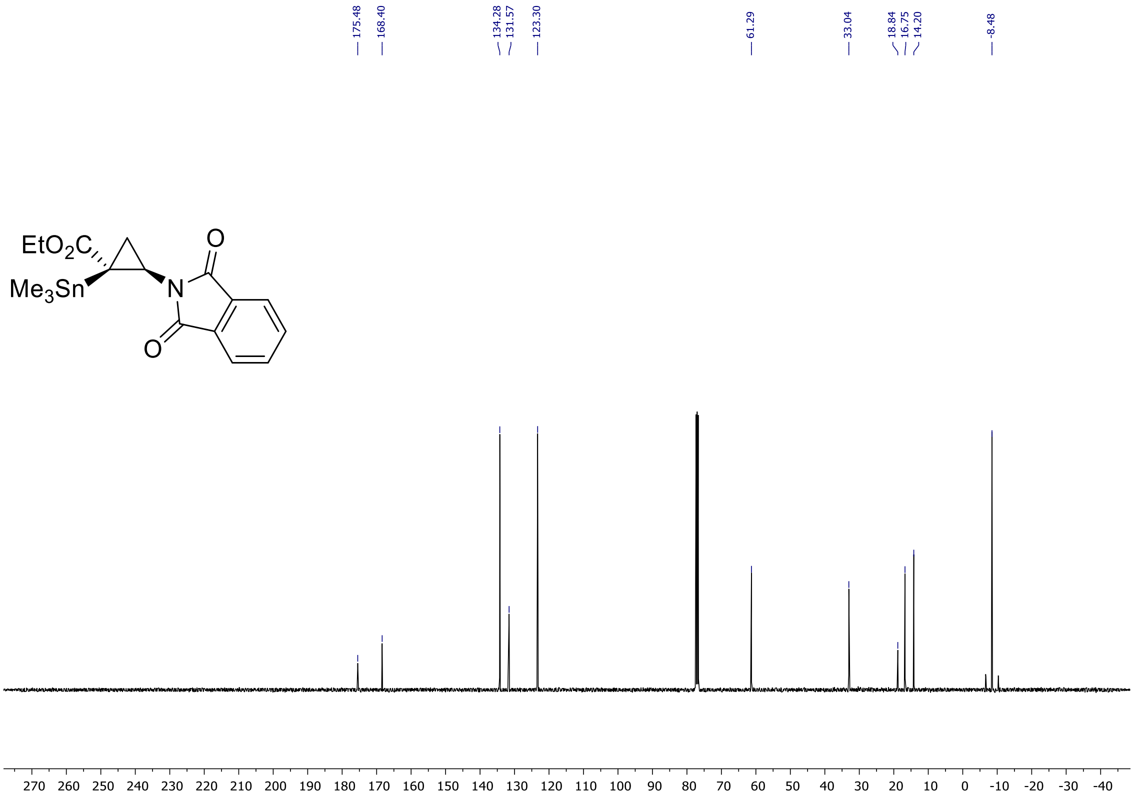

^{119}Sn-NMR (149 MHz, $CDCl_3$)

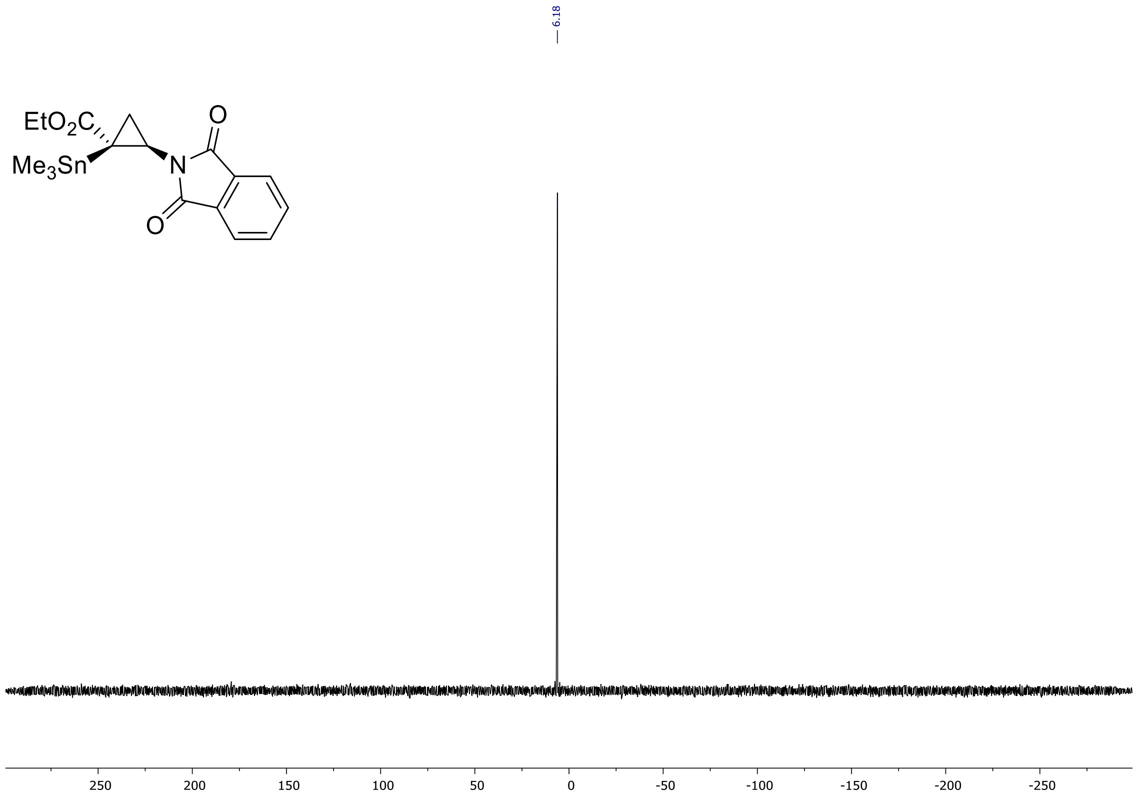

^{1}H-^{1}H-NOESY ($CDCl_3$)

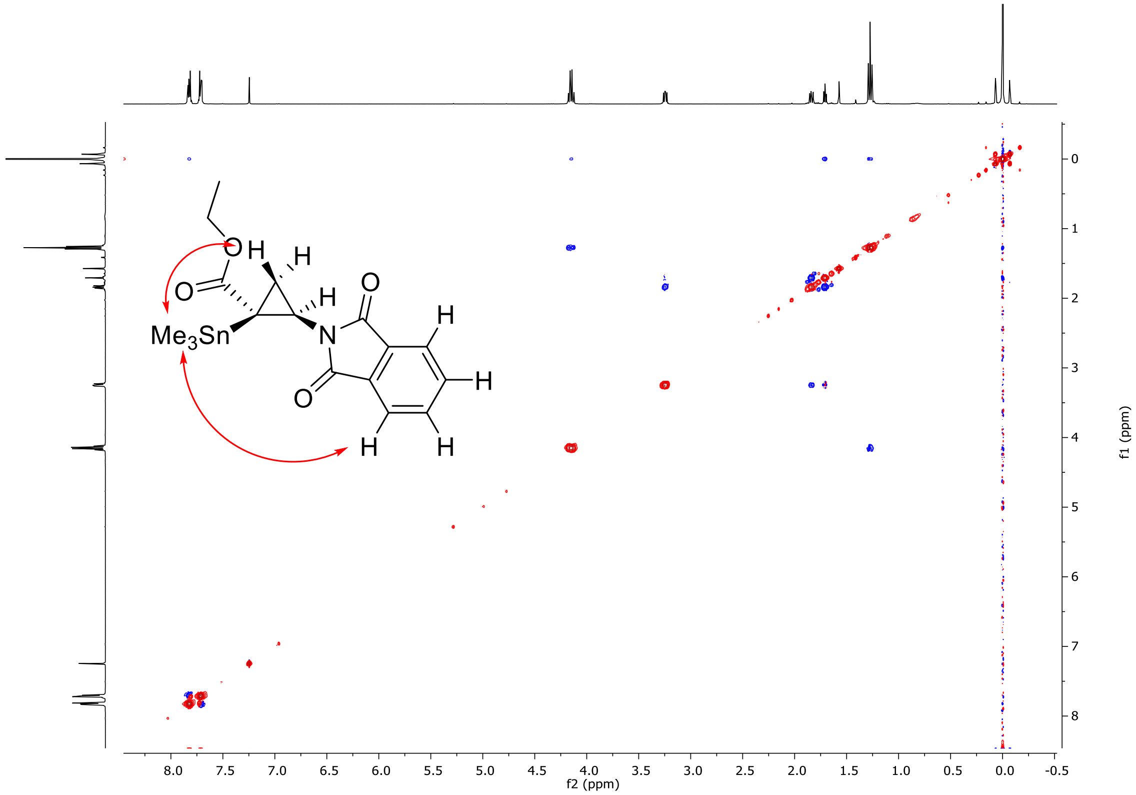

***cis*-2ae: ^{1}H-NMR (400 MHz, $CDCl_3$)**

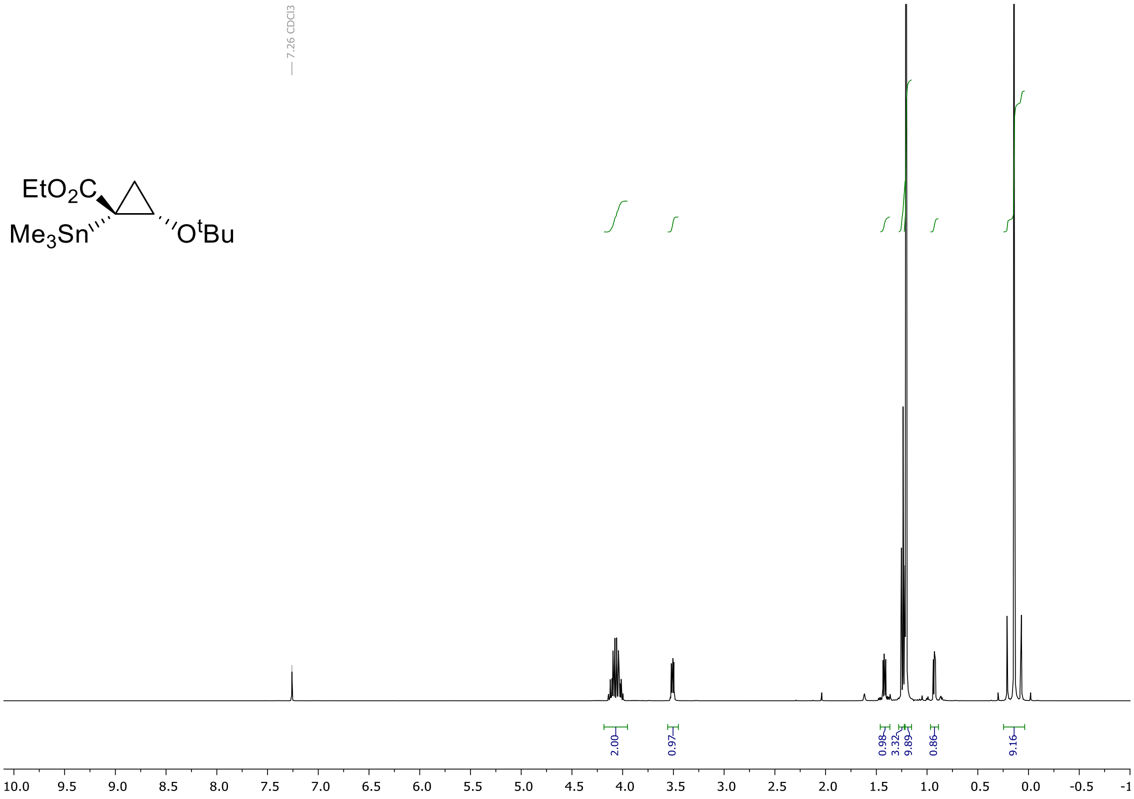

^{13}C{^{1}H}-NMR (100MHz, $CDCl_3$)

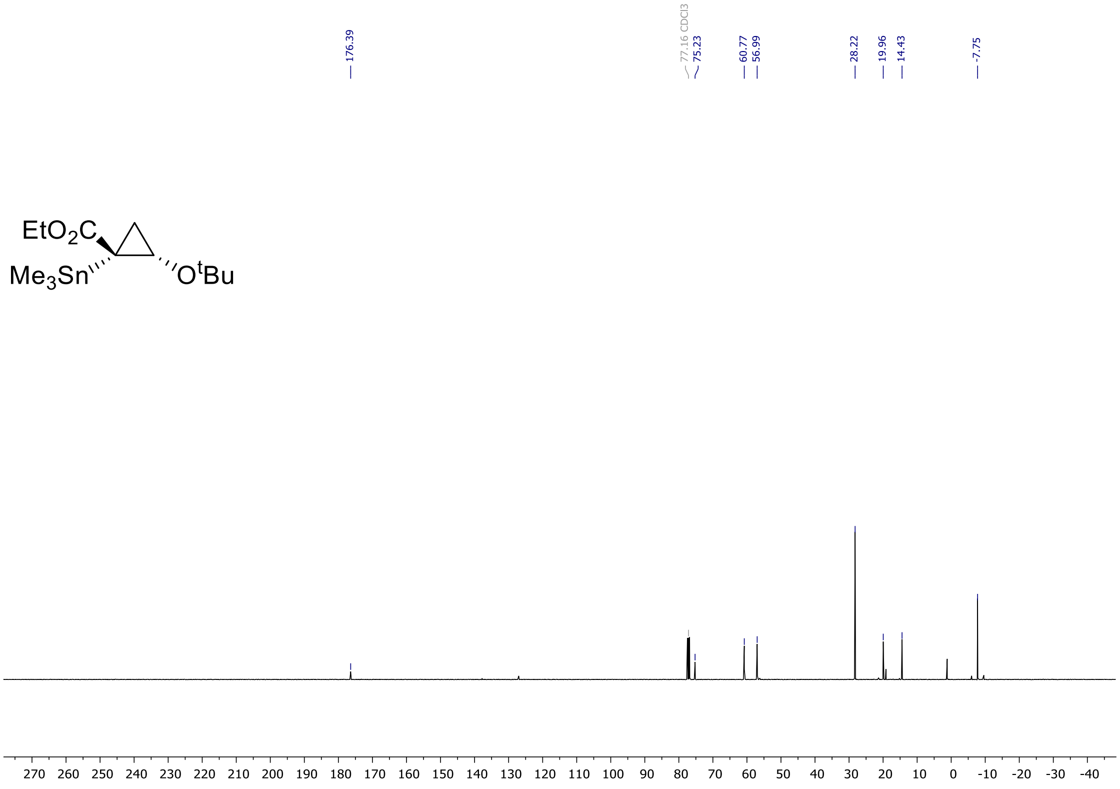

$^{119}Sn\{^1H\}$-NMR (149 MHz, $CDCl_3$)

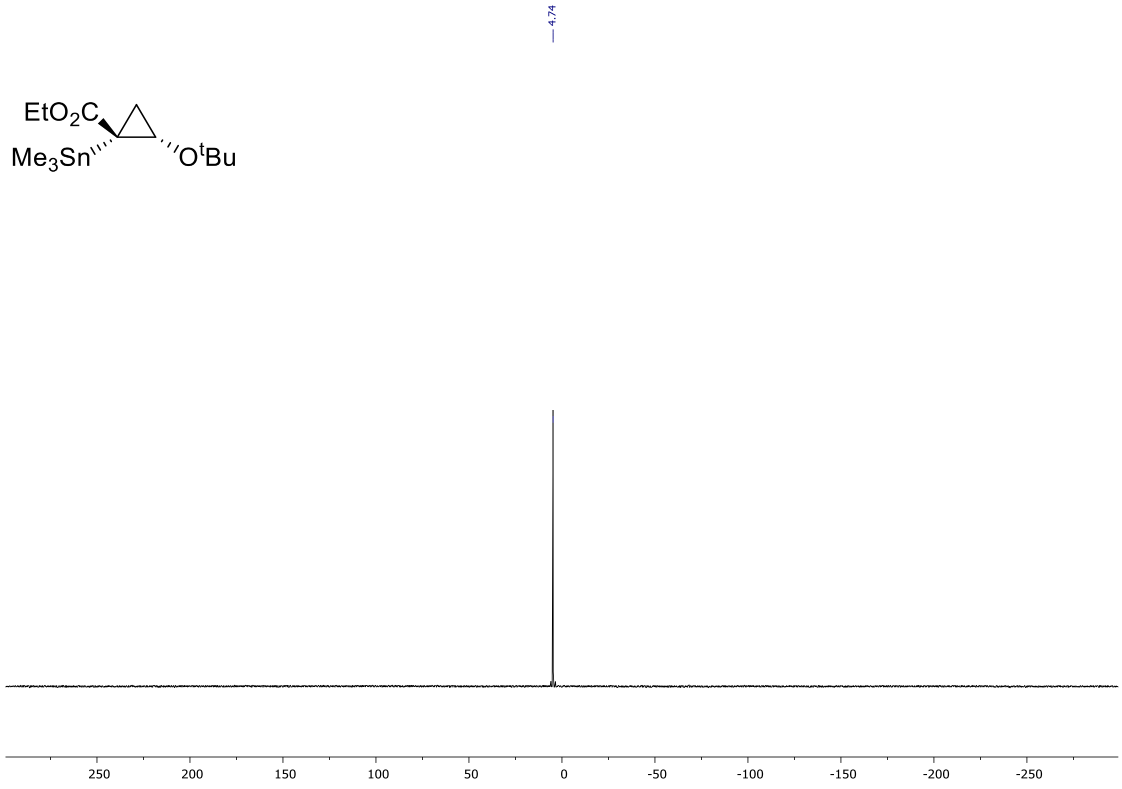

^1H-^1H-NOESY ($CDCl_3$)

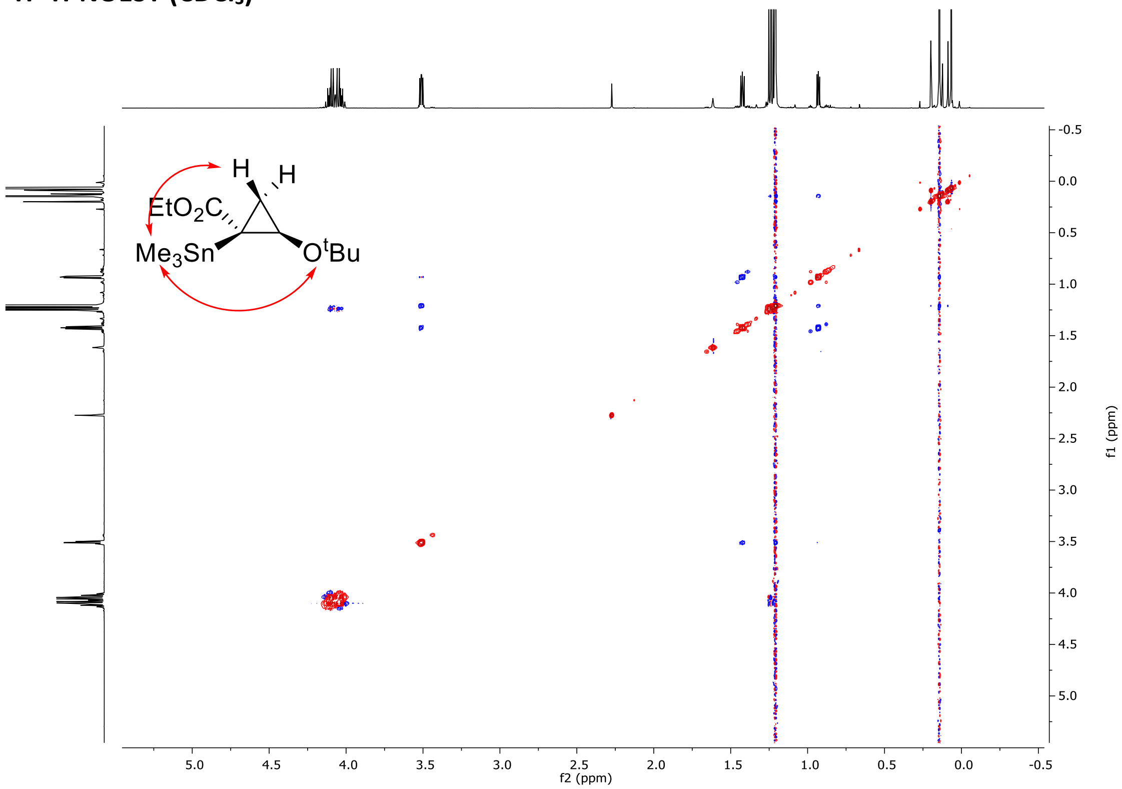

***trans*-2ae: ^{1}H-NMR (400 MHz, $CDCl_3$)**

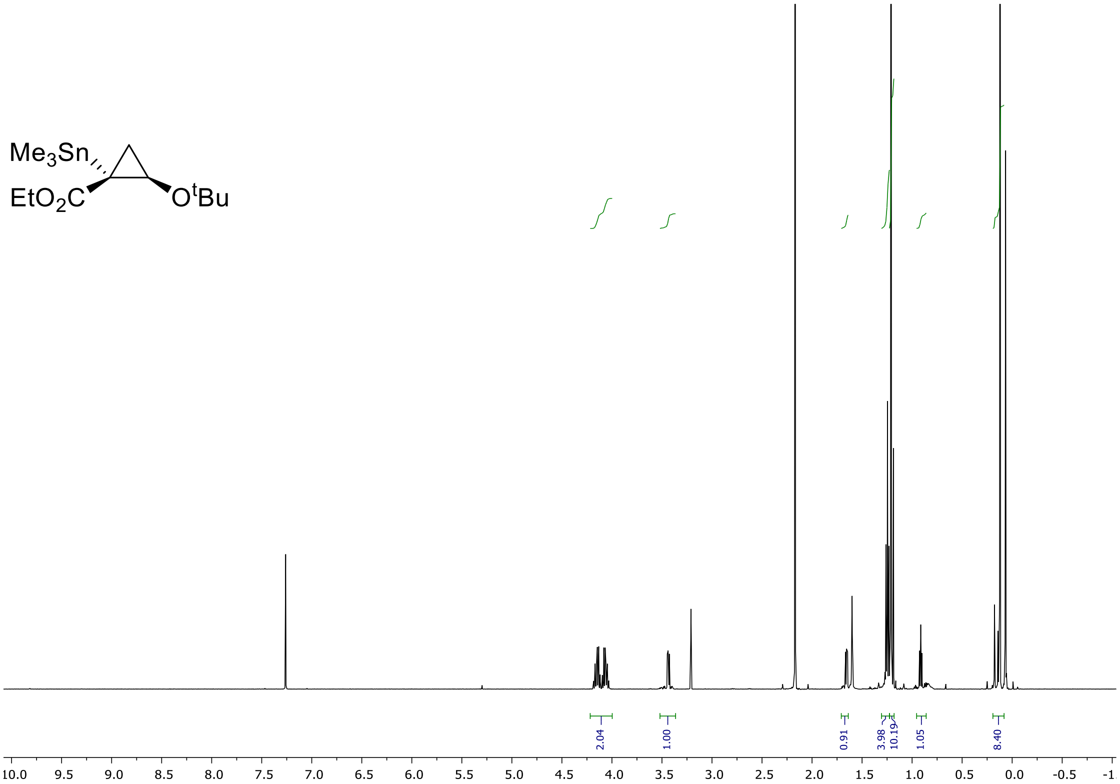

^{13}C{^{1}H}-NMR (100MHz, $CDCl_3$)

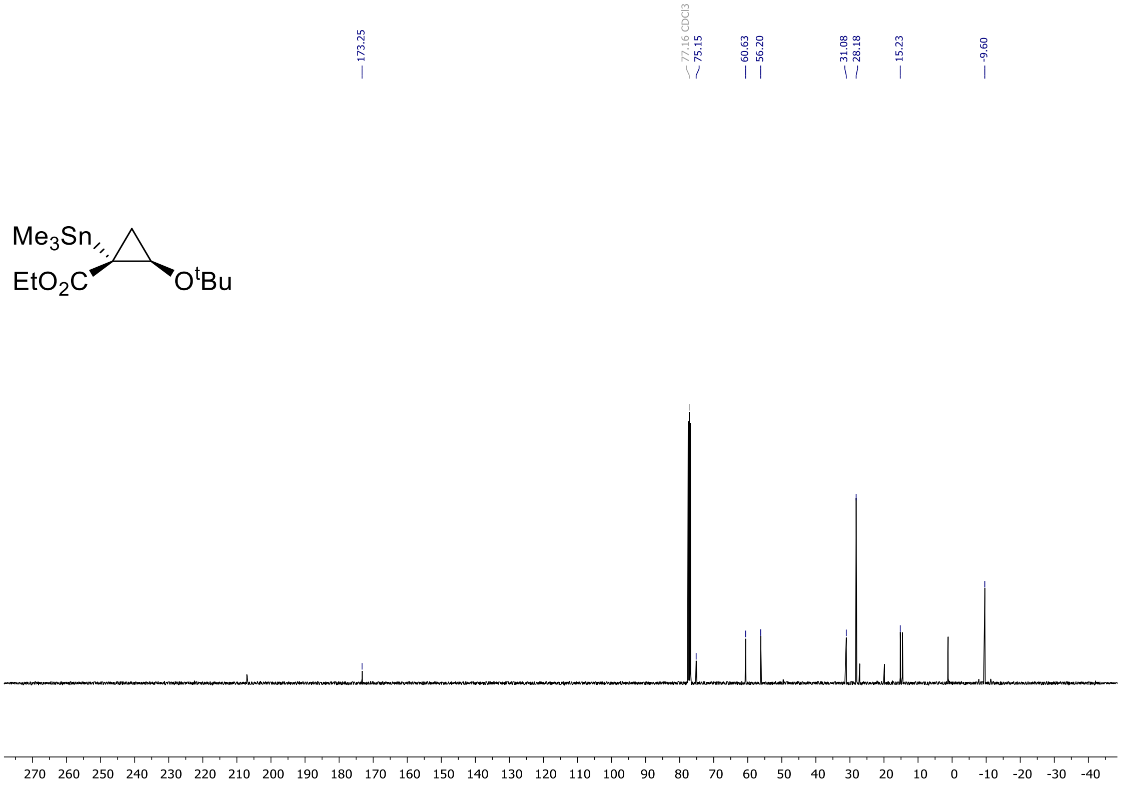

$^{119}Sn\{^1H\}$-NMR (149 MHz, $CDCl_3$)

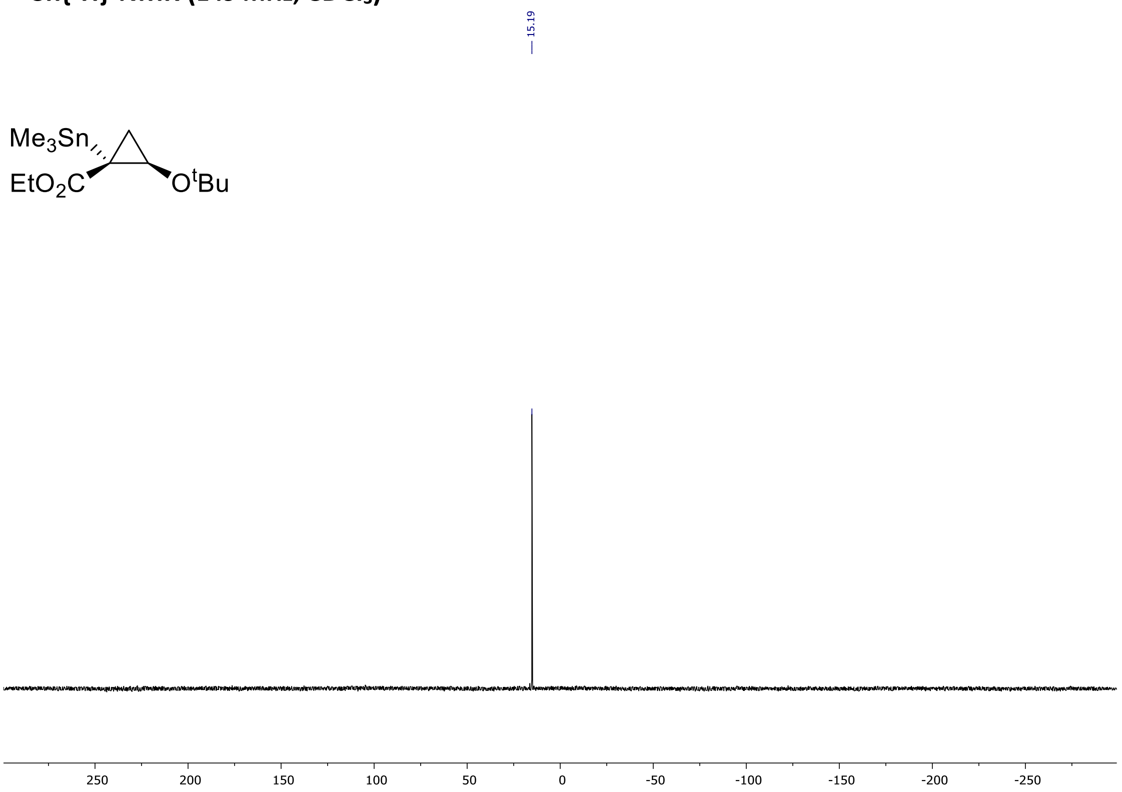

^1H-^1H-NOESY ($CDCl_3$)

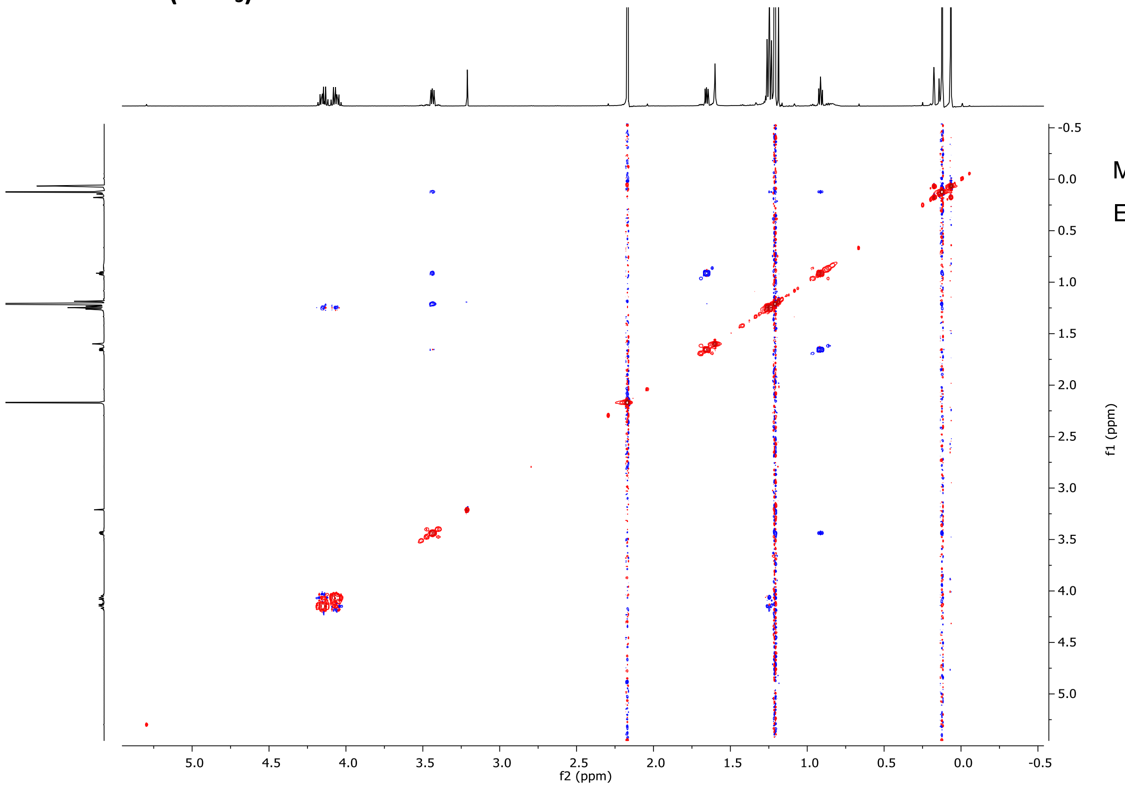

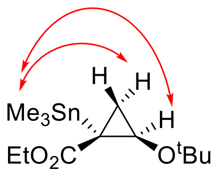

cis-2af: ^{1}H-NMR (400 MHz, $CDCl_3$)

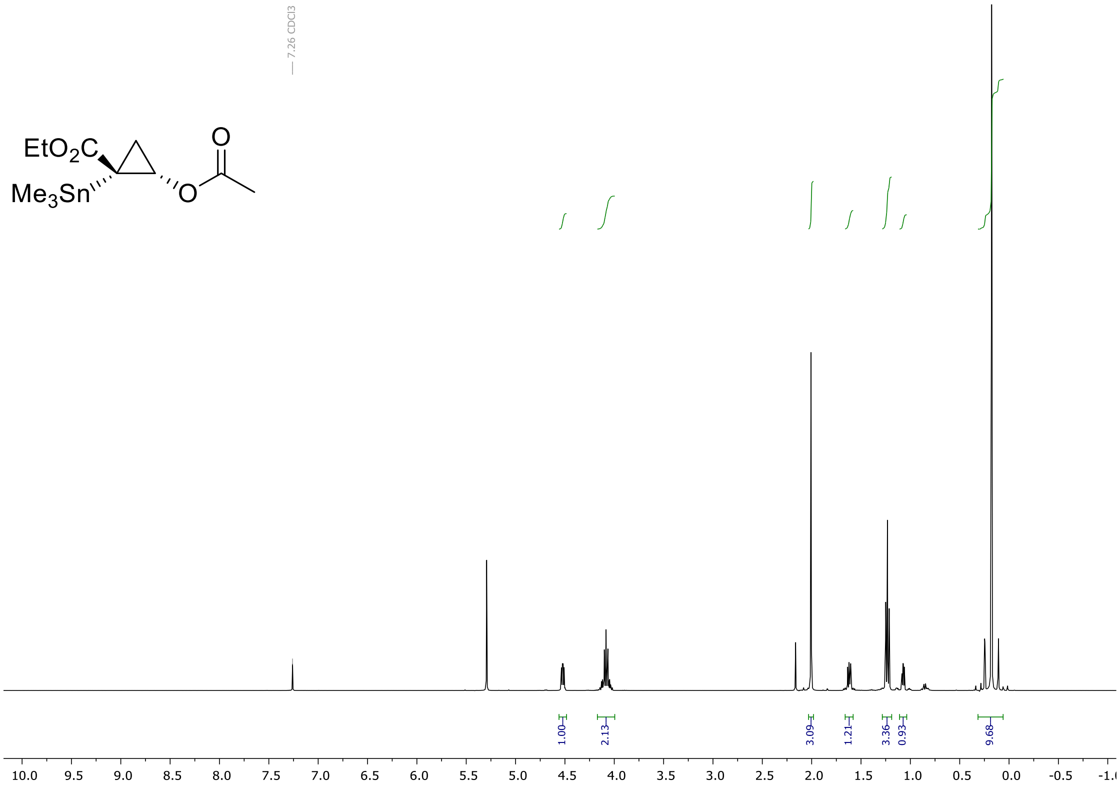

^{13}C-NMR (100 MHz, $CDCl_3$)

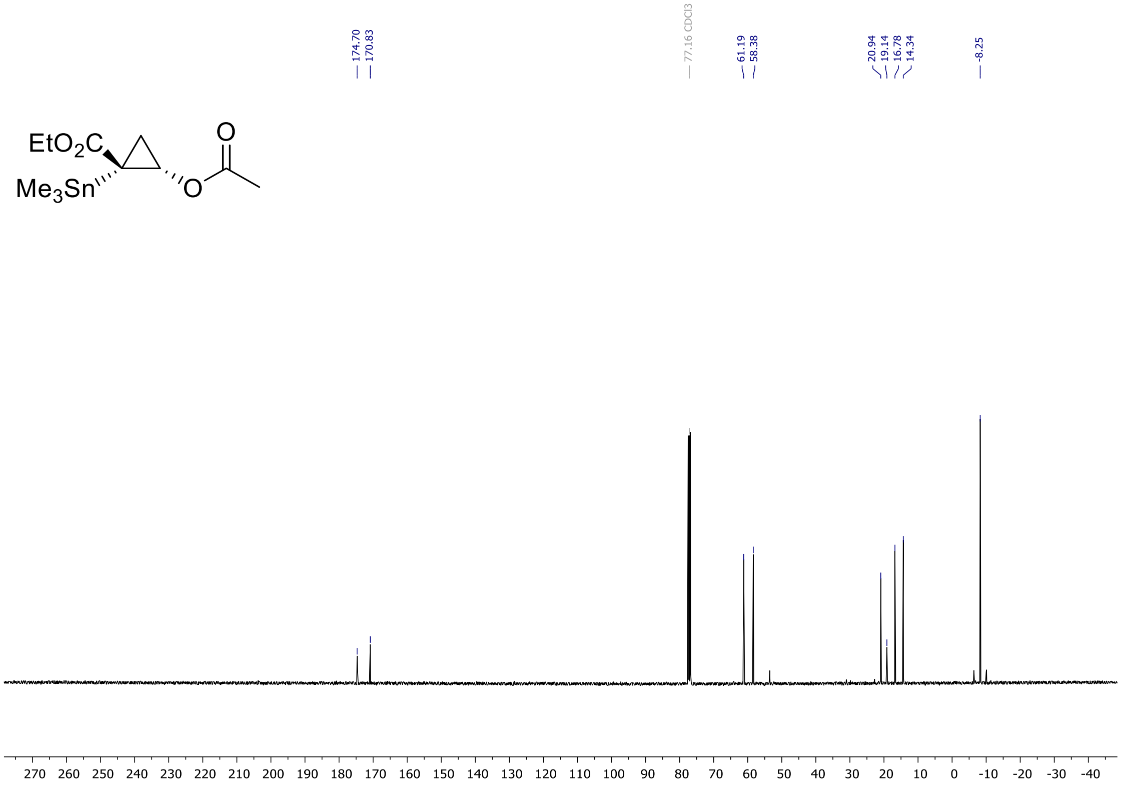

^{119}Sn-NMR (149 MHz, $CDCl_3$)

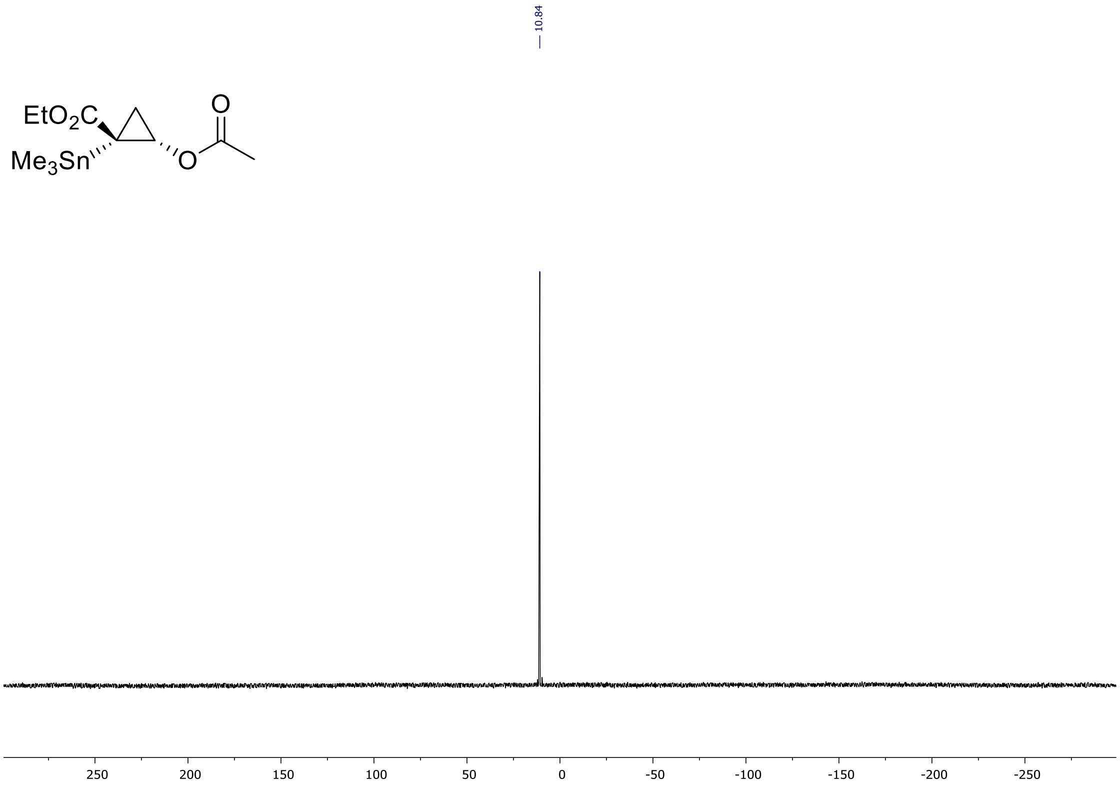

^{1}H-^{1}H NOESY ($CDCl_3$)

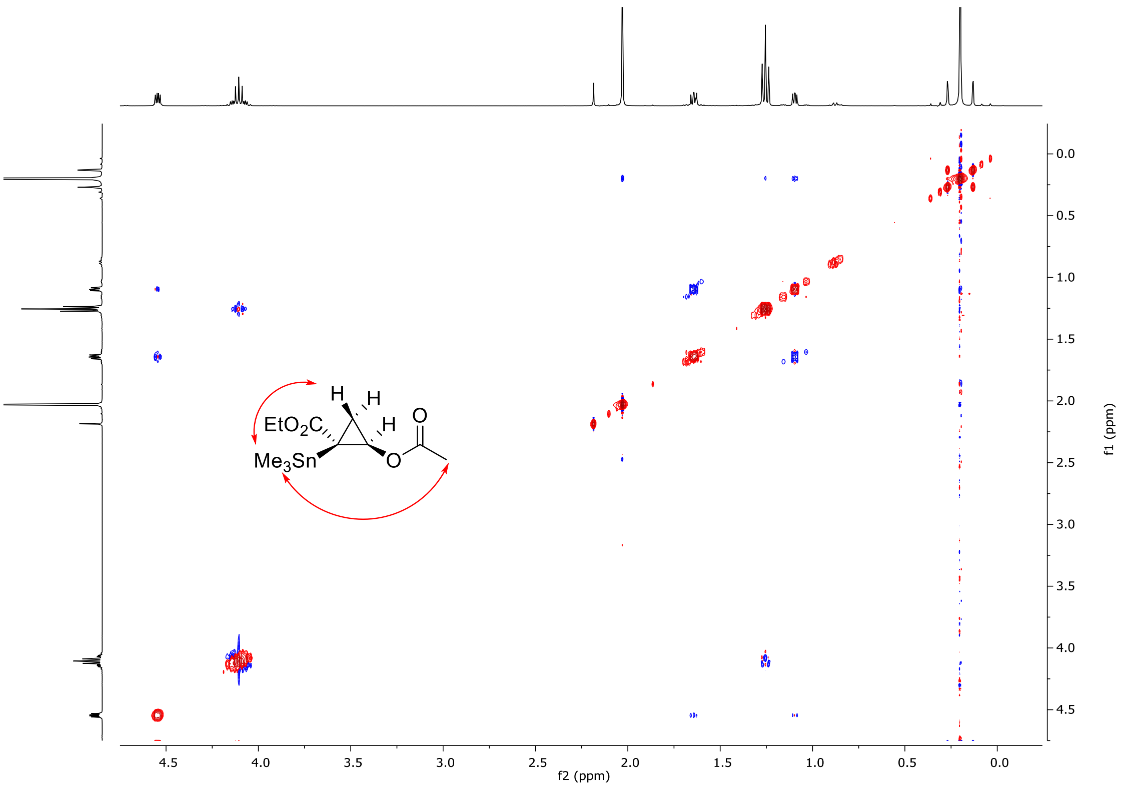

***trans*-2af: ^{1}H-NMR (400 MHz, CDCl$_3$)**

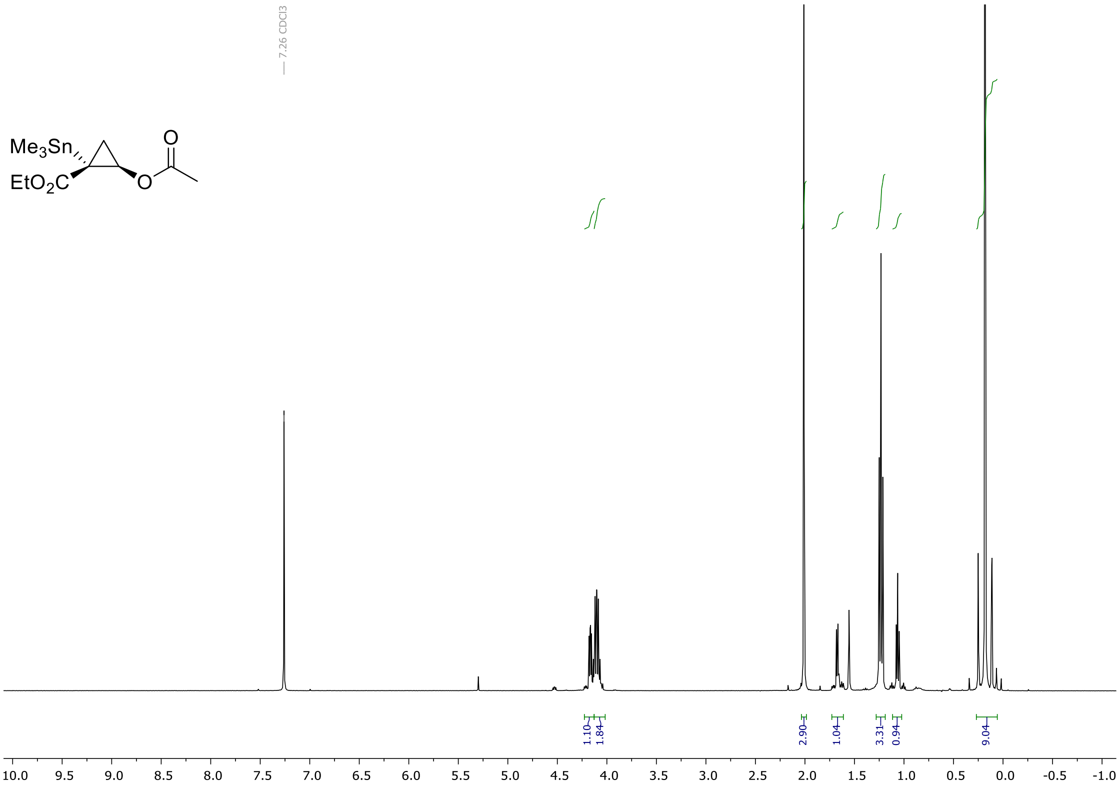

^{13}C-NMR (100 MHz, CDCl$_3$)

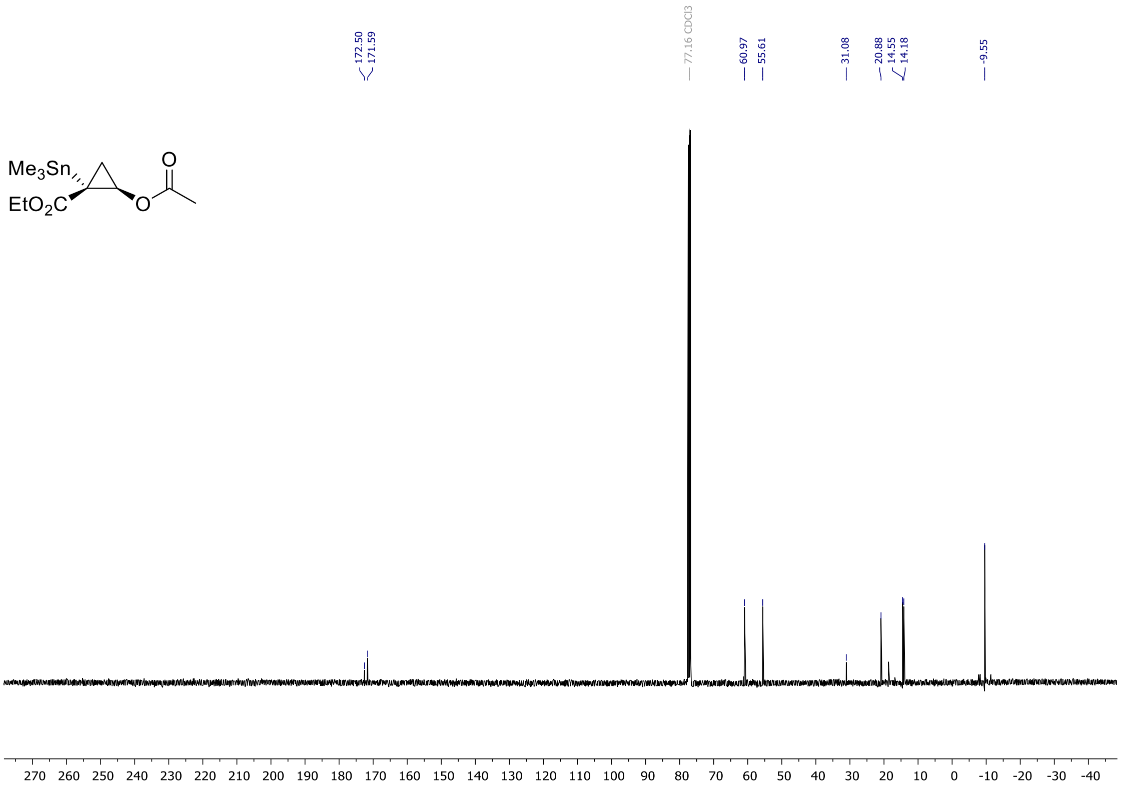

^{119}Sn-NMR (149 MHz, $CDCl_3$)

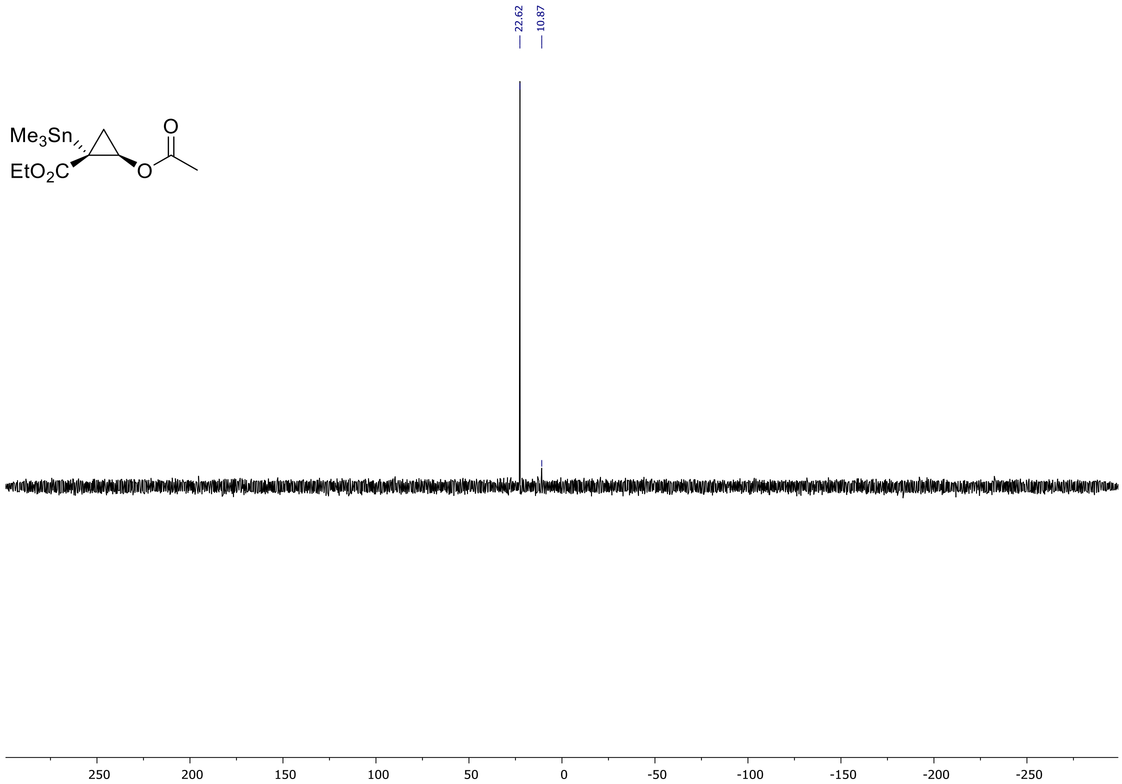

^{1}H-^{1}H NOESY ($CDCl_3$)

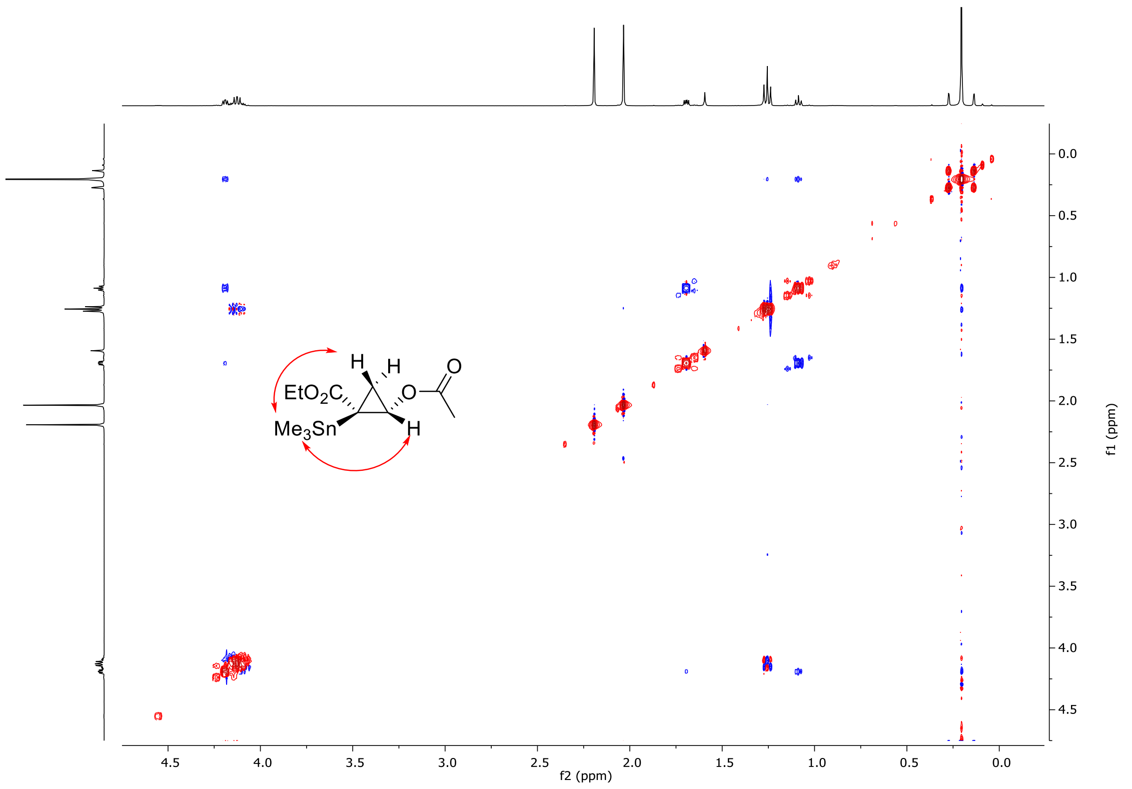

***cis*-2ag: ^{1}H-NMR (400 MHz, CDCl$_3$)**

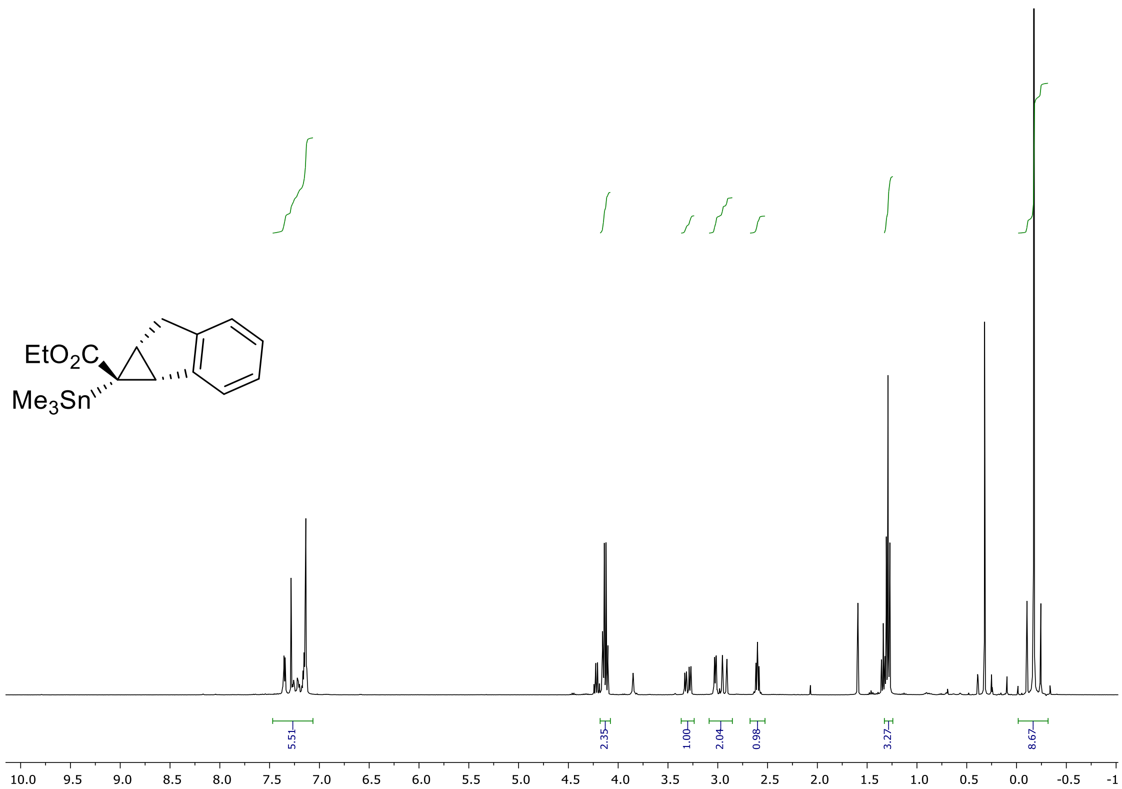

^{13}C-NMR (100 MHz, CDCl$_3$)

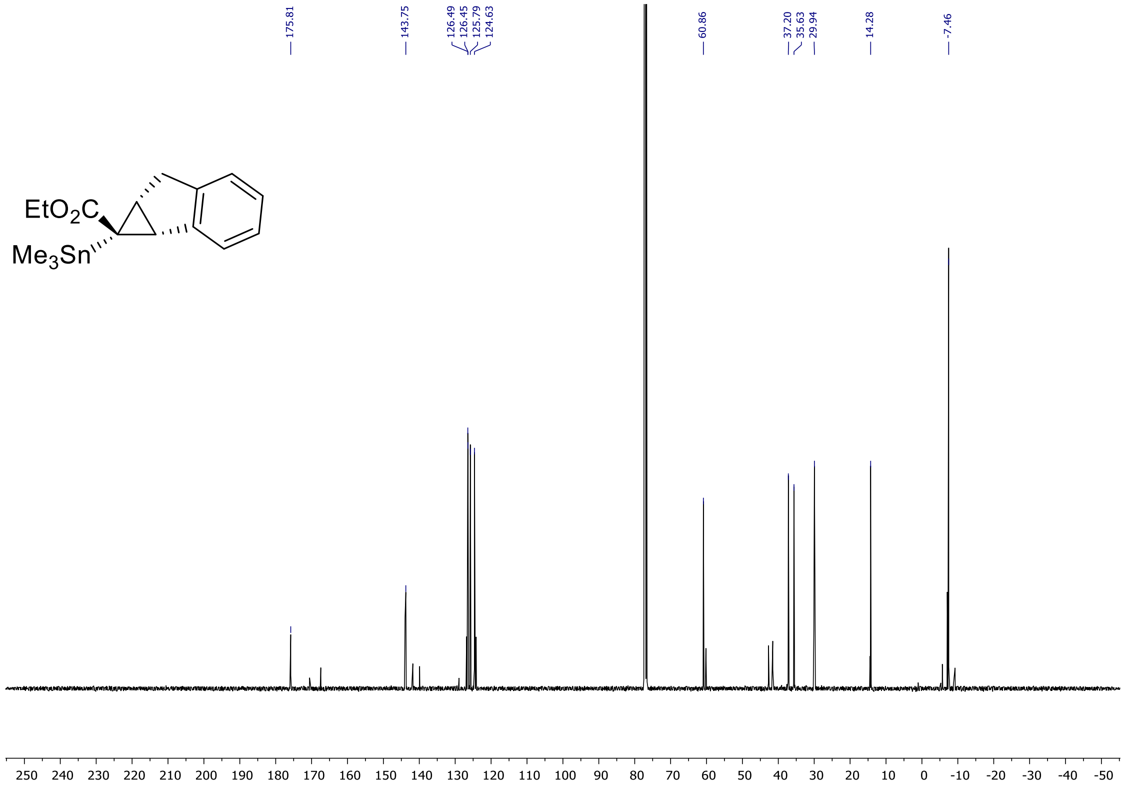

^{119}Sn-NMR (149 MHz, $CDCl_3$)

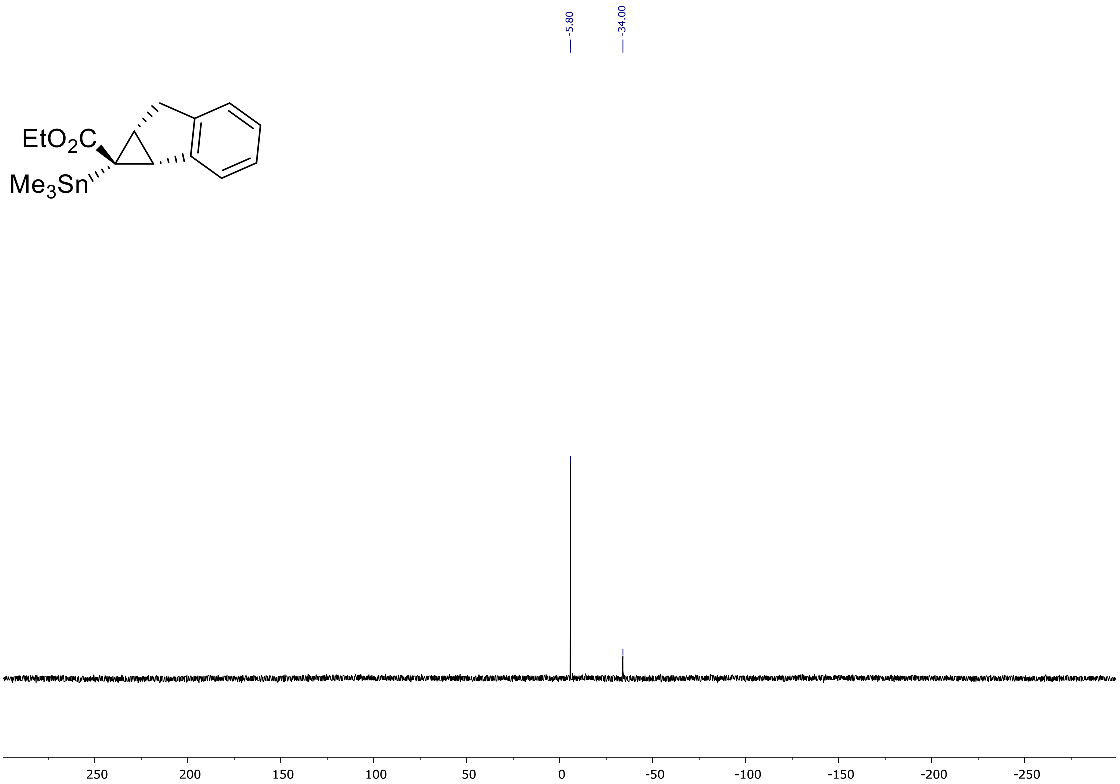

^{1}H-^{1}H NOESY ($CDCl_3$)

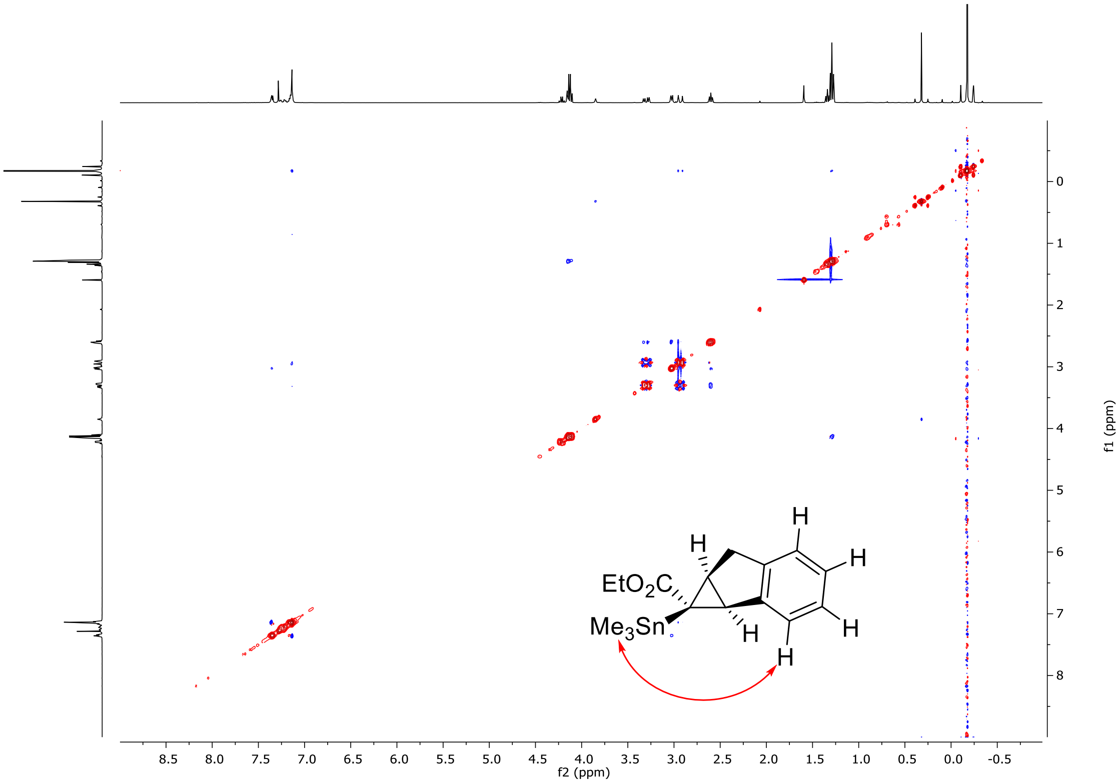

***trans*-2ag: ^{1}H-NMR (400 MHz, $CDCl_3$)**

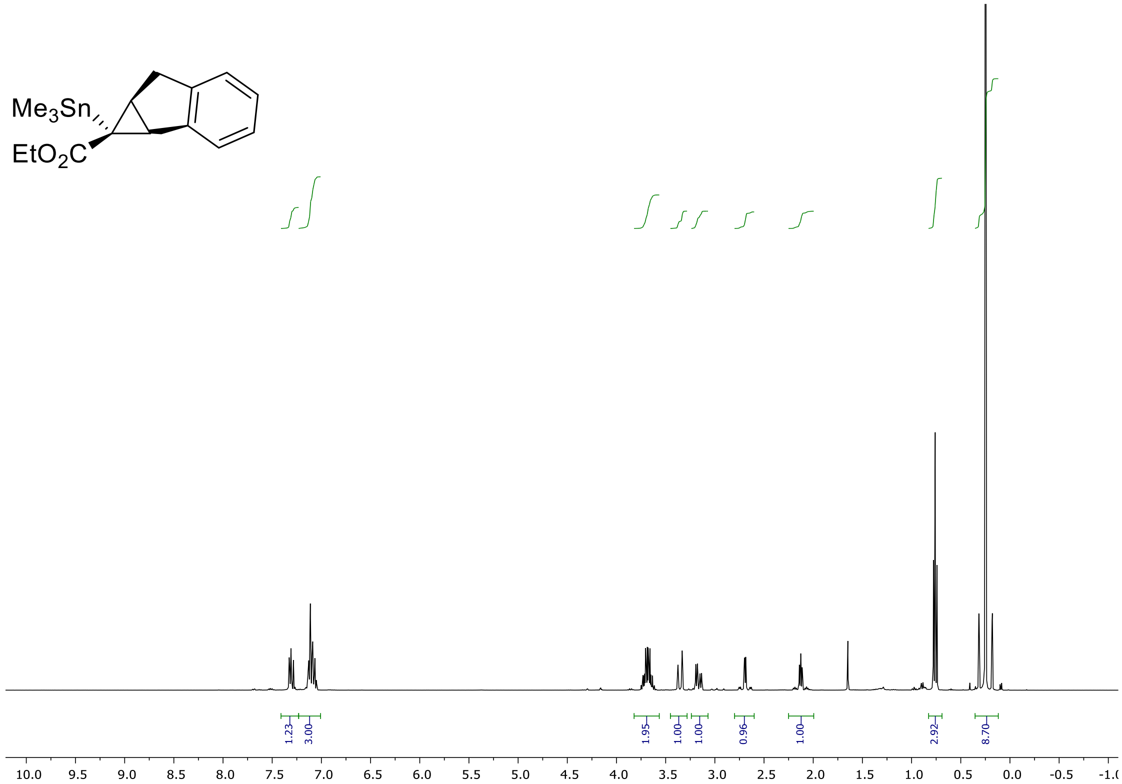

^{13}C-NMR (100 MHz, $CDCl_3$)

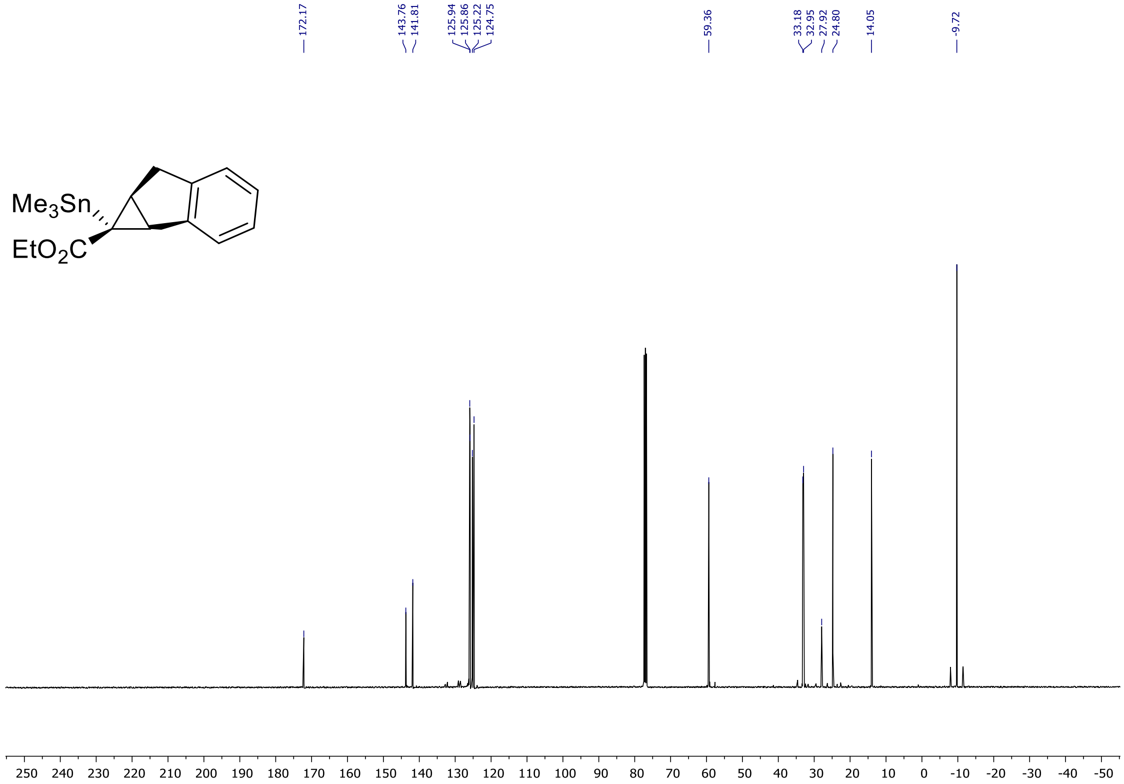

^{119}Sn-NMR (149 MHz, CDCl$_3$)

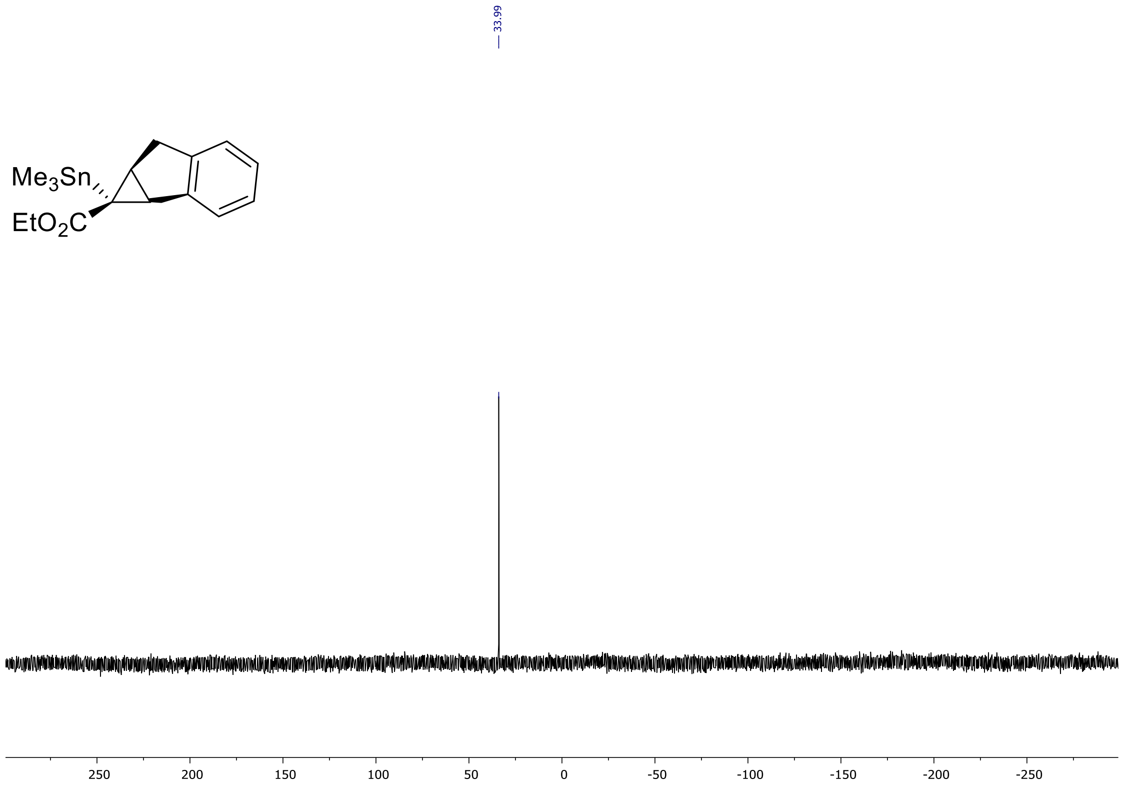

^{1}H-^{1}H NOESY

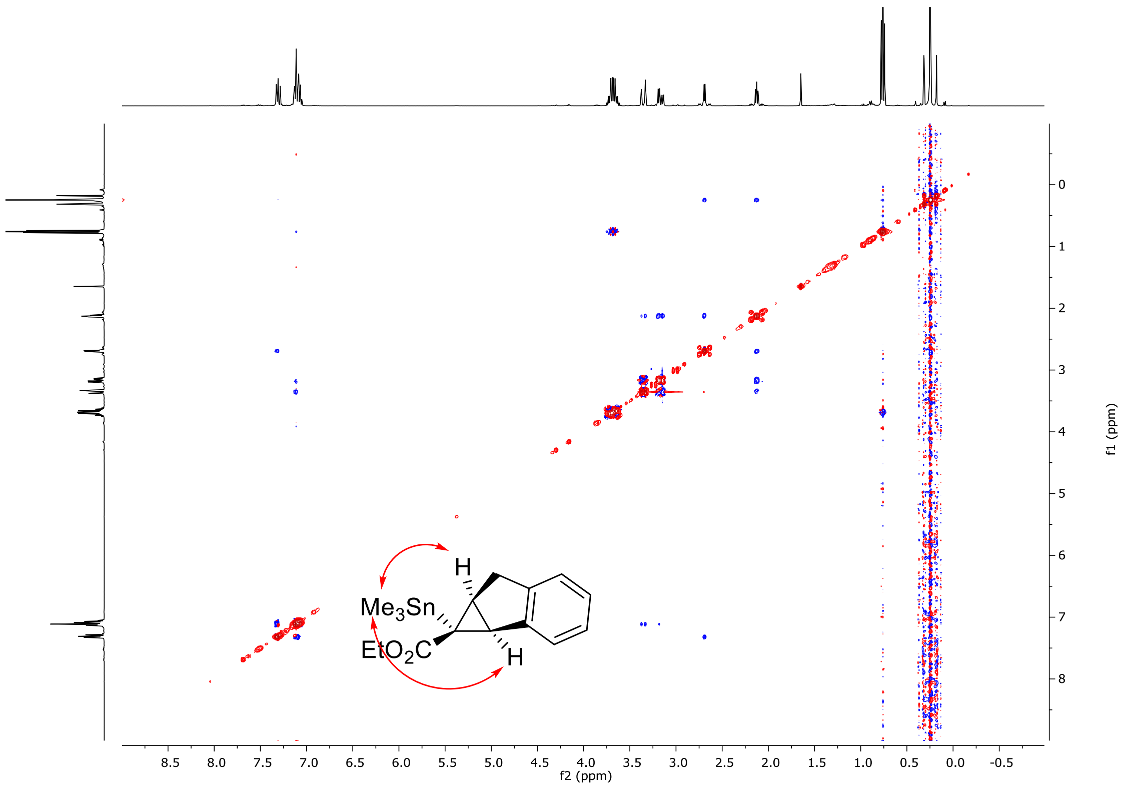

trans-14: ^{1}H-NMR (400 MHz, CDCl$_3$)

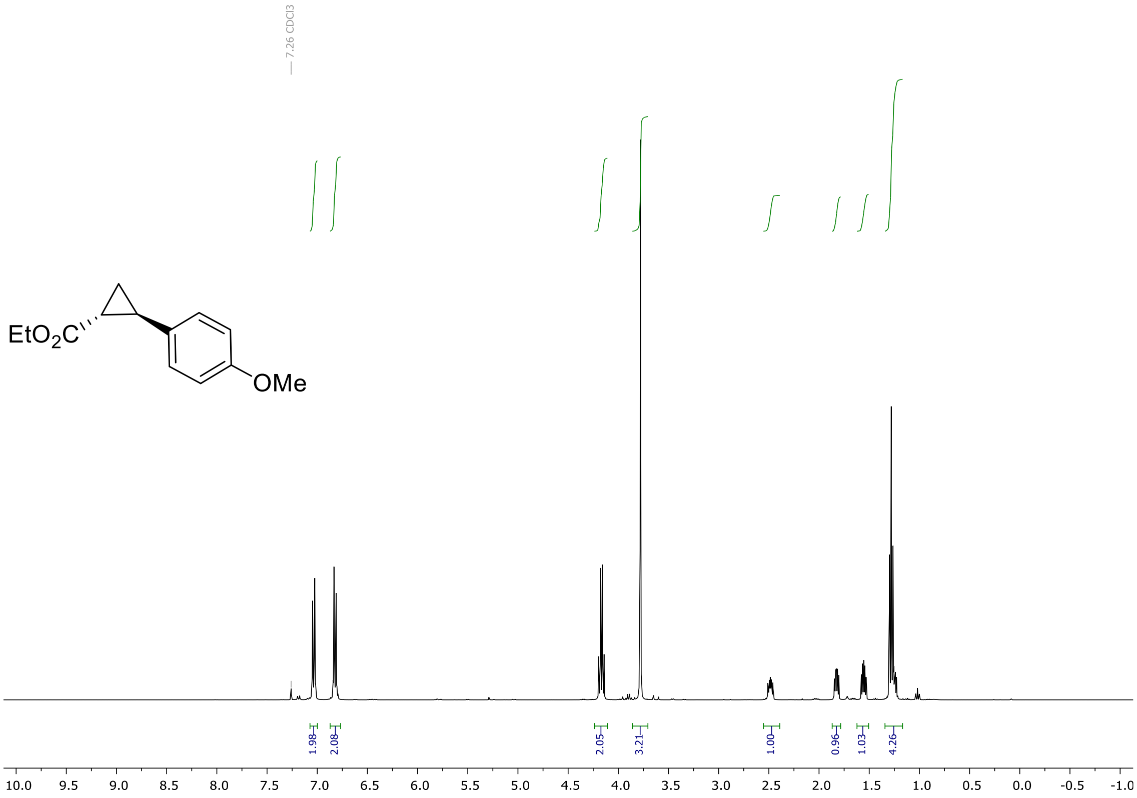

^{13}C-NMR (100 MHz, CDCl$_3$)

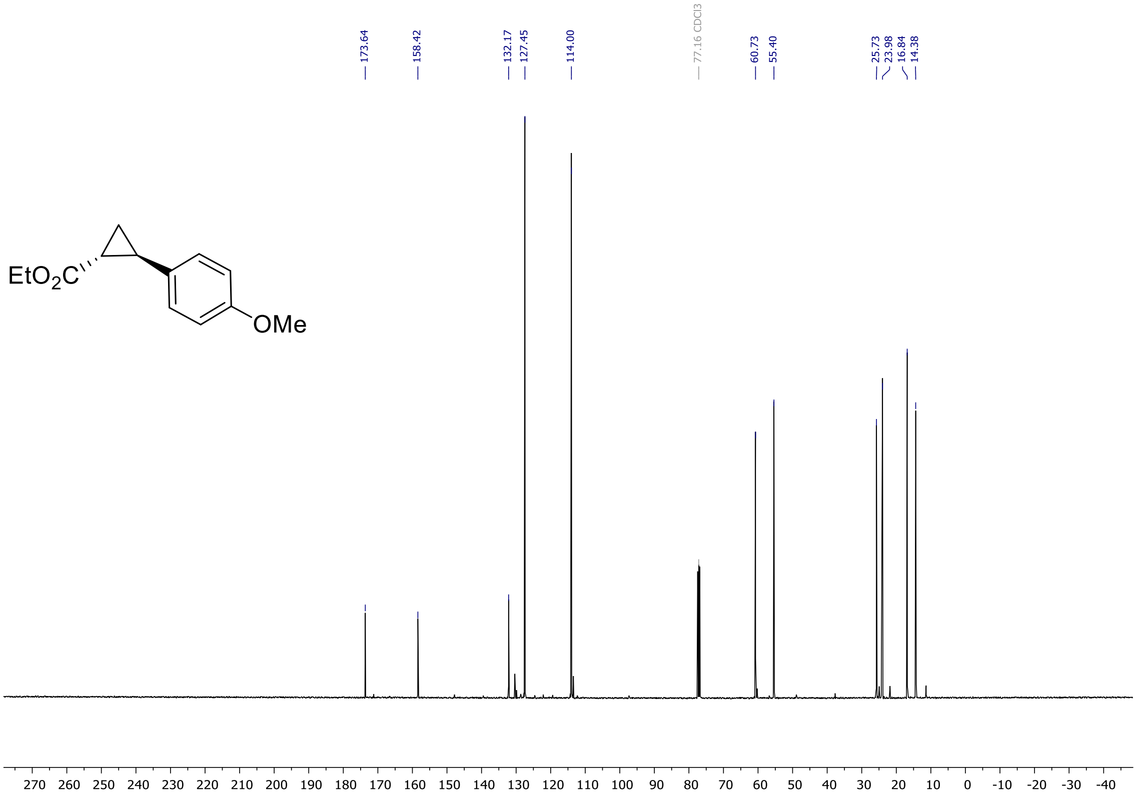

cis-14: ^{1}H-NMR (400 MHz, $CDCl_3$)

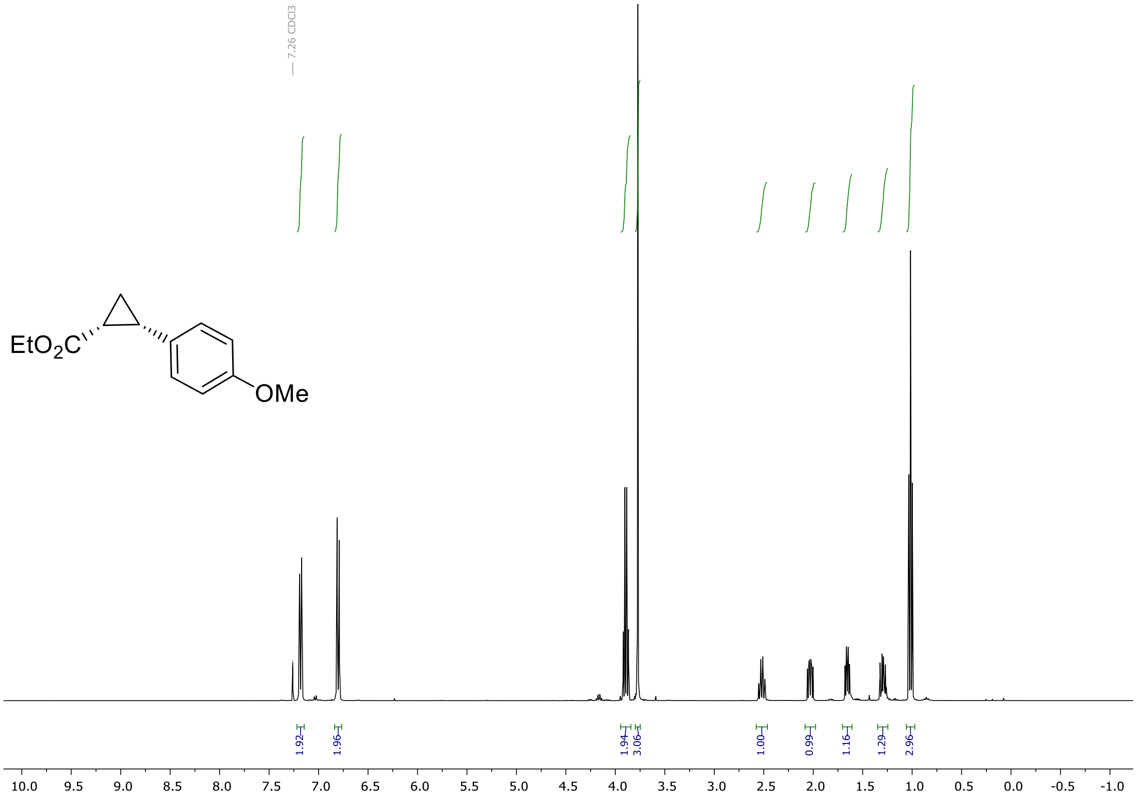

^{13}C-NMR (100 MHz, $CDCl_3$)

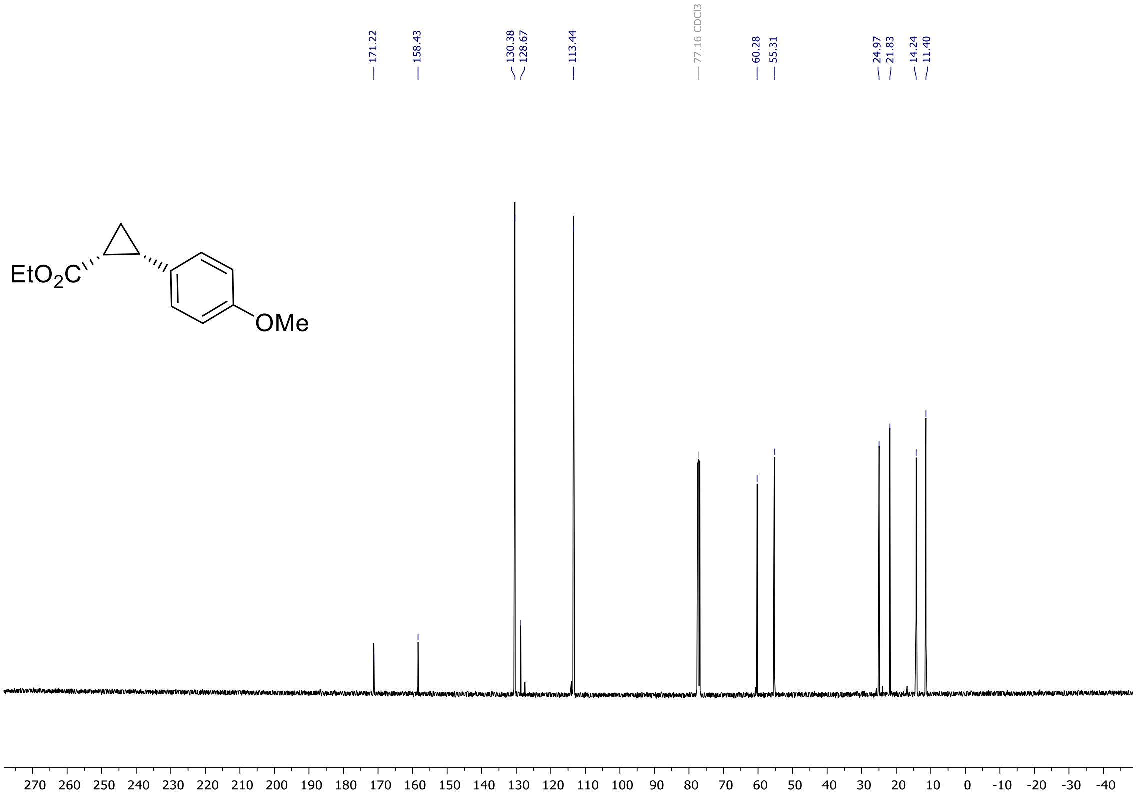

***trans*-13: ^{1}H-NMR (400 MHz, CDCl$_3$)**

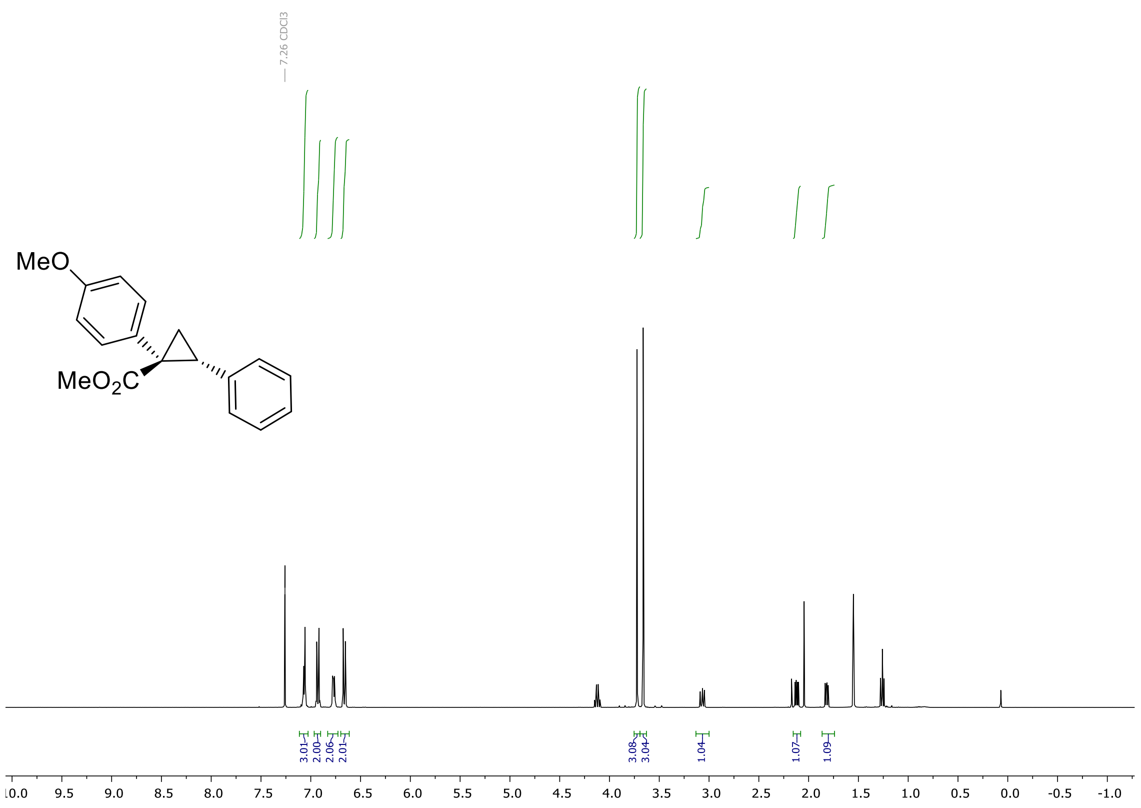

^{13}C-NMR (100 MHz, CDCl$_3$)

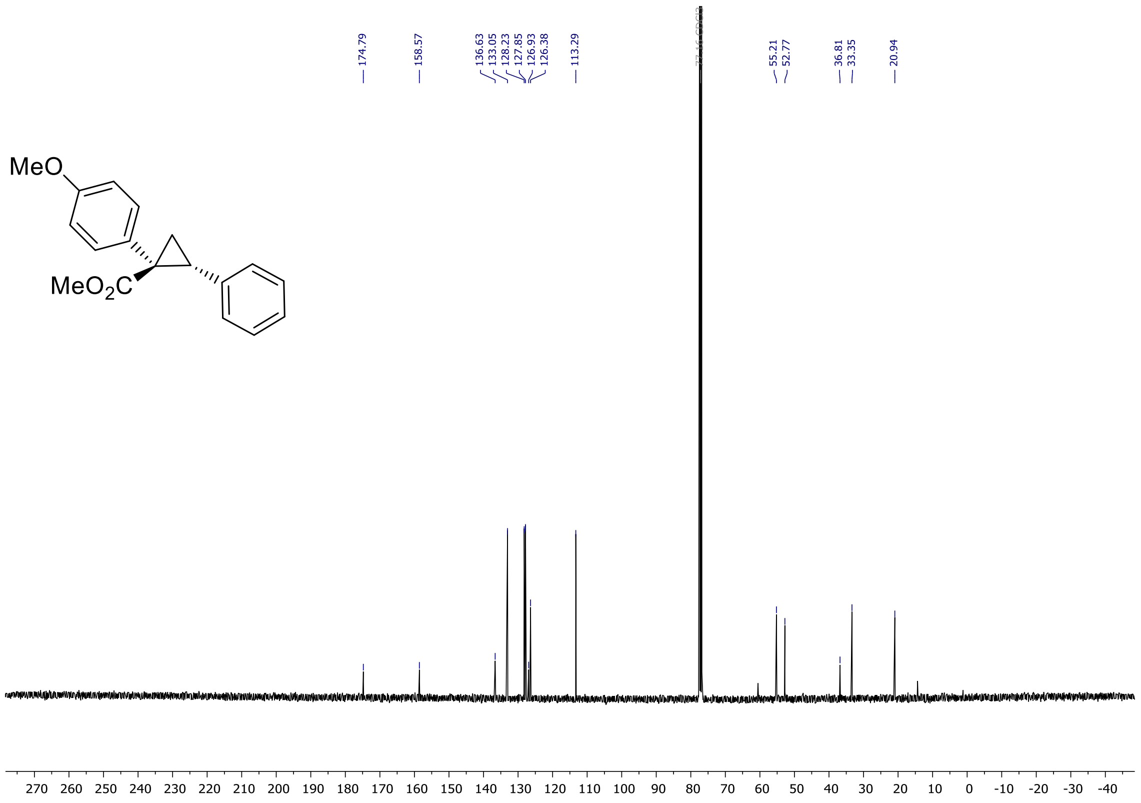

***cis*-15: ^{1}H-NMR (400 MHz, $CDCl_3$)**

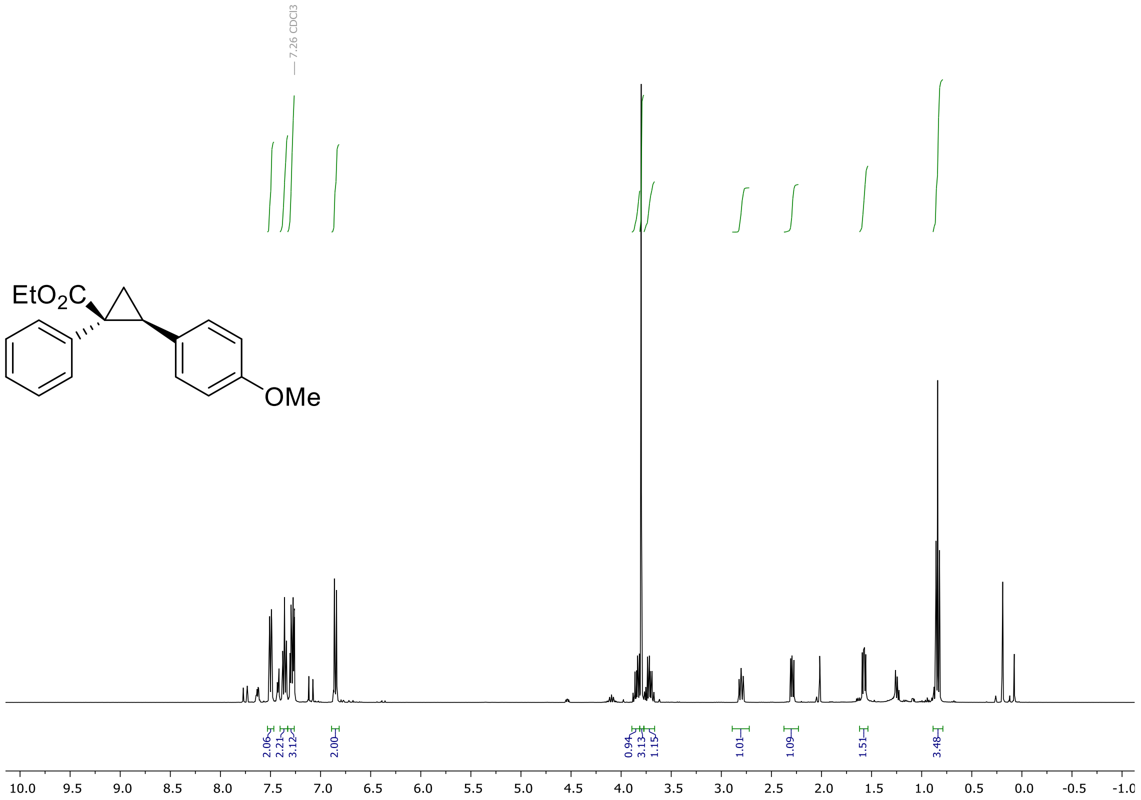

^{13}C-NMR (400 MHz, $CDCl_3$)

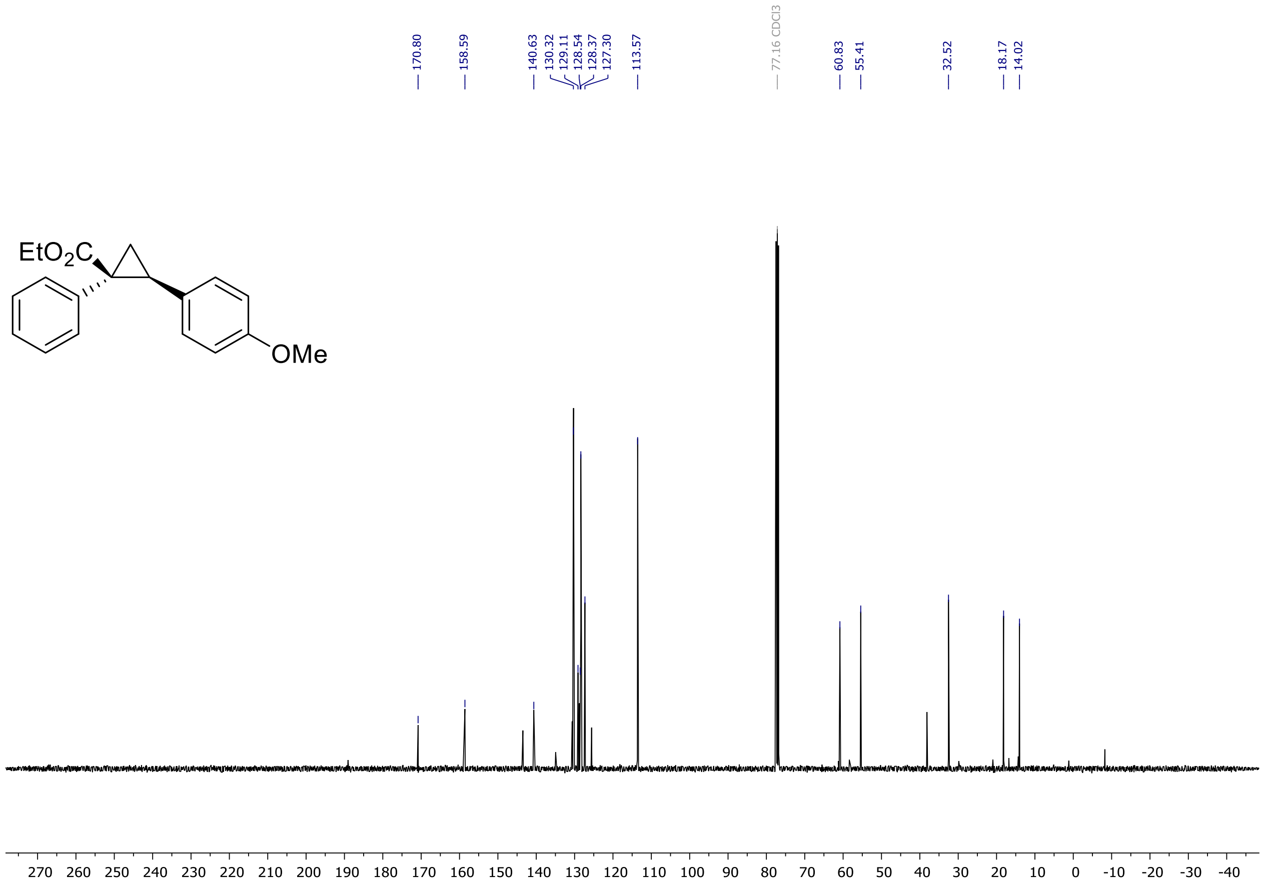

^{1}H-^{1}H-NOESY ($CDCl_3$)

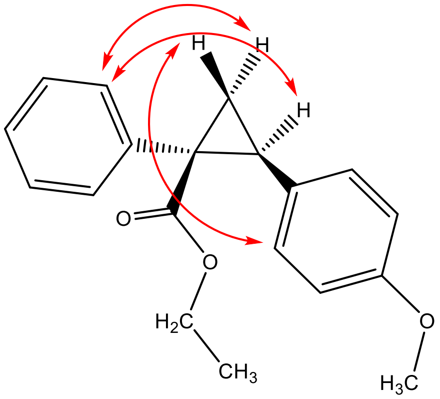

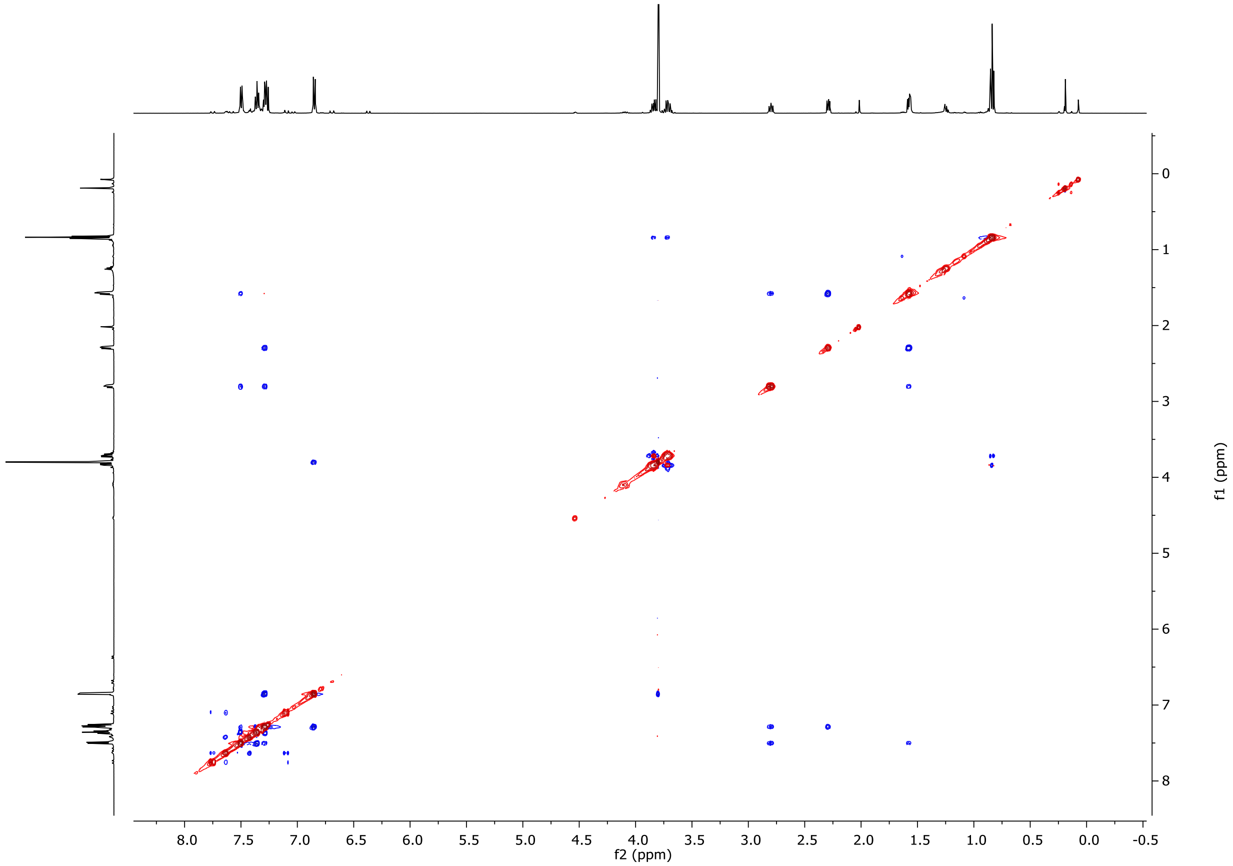

trans-15 ^{1}H-NMR (400 MHz, CDCl$_3$)

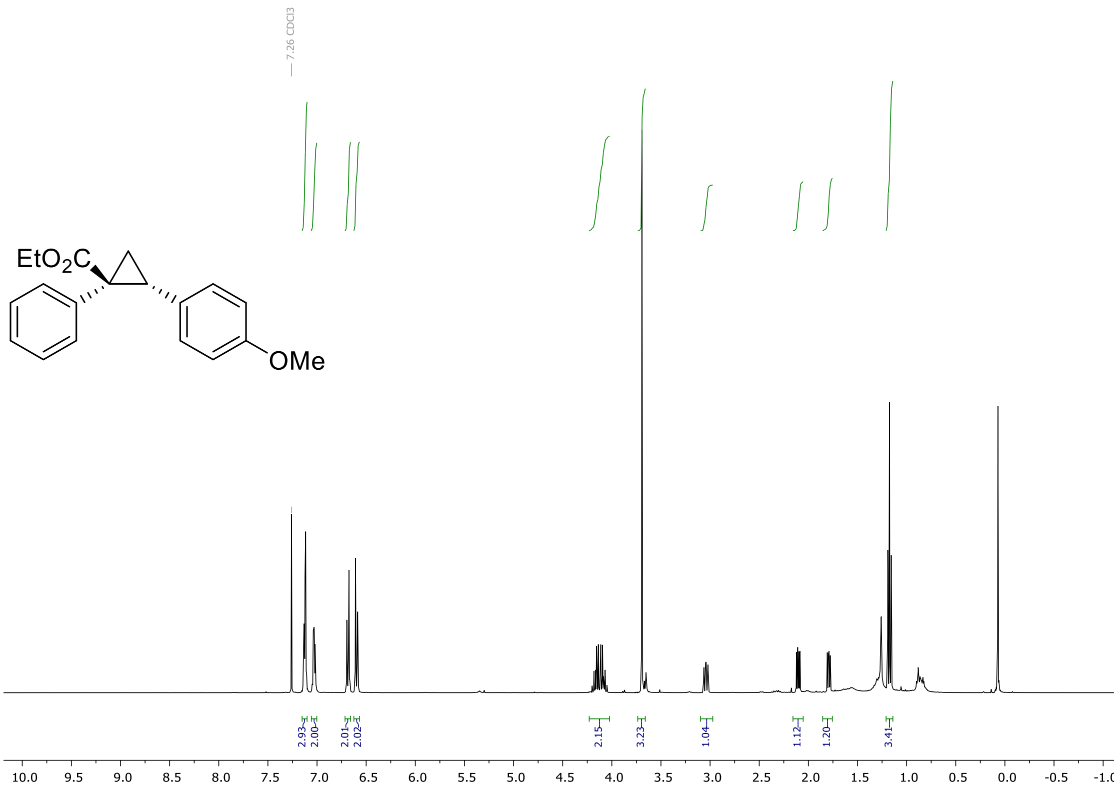

^{13}C-NMR (400 MHz, CDCl$_3$)

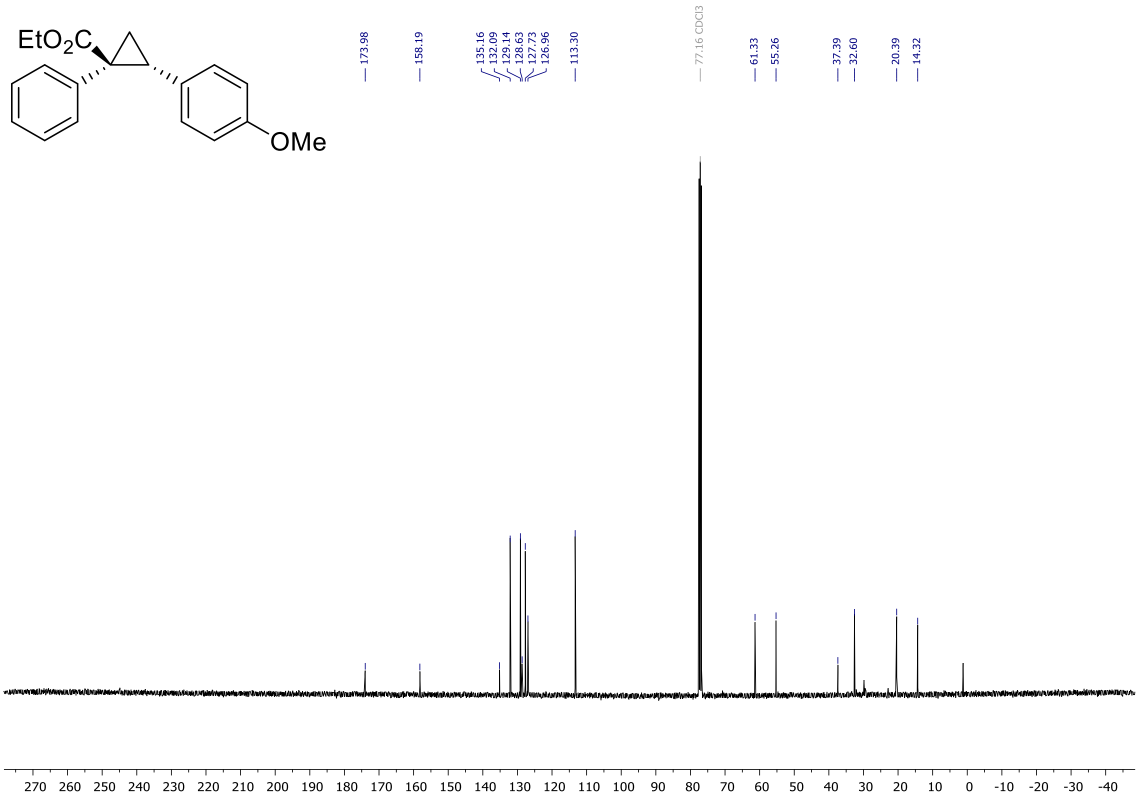

^{1}H-^{1}H-NOESY ($CDCl_3$)

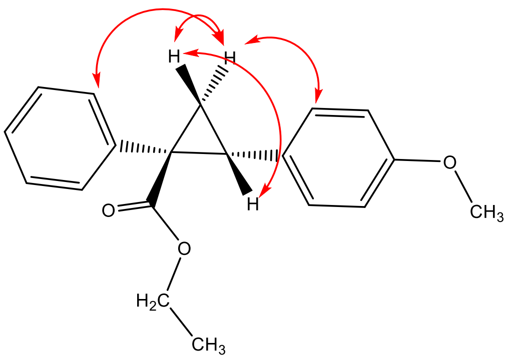

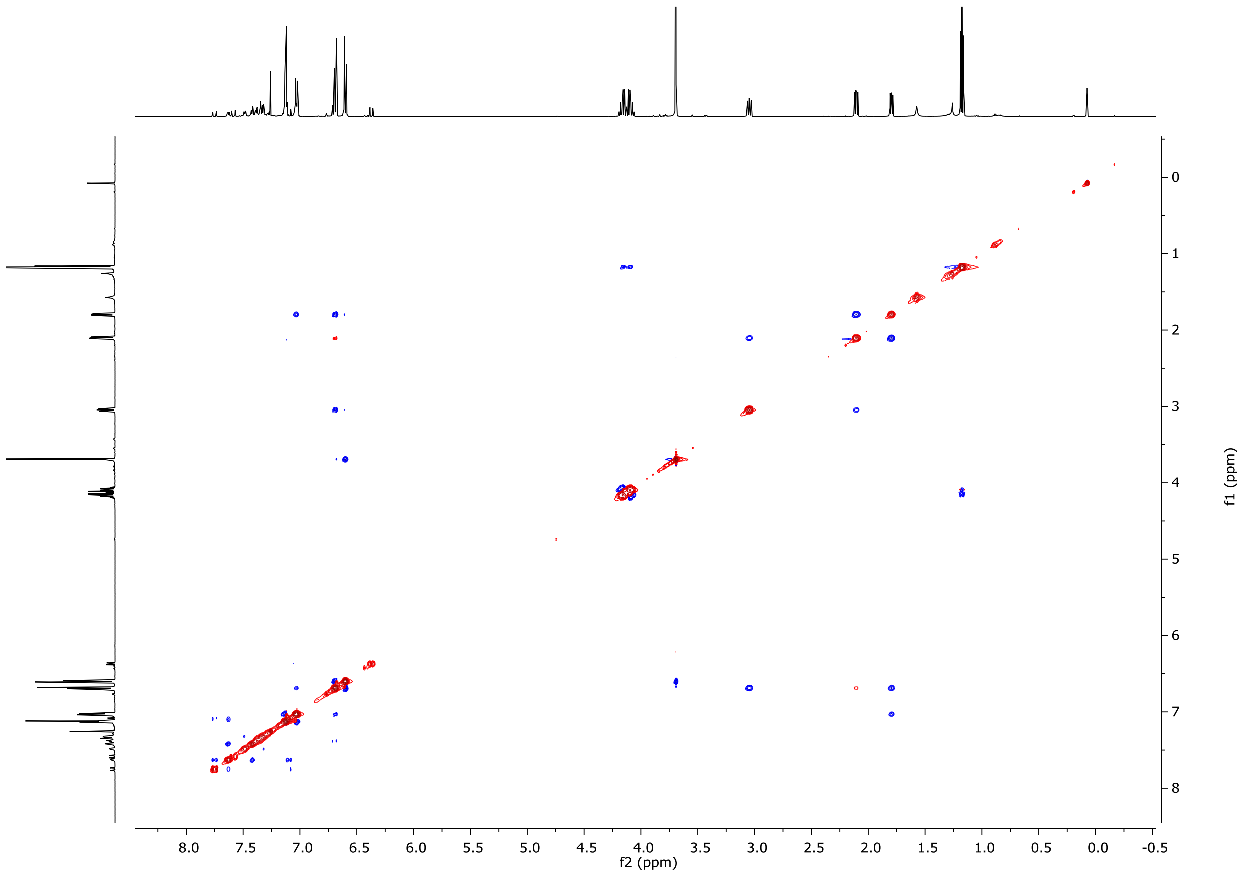

***cis*-16 ^{1}H-NMR (400 MHz, $CDCl_3$)**

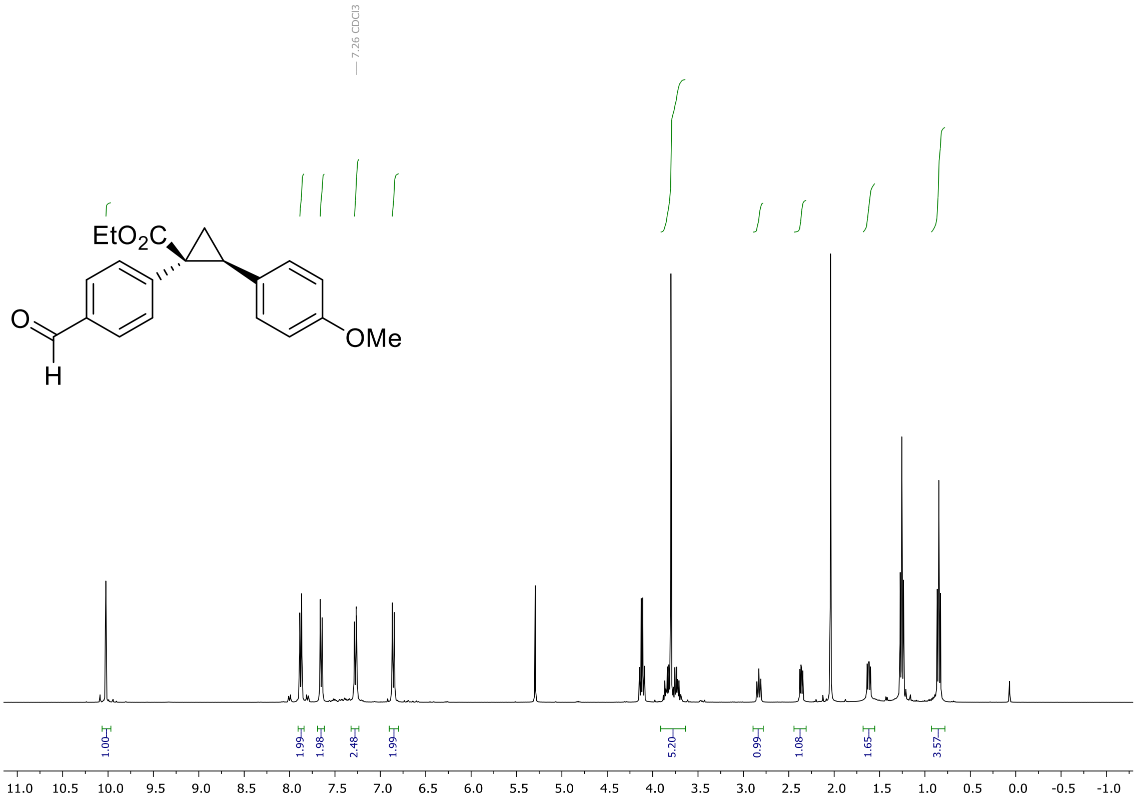

^{13}C-NMR (400 MHz, $CDCl_3$)

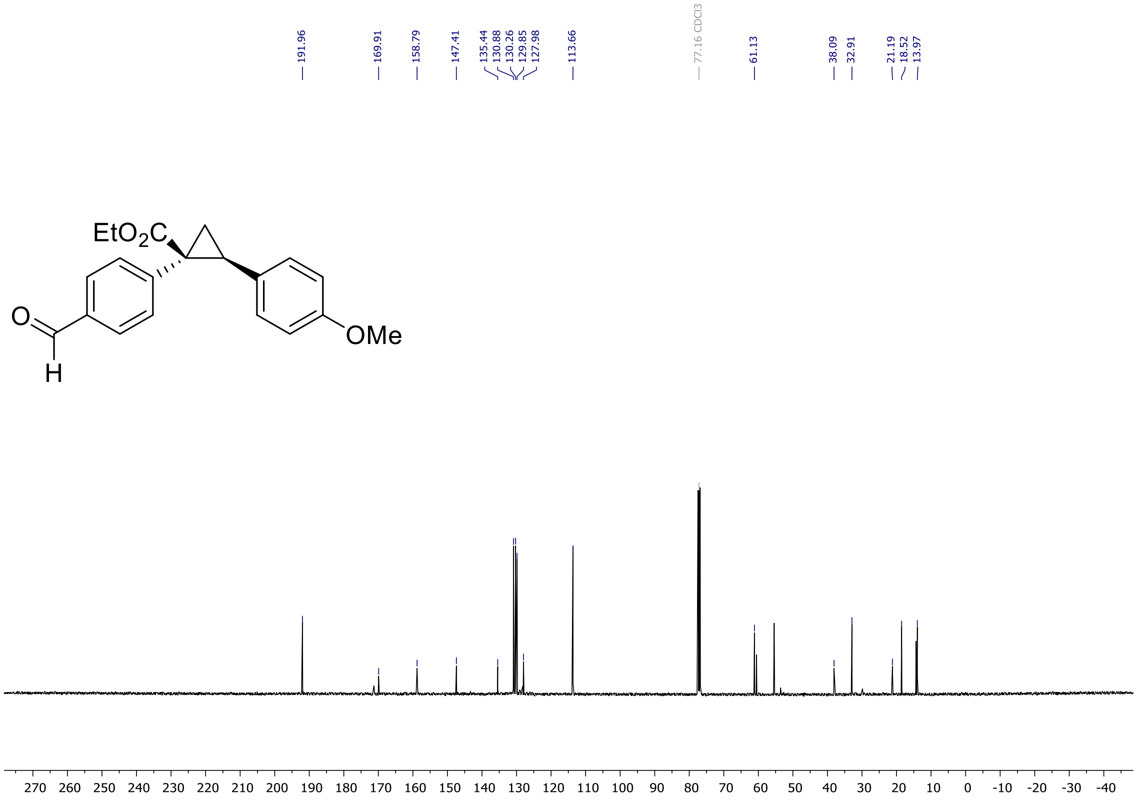

cis-17 ^{1}H-NMR (400 MHz, $CDCl_3$)

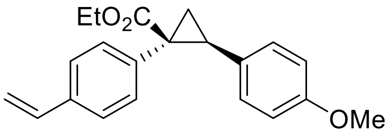

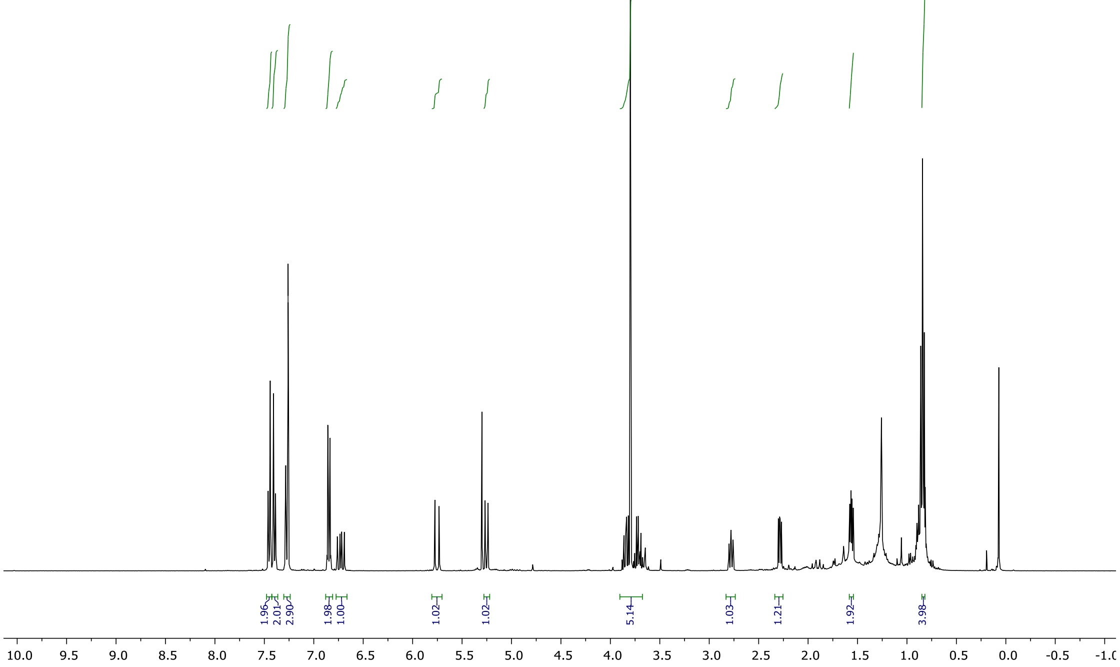

^{13}C-NMR (400 MHz, $CDCl_3$)

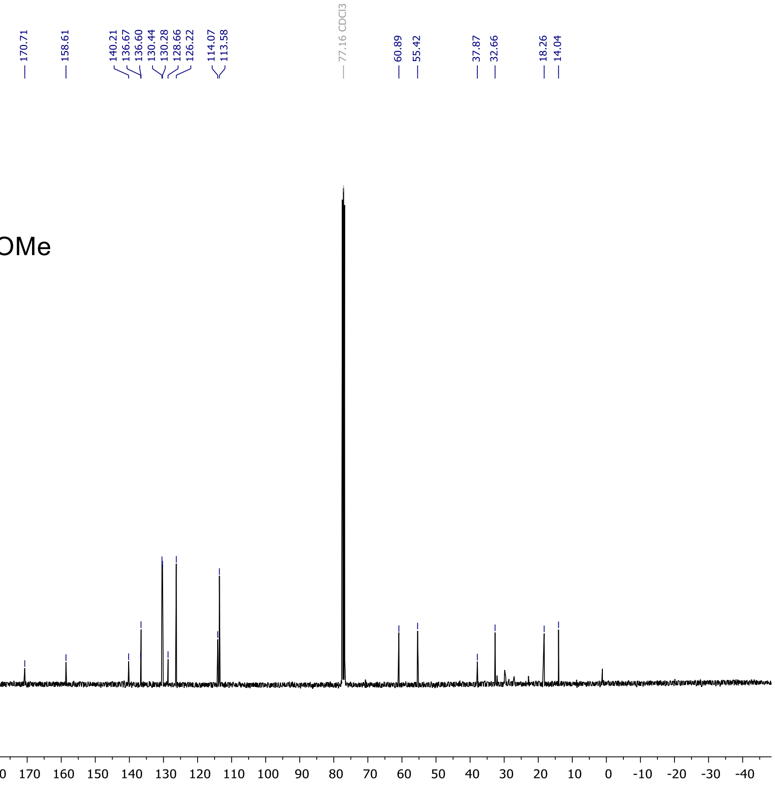

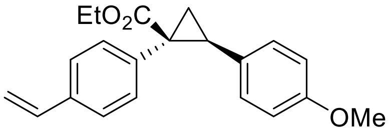

Supplement: Supplementary file 1 — Supplementary [file ANIE-59-13900-s001.pdf]
